# A new archosauriform species from the Panchet Formation of India and the diversification of Proterosuchidae after the end-Permian mass extinction

**DOI:** 10.1098/rsos.230387

**Published:** 2023-10-25

**Authors:** Martín D. Ezcurra, Saswati Bandyopadhyay, Dhurjati P. Sengupta, Kasturi Sen, Andrey G. Sennikov, Roland B. Sookias, Sterling J. Nesbitt, Richard J. Butler

**Affiliations:** ^1^ Sección Paleontología de Vertebrados, CONICET−Museo Argentino de Ciencias Naturales ‘Bernardino Rivadavia’, Ciudad Autónoma de Buenos Aires, Argentina; ^2^ School of Geography, Earth and Environmental Sciences, University of Birmingham, Edgbaston, Birmingham, UK; ^3^ Geological Studies Unit, Indian Statistical Institute, 203 B. T. Road, Kolkata 700108, India; ^4^ Borissiak Paleontological Institute RAS, Moscow, Russia; ^5^ Evolution and Diversity Dynamics laboratory, Département de Géologie, Université de Liège, Liege, Belgium; ^6^ Department of Geosciences, Virginia Tech, Blacksburg, VA, USA

**Keywords:** Archosauromorpha, Proterosuchidae, Induan, Mesozoic, phylogeny, biogeography

## Abstract

Proterosuchidae represents the oldest substantial diversification of Archosauromorpha and plays a key role in understanding the biotic recovery after the end-Permian mass extinction. Proterosuchidae was long treated as a wastebasket taxon, but recent revisions have reduced its taxonomic content to five valid species from the latest Permian of Russia and the earliest Triassic (Induan) of South Africa and China. In addition to these occurrences, several isolated proterosuchid bones have been reported from the Induan Panchet Formation of India for over 150 years. Following the re-study of historical specimens and newly collected material from this unit, we erect the new proterosuchid species *Samsarasuchus pamelae*, which is represented by most of the presacral vertebral column. We also describe cf. proterosuchid and proterosuchid cranial, girdle and limb bones that are not referred to *Samsarasuchus pamelae*. Phylogenetic analyses recovered *Samsarasuchus pamelae* within the new proterosuchid clade Chasmatosuchinae. The taxonomic diversity of Proterosuchidae is substantially expanded here, with at least 11 nominal species and several currently unnamed specimens, and a biogeographical range encompassing present-day South Africa, China, Russia, India, Brazil, Uruguay and Australia. This indicates a broader taxonomic, phylogenetic and biogeographic diversification of Proterosuchidae than previously thought in the aftermath of the end-Permian mass extinction.

## 1. Introduction

The end-Permian mass extinction is the most severe extinction event documented in the fossil record [1]. Although this biotic crisis apparently had more pronounced consequences in marine ecosystems, it also resulted in a major turnover of terrestrial biotic assemblages [2–4]. On land, tetrapod assemblages that were taxonomically and numerically dominated by therapsid synapsids and parareptiles during the late Palaeozoic Era were replaced by assemblages dominated by diapsid reptiles during the Triassic Period [3,5]. The empty ecospace that was freed up by the end-Permian mass extinction probably allowed the evolutionary radiation of archosauromorph diapsids (crocodylians, dinosaurs and all taxa closer to them than to lepidosaurs), which is considered one of the most spectacular morphological diversifications documented in the vertebrate fossil record (e.g. [6]). Triassic archosauromorphs flourished on land, displaying disparate body plans that included bulky specialized herbivores (e.g. rhynchosaurs, most allokotosaurs), lizard-like small- to medium-sized predators (e.g. some tanystropheids, prolacertids, proterosuchids), bulky hypercarnivores with huge heads (i.e. erythrosuchids), semi-aquatic to fully aquatic forms (e.g. some tanystropheids, phytosaurs, probably at least some proterochampsians), armoured armadillo-like taxa (i.e. aetosaurs) and gracile bipeds with relatively elongated necks (e.g. dinosaurs, shuvosaurids) [7–19]. Eventually, some Triassic archosauromorphs also explored completely new adaptive landscapes for the group, such as the presence of marine taxa (e.g. dinocephalosaurids, and some phytosaurs and poposauroids; [20–24]) and the first vertebrates to conquer the air via active flight (pterosaurs; [25,26]).

The origin of Archosauromorpha can be traced back to the middle–late Permian, with a few occurrences in western Europe (the early diverging *Protorosaurus speneri*), European Russia (the proterosuchid *Archosaurus rossicus* and the possible archosauriform *Eorasaurus olsoni*), continental Africa (the early diverging *Aenigmastropheus parringtoni*) and probably South America (an unnamed proterosuchid and early diverging taxa) [13,27–33]. The presence of Permian archosauriforms implies that the origin of the main non-archosauriform archosauromorph clades should also extend back well into the Permian, although Palaeozoic fossil evidence is still lacking for the vast majority of these groups (e.g. tanystropheids, rhynchosaurs, allokotosaurs, prolacertids; [6]). The only archosauromorph lineage with body fossils on both sides of the vertebrate fossil-defined Permo-Triassic boundary is Proterosuchidae [11,14,18,27,34]. The members of this clade are characterized by a low and elongated skull with a large and strongly downturned premaxilla, moderately long cervical series, relatively gracile limb bones and a plesiomorphic sprawling locomotion [11,35–37]. The unusual oversized and downturned premaxilla of proterosuchids became increasingly pronounced and distinctive through ontogeny, and mutual social and/or sexual selection may be an explanation for the function and evolutionary origin of this bizarre feature [38]. The endocranial (brain and inner ear moulds) anatomy of *Proterosuchus fergusi* suggests auditory capabilities specialized towards low-frequency sounds and probably semi-aquatic habits [39].

The general proterosuchid body plan described above has been used by several authors during the twentieth century to refer multiple species of gracile, medium-sized archosauromorphs from the Early–Middle Triassic to this group (e.g. [11,18,35,37,40,41]). As a result, Proterosuchidae became somewhat of a wastebasket group. The taxonomic content of the group was comprehensively tested in a quantitative analysis of Permo-Triassic archosauromorphs by Ezcurra [18]. This analysis found that Proterosuchidae as previously conceived was a polyphyletic group that included tanystropheid or tanystropheid-like taxa (*Exilisuchus tubercularis*), immediate sister taxa to Archosauriformes (*Tasmaniosaurus triassicus*), taxa more closely related to erythrosuchids and crownward archosauriforms (*Sarmatosuchus otschevi*, *Kalisuchus rewanensis*, *Chasmatosuchus* spp.), early diverging erythrosuchids (*Fugusuchus hejiapanensis*) and even crown archosaurs (*Chasmatosaurus ultimus*) [18,42,43]. As a consequence, the only taxa recovered as unambiguous proterosuchids by the analysis of Ezcurra [18] were three species of *Proterosuchus* from the Induan of South Africa (*Proterosuchus fergusi*, *Proterosuchus alexanderi* and *Proterosuchus goweri*), one species from the Induan of China (‘*Chasmatosaurus*’ *yuani*), and one species from the late Changhsingian of European Russia (*Archosaurus rossicus*). More recently, Ezcurra *et al.* [13] found that a partial braincase from the Late Permian–?earliest Triassic of Uruguay was very likely a proterosuchid. In addition, a few isolated partial bones from the Lower Triassic of India are also probably referable to Proterosuchidae [11,18,37,44–46].

The most abundant and informative proterosuchid specimens come from the Lower Triassic continental beds of the Karoo Basin of South Africa [11,36,47–49]. The fossil content of these rocks has been crucial for improving our understanding of the effects of the end-Permian mass extinction and its immediate aftermath in terrestrial ecosystems (e.g. [50–55]). This is as a result of the high abundance of taxonomically diverse and well-preserved tetrapod specimens in parallel with detailed stratigraphic control within the tetrapod-bearing units [53]. The proterosuchid genus *Proterosuchus* includes one of the first new vertebrate species (i.e. species not known in the Permian) to appear in the *Lystrosaurus declivis* Assemblage Zone of South Africa and, hence, after the mass extinction [50,51]. Unambiguous proterosuchid specimens are currently not recognized in post-Induan rocks, and thus the clade appears to be strongly temporally restricted [18]. As a consequence, proterosuchids have been considered as ‘disaster taxa’ because of their geographically broad diversification during a short time span in the aftermath of the mass extinction event [18,56]. In addition, proterosuchids are phylogenetically positioned at the base of Archosauriformes by definition and, as such, they are crucial to understanding the origin of that clade [18,57]. Thus, proterosuchids play a key role in understanding phylogeny, palaeoecology and macroevolution of the very beginning of the archosauriform radiation.

The importance of proterosuchids for more deeply comprehending the archosauromorph evolutionary radiation led us to try to expand current knowledge of this clade. Purported proterosuchid records from the Lower Triassic Panchet Formation of northeast India have attracted little attention since their initial reports in the nineteenth century [58]. Nevertheless, the few isolated *Proterosuchus*-like bones known from the Panchet beds are well preserved and are associated with abundant tetrapod fossils apparently numerically dominated by the dicynodont synapsid *Lystrosaurus* [44–46,58,59]. This combination of observations suggests that the Panchet Formation has high potential to provide new information about proterosuchid diversification in the earliest Triassic. We thus undertook a revision of historically collected proterosuchid specimens and their associated tetrapod assemblage in parallel with new fieldwork in the Panchet Formation in search for additional proterosuchid specimens. Here, we report the results of this project, which includes the detailed description of multiple proterosuchid bones, including the erection of a new proterosuchid species and an analysis of its phylogenetic relationships. Our results have important implications for understanding the taxonomic content of Proterosuchidae and the early macroevolution and biogeography of Archosauriformes.

### 1.1. The history of the Panchet proterosuchid specimens

The first tetrapod fossils from the Panchet Formation were discovered by William Blanford and Ambrose Tween of the Geological Survey of India (GSI) in 1860 [60,61]. These remains were found in the sandstones exposed adjacent to the Damodar River (=Damuda River in Huxley [58]) near Deoli village, and consisted of multiple isolated cranial and postcranial bones. The director of the GSI at that time, Thomas Oldham, sent the fossils to be studied by anatomist and evolutionary biologist Thomas Huxley, already a prominent figure of British science following the 1860 Oxford evolution debate [62]. Huxley [58] identified most of the amniote bones (cranial, girdle and limb elements) as belonging to dicynodont synapsids. These remains were the first reports of dicynodonts outside of South Africa and Huxley [58] concluded that they were numerically abundant in these Indian beds. In addition to these dicynodont specimens, Huxley [58] erected the new genus and species *Ankistrodon indicus* based on a small cranial fragment with two teeth (GSI 2259). *Ankistrodon indicus* was interpreted as a ‘thecodont reptile’, distinct from most reptiles known at the time because of a combination of recurved tooth crowns with distal denticles and an absence of mesial denticles. The holotype of ‘*Ankistrodon indicus*’ was not only the first proterosuchid specimen reported (although originally interpreted as a dinosaur), but to our knowledge was the first Early–Middle Triassic archosauromorph named in the scientific literature.

Huxley [58] also described several cervical, dorsal, sacral and caudal vertebrae that he considered as belonging to a single species of enigmatic affinities. He noted that the cervical vertebrae showed striking resemblances to those of *Protorosaurus speneri*—a species now considered one of the earliest-diverging archosauromorphs [18,30]—whereas the sacral vertebrae were identified as more similar to those of the dicynodont *Dicynodon orientalis*, now *Lystrosaurus murrayi* [*sensu* 63]. Huxley ([58], p. 19) considered that ‘it is a very difficult matter to decide whether they belong to *Dicynodon*, or to some other genus of Saurian’. Nevertheless, Huxley [58] concluded that these vertebrae were assignable to *Dicynodon* because 1) most of the bones of the Panchet collection belonged to *Dicynodon*, 2) they were as abundant as the dicynodont humeri and girdle bones, 3) their size matched that of the dicynodont remains, and 4) the sacral vertebrae were very similar to those of *Dicynodon*.

After the description of the first fossil tetrapod remains from the Panchet Formation [58], sporadic discoveries over the next two decades by members of the Geological Survey of India increased the fossil tetrapod collections from this unit. Again, almost all these specimens were isolated partial bones, but some of them provided novel information, mainly about dicynodonts, and were reported and described by Lydekker [64]. Among these bones, Lydekker [64] reported an element identified by him as a coracoid or ischium that he suggested could belong to *Ankistrodon* or to some other unknown reptile. This specimen (GSI 2173; Lydekker [64]: plate II, 8) is transversely thicker than would be expected for an archosauromorph coracoid or ischium, and is interpreted here as an indeterminate bone of a non-archosauromorph. Lydekker [64] considered the name *Ankistrodon* to be preoccupied by a previously named genus of reptile, and proposed the replacement genus *Epicampodon*, resulting in the new combination *Epicampodon indicus*. However, *Ankistrodon* was not actually preoccupied and *Epicampodon* was subsequently considered a *nomen vanum* [35]. A few years later, Lydekker [65] listed *Epicampodon indicus* within Dinosauria and the family Anchisauridae and later von Huene [66,67] considered it as a possible dinosaur and maybe assignable to *Thecodontosaurus*, which at that time was classified as a theropod dinosaur.

Von Huene [44] transferred *Ankistrodon indicus* to the proterosuchid genus *Chasmatosaurus*, which resulted in the new combination *Chasmatosaurus indicus*. In addition, von Huene [44] also reidentified the supposed dicynodont vertebrae described by Huxley [58] as belonging to a non-dinosaurian ‘thecodont’ and referred them to *Chasmatosaurus indicus*. This reidentification was mainly based on the presence of proportionally elongated vertebrae and laminae [44]. This taxonomic decision was subsequently followed by other authors (e.g. [68–70]). Late in his career, von Huene mentioned the presence of the genus *Chasmatosaurus* in the Panchet beds, but, surprisingly, in the same contribution he also listed ‘*Epicampodon*’ among the plateosauravid saurischian dinosaur genera, being supposedly closely related to the sauropodomorphs *Plateosauravus* and *Euskelosaurus* [71].

Charig & Reig [35] pointed out that the generic name *Ankistrodon* Huxley, 1865 [58] had temporal priority over *Chasmatosaurus* Haughton, 1924 [72] and, as a result, species of the latter genus should be transferred to *Ankistrodon* and not the other way round (contra von Huene [44]). Nevertheless, these authors considered the holotype of *Ankistrodon indicus* to be indeterminate and thus proposed that the genus and species should be considered *nomina dubia*. Similarly, they stated that the multiple vertebrae originally described by Huxley [58] cannot be referred to ‘*Ankistrodon indicus*’, but could be considered as cf. *Chasmatosaurus* sp. These conclusions were followed by subsequent authors (e.g. [18,37]). Welman [48] interpreted the holotype of the South African species *Proterosuchus fergusi* as diagnostic, and because *Proterosuchus* and *Proterosuchus fergusi* had priority over all other valid proterosuchid taxa he considered *Chasmatosaurus* and *Chasmatosaurus vanhoepeni* as subjective junior synonyms. Thus, in more recent years, several authors have mentioned the presence of the genus *Proterosuchus* in the Panchet Formation (e.g. [46,73–76]). *Proterosuchus fergusi* is still considered a valid species [49].

In December 1957, Pamela Robinson and staff from the GSI and the Indian Statistical Institute (ISI) (C. Tripathi, P. P. Satsangi, S. T. Rajurka, S. L. Jain and T. K. Roy Chowdhury) conducted fieldwork in the Panchet Formation [77]. They found isolated but well-preserved bones and a partial temnospondyl skull in the conglomeratic sandstones of the upper part of the unit, but no associated skeletons were recovered in these levels. Nevertheless, Robinson [77] reported for the first time the discovery of associated skulls and postcrania in the lower shales of the Panchet Formation. All these specimens were identified as a species of the dicynodont *Lystrosaurus* and no other tetrapods were recovered in this part of the unit [77]. Robinson [77] did not provide information about the identity of the new specimens collected in the upper sandstones. However, Hughes [69] mentioned (in the acknowledgements section) that Pamela Robinson had allowed him access to newly collected ‘*Chasmatosaurus indicus*' specimens. We believe that the latter proterosuchid specimens were likely collected during the fieldwork of 1957; proterosuchid specimens were located by us in the Pamela Robinson collection of the Natural History Museum (NHMUK) in London and they are probably the specimens mentioned by Robinson [77] and Hughes [69] (NHMUK PV R37576–37587; see below). In early 1964, Satsangi [45] completed further fieldwork in the Panchet Formation and found three isolated and incomplete proterosuchid bones; two dentaries (GSI 18123, 18124) and an ilium (GSI 18125). Satsangi [45] referred these three specimens to ‘*Chasmatosaurus*’ sp. and highlighted that as no articulated proterosuchid skeleton had been found to date in the Panchet Formation, these bones were of considerable interest. Recently, Pal [46] described an anterior cervical vertebra (PGRU/GL/M/VF-002), a middle caudal vertebra (PGRU/GL/M/VF-003) and the distal portion of a left humerus (PGRU/GL/M/VF-001) from the Panchet Formation, and referred these to *Proterosuchus*.

### 1.2. Palaeontological and geological context of the Panchet proterosuchid specimens

Outcrops of the Panchet Formation occur within the Damodar Basin, situated within the states of West Bengal and Jharkhand, northeast India. It is underlain by the late Palaeozoic Raniganj Formation, which is rich in coal and carbonaceous shale beds [78]. The Panchet Formation has been divided into lower and upper portions based on lithological differences (hereafter informally referred to as the lower and upper Panchet Formation, respectively; [79]). The lower Panchet Formation has red shales that form lenses of various sizes, which have been suggested to be of lacustrine origin [77]. These shales have yielded some articulated partial skeletons of the dicynodont *Lystrosaurus* [77,78]. By contrast, the upper Panchet Formation is dominated by yellow to brownish medium grained sandstones, with larger clasts especially abundant in some areas. These stratigraphic levels have been interpreted as deltaic [77]. Preserved within the sandstones are locally abundant isolated fossil vertebrate bones [58,77], which represent the bulk of the vertebrate fossil record of the Panchet Formation. Bones show evidence of transportation before burial (e.g. abrasion, breakage) and articulated specimens are extremely rare [77,78].

The fossil vertebrate assemblage of the Panchet Formation is relatively diverse compared to other Early Triassic assemblages. Fishes are represented by scales, teeth and tooth plates of an unknown number of species of actinopterygians, dipnoans and chondrichthyans [80]. Numerous temnospondyls are known, including the trematosaurids *Indolyrocephalus* and *Gonioglyptus*, the rhytidosteid *Indobrachyops*, the lydekkerinid *Lydekkerina,* the lapillopsid *Manubrantlia*, the plagiosaurid *Capulomala,* the brachyopid *Pachygonia*, a probable tupilakosaurid, and an indeterminate benthosuchid [74–76,81,82]. Synapsids include the dicynodonts *Lystrosaurus murrayi*, *Lystrosaurus* cf. *L. curvatus* and *Lystrosaurus* cf. *L. declivis* [59,78,83], as well as the cynodonts *Thrinaxodon bengalensis* and *Panchetocynodon damodarensis* [73,84]. Finally, sauropsids include an indeterminate non-archosauromorph neodiapsid [85], and the proterosuchid specimens already mentioned [11,45,46,58].

Regarding more controversial specimens, the species *Teratosaurus*(?) *bengalensis* was erected based on an isolated tooth and considered to differ from ‘*Ankistrodon indicus*’ because of the presence of mesial denticles [86]. However, the morphology of *Teratosaurus*(?) *bengalensis* is consistent with that of proterosuchids and the teeth of ‘*Ankistrodon indicus*’ lack their apical ends (see description of ‘Dentition’). It is likely that *Teratosaurus*(?) *bengalensis* is not a valid species, but its revision goes beyond the scope of this work. An isolated vertebral centrum was interpreted as belonging to a rhynchocephalian—a group that at that time included rhynchosaurs [86]. Subsequently, this specimen was reinterpreted as a nothosaur [87], but Robinson [88] cast doubts on this identification because the supposed nothosaur similarities were a consequence of damage. Finally, a putative procolophonid remains from the Panchet Formation [89] was based on a fragmentary and misidentified specimen (T. RoyChowdhury, personal communication 2007).

Regarding the age of the Panchet Formation, Owen [90] correlated this unit with the Karoo Basin in South Africa and considered its age to be between the Upper Carboniferous and, more likely, the Triassic. Studies during subsequent decades agreed with the probable Triassic age of the Panchet Formation based mainly on its fossil floral and faunal content [64,91]. Koken [92] placed the deposition time of the Panchet Formation in the lower half of the Late Triassic, von Huene [67] proposed an approximately Middle Triassic age based on the faunal content of the unit, Cotter [93] considered a Lower Triassic age, and Dasgupta [86] a Permian–Lower Triassic age based on a supposed mixture of Palaozoic and Triassic faunal content. The report of Robinson [77] of the first articulated specimens of the dicynodont *Lystrosaurus* in the lower levels of the Panchet Formation allowed this unit to be correlated with the earliest Triassic (Induan) of the Karoo Basin (now *Lystrosaurus declivis* Assemblage Zone) of South Africa, and the probably coeval *Lystrosaurus*-bearing levels of the Jiucaiyuan Formation of China. Anderson & Cruickshank ([94]: chart 2.1) assigned the Panchet Formation an early Induan age based on a comprehensive analysis of the global vertebrate content of Triassic continental assemblages. An Early Triassic, probably Induan, age has been followed by more recent authors (e.g. [59,74,75,84,95,96]).

Gupta & Das [78] described three dicynodont skulls from the shales of the lower Panchet Formation and identified them as *Lystrosaurus* cf. *L. curvatus* and *Lystrosaurus* cf. *L. declivis*. As a result, these authors proposed the co-occurrence of these two dicynodont taxa with *Lystrosaurus murrayi* in the lower Panchet Formation but concluded that only *Lystrosaurus murrayi* occurs in the upper portion of the unit. Gupta & Das [78] discussed the biostratigraphic implications of different stratigraphic ranges of dicynodont taxa in the Panchet Formation based on similar ranges also present in the uppermost Permian–lowermost Triassic *Daptocephalus* and *Lystrosaurus declivis* Assemblage Zones (AZs) of the Karoo Basin of South Africa. *Lystrosaurus maccaigi* and *Lystrosaurus curvatus* occur on both sides of the Permo-Triassic boundary in the Karoo [55]. The other two *Lystrosaurus* species recorded in the Karoo, *Lystrosaurus murrayi* and *Lystrosaurus declivis*, co-occur with the latter two species in the Palingkloof Member of the Balfour Formation that corresponds to first metres after the Permo-Triassic boundary, but they extend higher in the stratigraphic sequence throughout the *Lystrosaurus declivis* AZ [53,55,97]. Thus, the co-occurrence of *Lystrosaurus* cf. *L. curvatus*, *Lystrosaurus* cf. *L. declivis* and *Lystrosaurus murrayi* in the lower Panchet Formation allowed Gupta & Das [78] to correlate this part of the unit with the lowermost Induan Palingkloof Member of the Balfour Formation, which is also the unit that preserves the oldest occurrences of the proterosuchid *Proterosuchus* spp. in South Africa [97]. The presence of only *Lystrosaurus murrayi* in the upper Panchet Formation may indicate a correlation with the Katberg Formation [78], the unit that immediately overlies the Balfour Formation in the Karoo Basin and its first metres yielded the youngest known specimens of *Proterosuchus* spp. in South Africa [55,97]. Thus, current evidence indicates that the lower Panchet Formation yields a vertebrate assemblage that lived immediately or shortly after the Permo-Triassic boundary and that the upper Panchet Formation is somewhat younger. The stratigraphic range of *Lystrosaurus murrayi* probably extends around the Induan−Olenekian boundary in South Africa [97] and, thus, the upper Panchet Formation would have a similar temporal range. However, we consider that further geological and palaeontological research throughout the Panchet Formation is necessary to test and refine this current chronostratigraphic scheme.

## 2. Material and methods

### 2.1. Panchet proterosuchid specimens, fieldwork and fossil preparation

To revisit the anatomy, taxonomy and phylogeny of the proterosuchid specimens from the Panchet Formation, we studied in person the historical specimens published by Huxley [58] (the holotype of ‘*Ankistrodon indicus*’, the vertebrae originally referred to ‘*Dicynodon orientalis*’, and other bones of the original Blanford and Tween collection) and those more recently described by Satsangi [45]. Recently, Pal [46] reported that the proterosuchid vertebrae described by Huxley [58] were lost, but these specimens were accessed and studied first hand by one of us (M.D.E.) in February 2015 at the GSI in Kolkata. We agree with previous proposals that the supposed dicynodont vertebrae described by Huxley [58] belong to an archosauromorph [35,44]. In addition, tooth-bearing bones previously identified as belonging to temnospondyls by Huxley [58] are reinterpreted here as archosauromorph specimens. We also located and studied previously unpublished archosauriform vertebrae from the Panchet Formation in the Pamela Robinson collection at NHMUK. We added to these previously collected specimens several new Panchet archosauriform bones found during fieldwork by one of us (K.S.) during the late 1990s and more recently during fieldwork conducted by several of the present authors (M.D.E., S.B., D.P.S., R.B.S. and R.J.B.) in early 2015 (see below). All these specimens came from the yellow-brownish conglomeratic sandstones of the upper Panchet Formation and from at least two different localities, Dumdumi and Banspatali. As a result of these fieldtrips and restudied historical specimens, we identified approximately 90 archosauriform bones belonging to the skull, all regions of the vertebral column, pelvic girdle and limbs.

Our fieldwork was conducted between 17 and 20 January 2015 and focused on the sandstone beds of the upper Panchet Formation at localities south of the Damodar River. All the archosauriform specimens were collected in the conglomeratic sandstones of the Deoli locality (23°39′03.1″ N, 86°52′56.0″ E), which were very rich in vertebrate fossil remains. In addition, we also collected fish, temnospondyl and synapsid fossil remains at this locality. These same sandstones and, in a very few cases, the red mudstones in other localities (e.g. north of Dumdumi village and the type locality of *Panchetocynodon damodarensis*: 23°38′1.60″ N, 86°53′39.60″ E; northeast of Dumdumi village: 23°37′56.80″ N, 86°53′50.90″ E; south of Dumdumi village: 23°37′26.80″ N, 86°53′39.60″ E; Tripathi locality 14, near Iswarda: 23°37′04.4″ N, 86°56′26.7″ E) yielded a lower richness of fossil vertebrate remains during our fieldwork, all belonging to fishes, dicynodonts, and indeterminate tetrapods. Some other outcrops of the Panchet Formation did not yield fossil specimens (e.g. southeast of Dumdumi village: 23°37′42.30″ N, 86°53′44.50″ E; southwest of Dumdumi village: 23°37′25.60″ N, 86°53′21.60″ E) or former outcrops are no longer exposed (e.g. Tripathi locality 13, near Iswarda: 23°37′15.46″ N, 86°56′.70″ E). All the specimens collected during our fieldwork were found isolated, without articulation between bones. The specimens were collected in small blocks of matrix containing the bones following excavation using hammers and chisels. If necessary, broken bones were adhered with cyanoacrylate adhesives. In the ISI, the matrix was softened by soaking it in water and the specimens were then prepared mechanically with tungsten carbide needles. Preparation with 5% acetic acid was not successful because it did not remove the matrix.

### 2.2. Anatomical terminology

We follow here the nomenclature for vertebral laminae of Wilson [98] and the terminology for limb orientation in sprawling animals of Rewcastle [99].

### 2.3. Phylogenetic analyses

In order to reconstruct the phylogenetic relationships of *Samsarasuchus pamelae* gen. et sp. nov. (see Systematic Palaeontology), we used the most recent iteration of the phylogenetic data matrix of the CoArTreeP (the Complete Archosauromorph Tree Project; see Ezcurra [18] for the first iteration of this project), which was published by Ezcurra & Sues [100]. This is the most extensive phylogenetic dataset currently available for Permian and Triassic archosauromorphs and was originally focused on non-eucrocopodan archosauriforms [18]. The Ezcurra & Sues [100] version of the matrix is a result of the fusion of different versions of the dataset that have diverged independently because of expansions in the character and taxonomic sampling in recent years (e.g. [6,13,14,26,101–108]). We scored our new taxon, *Samsarasuchus pamelae* gen. et sp. nov., as two different terminals: ‘*Samsarasuchus pamelae*’, which includes the holotype, paratype and referred specimens (i.e. all presacral vertebrae to the exclusion of the axis and posterior dorsal vertebrae), and ‘*Samsarasuchus pamelae* expanded’, which includes the holotype, paratype, referred specimens, and cf. proterosuchid and proterosuchid specimens from the Panchet Formation. This approach allowed us to assess the phylogenetic implications of including or not including cf. proterosuchid and proterosuchid specimens that currently cannot be referred unambiguously to *Samsarasuchus pamelae* gen. et sp. nov. We added to the dataset 11 terminals sampling the following putative Early Triassic non-eucrocopodan archosauriforms (table 1): 1) ‘Arcadia proterosuchian vertebrae’, which are vertebrae from the Lower Triassic Arcadia Formation of Australia previously referred to *Kalisuchus rewanensis* (hereafter ‘Arcadia proterosuchian’) [18,111]; 2) ‘*Blomosuchus georgii* holotype’ from the Induan Vokhmian Gorizont Russia (based only on the holotype specimen; see Ezcurra [18]); 3) ‘*Vonhuenia friedrichi* combined’ that includes the already scored terminal ‘*Vonhuenia friedrichi* holotype’ and the referred specimens to the species from the Induan Vokhmian Gorizont (*sensu* Sennikov [40]); 4) ‘*Tsylmosuchus* spp.’ that includes the holotypes of the three species of *Tsylmosuchus* from the lower Olenekian Rybinskian and Ustmylian gorizonts of Russia (*sensu* Sennikov [112]); 5) ‘*Chasmatosuchus rossicus* holotype’ that includes only the holotype of the species from the lower Olenekian Rybinskian Gorizont (the complete hypodigm of the species, ‘*Chasmatosuchus rossicus* complete’, was already scored in the matrix); 6) *Gamosaurus lozovskii*, which includes the holotype and a referred cervical vertebra, with overlapping morphology with the latter specimen, from the upper Olenekian Yarengian Gorizont of Russia [40,113]; 7) ‘*Jaikosuchus* Ochev hypodigm’ that includes the holotype of *Jaikosuchus magnus* (included as part of ‘*Chasmatosuchus magnus* combined’ in previous versions of this matrix) and a referred fibula found in the same locality of the upper Olenekian Yarengian Gorizont (*sensu* Ochev [113]); 8–10) three archosauriform vertebrae recently described from the Induan–Olenekian Sanga do Cabral Formation of Brazil (UNIPAMPA 271, 684, 750), which were assigned to cf. *Proterosuchus* and cf. *Chasmatosuchus* [110]; and 11) ‘Long Reef proterosuchian’, which are two vertebrae from the ?lower Olenekian Bulgo Sandstone of Australia [114]. ‘*Blomosuchus georgii* holotype’, *Gamosaurus lozovskii*, and the ‘Long Reef proterosuchian’ were scored in the dataset of Ezcurra [18], but were not included in later versions of the matrix and were reintroduced here. Finally, we included a terminal (*Kalisuchus rewanensis* combined) that combines the scorings of the holotype of *Kalisuchus rewanensis* and those of the vertebrae of the Arcadia Formation and another terminal (Jaikosuchus + Vytshegdosuchus) that combines the scorings of *Vytshegdosuchus zheshartensis* and ‘*Jaikosuchus magnus* holotype’. These two terminals are included to explore the phylogenetic implications of considering each of them as a single species (i.e. *Vytshegdosuchus zheshartensis* as a junior synonym of *Jaikosuchus magnus* and the Arcadia vertebrae as referrable to *Kalisuchus rewanensis*). All these additions to the taxonomic sampling of the phylogenetic dataset allow a comprehensive test of the taxonomic content of Proterosuchidae and the spatio-temporal distribution of the clade.

**Table 1.** Specimens used here for the scoring in the data matrix of non-erythrosuchid, non-eucrocopod archosauriform terminals in our phylogenetic analyses (or their combination with other specimens to test hypotheses of more inclusive hypodigms). All specimens studied at first hand by at least one of the authors with the exception of those indicated with an asterisk.

terminal	specimen(s)
*Antarctanax shackletoni*	UWBM 95531* (scorings mainly based on Peecook *et al.* [109])
Arcadia proterosuchian vertebrae	QMF9529–9537, 9548, 10125, 17990, 60371
*Archosaurus rossicus* holotype	PIN 1100/55 (holotype)
*Archosaurus rossicus* complete	PIN 1100/55 (holotype *Archosaurus rossicus*), 1100/66, 1100/66a, 1100/66b, 1100/78, 1100/84, 1100/85, 1100/85a, 1100/85b
‘*Blomosuchus georgii*’	PIN 1025/348 (holotype)
‘*Chasmatosaurus*’ *yuani*	IVPP V36315 (holotype), V2719, V4067
*Chasmatosuchus rossicus* holotype	PIN 2252/381 (holotype)
*Chasmatosuchus rossicus* complete	PIN 2252/381 (holotype *Chasmatosuchus rossicus*), 160/9, 160/10, 2243/167, 2252/12–15, 2252/383, 2252/384, 2252/386, 2354/26, 2354/110, 2355/25, 3200/212, 3200/217, 3200/472
‘*Chasmatosuchus*’ *vjushkovi*	PIN 2394/4 (holotype)
FC-DPV 2641	FC-DPV 2641
*Gamosaurus lozovskii*	PIN 3361/13 (holotype), 3361/14
*Jaikosuchus magnus*	PIN 951/65 (holotype)
*Jaikosuchus* Ochev hypodigm	PIN 951/65 (holotype), 951/41
*Kalisuchus rewanensis* holotype	QMF8998 (holotype)
*Kalisuchus rewanensis* combined	QMF8998 (holotype *Kalisuchus rewanensis*), QMF9521, QMF9529–9537, 9548, 10125, 17990, 60371
Long Reef proterosuchian	SAM P41754
NMQR 3570	NMQR 3570
*Proterosuchus alexanderi*	NMQR 1484 (holotype)
*Proterosuchus fergusi*	See hypodigm in Ezcurra & Butler [49]
*Proterosuchus goweri*	NMQR 880 (holotype)
*Samsarasuchus pamelae*	holotype, paratype, and referred specimens (see Systematic Palaeontology)
*Samsarasuchus pamelae* expanded	holotype, paratype, referred specimens, and Panchet cf. proterosuchid and proterosuchid specimens (see Systematic Palaeontology)
*Sarmatosuchus otschevi*	PIN 2865/68 (holotype)
*Tsylmosuchus* spp.	PIN 1043/42 (holotype *Tsylmosuchus donensis*), PIN 2424/6 (holotype *Tsylmosuchus samariensis*), PIN 4332/1 (holotype *Tsylmosuchus jakovlevi*)
UNIPAMPA 271	UNIPAMPA 271* (scorings mainly based on De-Oliveira *et al.* [110])
UNIPAMPA 684	UNIPAMPA 684* (scorings mainly based on De-Oliveira *et al.* [110])
UNIPAMPA 750	UNIPAMPA 750* (scorings mainly based on De-Oliveira *et al.* [110])
*Jaikosuchus* + *Vytshegdosuchus*	PIN 3361/134 (holotype of *Vytshegdosuchus zheshartensis*), PIN 4383/1, PIN 951/65 (holotype of *Jaikosuchus magnus*)
*Vonhuenia friedrichi* holotype	PIN 1025/11 (holotype)
*Vonhuenia friedrichi* combined	PIN 1025/11 (holotype *Vonhuenia friedrichi*), 1025/14, 1025/15, 1025/355, 1025/348 (holotype ‘*Blomosuchus georgii*’), 1025/404–406, 1025/419, 1025/420, 1025/422, 1025/425

We modified 14 characters and added 19 characters (see electronic supplementary material), and the following characters were considered additive: 1, 2, 7, 10, 17, 19–21, 28, 29, 34, 36, 40, 42, 46, 50, 54, 66, 71, 74–76, 100, 122, 127, 146, 153, 156, 157, 171, 176, 177, 187, 202, 221, 227, 263, 266, 278, 279, 283, 311, 324, 327, 331, 337, 342, 345, 351, 352, 354, 361, 365, 368, 370, 377, 379, 386, 387, 398, 410, 414, 416, 424, 425, 430, 435, 446, 448, 454, 455, 458, 460, 463, 464, 470, 472, 478, 482, 483, 485, 489, 490, 502, 504, 510, 516, 520, 521, 529, 537, 546, 552, 556, 557, 567, 569, 571, 574, 581, 582, 588, 636, 648, 652, 662, 701, 731, 735, 737, 738, 743, 749, 766, 784, 803, 810, 816, 850, 851, 872, 875, 885, 888, 894, 898 and 900 because they represent nested sets of homologies. Characters 9 and 119 were deactivated (following Ezcurra *et al.* [101] and Butler *et al.* [115]). We also excluded/deactivated the following 40 terminals before the tree searches of the first analysis (Analysis 1) because they were scored only for the purpose of morphological disparity analyses, are not diagnostic at a species level, or are combinations between other operational taxonomic units: ISIR 1132, *Protanystropheus antiquus*, *Trachelosaurus fischeri*, *Tanystropheus haasi*, UFRGS-PV-492-T, *Malerisaurus* all NA, *Arctosaurus osborni*, CRILAR-Pv 461, CRILAR-Pv 462, CRILAR-Pv 497, Chanares rhynchosaur, PVSJ 2728, *Eorasaurus olsoni*, *Archosaurus rossicus* holotype, *Blomosuchus georgii* holotype, *Samsarasuchus pamelae* expanded, *Kalisuchus rewanensis* combined, UFSM 11444, UFSM 11394, *Vonhuenia friedrichi* holotype, *Chasmatosuchus rossicus* holotype, *Jaikosuchus* Ochev hypodigm, *Chasmatosuchus vjushkovi*, *Koilamasuchus gonzalezdiazi*, CRILAR-Pv 499, *Shansisuchus kuyeheensis*, *Uralosaurus* combined, *Osmolskina czatkoviensis*, *Osmolskina* complete, *Triopticus primus*, Otter Sandstone archosaur, *Dagasuchus santacruzensis*, *Ctenosauriscus koeneni*, *Hypselorhachis mirabilis*, Waldhaus poposauroid, *Jaikosuchus + Vytshegdosuchus*, *Bystrowisuchus flerovi*, *Bromsgroveia walkeri*, *Lutungutali sitwensis* and *Nyasasaurus parringtoni*. The final modified dataset is composed of 205 active terminals (Analysis 1) and 906 active characters (see electronic supplementary material).

Six additional analyses were conducted with the following changes in the taxonomic sampling: Analysis 2, replacement of *Jaikosuchus magnus* with ‘*Jaikosuchus* Ochev hypodigm’ to explore the implications of the addition to the fibula originally referred to the species [113]; Analysis 3, replacement of *Jaikosuchus magnus* and *Vytshegdosuchus zheshartensis* with the ‘*Jaikosuchus + Vytshegdosuchus*' terminal to explore the implications of the hypothesis that they belong to the same species; Analysis 4, replacement of the ‘Arcadia proterosuchian vertebrae’ and *‘Kalisuchus rewanensis* holotype’ with ‘*Kalisuchus rewanensis* combined’ to explore the implications if they belong to the same species (following Thulborn [111]); Analysis 5, inclusion of the highly fragmentary ‘*Chasmatosuchus*’ *vjushkovi*; Analysis 6, inclusion of the less inclusive hypodigms of the putative proterosuchids, i.e. ‘*Vonhuenia friedrichi* holotype’, ‘*Archosaurus rossicus* holotype’ and ‘*Chasmatosuchus rossicus* holotype’, instead of their more inclusive hypodigms; and Analysis 7, inclusion of all the proterosuchid specimens from the Panchet Formation as the single terminal ‘*Samsarasuchus* expanded’.

The matrix of discrete morphological characters (available as electronic supplementary material, Files) was analysed under maximum parsimony using TNT v.1.5 [116,117]. The analyses were conducted under implied weighting with concavity constants (k) of *k* = 19–24. This decision of weighting against homoplasy follows the results and recommendation of Ezcurra [118], who recovered the result that implied weighting outperforms the results under equal weighting, and *k* values between 19 and 24 showed the best performances through the genealogy of this phylogenetic data matrix. The program was set to retain up to 250 000 trees in memory during the search of the most parsimonious trees (MPTs). The search strategy initially used a combination of the tree-search algorithms sectorial searches, drifting, ratchet and tree fusing, until 100 hits of the same minimum tree length were achieved. The shortest trees obtained were then subjected to a final round of TBR branch swapping. Zero length branches in any of the recovered MPTs were collapsed. Homoplasy indices were calculated with a script that does not take into account *a priori* deactivated terminals (STATSb.run; see electronic supplementary material, of Spiekman *et al.* [119]). Branch support was quantified using a bootstrap resampling analysis, with 1000 pseudo-replicates and reporting both absolute and GC (‘Group present/Contradicted’; i.e. difference between the frequencies of recovery in pseudo-replicates of the clade in question and the most frequently recovered contradictory clade) frequencies [120].

### 2.4. Leaf stability analysis

Some species traditionally identified as proterosuchids have had relatively unstable phylogenetic positions in recent decades (e.g. *Chasmatosuchus rossicus*, *Jaikosuchus magnus*, *Vonhuenia friedrichi*, *Fugusuchus hejiapanensis*; [11,18,43]). To quantify this instability and test if it is caused by higher amounts of missing data in this part of the tree than the average of the dataset or because it is driven by temporal or phylogenetic patterns, we calculated the leaf stability [121,122] of each operational taxonomic unit of Analysis 5, which has the most extensive taxon sampling. The analysis was conducted under implied weighting with *k* = 20 and we saved in memory all the trees generated from 1000 bootstrap pseudo-replicates and calculated the leaf stability index (LSI) of Thorley & Wilkinson [121], which is the average difference between the first and second most frequent resolution of triplets of terminals. We plotted the LSI and the amount of missing data of each terminal against geological time. We also plotted and conducted phylogenetic generalized least-square (pGLS) regressions of LSI versus the amount of missing data and LSI versus the mean of the chronostratigraphic uncertainty of each terminal, respectively. These plots and regressions allow investigation of patterns of leaf instability with respect to completeness and geological time. Finally, we plotted violin plots and conducted phylogenetic ANOVAs between the LSIs of four groups: 1) the unambiguous proterosuchid terminals recovered in Analysis 5; 2) non-proterosuchid Permian−Early Triassic archosauromorphs; 3) all Permian−Early Triassic archosauromorphs; and 4) Middle Triassic archosauromorphs. The calculation of LSI was conducted in TNT 1.5 [117] using a script newly written for this purpose (available in electronic supplementary material). The pGLS regressions were conducted with the procD.pgls function (using type II sum of squares and 999 iterations) of the R package geomorph [123] and the phylogenetic ANOVAs with the phylANOVA function (conducting posthoc tests, 999 simulations and the Bonferroni correction) of the R package phytools [124]. The pGLS regressions and phylogenetic ANOVAs used one, randomly selected, MPT of Analysis 5 that was time-calibrated with the timePaleoPhy function of the R package paleotree [125], using the ‘equal_paleotree_legacy’ method with a minimum branch length of 0.1. We did not use other MPTs and alternative minimum branch length or stochastic calibration methods (e.g. ‘cal3’) because very similar phylogenetic topologies, such as those recovered in our phylogenetic analyses, have a low influence on the results of this kind of phylogenetically informed statistical analyses. All time-calibrations, analyses and plots were conducted in R 4.2.1 [126].

### 2.5. Institutional abbreviations

**AM**, Albany Museum, Makhanda (previously Grahamstown), South Africa; **BP**, Evolutionary Studies Institute (formerly Bernard Price Institute for Palaeontological Research), University of the Witwatersrand, Johannesburg, South Africa; **CGS**, Council for Geoscience (GHG after the institutional abbreviations refers to the collector Gideon H. Groenewald), Pretoria, South Africa; **CRILAR-Pv**, Centro Regional de Investigaciones y Transferencia Tecnológica de La Rioja, Paleontología de Vertebrados, Anillaco, Argentina; **FC-DPV**, Vertebrados Fósiles, Facultad de Ciencias, Montevideo, Uruguay; **GSI**, Geological Survey of India, Kolkata, India; **ISI**, Indian Statistical Institute, Kolkata, India; **IVPP**, Institute of Vertebrate Paleontology and Paleoanthropology, Beijing, China; **MCNAM PV**, Museo de Ciencias Naturales y Antropológicas de Mendoza (J. C. Moyano), Paleovertebrados, Mendoza, Argentina; **NHMUK PV**, Natural History Museum, London, UK; **NMQR**, National Museum, Bloemfontein, South Africa; **NMT**, National Museum of Tanzania, Dar es Salaam, Tanzania; **PGRU/GL/M/VF**, Post-Graduation College, Museum of the Department of Geology, University of Ranchi, Ranchi, India; **PIN**, Borissiak Paleontological Institute of the Russian Academy of Sciences, Moscow, Russia; **PVL**, Paleontología de Vertebrados, Instituto ‘Miguel Lillo’, San Miguel de Tucumán, Argentina; **PVSJ**, División de Paleontología de Vertebrados del Museo de Ciencias Naturales y Universidad Nacional de San Juan, San Juan, Argentina; **QMF**, Queensland Museum, Brisbane, Queensland, Australia; **RC**, Rubidge Collection, Wellwood, Graaff-Reinet, South Africa; **SAM**, South Australian Museum, Adelaide, Australia; **SAM-PK,** Iziko South African Museum, Cape Town, South Africa; **SMNS**, Staatliches Museum für Naturkunde Stuttgart, Stuttgart, Germany; **SNSB-BSPG**, Staatliche Naturwissenschaftliche Sammlungen Bayerns-Bayerische Staatssammlung für Paläontologie und Geologie, Munich, Germany; **UFRGS**, Universidade Federal do Rio Grande do Sul, Porto Alegre, RS, Brazil; **UFSM**, Universidade Federal de Santa Maria, Santa Maria, Brazil; **UNIPAMPA**, Universidade Federal do Pampa, São Gabriel, Brazil; **UTGD**, School of Earth Sciences, University of Tasmania, Hobart, Australia; **UWBM**, Burke Museum of Natural History and Culture, Seattle, Washington, USA.

## 3. Systematic Palaeontology

Diapsida Osborn, 1903 [127] [Gauthier & de Queiroz (2020)] [128]

Archosauromorpha von Huene, 1946 [129] [Gauthier (2020)] [130]

Archosauriformes Gauthier, Kluge & Rowe, 1988 [131] [Gauthier (2020)] [132]

Proterosuchidae von Huene, 1908 [133] *sensu* Ezcurra, Butler & Gower, 2013 [11]

Chasmatosuchinae nov.

### 3.1. LSID

urn:lsid:zoobank.org:act:D7B131F6–15E1-4DF8-8C57-BADF69AD404E.

### 3.2. Phylocode registration number

Chasmatosuchinae is identified in the international clade names repository as registration number 1010.

### 3.3. Phylogenetic definition

The most inclusive clade containing *Chasmatosuchus rossicus* von Huene, 1940 [134], but not *Proterosuchus fergusi* Broom, 1903 [135], ‘*Chasmatosaurus*’ *yuani* Young, 1936 [136], *Proterosuchus alexanderi* (Hoffman, 1965) [47], *Proterosuchus goweri* Ezcurra & Butler, 2015 [49], *Erythrosuchus africanus* Broom, 1905 [137], or *Alligator mississippiensis* Daudin, 1802 [138]. This is a maximum clade definition.

### 3.4. Reference phylogeny

Phylogenetic hypothesis recovered in this paper.

### 3.5. Composition

The composition is based on the reference phylogeny. Chasmatosuchinae includes *Chasmatosuchus rossicus*, *Jaikosuchus magnus*, *Samsarasuchus pamelae*, *Archosaurus rossicus*, *Gamosaurus lozovskii*, *Tsylmosuchus* spp., *Vonhuenia friedrichi* and indeterminate specimens from the Arcadia Formation and Bulgo Sandstone of the Sydney Basin of Australia and the Sanga do Cabral Formation of Brazil.

### 3.6. Diagnosis

Chasmatosuchines differ from other proterosuchids in the presence of anterior–middle and sometimes posterior postaxial cervical vertebrae with distally restricted transverse expansion of the neural spines (not mammillary process); third to eighth or ninth presacral vertebrae with diagonal, anterodorsally-to-posteroventrally oriented ridge that reaches the base of the prezygapophysis and is not connected to the diapophysis on the lateral surface of the neural arch; fourth to eight presacral vertebrae with posterior expansion of the dorsal portion of the neural spine, resulting in a posterodorsally tilted posterior margin set at an angle higher than 15° with respect to the anterior margin of the neural spine in lateral view; ninth presacral centrum with a ventral keel and anterior caudal vertebrae with surface lateral to the base of the neural spine with a very deep fossa, well-defined laterally and that transversely constricts the anterior half of the neural spine.

*Samsarasuchus pamelae* gen. et sp. nov.

Figures 1*e*, 2–5*a,b,e,f*, figures 6 and 7*a–c*, 8, 10–16 and tables 2–6.

**Figure 1.**
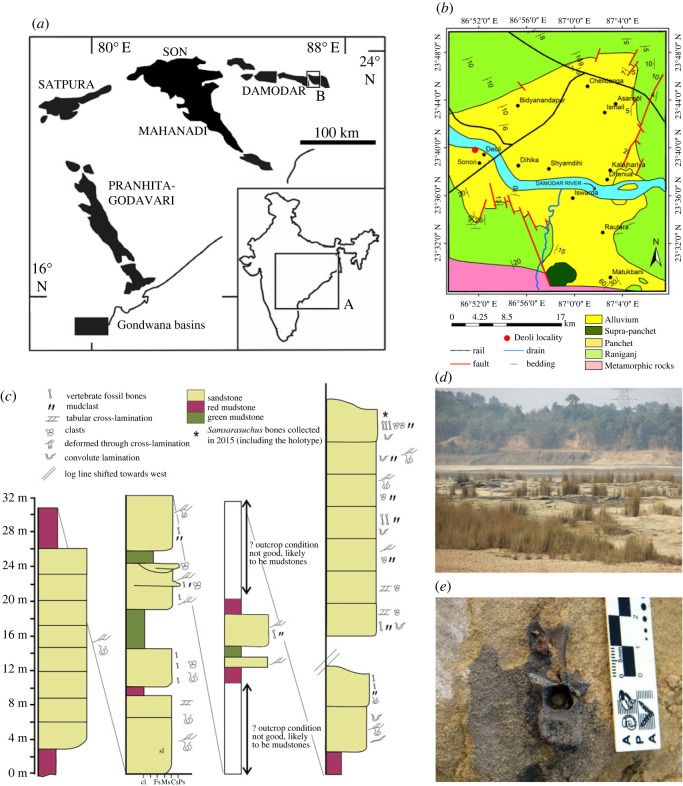
Geographical and stratigraphic occurrence of specimens of *Samsarasuchus pamelae* gen. et sp. nov. and Panchet cf. proterosuchid and proterosuchid specimens. (*a*) Gondwana basins of peninsular India showing the studied area of the Damodar Basin, which has yielded specimens of *Samsarasuchus pamelae*; (*b*) geological map showing the location of the Deoli locality that yielded *Samsarasuchus pamelae* bones discovered by the 2015 fieldtrip; (*c*) composite stratigraphic column of the Panchet Formation, including the occurrence of the *Samsarasuchus pamelae* bones discovered by the 2015 fieldtrip; (*d*) general overview of the sandstones of the Deoli locality on the shore of the Damodar River (January 2015); and (*e*) close up of the holotype of *Samsarasuchus pamelae* (ISIR 1091) as found in the field.

**Figure 2.**
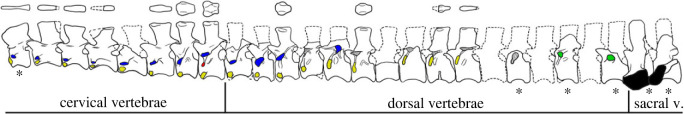
Drawing of the composite reconstruction of the presacral and sacral vertebral series of *Samsarasuchus pamelae* gen. et sp. nov. and Panchet cf. proterosuchid specimens (axis and 12th to 16th dorsal and sacral vertebrae; indicated with an asterisk). Neural spines in dorsal view (top row) and vertebrae in left lateral view (bottom row). Diapophyses indicated in blue, parapophyses in yellow, synapophyses in green, third articular rib facet in red, and iliac facets on sacral vertebrae in black. v, vertebrae.

**Figure 3.**
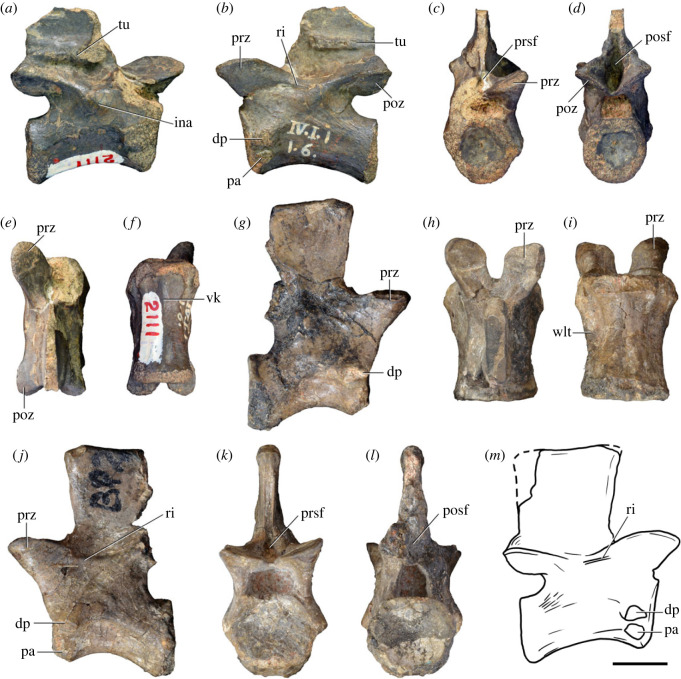
Anteriormost postaxial cervical vertebrae of *Samsarasuchus pamelae* gen. et sp. nov. (*a–f*) Third cervical vertebra (GSI 2111), (*g–l*) third or fourth cervical vertebra (NHMUK PV R37578), and (*m*) drawing of reconstructed third cervical vertebra (based on GSI 2111 and NHMUK PV R37578) in (*a, g, m*) right lateral, (*b, j*) left lateral, (*c, k*) anterior, (*d, l*) posterior, (*e, h*) dorsal, and (*f, i*) ventral views. dp, diapophysis; ina, inflated area; pa, parapophysis; posf, postspinal fossa; poz, postzygapophysis; prsf, prespinal fossa; prz, prezygapophysis; ri, ridge; tu, tuberosity; vk, ventral keel; wlt, wing-like tuberosity. Scale bar equals 1 cm.

**Figure 4.**
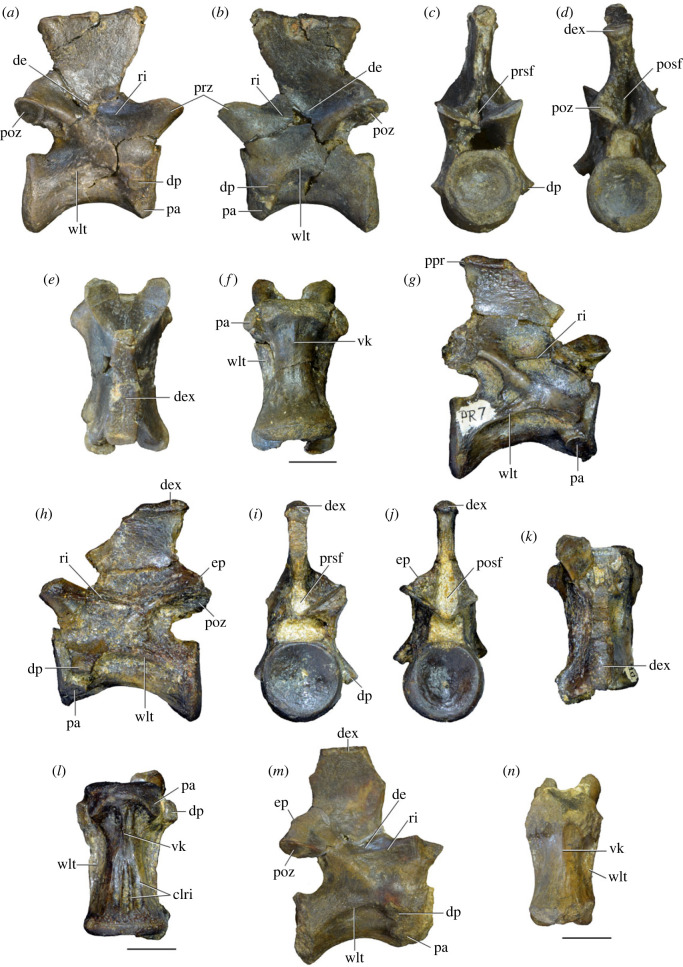
Fourth and fifth cervical vertebrae of *Samsarasuchus pamelae* gen. et sp. nov. (*a–f*) Fourth cervical vertebra (ISIR 1080, paratype), (*g–l*) fifth cervical vertebra (ISIR 1081) and (*m, n*) fifth cervical vertebra (ISIR 1084) in (*a, g, m*) right lateral, (*b, h*) left lateral, (*c, i*) anterior, (*d, j*) posterior, (*e, k*) dorsal and (*f, l, n*) ventral views. clri, collateral ridges; de, depression; dex, distal expansion; dp, diapophysis; ep, epipophysis; pa, parapophysis; posf, postspinal fossa; poz, postzygapophysis; ppr, posterior projection; prsf, prespinal fossa; prz, prezygapophysis; ri, ridge; vk, ventral keel; wlt, wing-like tuberosity. Scale bars equal 1 cm.

**Figure 5.**
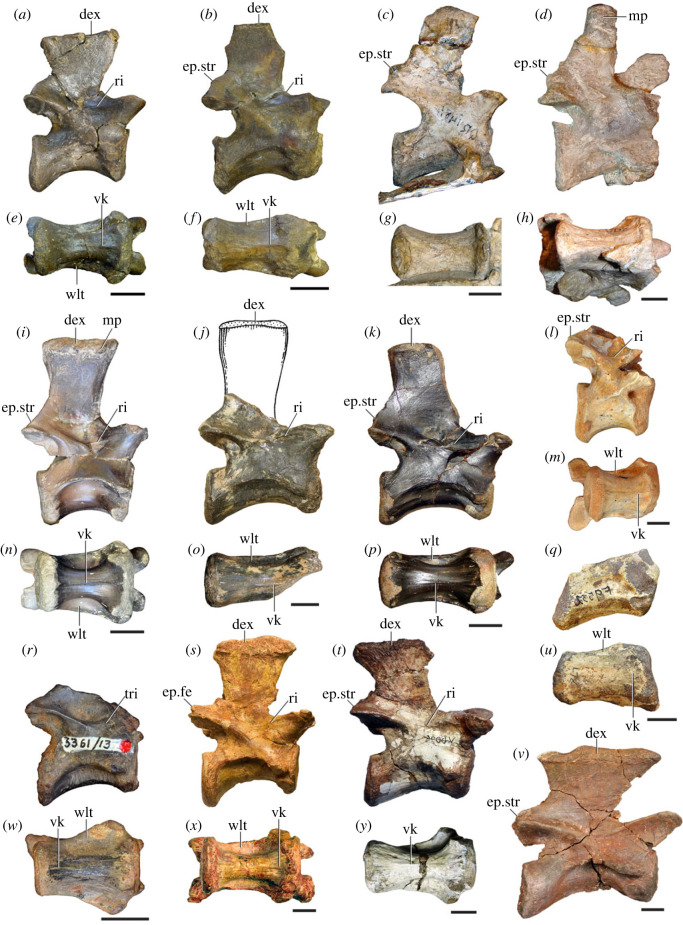
Comparison between third to sixth cervical vertebrae of selected late Permian to Middle Triassic archosauriforms. (*a, e*) *Samsarasuchus pamelae* (Cv4, ISIR 1080, paratype), (*b, f*) *Samsarasuchus pamelae* (Cv5, ISIR 1084), (*c, g*) *Proterosuchus fergusi* (Cv4, NMQR 1484, reversed), (*d, h*) *Proterosuchus fergusi* (Cv6?, BP/1/3993, reversed), (*i, n*) *Chasmatosuchus rossicus* (probable Cv4, PIN 3200/217, reversed), (*j, o*) *Tsylmosuchus samariensis* (probable Cv5, PIN 2424/6, holotype; line drawing in (*j*) outlines the currently missing neural spine following Sennikov [112]: figure 1), (*k, p*) *Tsylmosuchus jakovlevi* (probable Cv4, PIN 4332/1, holotype, reversed), (*l, m*) *Archosaurus rossicus* (probable Cv5, PIN 1100/66b, reversed), (*q, u*) ‘Arcadia proterosuchian’ (Cv3–4, QMF9532, reversed), (*r, w*) *Gamosaurus lozovskii* (probable Cv5, PI N 3361/13, holotype), (*s, x*) *Jaikosuchus magnus* (probable Cv5, PIN 951/65, holotype, reversed), (*t*) *Xilousuchus sapingensis* (Cv4, IVPP V6026, holotype, reversed), (*v*) *Teleocrater rhadinus* (probable Cv5, NMT RB512, reversed), and (*y*) *Xilousuchus sapingensis* (Cv5, IVPP V6026, holotype) in (*a–d, i–l, q–t, v*) lateral and (*e–h, m–p, u, w–y*) ventral views. dex, distal expansion; ep.fe, epipophysis with free end; ep.str, epipophysis with subtle transition; mp, mammillary process; ri, ridge; tri, thick ridge; vk, ventral keel; wlt, wing-like tuberosity. Scale bars equal 1 cm.

**Figure 6.**
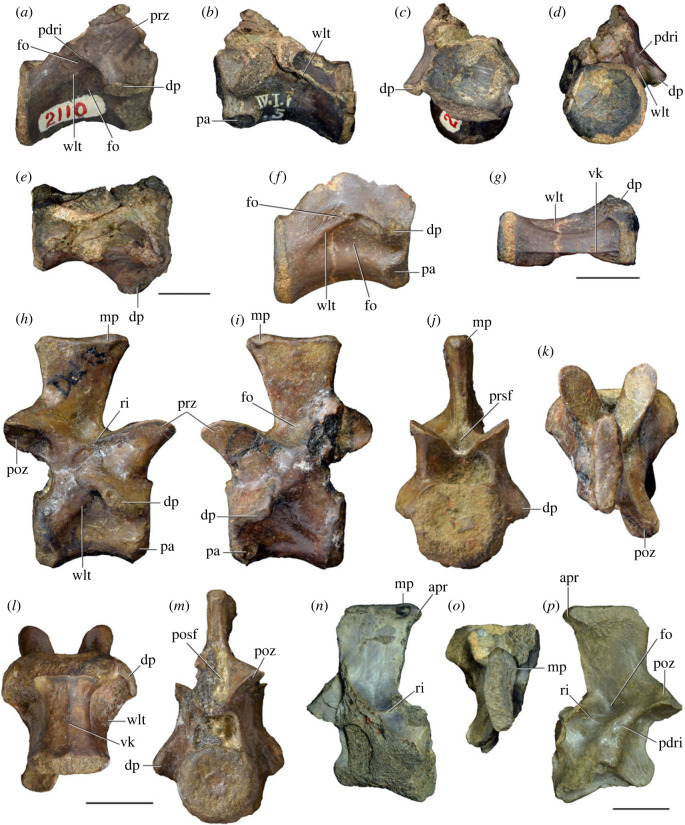
Sixth and seventh cervical vertebrae of *Samsarasuchus pamelae* gen. et sp. nov. (*a–e*) Sixth cervical vertebra (GSI 2110), (*f, g*) sixth cervical vertebra (NHMUK PV R 37587), (*h–m*) seventh cervical vertebra (NHMUK PV R 37580), and (*n–p*) seventh cervical vertebra (ISIR 1087) in (*a, f, h, n*) right lateral, (*b, i, p*) left lateral, (*c, j*) anterior, (*d, m*) posterior, (*e, k, o*) dorsal, and (*g, l*) ventral views. apr, anterior projection; dp, diapophysis; fo, fossa; mp, mammillary process; pa, parapophysis; pdri, posterodorsally oriented ridge; posf, postspinal fossa; poz, postzygapophysis; prsf, prespinal fossa; prz, prezygapophysis; ri, ridge; vk, ventral keel; wlt, wing-like tuberosity. Scale bars equal 1 cm.

**Figure 7.**
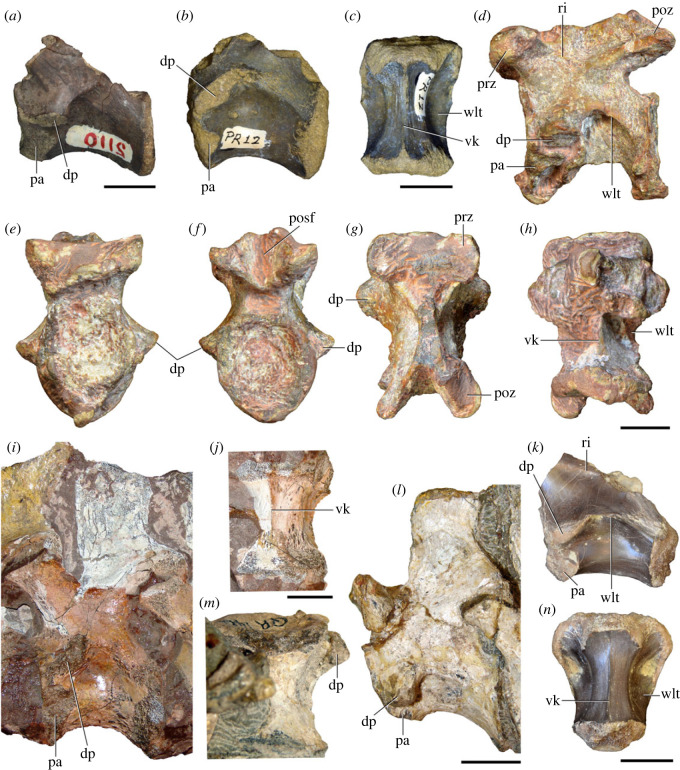
Comparison between sixth cervical vertebrae of selected Early Triassic non-eucrocopodan archosauriforms. (*a*) *Samsarasuchus pamelae* (GSI 2110, reversed), (*b, c*) *Samsarasuchus pamelae* (ISIR 1086), (*d–h*) ‘Arcadia proterosuchian’ (QMF60371), (*i, j*) *Proterosuchus fergusi* (SAM-PK-11208, reversed), (*m, l*) *Proterosuchus alexanderi* (NMQR 1484), and (*k, n*) *Chasmatosuchus rossicus* (PIN 3200/472, reversed) in (*a, b, d, i, l, k*) lateral, (*c, h, j, m, n*) ventral, (*e*) anterior, (*f*) posterior, and (*g*) dorsal views. dp, diapophysis; pa, parapophysis; posf, postspinal fossa; poz, postzygapophysis; prz, prezygapophysis; ri, ridge; vk, ventral keel; wlt, wing-like tuberosity. Scale bars equal 1 cm.

**Figure 8.**
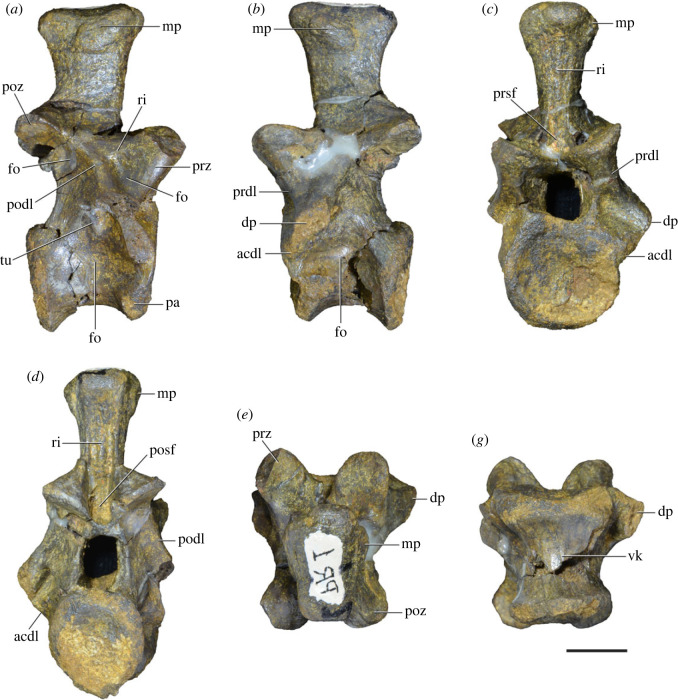
Eighth cervical vertebra of *Samsarasuchus pamelae* gen. et sp. nov. (ISIR 1090) in (*a*) right lateral, (*b*) left lateral, (*c*) anterior, (*d*) posterior, (*e*) dorsal, and (*g*) ventral views. acdl, anterior centrodiapophyseal lamina; dp, diapophysis; fo, fossa; mp, mammillary process; pa, parapophysis; podl, postzygodiapophyseal lamina; posf, postspinal fossa; poz, postzygapophysis; prdl, prezygodiapophyseal lamina; prsf, prespinal fossa; prz, prezygapophysis; ri, ridge; tu, tuberosity; vk, ventral keel. Scale bar equals 1 cm.

**Figure 9.**
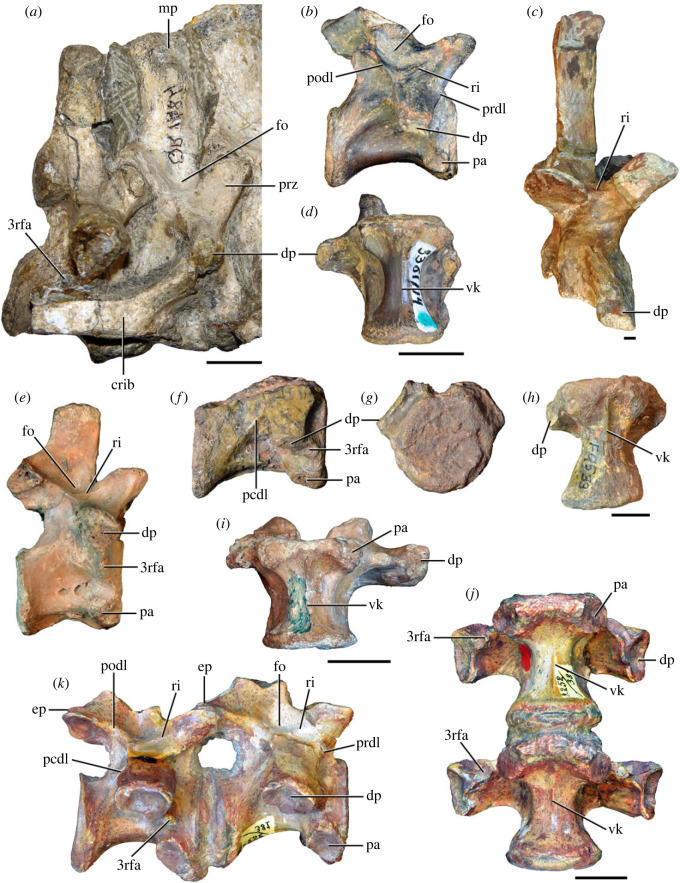
Comparison between eighth and ninth cervical vertebrae of selected Early and Middle Triassic non-eucrocopodan archosauriforms. (*a*) *Proterosuchus alexanderi* (Cv8−9, NMQR1484, reversed), (*b, d*) *Gamosaurus lozovskii* (probable Cv8, PIN 3361/14), (*c*) *Erythrosuchus africanus* (probable Cv8, NHMUK PV R3592), (*e, i*) *Vonhuenia friedrichi* (probable Cv9, PIN 1025/11, holotype, (*e*) reversed), (*f–h*), ‘Arcadia proterosuchian’ (probable Cv8, QMF9533), and (*j, k*) *Chasmatosuchus rossicus* (probable Cv8−9, PIN 2252/381, holotype, K reversed) in (*a–c, e, f, k*) lateral, (*d, h, i, j*) ventral, and (*g*) anterior views. 3rfa, third rib facet; crib, cervical rib; dp, diapophysis; ep, epipophysis; fo, fossa; mp, mammillary process; pa, parapophysis; pcdl, posterior centrodiapophyseal lamina; podl, postzygodiapophyseal lamina; prdl, prezygodiapophyseal lamina; prz, prezygapophysis; ri, ridge; vk, ventral keel. Scale bars equal 1 cm.

**Figure 10.**
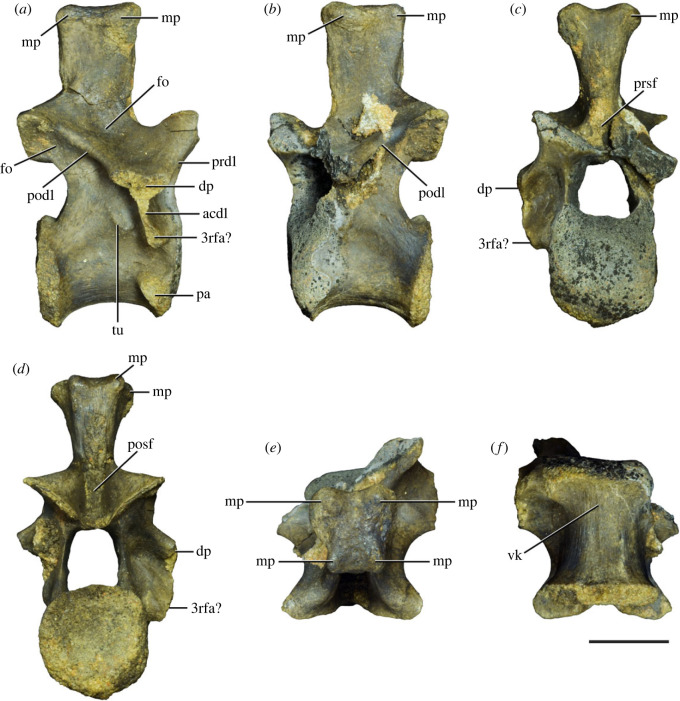
Ninth cervical vertebra of *Samsarasuchus pamelae* gen. et sp. nov. (ISIR 1091, holotype) in (*a*) right lateral, (*b*) left lateral, (*c*) anterior, (*d*) posterior, (*e*) dorsal, and (*f*) ventral views. 3rfa, third rib facet; acdl, anterior centrodiapophyseal lamina; dp, diapophysis; fo, fossa; mp, mammillary process; pa, parapophysis; podl, postzygodiapophyseal lamina; posf, postspinal fossa; prdl, prezygodiapophyseal lamina; prsf, prespinal fossa; tu, tuberosity; vk, ventral keel. Scale bar equals 1 cm.

**Figure 11.**
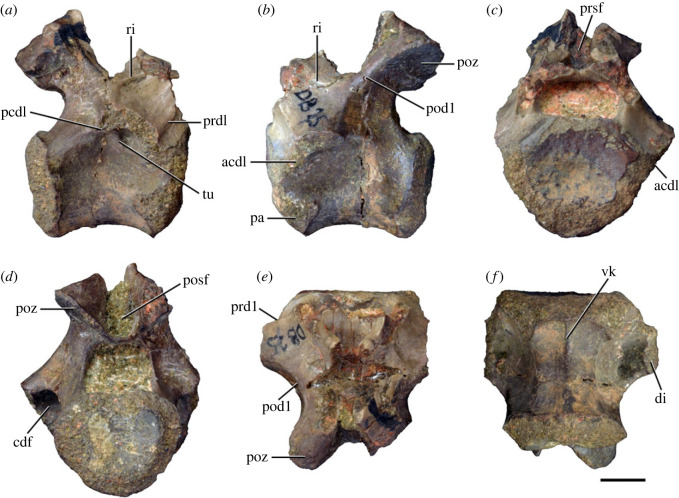
Probable first dorsal vertebra of *Samsarasuchus pamelae* gen. et sp. nov. (NHMUK PV R37583) in (*a*) right lateral, (*b*) left lateral, (*c*) anterior, (*d*) posterior, (*e*) dorsal, and (*f*) ventral views. acdl, anterior centrodiapophyseal lamina; cdf, centrodiapophyseal fossa; dp, diapophysis; pa, parapophysis; pcdl, posterior centrodiapophyseal lamina; podl, postzygodiapophyseal lamina; posf, postspinal fossa; poz, postzygapophysis; prdl, prezygodiapophyseal lamina; prsf, prespinal fossa; ri, ridge; tu, tuberosity; vk, ventral keel. Scale bar equals 5 mm.

**Figure 12.**
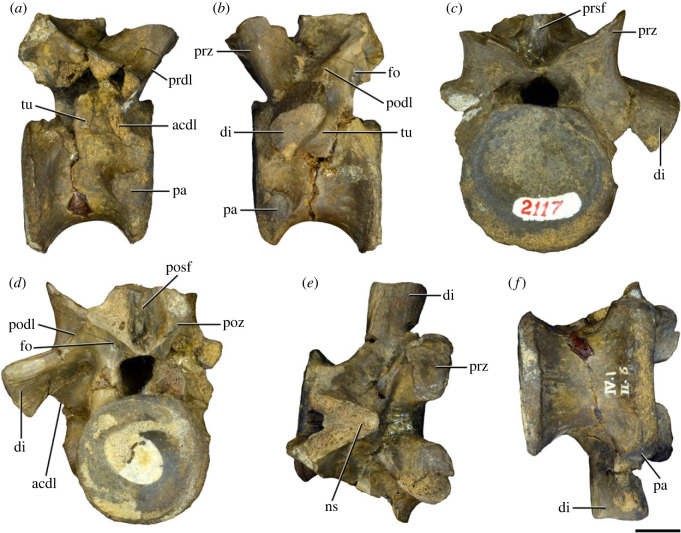
Probable second dorsal vertebra of *Samsarasuchus pamelae* gen. et sp. nov. (GSI 2117) in (*a*) right lateral, (*b*) left lateral, (*c*) anterior, (*d*) posterior, (*e*) dorsal, and (*f*) ventral views. acdl, anterior centrodiapophyseal lamina; dp, diapophysis; fo, fossa; ns, neural spine; pa, parapophysis; podl, postzygodiapophyseal lamina; posf, postspinal fossa; poz, postzygapophysis; prdl, prezygodiapophyseal lamina; prsf, prespinal fossa; prz, prezygapophysis; tu, tuberosity. Scale bar equals 1 cm.

**Figure 13.**
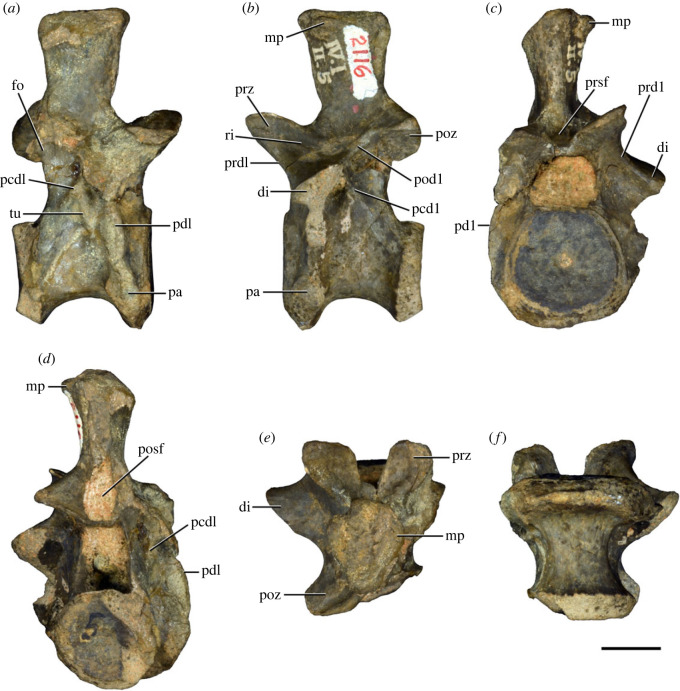
Probable third dorsal vertebra of *Samsarasuchus pamelae* gen. et sp. nov. (GSI 2116) in (*a*) right lateral, (*b*) left lateral, (*c*) anterior, (*d*) posterior, (*e*) dorsal, and (*f*) ventral views. dp, diapophysis; fo, fossa; mp, mammillary process; pa, parapophysis; pcdl, posterior centrodiapophyseal lamina; pdl, paradiapophyseal lamina; podl, postzygodiapophyseal lamina; posf, postspinal fossa; poz, postzygapophysis; prdl, prezygodiapophyseal lamina; prsf, prespinal fossa; prz, prezygapophysis; ri, ridge; tu, tuberosity. Scale bar equals 1 cm.

**Figure 14.**
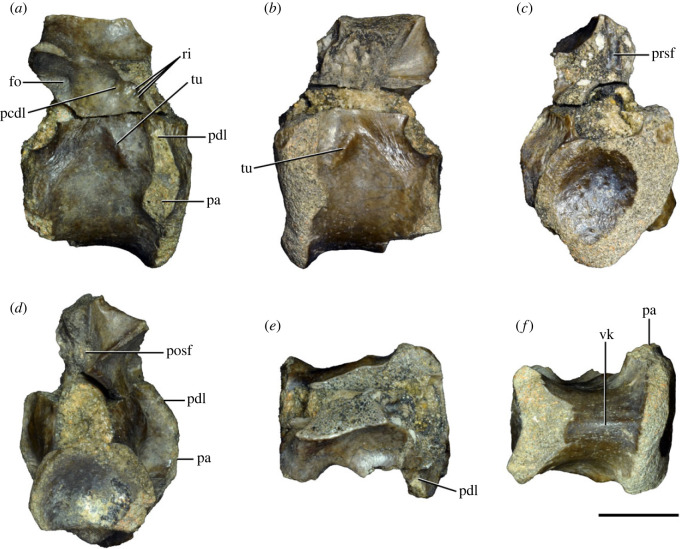
Probable fourth dorsal vertebra of *Samsarasuchus pamelae* gen. et sp. nov. (ISIR 1098) in (*a*) right lateral, (*b*) left lateral, (*c*) anterior, (*d*) posterior, (*e*) dorsal, and (*f*) ventral views. fo, fossa; pa, parapophysis; pcdl, posterior centrodiapophyseal lamina; pdl, paradiapophyseal lamina; posf, postspinal fossa; prsf, prespinal fossa; ri, ridge; tu, tuberosity; vk, ventral keel. Scale bar equals 1 cm.

**Figure 15.**
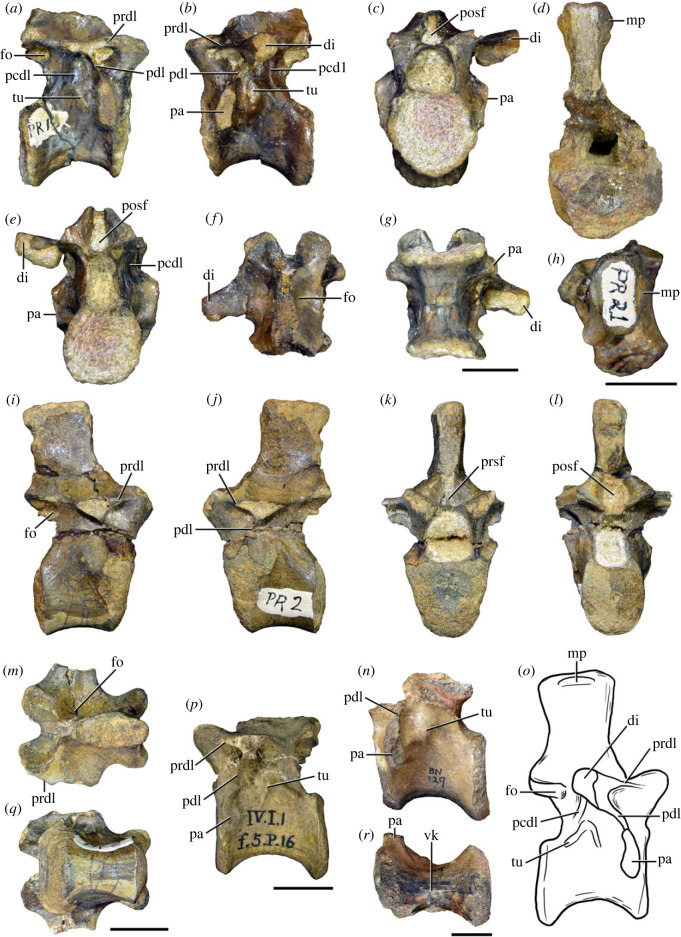
Probable fifth−sixth dorsal vertebrae of *Samsarasuchus pamelae* gen. et sp. nov. (*a–c, e–g*) ISIR 1096, (*d, h*) ISIR 1097, (*i–m, q*) ISIR 1094, (*n, r*) NHMUK PV R37577, (*o*) reconstructed vertebra (based on ISIR 1094, ISIR 1096, ISIR 1097, NHMUK PV R37577, and GSI 2260), and (*p*) GSI 2260 in (*a, i, o*) right lateral, (*b, j, n, p*) left lateral, (*c, d, k*) anterior, (*e, l*) posterior, (*f, h, m*) dorsal, and (*g, q, r*) ventral views. dp, diapophysis; fo, fossa; mp, mammillary process; pa, parapophysis; pcdl, posterior centrodiapophyseal lamina; pdl, paradiapophyseal lamina; posf, postspinal fossa; prdl, prezygodiapophyseal lamina; prsf, prespinal fossa; tu, tuberosity; vk, ventral keel. Scale bars equal 1 cm.

**Figure 16.**
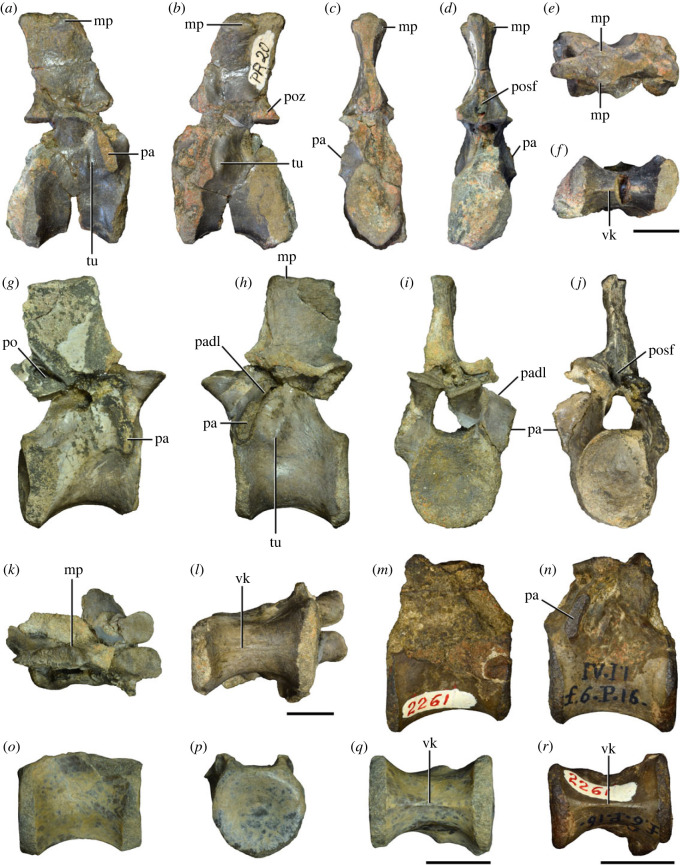
Middle dorsal (probable seventh to eleventh) vertebrae of *Samsarasuchus pamelae* gen. et sp. nov. (*a–f*) ISIR 1101, (*g–l*) ISIR 1102, (*m, n, r*) GSI 2261, and (*o–q*) ISIR 1104 in (*a, g, m, o*) right lateral, (*b, h, n*) left lateral, (*c, i*) anterior, (*d, j, p*) posterior, (*e, k*) dorsal, and (*f, l, q, r*) ventral views. mp, mammillary process; pa, parapophysis; padl, paradiapophyseal lamina; po, pocket; posf, postspinal fossa; poz, postzygapophysis; tu, tuberosity; vk, ventral keel. Scale bars equal 1 cm.

**Table 2.** Measurements in millimetres of cervical vertebrae 2–4 of Panchet cf. proterosuchid specimens (Cv2) and *Samsarasuchus pamelae* gen. et sp. nov (Cv3–4). Cv2a (ISIR 1079), Cv2b (NHMUK PV R37582), Cv3a (GSI 2111), Cv3/4a (GSI 2109), Cv3/4b (NHMUK PV R37578), Cv4a (ISIR 1080, paratype) and Cv4b (ISIR 1085). Cv, cervical vertebra. Values with an asterisk indicate incomplete measurements (owing to post-mortem damage) and the value given is the maximum measurable. The maximal deviation of the callipers is 0.02 mm, but measurements were rounded to the nearest 0.1 mm.

	Cv2a	Cv2b	Cv3a	Cv3/4a	Cv3/4b	Cv4a	Cv4b
length of centrum	25.7	40.0	25.2	27.1	21.4	27.7	30.7
anterior height of centrum	8.7*	34.3	13.4	14.9	13.2*	15.8	17.5
anterior width of centrum	17.8*	25.7	[12.6]	15.4	14.7	16.0	20.1
posterior height of centrum	10.0*	32.1	12.2	16.0	13.5*	16.5	19.2
posterior width of centrum	18.0	[27.0]	11.9	16.8	15.0	16.2	18.4
length across zygapophyses	36.3	48.8	30.5	—	24.7*	35.8	33.3*
height neural spine	8.2*	42.8	15.1*	—	19.1	22.2	9.0*
length neural spine at base	—	38.6	16.7	—	—	14.9	13.0*
maximum height	29.8*	85.4	32.6*	28.5*	41.1*	48.8	40.0*

**Table 3.** Measurements in millimetres of cervical vertebrae 5–6 of *Samsarasuchus pamelae* gen. et sp. nov. Cv5a (GSI 2115), Cv5b (ISIR 1081), Cv5c (ISIR 1084), Cv6a (GSI 2110) and Cv6b (NHMUK PV R37587). Cv, cervical vertebra. Values with an asterisk indicate incomplete measurements (owing to post-mortem damage) and the value given is the maximum measurable. The maximal deviation of the callipers is 0.02 mm, but measurements were rounded to the nearest 0.1 mm.

	Cv5a	Cv5b	Cv5c	Cv6a	Cv6b
length of centrum	28.3	29.3	29.6	26.1	22.6
anterior height of centrum	17.0	16.1	12.8*	15.5*	14.5
anterior width of centrum	16.8	15.2	13.7*	16.7	7.6*
posterior height of centrum	17.9	16.0	15.7	16.2	14.4*
posterior width of centrum	17.1*	16.6	14.1*	16.5	7.6*
length across zygapophyses	—	34.4*	34.9	—	—
height neural spine	13.4*	19.5	20.9	—	—
length neural spine at base	16.9	15.9*	15.2	—	—
maximum height	41.2*	45.0	44.7	27.4*	21.7*

**Table 4.** Measurements in millimetres of cervical vertebrae 7–9 of *Samsarasuchus pamelae* gen. et sp. nov. Cv7a (NHMUK PV R37580), Cv7b (ISIR 1087), Cv7c (ISIR 1088), Cv7d (ISIR 1089), Cv8 (ISIR 1090) and Cv9 (ISIR 1091, holotype). Cv, cervical vertebra. Values with an asterisk indicate incomplete measurements (owing to post-mortem damage) and the value given is the maximum measurable. The maximal deviation of the callipers is 0.02 mm, but measurements were rounded to the nearest 0.1 mm.

	Cv7a	Cv7b	Cv7c	Cv7d	Cv8	Cv9
length of centrum	17.6	—	14.7*	18.5	21.2	18.3
anterior height of centrum	12.4*	—	12.3	13.2*	18.5	13.5
anterior width of centrum	14.4	—	11.7*	12.9*	17.9	13.8
posterior height of centrum	11.8*	—	—	12.7	17.6*	14.3
posterior width of centrum	12.1*	—	—	12.9	14.9*	13.6*
length across zygapophyses	25.5	24.4*	—	—	27.3	23.2
height neural spine	15.9	20.1	—	—	21.4	17.3
length neural spine at base	9.3	12.2	9.4	—	10.9	8.6
maximum height	34.8*	35.7*	23.4*	22.6*	51.5*	40.7

**Table 5.** Measurements in millimetres of anterior dorsal vertebrae of *Samsarasuchus pamelae* gen. et sp. nov. ADa (NHMUK PV R37583: ?D1), ADb (GSI 2117: ?D2), ADc (GSI 2116: ?D3), ADd (ISIR 1092: ?D3), ADe (GSI 2260: ?D5–6), ADf (NHMUK PV R37577: ?D5–6), ADg (ISIR 1098: ?D4), and ADh (ISIR 1099: ?D4). AD, anterior dorsal vertebra. Values with an asterisk indicate incomplete measurements (owing to post-mortem damage) and the value given is the maximum measurable. The maximal deviation of the callipers is 0.02 mm, but measurements were rounded to the nearest 0.1 mm.

	ADa	ADb	ADc	ADd	ADe	ADf	ADg	ADh
length of centrum	17.8	28.4	22.1	15.3*	18.5	26.9	20.5	38.8
anterior height of centrum	13.4*	32.4	19.4	12.1	14.7	19.7*	16.4	34.9*
anterior width of centrum	15.5	33.9	19.7	14.4	14.1	16.9*	[14.7]	24.2*
posterior height of centrum	11.8*	30.7	16.2*	10.9*	14.3	20.9	10.9*	35.1*
posterior width of centrum	14.0*	30.5	16.8*	11.6*	12.8	19.4*	15.1	29.9*
length across zygapophyses	—	36.1*	27.5	—	21.0*	—	16.4*	—
height neural spine	—	—	19.6	—	—	—	—	—
length neural spine at base	—	13.6	10.2	—	—	—	—	—
maximum height	25.4*	55.1*	50.7*	14.2*	27.6*	36.4*	32.5*	55.3*

**Table 6.** Measurements in millimetres of anterior and middle dorsal vertebrae of *Samsarasuchus pamelae* gen. et sp. nov. ADi (ISIR 1094: ?D5–6), ADj (ISIR 1096: ?D5–6), MDa (ISIR 1101), MDb (ISIR 1102), MDc (GSI 2261), and MDe (ISIR 1103). AD, anterior dorsal vertebra; MD, middle dorsal vertebra. Values with an asterisk indicate incomplete measurements (owing to post-mortem damage) and the value given is the maximum measurable. The maximal deviation of the callipers is 0.02 mm, but measurements were rounded to the nearest 0.1 mm.

	ADi	ADj	MDa	MDb	MDc	MDe
length of centrum	17.3*	20.6	22.5	24.9	17.3	17.2
anterior height of centrum	13.5*	14.7	17.4*	18.6	13.0	12.0
anterior width of centrum	13.3*	15.6	—	19.0	12.9	11.0
posterior height of centrum	12.0*	14.4	17.3*	20.3	12.7*	10.9
posterior width of centrum	10.7*	13.9*	—	[18.6]	12.1*	10.0
length across zygapophyses	22.1*	22.9*	—	33.6	—	—
height neural spine	16.7*	5.6*	23.6	20.6	—	—
length neural spine at base	11.0	12.4*	15.0	16.5	—	—
maximum height	40.7*	31.9*	52.4*	54.4	24.0*	16.2*

### 3.7. LSID

urn:lsid:zoobank.org:act:553454BE-4D1A-4C2C-918D-A4030A0A297D, urn:lsid:zoobank.org:act:A6423008-10FE-4335-9961-FC923111AEFE.

### 3.8. Etymology

The genetic epithet is formed by the Sanskrit word ‘Saṁsāra’ (Samsara) that in Hinduism is related to the cycle of re-birth, existence and death, and ‘*Σο*ῦ*χο*ς’ (Suchus), which is the name of the Egyptian crocodile-headed deity Sebek or Sobek in ancient Greek, referring to the re-birth of ecosystems after the end-Permian mass extinction and the most common ending (-suchus) of archosauromorph genera. The specific epithet is for the first name of the British palaeontologist Pamela Lamplugh Robinson (1919–1994) in honour of her contributions to Indian vertebrate palaeontology and especially for having prompted a renewed interest in the vertebrate palaeontology of the Panchet Formation in the 1960s after a long gap in research since the end of the ninteenth century. In addition, Pamela Robinson led the fieldtrip that resulted in the discovery of several referred specimens of this new proterosuchid species (NHMUK collection).

### 3.9. Holotype

ISIR 1091, nearly complete ninth cervical vertebra (Cv [cervical] 9) lacking the left prezygapophysis and most of the left diapophysis.

### 3.10. Paratype

ISIR 1080, complete isolated probable fourth cervical vertebra (Cv4). We designate a paratype because it allows a direct comparison and distinction with the holotype of several Early Triassic Russian species (i.e. *Gamosaurus lozovskii*, *Jaikosuchus magnus*, *Tsylmosuchus jakovlevi*, *Tsylmosuchus samariensis*), which all represent anterior cervical vertebrae.

Referred specimens ISIR 1098 (anterior dorsal vertebra) and ISIR 1102 (middle dorsal vertebra) were found a few metres from ISIR 1080 but, although they are of a size congruent with that which would be expected for a single individual, we cannot confirm it because of the taphonomic settings of the locality at which they were found (see above).

### 3.11. Type horizon and locality

The type series comes from the yellow-brownish conglomeratic sandstones of the upper Panchet Formation (Early Triassic: middle−late Induan), Damodar Basin at the Damodar River bed locality, near Deoli village (holotype: 23°39′03.2″ N, 86°53′03.4″ E; paratype: 23°39′03.5″ N, 86°53′03.3″ E), West Bengal, east India (figure 1). The holotype and paratype were found ten metres apart from one another in approximately the same stratigraphic level on 18 January 2015.

### 3.12. Referred specimens

GSI 2111 (Huxley [58]: plate I, figure 6), Cv3; GSI 2109 (Huxley [58]: plate I, figure 4), ISIR 1082, ISIR 1083, NHMUK PV R37578, PGRU/GL/M/VF-002, Cv3 or Cv4; ISIR 1085, Cv4; GSI 2115 (Huxley [58]: plate II, figure 4), ISIR 1081, ISIR 1084, Cv5; GSI 2110 (Huxley [58]: plate I, figure 5), ISIR 1086, NHMUK PV R37587, Cv6; ISIR 1087–1089, NHMUK PV R37580, Cv7; ISIR 1090, Cv8; ISIR 1108, partial neural spine of middle−posterior cervical or middle dorsal vertebra; ISIR 1100, distal end of neural spine of posterior cervical or anterior dorsal vertebra; GSI 2116 (Huxley [58]: plate II, figure 5), GSI 2117 (Huxley [58]: plate II, figure 6), GSI 2260 (Huxley [58]: figure 5), ISIR 1092, ISIR 1094–1099, NHMUK PV R37583, NHMUK PV R37577, anterior dorsal vertebrae; GSI 2261 (Huxley [58]: figure 6), ISIR 1101–1103, middle dorsal vertebra; ISIR 1104, probable middle dorsal centrum; ISIR 1093, partial dorsal neural arch.

### 3.13. Diagnosis

*Samsarasuchus pamelae* is a chasmatosuchine proterosuchid that differs from other non-archosaurian archosauriforms in the following autapomorphies (among non-archosaurian archosauriforms) present in its holotype (ninth cervical vertebra): posteriormost cervical vertebra (ninth cervical vertebra) with two pairs (i.e. four in total) of mammillary processes on the neural spine; and posteriormost cervical vertebra (ninth cervical vertebra) with dorsolaterally oriented mammillary processes on the anterior region of the neural spine. In addition, the holotype of *Samsarasuchus pamelae* and the other postaxial cervical and anterior–middle dorsal vertebrae referred to this species share the following unique combination of character states that allow the species to be distinguished from other non-erythrosuchid, non-eucrocopod archosauriform nominal species: third cervical to anterior dorsal vertebrae (*ca* third dorsal vertebra) with a thin anterodorsally-to-posteroventrally oriented ridge that reaches the base of the prezygapophysis on the dorsolateral surface of the neural arch (absent in *Proterosuchus fergusi*, *Proterosuchus alexanderi* and *Sarmatosuchus otschevi*); third cervical to middle dorsal vertebrae with height of neural spine 1.00–1.34 times the height of the centrum (higher ratios present in *Proterosuchus alexanderi*, *Jaikosuchus magnus*, *Tsylmosuchus jakovlevi*, *Tsylmosuchus samariensis*, *Chasmatosuchus rossicus*); seventh cervical to at least middle dorsal vertebrae (*ca* tenth dorsal vertebra) with mammillary processes close to the dorsal surface of the neural spine (also present in *Proterosuchus alexanderi*, but mammillary processes restricted more anteriorly in the dorsal series in *Proterosuchus fergusi* and ‘*Chasmatosaurus*’ *yuani* or processes absent in *Vonhuenia friedrichi* and *Sarmatosuchus otschevi*); seventh cervical to anterior–middle dorsal vertebrae without epipophysis (epipophysis present in *Proterosuchus alexanderi* and *Chasmatosuchus rossicus*); eighth cervical to middle dorsal vertebrae with posterodorsally-to-anteroventrally oriented tuberosity ventral to the diapophysis at or close to the level of the neurocentral suture (also present in ‘*Chasmatosaurus*’ *yuani*, *Chasmatosuchus rossicus*, *Gamosaurus lozovskii*, *Sarmatosuchus otschevi* and *Vonhuenia friedrichi*; absent in *Proterosuchus fergusi*, *Proterosuchus alexanderi*); and ninth cervical to at least anterior dorsal vertebrae (*ca* fifth−sixth dorsal vertebrae) with a prezygodiapophyseal lamina (also present in *Vonhuenia friedrichi*, *Chasmatosuchus rossicus*, *Gamosaurus lozovskii* and *Sarmatosuchus otschevi*; absent in *Proterosuchus fergusi*, *Proterosuchus alexanderi* and ‘*Chasmatosaurus*’ *yuani*). This unique combination of character states supports the assignment of all these postaxial cervical to middle dorsal vertebrae as referred specimens of *Samsarasuchus pamelae*.

### 3.14. Geographic occurrence of referred specimens

All GSI specimens, ISIR 1087, ISIR 1098, ISIR 1092, ISIR 1093, ISIR 1099, ISIR 1101 and ISIR 1102 were collected in the Deoli locality, close to Deoli village; ISIR 1094, ISIR 1103, ISIR 1104, NHMUK PV R37580 and PGRU/GL/M/VF-002 were collected in the Dumdumi locality, close to Dumdumi village; NHMUK PV R37583, NHMUK PV R37585 and NHMUK PV R37587 were collected in the riverbank of the Damodar River, besides the Railway Bridge; and NHMUK PV R37577 and NHMUK PV R37578 were collected in the Banspatali locality (=Banspetali locality in Das & Gupta [73]: figure 1). Locality data for the GSI specimens are from Satsangi [45] and Blanford (in Huxley [58]), data from PGRU/GL/M/VF specimens is from S. Pal (pers. comm. 2021), data from the NHMUK specimens are from the records of that institution, and data from the ISI specimens is from our own field records. Specimens not mentioned here lack precise locality data. All the localities occur in the west of West Bengal, northeast India.

### 3.15. Stratigraphic occurrence of referred specimens

All the referred specimens come from the yellow-brownish conglomeratic sandstones of the upper Panchet Formation (middle–late Induan), Damodar Basin.

## 4. Description of *Samsarasuchus pamelae*

We decided to describe the complete hypodigm (type series and referred specimens) of *Samsarasuchus pamelae* and the cf. proterosuchid and proterosuchid bones (see below) of the Panchet Formation in two different description sections. Bones are described following traditional anatomical order and in subsections of elements with similar morphology for the sake of practicality. As a result, if in the future it is determined that a specimen or a particular portion of the skeleton belongs or not to *Samsarasuchus pamelae*, it should be easy to identify the relevant information in the following descriptions. Comparisons are focused on non-eucrocopodan archosauriforms, but detailed comparisons with other groups are made if necessary, such as in the case of comparisons with the anterior−middle cervical vertebrae of aphanosaurian and early poposauroid archosaurs because of their similarities with those of *Samsarasuchus pamelae*.

### 4.1. Postcranial axial skeleton

Preserved vertebrae of the type series and specimens referred to *Samsarasuchus pamelae* include elements of most of the presacral series (figure 2).

#### 4.1.1. Cervical vertebrae

##### 4.1.1.1. Cervical vertebrae 3–5

There are 11 postaxial vertebrae that are interpreted as being third to fifth cervical vertebrae (Cv3–5, = postaxial anterior cervical vertebrae; figures 3–5*a,b,e,f*, tables 2 and 3). GSI 2109 (Huxley [58]: plate I, figure 4) preserves a complete centrum and both prezygapophyses, although the anterior margin of the right prezygapophysis is damaged. GSI 2111 (Huxley [58]: plate I, figure 6; figure 3*a–f*) is fairly complete, only missing the right prezygapophysis and the distal end of the neural spine. However, it seems that most of the neural spine was originally complete (Huxley [58]: plate I, figure 6) and was lost after the description of Huxley [58]. The articular surfaces of the parapophyses and diapophyses and the anterior margin of the neural spine are damaged. GSI 2115 (Huxley [58]: plate II, figure 4) lacks the right parapophysis and diapophysis, both prezygapophyses, part of the right postzygapophysis and the distal end of the neural spine. ISIR 1080 is an almost complete vertebra (figure 4*a–f*). NHMUK PV R37578 is fairly complete, lacking most of the postzygapophyses and the posterodorsal tip of the neural spine (figure 3*g–l*). ISIR 1081 lacks the right zygapophyses, the distal end of the left prezygapophysis and the anterodorsal end of the neural spine (figure 4*g–l*). ISIR 1082 lacks the posterior half of the centrum, right prezygapophysis and most of the left prezygapophysis and postzygapophysis, neural spine and distal tip of the right postzygapophysis. ISIR 1083 lacks the posterior end of the centrum, right prezygapopysis, both postzygapophyses, neural spine, most of the left prezygapophysis and the parapophyses and diapophyses are damaged. ISIR 1084 lacks the distal tips of the parapophyses, diapophyses, prezygapophyses and left postzygapophysis, the anterodistal and posterodistal corners of the neural spine, and the anterior and posterior surfaces of the centrum are damaged (figure 4*m*,*n*). ISIR 1085 lacks most of the left prezygapophysis and neural spine, both postzygapophyses and the left diapophysis is damaged. Finally, PGRU/GL/M/VF-002 (Pal [46]: figure 2) lacks the posterior half of the centrum and the left prezygapophysis.

These vertebrae are interpreted to belong to the third to fifth cervical positions because the parapophysis and diapophysis are situated on the anteroventral corner of the centrum, closely approaching each other, the diapophysis is restricted to the anterior third and mid-height of the centrum, and the tuberosity that projects posteriorly from the diapophysis is parallel to the ventral margin of the centrum. This combination of features is present in the Cv3–5 of *Proterosuchus alexanderi* (NMQR 1484) and *Proterosuchus fergusi* (BP/1/3993, SNSB-BSPG 1934 VIII 514; SAM-PK-11208) (figure 5*c,d*). Within this region of the cervical series, one specimen (GSI 2111) is identified as a Cv3 because the diapophysis is adjacent to the anterior margin of the centrum, mainly ventrally oriented, and the tuberosity that projects posteriorly from it is restricted to the anterior third of the centrum (figure 3*a–f*). Two specimens (the paratype ISIR 1080 and ISIR 1085) are probably Cv4 because the diapophysis is still adjacent to the anterior margin of the centrum, but more lateroventrally oriented, and the tuberosity that projects from it is better developed laterally and reaches the posterior third of the centrum (figure 4*a–f*). Five specimens (GSI 2109, ISIR 1082, ISIR 1083, NHMUK PV R37578, PGRU/GL/M/VF-002) show a similar morphology, but with a less clear differentiation of the above-mentioned rib facet features; thus, they are identified as either Cv3 or Cv4 (e.g. figure 3*g–l*). Three specimens (GSI 2115, ISIR 1081, ISIR 1084) have a diapophysis that is distinctly posteriorly displaced from the anterior margin of the centrum, lateroventrally oriented and proportionally longer, and the tuberosity that projects from it is well developed laterally and reaches the posterior third of the centrum (figure 4*g–n*). These specimens are interpreted as fifth cervical vertebrae.

Regarding other features—excluding the position and development of the rib facets—there is a considerable amount of intraspecific morphological variation in articulated sequences of the anterior postaxial cervical vertebrae of specimens of *Proterosuchus* spp. from South Africa (e.g. orientation of the neural spine, development of the ventral keel on the centrum; BP/1/3993, SNSB-BSPG 1934 VIII 514; SAM-PK-11208; NMQR 1484). Thus, although the Cv3–5 of *Samsarasuchus pamelae* have some other differences that may indicate serial variation within this region of the neck (see below), they seem to be unreliable indicators of position. The anterior postaxial cervical vertebrae of *Samsarasuchus pamelae* are compared with early archosauriform vertebral sequences that include Cv3–5 (e.g. *Proterosuchus fergusi*: BP/1/3993, BP/1/4016, SAM-PK 11208, SAM-PK-K140, SNSB-BSPG 1934 VIII 514; *Proterosuchus alexanderi*: NMQR 1484; *Sarmatosuchus otschevi*: [139]; *Garjainia prima*: [43,140]; *Bharitalasuchus tapani*: [102]), but also with isolated vertebrae that present the combination of parapophyseal and diapophyseal features listed above. These isolated vertebrae include the holotypes of *Gamosaurus lozovskii* (PIN 3361/13), *Jaikosuchus magnus* (PIN 951/65), *Tsylmosuchus jakovlevi* (PIN 4332/1) and *Tsylmosuchus samariensis* (PIN 2424/6), and specimens that have been referred to *Chasmatosuchus rossicus* (PIN 3200/217; [40]), cf. *Chasmatosuchus* (UNIPAMPA 750; [110]), *Archosaurus rossicus* (PIN 1100/66a, b; [27]), ‘*Blomosuchus georgii*’ (PIN 1025/420; [41]) and *Kalisuchus rewanensis* (QMF9532; [111]).

The 11 Cv3–5 of *Samsarasuchus pamelae* have an overall similar morphology despite the minor differences related to position and are described together. The centrum is 1.66–1.88 times longer than the height of its anterior articular surface, with larger individuals (i.e. those with broader centra) tending to possess proportionally shorter centra. This ratio overlaps the range present in Cv3–5 of *Proterosuchus fergusi* (1.73: BP/1/3993, Cv4; 1.53: SNSB-BSPG 1934 VIII 514, Cv3; 1.67: SAM-PK 11208, Cv5; 1.66: SAM-PK-K140, Cv3) and *Proterosuchus alexanderi* (1.40–1.95: NMQR 1484) (figure 5*c,d*), includes the ratio of a specimen referred to ‘*Blomosuchus georgii*’ (1.67: PIN 1025/420), is similar to that in the holotype of *Gamosaurus lozovskii* (1.92: PIN 3361/13; figure 5*r*) and *Jaikosuchus magnus* (1.94: PIN 951/65; figure 5*s*), and vertebrae referred to *Chasmatosuchus rossicus* (1.62: PIN 3200/217; figure 5*i*), the cf. *Chasmatosuchus* from Brazil (1.98: [110]), and the ‘Arcadia proterosuchian’ (1.89: QMF9532, figure 5*q*), but is considerably greater than that present in anterior cervical vertebrae referred to *Archosaurus rossicus* (1.36: PIN 1100/66a; 1.31: PIN 1100/66b; figure 5*l*), *Sarmatosuchus otschevi* (0.94–1.12: PIN 2865/68; [139]), and erythrosuchids (e.g. *Garjainia prima*: [43,140]; *Erythrosuchus africanus*: [141]; *Bharitalasuchus tapani*: [102]). By contrast, the length to anterior height ratios of the anterior cervical centra of the holotypes of *Tsylmosuchus jakovlevi* (2.32: PIN 4332/1; figure 5*j*) and *Tsylmosuchus samariensis* (2.54: PIN 2424/6; figure 5*k*) are greater than that in preserved vertebrae of *Samsarasuchus pamelae*.

The centra of the anterior cervical vertebrae of *Samsarasuchus pamelae* are moderately transversely compressed around mid-length in ventral view. The centrum is a parallelogram in lateral view, with the anterior articular surface positioned slightly dorsal to the posterior one, a very common feature among early archosauriforms [18]. The anterior and posterior articular facets of the centrum are concave and sub-circular. The deepest area of the articular facet is slightly displaced dorsal to the centre in both anterior and posterior articular facets and sometimes possesses a shallow notochordal pit. The ventral surface of the centrum has a sharp median longitudinal keel that is restricted to the anterior third of the centrum in some specimens (e.g. GSI 2111: Cv3, 2115: Cv5, ISIR 1080: Cv4, paratype, 1081: Cv5, 1083: Cv3/4, 1085: Cv4) and extends along most of the surface of the centrum but does not reach its posterior margin in others (e.g. GSI 2109: Cv3/4, ISIR 1084: Cv5). The ventral keel becomes lower towards the posterior end of the centrum in most of these vertebrae, but it increases in height in the posterior third of GSI 2109 (Cv3/4). A similarly developed longitudinal ventral keel is also present in the anterior postaxial cervical vertebrae of *Jaikosuchus magnus* (PIN 951/65, figure 5*x*), *Tsylmosuchus jakovlevi* (PIN 4332/1, figure 5*o*), *Tsylmosuchus samariensis* (PIN 2424/6, figure 5*p*), *Gamosaurus lozovskii* (PIN 3361/13, figure 5*w*), *Proterosuchus fergusi* (SAM-PK-K140), the ‘Arcadia proterosuchian’ (QMF9532, figure 5*u*), specimens referred to *Archosaurus rossicus* (PIN 1100/66a, 1100/66b; figure 5*m*), ‘*Blomosuchus georgii*’ (PIN 1025/420), cf. *Chasmatosuchus* from Brazil [110] and *Chasmatosuchus rossicus* (PIN 3200/217; figure 5*n*), *Sarmatosuchus otschevi* (PIN 2865/68), and erythrosuchids (e.g. *Garjainia prima*: [43,140]; *Erythrosuchus africanus*: [141]; *Bharitalasuchus tapani*: [102]). A longitudinal ventral keel is restricted to Cv3 in *Proterosuchus alexanderi* (NMQR 1484), in which the ventral surfaces of the fourth and fifth cervical centra are smoothly transversely convex. Beyond the longitudinal median keel, the ventral surface of the posterior third of the centrum of some specimens of *Samsarasuchus pamelae* also possesses a pair (e.g. GSI 2111: Cv3, 2115: Cv5, ISIR 1080: Cv4, paratype, 1085: Cv4) or two pairs (e.g. ISIR 1081: Cv5) of thin collateral ridges. These ridges possess different degrees of ventral development among the preserved specimens, and are particularly well developed in ISIR 1081 (Cv5; figure 4*m*). The ridges extend mainly parallel to the median longitudinal ridge, but they converge towards the median line anteriorly. They define a shallow transversely concave surface between them and the median longitudinal keel. Although these ridges are not present in GSI 2109 (Cv3/4), there is also a shallow transversely concave surface adjacent to the posterior third of the median longitudinal keel.

The parapophysis is positioned on the anteroventral corner of the centrum and its articular facet is sub-circular to oval, with an anteroposterior main axis, and concave. The lateral surface of the centrum is flat to slightly dorsoventrally convex. There is a circular, probably nutrient, foramen that pierces the lateral surface of the centrum immediately posterodorsal to the parapophysis in several specimens (e.g. GSI 2111: Cv3, 2115: Cv5, ISIR 1080: Cv4, paratype, 1083: Cv3/4, 1085: Cv4), but which seems to be absent in GSI 2109. The diapophysis is restricted to the anterior third of the centrum, lateroventrally directed and placed at mid-height with respect to the anterior articular facet. There is a tuberosity that extends posteriorly from the base of the diapophysis to near the posterior end of the centrum at its mid-height. This tuberosity curves ventrally, being parallel to the ventral margin of the centrum, and is low in Cv3 (GSI 2111), but becomes very well developed laterally (‘wing-like’ shelf *sensu* Ezcurra [18]) and posteriorly in Cv4 and Cv5. Indeed, although these tuberosities do not reach the posterior margin of the centrum, they extend along more than 70% of the length of the centrum in Cv4 and Cv5 of *Samsarasuchus pamelae* (e.g. ISIR 1080, 1081, 1084). Similarly developed, wing-like tuberosities that also extend along more than 70% of the length of the centrum are present in *Jaikosuchus magnus* (PIN 951/65, figure 5*x*), *Tsylmosuchus jakovlevi* (PIN 4332/1, figure 5*o*), *Tsylmosuchus samariensis* (PIN 2424/6, figure 5*p*), *Gamosaurus lozovskii* (PIN 3361/13, figure 5*w*), the ‘Arcadia proterosuchian’ (QMF9532, figure 5*u*), and specimens referred to *Archosaurus rossicus* (PIN 1100/66a, 1100/66b; figure 5*m*), cf. *Chasmatosuchus* from Brazil [110] and *Chasmatosuchus rossicus* (PIN 3200/217; figure 5*n*). A similarly laterally developed tuberosity that extends posteriorly from the diapophysis occurs in the anterior cervical vertebrae of some early poposauroid archosaurs (e.g. *Xilousuchus sapingensis*: IVPP V6026), being restricted to the anterior approximately 70% of the length of the centrum. An opposite condition to that of the above-mentioned taxa is present in *Prolacerta broomi* (BP/1/2675), *Teyujagua paradoxa* (UNIPAMPA 653 cast), *Proterosuchus fergusi* (e.g. SNSB-BSPG 1934 VIII 514; BP/1/3993, 4016; SAM-PK-K140), *Proterosuchus alexanderi* (NMQR 1484), a specimen referred to ‘*Blomosuchus georgii*’ (PIN 1025/420), *Sarmatosuchus otschevi* (PIN 2865/68) and erythrosuchids (e.g. *Garjainia prima*: [43,140]; *Erythrosuchus africanus*: [141]; *Bharitalasuchus tapani*: [102]), in which the tuberosity that extends posteriorly from the diapophysis is low and anteroposteriorly short. The neurocentral suture is closed in all the preserved postaxial anterior cervical vertebrae of *Samsarasuchus pamelae*.

The prezygapophysis is anterodorsally directed and extends anteriorly beyond the level of the anterior margin of the centrum. The dorsal margin of the prezygapophysis possesses a gentle flexure in lateral view. The postzygapophysis extends posteriorly slightly beyond the level of the posterior margin of the centrum. There is a very shallow lateral fossa immediately ventral to its base, as in *Jaikosuchus magnus* (PIN 951/65), *Tsylmosuchus jakovlevi* (PIN 4332/1), *Tsylmosuchus samariensis* (PIN 2424/6), *Gamosaurus lozovskii* (PIN 3361/13), specimens referred to *Archosaurus rossicus* (PIN 1100/66a, 1100/66b), cf. *Chasmatosuchus* from Brazil [110] and *Chasmatosuchus rossicus* (PIN 3200/217), some specimens of *Proterosuchus fergusi* (e.g. BP/1/3993), and some erythrosuchids (*Garjainia prima*: [43]; *Erythrosuchus africanus*: SAM-PK-3028). By contrast, this fossa ventral to the postzygapophysis is absent in *Sarmatosuchus otschevi* (PIN 2865/68). The dorsal surface of the postzygapophysis of *Samsarasuchus pamelae* is posterodorsally expanded, forming a dorsally convex margin in lateral view in some specimens (e.g. ISIR 1080: Cv4, paratype; ISIR 1081: Cv5; ISIR 1084: Cv5), whereas this margin is not expanded and is straight in lateral view in others (e.g. GSI 2111: Cv3; PGRU/GL/M/VF-002: Cv3/4; GSI 2115: Cv5). In particular, specimens of Cv5 (GSI 2115, ISIR 1081, 1084; figure 4*g*,*h,j,l,m,n*) possess a distinct, but low, swelling dorsal to the postzygapophysis, which is interpreted here as an epipophysis as present in several other archosauromorphs [9,17,18,142]. Among non-eucrocopodan archosauriforms, similar epipophyses occur in *Tsylmosuchus samariensis* (PIN 2424/6) and specimens referred to *Archosaurus rossicus* (PIN 1100/66a, 1100/66b), ‘*Blomosuchus georgii*’ (PIN 1025/420), cf. *Chasmatosuchus* from Brazil [110], and *Chasmatosuchus rossicus* (PIN 3200/217), and more posterodorsally expanded epipophyses are present in *Proterosuchus fergusi* (e.g. BP/1/3993), *Proterosuchus alexanderi* (NMQR 1484) and *Jaikosuchus magnus* (PIN 951/65). The epipophyses of *Samsarasuchus pamelae* are positioned distinctly dorsal to the rim of the articular facet of the postzygapophysis, as in several non-archosauriform archosauromorphs (e.g. *Tanystropheus* sp.: SMNS 54654; *Boreopricea funerea*: PIN 3708/1) and the above-mentioned early archosauriforms to the exclusion of *Jaikosuchus magnus*. The epipophyses of this latter species are adjacent to the rim of the articular facet (PIN 951/65), as is the case in archosaurs [9]. Finally, the epipophyses of *Samsarasuchus pamelae* merge smoothly into the margin of the postzygapophysis dorsally and ventrally, resembling the condition in *Proterosuchus fergusi* (e.g. BP/1/3993), *Proterosuchus alexanderi* (NMQR 1484), *Tsylmosuchus samariensis* (PIN 2424/6), and specimens referred to *Archosaurus rossicus* (PIN 1100/66a, 1100/66b), ‘*Blomosuchus georgii*’ (PIN 1025/420), cf. *Chasmatosuchus* from Brazil [110], and *Chasmatosuchus rossicus* (PIN 3200/217). By contrast, the epipophyses have a distinct ventral notch, resulting in a free end, in *Boreopricea funerea* (PIN 3708/1), *Jaikosuchus magnus* (PIN 951/65) and several archosaurs [9].

The articular facet of the prezygapophysis is oval, being considerably longer anteroposteriorly than broad, and possesses an anterolateral main axis in dorsal view. The articular facet of the postzygapophysis is smaller and sub-circular in GSI 2111 (Cv3), but oval, with a transverse main axis, in GSI 2115 (Cv5), ISIR 1080 (Cv4, paratype) and ISIR 1084 (Cv5). Both zygapophyseal articular facets are slanted medially. There is a low and very thin ridge with an anteroposterior main axis, but with a slight posteroventral slant, that extends along the lateral surface of the neural arch and base of the prezygapophysis in all the specimens of *Samsarasuchus pamelae* in which this region is preserved (e.g. figures 3*b*,*j* and 4*a,b,h,i*). A very similar diagonal ridge is present in the anterior to middle cervical vertebrae of several non-eucrocopodan archosauriforms, such as *Jaikosuchus magnus* (PIN 951/65), *Tsylmosuchus jakovlevi* (PIN 4332/1), *Tsylmosuchus samariensis* (PIN 2424/6), *Garjainia prima* (PIN 951/64) and specimens referred to *Archosaurus rossicus* (PIN 1100/66a, 1100/66b), ‘*Blomosuchus georgii*’ (PIN 1025/420), cf. *Chasmatosuchus* from Brazil [110] and *Chasmatosuchus rossicus* (PIN 3200/217), the aphanosaurian archosaur *Teleocrater rhadinus* (NMT RB511), and the poposauroid archosaur *Xilousuchus sapingensis* (IVPP V6026) (figure 5: ri). In particular, a probably homologous, but considerably thicker ridge is present in *Gamosaurus lozovskii* (PIN 3361/13, figure 5*r*: tri). By contrast, a thin or thick ridge is absent in *Proterosuchus alexanderi* (NMQR 1484), *Proterosuchus fergusi* (SNSB-BSPG 1934 VIII 514; BP/1/3993, 4016; SAM-PK-K140), *Sarmatosuchus otschevi* (PIN 2865/68) and deeply nested erythrosuchids (e.g. *Erythrosuchus africanus*: SAM-PK 3028). In *Samsarasuchus pamelae*, this ridge reaches posteriorly a thick and rounded tuberosity that extends anteroventrally from the base of the postzygapohysis. The anterior end of this tuberosity is laterally inflated on both sides of the vertebra in GSI 2111 (Cv3), but not in other specimens (e.g. GSI 2115: Cv5, ISIR 1080: Cv4, paratype, 1081: Cv5). There is no hyposphene or hypantrum.

The prespinal fossa is moderately deep and restricted to the base of the neural spine (e.g. figures 3*c,k* and 4*c,j*), whereas the postspinal fossa is transversely broader and deeper (e.g. figures 3*d,l* and 4*d,k*). The latter fossa invades the posterior surface of the neural spine along its entire extension in ISIR 1080 (paratype), which is the only postaxial anterior cervical vertebra with a complete posterior margin of the neural spine (figure 4*a*). In this specimen, the postspinal fossa is delimited collaterally by a pair of thin, vertical ridges. By contrast, the postspinal fossa of GSI 2115 (Cv5) is not as dorsally developed as in ISIR 1080 (Cv4, paratype) and some other specimens (e.g. GSI 2111: Cv3). There is a very shallow depression immediately lateral to the base of the neural spine in several specimens (e.g. ISIR 1080: Cv4, paratype, ISIR 1084: Cv5; e.g. figure 4*a,b,m*), but it is absent in others (e.g. ISIR 1085: Cv4). A depression or fossa lateral to the base of the neural spine is widespread among most non-eucrocopodan archosauriforms [18].

The preserved postaxial anterior cervical neural spines of *Samsarasuchus pamelae* are 1.22–1.34 times taller than the heights of the respective centra, resembling the condition in *Proterosuchus fergusi* (1.23–1.34: BP/1/3993, Cv4, probable Cv5; 1.28: SAM-PK-11208, Cv5; 1.25: SNSB-BSPG 1934 VII 514, Cv3), *Proterosuchus alexanderi* (1.24–1.61: NMQR 1484, Cv3, Cv4) and *Garjainia prima* (1.21–1.40: PIN 2394/5-11–5-13, Cv3–5) (figure 5*a–d*). By contrast, the postaxial anterior cervical neural spines are proportionally taller in *Jaikosuchus magnus* (ratio = 1.64: PIN 951/65), *Tsylmosuchus jakovlevi* (1.79: PIN 4332/1), *Tsylmosuchus samariensis* (2.11: PIN 2424/6) and a specimen referred to *Chasmatosuchus rossicus* (1.79: PIN 3200/217) (figure 5*i–k,s*) and cf. *Chasmatosuchus* from Brazil (1.44: [110]), but proportionally shorter in *Sarmatosuchus otschevi* (0.96–0.98: PIN 2865/68, Cv3, Cv4). Although the postaxial anterior cervical neural spines of *Samsarasuchus pamelae* have a similar proportional height, there is considerable variation in the morphology of their anterior and posterior margins among specimens, as also occurs in this region of the axial skeleton in preserved specimens of *Proterosuchus fergusi* (e.g. SNSB-BSPG 1934 VIII 514, CGS GHG 231, SAM-PK-11208, BP/1/3993). The anterior margin of the neural spine of ISIR 1080 (paratype, Cv4) slants anteriorly from its base at an angle of 70° with respect to the axial plane, resulting in a distinct anterior overhang in lateral view (figure 4*a,b*). By contrast, the anterior projection of the neural spine is considerably weaker (e.g. ISIR 1084: Cv5) or absent (e.g. GSI 2111: Cv3 [at least at its base], PGRU/GL/M/VF-002: Cv3/4) in other specimens. The posterior margin of the neural spine is vertical as far as it is preserved in GSI 2111 (Cv3) and slants posteriorly in other specimens (e.g. ISIR 1080: paratype, Cv4, 1081: Cv5, 1084: Cv5, PGRU/GL/M/VF-002: Cv3/4; e.g. figure 4*a,b,g,h*). In particular, the posterior slant of the posterior margin of the neural spine is more pronounced in ISIR 1080 (paratype, Cv4; figure 4*a,b*) and particularly in PGRU/GL/M/VF-002 (Cv3/4) than in other specimens. As a result of the conspicuous slant of the anterior and posterior margins, the neural spines acquire a distinct fan-shaped profile in lateral view (e.g. ISIR 1080: paratype, Cv4, 1081: Cv5). However, PGRU/GL/M/VF-002 (Cv3/4) lacks an anterior overhang and the neural spine is not fan-shaped in lateral view. Thus, Cv4 and Cv5 of *Samsarasuchus pamelae* (the condition is unknown in Cv3) share with several early archosauriforms the presence of a posterior margin of the neural spine that slants at more than 15° with respect to the vertical plane, including the ‘Arcadia proterosuchian’ (based on an isolated neural spine very similar to those of the anterior cervical vertebrae of *Samsarasuchus pamelae*; QMF10125), *Jaikosuchus magnus* (PIN 951/65, figure 5*s*), *Tsylmosuchus jakovlevi* (PIN 4332/1, figure 5*j*), *Tsylmosuchus samariensis* (PIN 2424/6, figure 5*k*), a specimen referred to *Chasmatosuchus rossicus* (PIN 3200/217; figure 5*i*) and cf. *Chasmatosuchus* from Brazil [110], *Euparkeria capensis* (SAM-PK 5867), *Tropidosuchus romeri* (PVL 4601) and some archosaurs (e.g. *Xilousuchus sapingensis*: [143]; *Teleocrater rhadinus*: [144]; figure 5*t,v*). The morphology is different in Cv4–8 of *Proterosuchus fergusi* (e.g. BP/1/3993), *Proterosuchus alexanderi* (NMQR 1484), ‘*Chasmatosaurus*’ *yuani* (IVPP V4067), *Sarmatosuchus otschevi* [139] and *Garjainia prima* [140], in which the posterior margin of the neural spine is vertical or slants anterodorsally in lateral view (e.g. figure 5*c,d*).

The lateral surface of the neural spine possesses a thick, sub-rectangular longitudinal tuberosity in GSI 2111 (Cv3), which is absent in other specimens (e.g. GSI 2115: Cv5, ISIR 1080: paratype, Cv4, 1081: Cv5, 1084: Cv5, PGRU/GL/M/VF-002: Cv3/4). This tuberosity is placed immediately dorsal to the level of the dorsal margin of the postzygapophysis, becomes slightly dorsoventrally taller towards its anterior end, and has a rugose surface. This tuberosity possesses a similar shape and orientation on both sides of the neural spine, but it is slightly more ventrally positioned on the right side. This slight asymmetry and absence of the tuberosity in other cervical vertebrae of *Samsarasuchus pamelae* might indicate that it is a result of an abnormal bone outgrowth (exostosis) in this individual. The posterior surface of the base of the neural spine possesses a well developed, vertical ridge for attachment of interspinous ligaments in Cv5 specimens GSI 2115 and ISIR 1084. By contrast, this area of the neural spine is transversely concave in GSI 2111 (Cv3) and ISIR 1080 (paratype, Cv4). The distal margin of the neural spine possesses a poorly developed transverse expansion, but there is no evidence of distinct mammillary processes at least in ISIR 1080 (paratype, Cv4) and NHMUK PV R 37578 (Cv3/4). This condition resembles that present in *Jaikosuchus magnus* (PIN 951/65), *Tsylmosuchus jakovlevi* (PIN 4332/1), *Tsylmosuchus samariensis* (PIN 2424/6), the ‘Arcadia proterosuchian’ (QMF10125), the cf. *Chasmatosuchus* from Brazil [110], and some early poposauroids (*Xilousuchus sapingensis*, IVPP V6026) and aphanosaurs [104] (figure 5: dex). A specimen referred to *Chasmatosuchus rossicus* (PIN 3200/217) possesses a similar distal expansion, but it has distinct mammillary processes on the anterior region of the neural spine (figure 5*i*: mp). The presence of an expansion of the distal margin of the neural spine in *Samsarasuchus pamelaae* differs from the unexpanded spines of the anterior cervical vertebrae of *Proterosuchus alexanderi* (NMQR 1484) and *Proterosuchus fergusi* (BP/1/3993, SNSB-BSPG 1934 VIII 514) (figure 5*c,d*). The dorsal surface of the distal expansion of the neural spines of *Samsarasuchus pamelae* is flat to slightly transversely concave (e.g. ISIR 1080: paratype, Cv4) or slightly transversely convex (e.g. ISIR 1081: Cv5). In particular, the posterodistal corner of the neural spine possesses a short posterior expansion in ISIR 1081 (Cv5), which is absent in other specimens (e.g. ISIR 1080: paratype, Cv4, PGRU/GL/M/VF-002: Cv3/4).

##### 4.1.1.2. Cervical vertebra 6

This cervical position is represented by three specimens: GSI 2110, ISIR 1086 and NHMUK PV R37587 (figures 6*a–g* and 7*a–c* and table 3). GSI 2110 (Huxley [58]: plate I, figure 5, but figured reversed in the lateral view) preserves a complete centrum, but most of the neural arch is lost, with the exception of the right diapophysis, base of right prezygapophysis and the posterior half of the walls of the neural canal (figures 6*a–e* and 7*a*). The left diapophysis seems to have been lost after it was figured by Huxley [58]. The articular surfaces of the right parapophysis and diapophysis and the anteroventral margin of the centrum are damaged. ISIR 1086 is represented by a centrum, with damaged anterior and posterior margins, and the base of the neural arch (figure 7*b*,*c*). NHMUK PV R37587 preserves the right half of the centrum and base of the neural arch, but the distal end of the diapophysis is damaged (figure 6*f*,*g*). All these specimens possess a consistent morphology between each other and are interpreted to belong to the sixth cervical position because their morphology is similar to that of Cv4 and Cv5 but the diapophysis is proportionally longer anteroposteriorly and better projected laterally, and the lamina that projects posteriorly from the diapophysis has a stronger dorsal bowing, resembling the changes observed in the postaxial anterior cervical series of *Proterosuchus alexanderi* (NMQR 1484). This combination of features is also present in an unpublished archosauromorph vertebra from the Arcadia Formation (QMF60371), which is also interpreted here as a probable Cv6. The cervical ‘a’ of *Guchengosuchus shiguaiensis* (*sensu* Butler *et al.* [115]) is interpreted here as a Cv6/7 because the parapophysis is on the anteroventral corner of the centrum and the diapophysis is on the centrum-neural arch transition and close to the anterior margin of the vertebra, as in Cv6 of *Garjainia prima* and Cv6/7 of proterosuchids. As a result, the probable Cv6 of the ‘Arcadia proterosuchian’ and cervical ‘a’ of *Guchengosuchus shiguaiensis* are also compared with Cv6 of *Samsarasuchus pamelae*.

The centrum is 1.61 times longer than the height of its posterior articular surface in GSI 2110 and 1.56 times the height of its anterior articular surface in NHMUK PV R37587; thus, slightly proportionally shorter than more anterior postaxial centra. A probable Cv6 referred to *Chasmatosuchus rossicus* (PIN 3200/472; figure 7*k*,*n*) has a similar ratio (but a precise value cannot be calculated because the anterior and posterior rims of the centrum are damaged) and also resembles the ratio present in cervical ‘a’ (*sensu* Butler *et al.* [115]) of *Guchengosuchus shiguaiensis* (*ca* 1.5; Butler *et al.* [115]: figure 13). By contrast, a proportionally shorter Cv6 is present in *Proterosuchus alexanderi* (length-posterior height ratio = 1.25: NMQR 1484; figure 7*l*), *Proterosuchus fergusi* (1.16: CGS GHG 231; 1.10: SAM-PK 11208; figure 7*i*), a probable Cv6 of the ‘Arcadia proterosuchian’ (1.26: QMF60371; figure 7*d*) and more conspicuously in *Sarmatosuchus otschevi* (0.87: PIN 2865/68), *Garjainia prima* (1.05: PIN 951/64-6), and *Bharitalasuchus tapani* (0.73: [102]). The centrum is moderately transversely compressed at mid-length in ventral view. The centrum is a parallelogram in lateral view, with the anterior articular surface being situated slightly dorsal to the posterior one, as in other early archosauriforms [18]. The anterior and posterior articular facets of the centrum are concave and sub-circular. There is no distinct notochordal pit on the anterior surface of the centrum; the posterior surface of the centrum is partially preserved or covered with matrix in available specimens. Nevertheless, the deepest area of the anterior articular surface is slightly displaced dorsally from the centre of the facet, resembling the position of the notochordal pit of more anterior cervical centra (GSI 2111, 2115). The centrum possesses a sharp, low median longitudinal keel that extends along its entire ventral surface (figures 6*g* and 7*c*: vk), contrasting with the more restricted ventral keel of more anterior cervical elements. A similar ventral keel is present in Cv6 of *Proterosuchus fergusi* (CGS GHG 231, SAM-PK-11208; figure 7*j*: vk), *Sarmatosuchus otschevi* (PIN 2865/68), *Garjainia prima* [140], *Bharitalasuchus tapani* [102], the ‘Arcadia proterosuchian’ (QMF60371; figure 7*h*: vk), *Guchengosuchus shiguaiensis* (cervical ‘a’ *sensu* Butler *et al.* [115]), and the specimen referred to *Chasmatosuchus rossicus* (PIN 3200/472; figure 7*n*: vk), but Cv6 of *Proterosuchus alexanderi* lacks a ventral keel (NMQR 1484; figure 7*m*). The collateral ridges and depressions present in the posterior third of the centrum of some Cv3–Cv5 of *Samsarasuchus pamelae* (GSI 2111, 2115) is absent in the three preserved Cv6.

The parapophysis is positioned on the anteroventral corner of the centrum and lateroventrally projected on a moderately well-developed peduncle, which extends laterally slightly beyond the level of the lateral margin of the centrum. The articular facet of the parapophysis is sub-oval, slightly anteriorly facing and possesses a smooth, shallowly concave surface in GSI 2110 and a gently convex one in NHMUK PV R37587. The main axis of the parapophyseal articular facet is anteroventrally-to-posterodorsally oriented. The diapophysis is restricted to the anterior half of the centrum and lateroventrally projected, being considerably more laterally extended than the parapophysis (figure 6*c*), resembling the condition of Cv6 of *Proterosuchus alexanderi* (NMQR 1484), the ‘Arcadia proterosuchian’ (QMF60371; figure 7*e*), *Bharitalasuchus tapani* [102], and the specimen referred to *Chasmatosuchus rossicus* (PIN 3200/472). The diapophysis is sub-oval in cross-section, with an anteroposteriorly oriented main axis. There is a pair of low, straight ridges/tuberosities that extend posteroventrally and posterodorsally, respectively, from the base of the diapophysis in GSI 2110 (figure 6*a*: pdri, wlt). The posterodorsally oriented ridge resembles the postzygodiapophyseal lamina of cervical ‘a’ of *Guchengosuchus shiguaiensis* [115], and they are probably homologous structures. By contrast, this posterodorsal ridge is absent in NHMUK PV R37587 and ISIR 1086, resembling the condition in Cv6 of *Proterosuchus alexanderi* (NMQR 1484), the ‘Arcadia proterosuchian’ (QMF60371), *Sarmatosuchus otschevi* (PIN 2865/68), *Bharitalasuchus tapani* [102] and the specimen referred to as *Chasmatosuchus rossicus* (PIN 3200/472). The posteroventrally oriented tuberosity is strongly laterally developed and extends through the lateral surface of the centrum as a wing-like shelf (figure 6*a*–*g*: wlt), as occurs in Cv4 and Cv5 of *Samsarasuchus pamelae* and the probable Cv6 of the ‘Arcadia proterosuchian’ (QMF60371; figure 7*h*: wlt) and the specimen referred to as *Chasmatosuchus rossicus* (PIN 3200/472; figure 7*n*: wlt). This strongly laterally developed posteroventral tuberosity is absent in Cv6 of *Proterosuchus alexanderi* (NMQR 1484; figure 7*m*), *Proterosuchus fergusi* (CGS GHG 231, SAM-PK-11208; figure 7*j*), *Sarmatosuchus otschevi* (PIN 2865/68), *Guchengosuchus shiguaiensis* (cervical ‘a’; [115]), *Garjainia prima* [140] and *Bharitalasuchus tapani* [102]. The posteroventral tuberosity reaches the posterior third of the centrum and defines the dorsal margin of a shallow and not distinctly rimmed lateral fossa on the centrum. There is a small, moderately deep and lateroventrally facing fossa that is defined ventrally by the base of the posteroventrally oriented tuberosity and anteriorly by the diapophysis (figure 6*a*,*f*: fo). The posterodorsally oriented ridge of GSI 2110 extends a short distance along the base of the neural arch. The neurocentral suture is closed in the three specimens. The base of the prezygapophysis is anterodorsally oriented and its preserved lateral surface is shallowly concave. The presence of a thin lateral ridge on the lateral surface of the neural arch cannot be determined because of breakage, but it is present in Cv6 of the ‘Arcadia proterosuchian’ (QMF60371; figure 7*d*: ri) and the specimen referred to as *Chasmatosuchus rossicus* (PIN 3200/472; figure 7*k*: ri).

##### 4.1.1.3. Cervical vertebra 7

There are four specimens that are interpreted as Cv7: NHMUK PV R37580 and ISIR 1087–1089 (figure 6*h*–*p*; table 4). NHMUK PV R37580 is a fairly complete vertebra, but lacks the left postzygapophysis and the anterior and posterior rims of the centrum are slightly damaged (figure 6*h*–*m*). ISIR 1087 lacks most of the centrum and left prezygapophysis, the right zygapophyses, the distal tip of the left postzygapophysis and left lateral margin and posterior part of the distal end of the neural spine (figure 6*n*–*p*). ISIR 1089 lacks the ventral and left lateral margins of the anterior end of the centrum, the left parapophysis, part of the right parapophysis, the right prezygapophysis, the distal end of the left prezygapophysis, both postzygapophyses, and most of the left transverse process and neural spine. Finally, ISIR 1088 lacks the posterior half of the centrum, most of the parapophyses, diapophyses and neural spine, and both prezygapophyses and right postzygapophysis.

These vertebrae possess a lateroventrally directed diapophysis, the dorsal margin of which is positioned slightly dorsal to the level of the floor of the neural canal, as occurs in Cv6. This condition is intermediate between the lower diapophyses of the inferred Cv3–5 and the more dorsally positioned diapophyses of the very posterior cervical and anterior dorsal vertebrae of *Samsarasuchus pamelae*. In addition, the diapophysis is still close to the anterior margin of the centrum, and there is a thin ridge on the lateral surface of the neural arch, resembling Cv3–6, but contrasting with very posterior cervical and dorsal vertebrae. There is a small, but deep, fossa positioned posteroventrally to the transverse process, which is absent in more anterior cervical vertebrae, but resembles the centrodiapophyseal fossa of dorsal vertebrae. As a result of this combination of features, these vertebrae are interpreted to belong to the seventh cervical position.

The centrum is approximately 1.3–1.5 times longer than the height of its anterior articular surface in NHMUK PV R37580 (figure 6*h*,*i*), and 1.46 times longer than the height of its posterior articular surface in ISIR 1089, resembling the ratio present in the probable Cv6/7 of *Guchengosuchus shiguaiensis* (*ca* 1.5; Butler *et al.* [115]). A slightly lower length to anterior height ratio of the seventh cervical centrum is present in *Proterosuchus alexanderi* (1.26; NMQR 1484) and the ratio is considerably lower in *Sarmatosuchus otschevi* (0.87: PIN 2865/68), *Garjainia prima* (0.90: PIN 951/64-7) and *Bharitalasuchus tapani* (0.62: [102]). The centrum is moderately transversely compressed at mid-length and spool-shaped in ventral view. The centrum is gently parallelogram-shaped in lateral view, with the anterior articular surface being situated slightly dorsal to the posterior one, as in more anterior cervical vertebrae. The anterior and posterior articular facets of the centrum are shallowly concave and sub-circular. The ventral surface of the centrum possesses a very thin and low median longitudinal keel that extends along most of the length of the centrum, as in the seventh cervical centrum of *Proterosuchus alexanderi* (NMQR 1484), *Bharitalasuchus tapani* [102] and cervical ‘a’ of *Guchengosuchus shiguaiensis* [115]. By contrast, Cv7 of *Proterosuchus fergusi* (SAM-PK-11208) and *Sarmatosuchus otschevi* (PIN 2865/68) lacks a ventral keel on the centrum. The parapophysis is situated on the anteroventral corner of the centrum and its articular facet faces laterally. The parapophysis is low and extends laterally at the same level as the external rim of the anterior articular facet of the centrum. The lateral surface of the centrum is flat, lacking a lateral fossa. There are multiple, probably nutrient, foramina piercing the lateral surface of the centrum, which are mainly situated immediately posterodorsally to the parapophysis and close to the posterior margin of the centrum in ISIR 1089. The neurocentral suture is closed in all specimens.

There are no laminae connecting the diapophysis with the zygapophyses, as in *Proterosuchus fergusi* (SAM-PK-11208), *Proterosuchus alexanderi* (NMQR 1484) and *Sarmatosuchus otschevi* (PIN 2865/68), but contrasting with the presence of prezygodiapophyseal and postzygodiapophyseal laminae in Cv7 of *Garjainia prima* (PIN 951/64-7) and cervical ‘a’ of *Guchengosuchus shiguaiensis* [115]. The posterior end of the base of the diapophysis merges with a tuberosity that runs along the posteroventral corner of the neural arch (figure 6*h*,*l*: wlt). Both structures meet in a slightly acute angle in lateral view and roof a moderately deep, lateroventrally facing blind fossa. There is no articular facet for the reception of a third articular rib head, contrasting with the presence of such a structure in Cv7 of at least one specimen of *Proterosuchus fergusi* (SAM-PK-11208) and probably the Cv7 of *Garjainia prima* [140].

The prezygapophysis is anterodorsally directed and extends substantially anteriorly beyond the anterior margin of the centrum. The articular facet of the prezygapophysis slants medially and is oval, with an anteroposterior main axis. The lateral surface of the prezygapophysis possesses an anterodorsally-to-posteroventrally oriented, very thin ridge that extends posteriorly reaching close to the mid-length of the neural arch. This ridge seems to be a more subtle condition of the ridge present in the same position in more anterior cervical vertebrae (although it is also poorly developed in Cv5 specimen ISIR 1085). The postzygapophysis is mainly posteriorly directed, but with a small lateral component. The articular facet of the postzygapophysis faces lateroventrally and is oval, with an anteroposterior main axis. The base of the postzygapophysis is invaded by a moderately deep, well-defined and posterolaterally facing fossa. This fossa is oval, with an anteroventrally-to-posterodorsally oriented main axis. The vertebra lacks the hyposphene and epipophysis, as in Cv7 of *Sarmatosuchus otschevi* (PIN 2865/68) and *Garjainia prima* (PIN 951/64-7) but contrasting with the presence of a hyposphene in cervical ‘a’ of *Guchengosuchus shiguaiensis* [115].

The prespinal fossa is moderately deep and invades the base of the neural spine, resulting in an anteroposteriorly deep horizontal shelf between the prezygapophyses (figure 6*j*: prsf). The prespinal fossa extends dorsally slightly dorsal to the level of the base of the prezygapophysis and is delimited laterally by very thin edges of the anterolateral margins of the neural spine. Similarly, a moderately deep postspinal fossa invades the base of the neural spine between the postzygapophyses (figure 6*m*: posf). There is a moderately deep, circular fossa immediately lateral to the base of the neural spine and posteriorly to the base of the prezygapophysis (figure 6*i*: fo), resembling the condition in *Proterosuchus fergusi* (SAM-PK-11208) and cervical ‘a’ of *Guchengosuchus shiguaiensis* [115], but contrasting with the absence of this fossa in *Sarmatosuchus otschevi* (PIN 2865/68). This fossa faces mainly dorsally and is not internally subdivided. The neural spine is approximately 1.7 times higher than anteroposteriorly long at its base in ISIR 1087 and NHMUK PV R37580, which is a ratio considerably lower than in Cv7 of *Proterosuchus alexanderi* (2.27: NMQR 1484), *Proterosuchus fergusi* (2.13: SAM-PK-11208), *Sarmatosuchus otschevi* (2.39: PIN 2865/68) and *Garjainia prima* (2.52: PIN 951/64-7). The neural spine of Cv7 of *Samsarasuchus pamelae* is mainly vertically oriented, as in most early archosauriforms (e.g. *Proterosuchus alexanderi*: NMQR 1484; some specimens of *Proterosuchus fergusi*: SAM-PK-11208; *Sarmatosuchus otschevi*: [139]; *Garjainia prima*: [140]), but in Cv7 of some specimens of *Proterosuchus fergusi* (CGS GHG 231) and cervical ‘a’ of *Guchengosuchus shiguaiensis* [115] the neural spine is anterodorsally oriented. The neural spine of *Samsarasuchus pamelae* becomes gradually anteroposteriorly longer towards its dorsal margin as a result of its straight and divergent anterior and posterior margins. This condition is similar to that in cervical ‘a’ of *Guchengosuchus shiguaiensis* [115], but the gradual anteroposterior expansion of the neural spine in *Guchengosuchus shiguaiensis* is a result of an anteriorly curved anterior margin of the spine. The neural spine of Cv7 of other early archosauriforms has mainly straight and rather sub-parallel anterior and posterior margins in lateral view (e.g. *Proterosuchus alexanderi*: NMQR 1484; *Proterosuchus fergusi*: SAM-PK-11208; *Sarmatosuchus otschevi*: [139]; *Garjainia prima*: [140]). The distal end of the neural spine possesses a distinct anterior prong, which produces a sharp inflexion along the anterior margin of the spine, in ISIR 1087 (figure 6*n*,*p*: apr), which seems to be absent in NHMUK PV R37580. The presence of a similar projection on the posterodorsal corner of the neural spine cannot be determined because of damage. A low, rounded mammillary process is present and restricted to the dorsal margin and anterior half of the neural spine (figure 6*h*–*j*,*n*: mp), resembling the condition in Cv7 of *Proterosuchus alexanderi* (NMQR 1484), ‘*Chasmatosaurus*’ *yuani* (IVPP V4067) and some isolated neural spines of the ‘Arcadia proterosuchian’ (QMF10125). The neural spine of Cv7 of *Garjainia prima* (PIN 951/64-7) also has a mammillary process, but this is positioned at the anteroposterior mid-length of the spine. By contrast, the middle cervical vertebrae of *Sarmatosuchus otschevi* (PIN 2865/68) and *Guchengosuchus shiguaiensis* [115] lack mammillary processes. The dorsal margin of the neural spine is slightly transversely expanded posterior to the mammillary process in ISIR 1087 but not in NHMUK PV R37580. The dorsal surface of the neural spine is slightly transversely convex.

##### 4.1.1.4. Cervical vertebra 8

A fairly complete vertebra, ISIR 1090 (figure 8 and table 4), is interpreted as from the eighth cervical position because the parapophysis is positioned on the anteroventral corner of the centrum, the diapophysis is placed level with the floor of the neural canal and posteriorly displaced from the anterior margin of the neural arch, there is a short anterior centrodiapophyseal lamina, and the mammillary processes are well developed and distinctly separated from the anterior margin of the neural spine. The position and development of the rib articular processes closely resemble those of Cv8 of *Proterosuchus alexanderi* (NMQR 1484). Similarly, this combination of parapophyseal and diapophyseal features occurs in the anteriormost preserved vertebrae of the holotype of *Cuyosuchus huenei* (MCNAM PV 2669) and the holotype of *Chasmatosuchus rossicus* (PIN 2252/381; figure 9*j*,*k*), a specimen referred to as *Gamosaurus lozovskii* (PIN 3361/14; [40]; figure 9*b*,*d*) and the ‘Arcadia proterosuchian’ (QMF9533; Thulborn [111]: plate 3K–M; figure 9*f*–*h*). Thus, these three vertebrae are here interpreted as probable Cv8. Cervical ‘b’ of *Guchengosuchus shiguaiensis* (*sensu* Butler *et al.* [115]) probably represents Cv7/8 because of the shape of its rib articular processes and is probably the element that followed cervical ‘a’ in the cervical series, the latter having been interpreted here as Cv6/7. Finally, Gower ([141]: figure 22*a* and table 1) interpreted an anterior ‘pectoral’ vertebra of NHMUK PV R3592 (figure 9*c*), a specimen of *Erythrosuchus africanus*, as a possible Cv8 and the morphology of the following vertebra (see description of Cv9) in the series supports this interpretation. Therefore, ISIR 1090 is compared with the above-mentioned specimens.

The centrum is 1.15 times longer than the height of its anterior articular surface, being proportionally longer than in Cv8 of *Proterosuchus fergusi* (0.97: SAM-PK-11208), *Sarmatosuchus otschevi* (0.76: PIN 2865/68), *Garjainia prima* (0.82: PIN 951/64-8) and substantially longer than in *Erythrosuchus africanus* (0.47: Gower [141]: table 1) and *Bharitalasuchus tapani* (0.58: [102]). By contrast, the probable Cv8 of *Cuyosuchus huenei* (1.26: MCNAM PV 2669), the ‘Arcadia proterosuchian’ (1.40: QMF9533), the holotype of *Chasmatosuchus rossicus* (1.44: PIN 2252/381), the specimen referred to as *Gamosaurus lozovskii* (1.67: PIN 3361/14) and cervical ‘b’ of *Guchengosuchus shiguaiensis* (1.37: IVPP V8808) are proportionally longer. The centrum is moderately transversely compressed at mid-length and spool-shaped in ventral view. The centrum is slightly parallelogram in lateral view, with the anterior articular surface being situated slightly dorsal to the posterior one. The anterior and posterior articular facets of the centrum are shallowly concave and sub-circular. The ventral surface of the centrum possesses a very thin and low median longitudinal keel that extends along almost the entire length of the centrum (figure 8*g*: vk), resembling the condition in Cv8 of *Proterosuchus alexanderi* (NMQR 1484), *Garjainia prima* (PIN 951/64-8), *Bharitalasuchus tapani* [102], cervical ‘b’ of *Guchengosuchus shiguaiensis* [115] and probable Cv8 of the holotype of *Chasmatosuchus rossicus* (PIN 2252/381; figure 9*j*), the ‘Arcadia proterosuchian’ (QMF9533; figure 9*h*), and the specimen referred to as *Gamosaurus lozovskii* (PIN 3361/14; figure 9*d*). By contrast, *Proterosuchus fergusi* (SAM-PK-11208) and *Sarmatosuchus otschevi* (PIN 2865/68) lack a ventral keel on their eighth cervical centra. The parapophysis is positioned on the anteroventral corner of the centrum and its articular facet is lateroventrally facing. The parapophysis is low and extends laterally at the same level as the anterior articular facet of the centrum. The lateral surface of the centrum possesses a moderately deep, lateral fossa lacking in any pronounced rim (figure 8*a*,*b*: fo). There is only one small probable nutrient foramen piercing the fossa on the left side of the centrum. The neurocentral suture is closed.

The neural arch possesses very short anterior centrodiapophyseal and prezygodiapophyseal laminae, and a long and thick postzygodiapophyseal lamina (figure 8: acdl, podl, prdl), as in Cv8 of *Sarmatosuchus otschevi* (PIN 2865/68) and *Garjainia prima* [140], the probable Cv8 of *Chasmatosuchus rossicus* (PIN 2252/381), the specimen referred to as *Gamosaurus lozovskii* (PIN 3361/14), and *Cuyosuchus huenei* (MCNAM PV 2669) and cervical ‘b’ of *Guchengosuchus shiguaiensis* [115]. The posterior centrodiapophyseal lamina is absent, as in *Sarmatosuchus otschevi* (PIN 2865/68), but this structure is present in *Garjainia prima* [140], the probable Cv8 of *Chasmatosuchus rossicus* (PIN 2252/381), the ‘Arcadia proterosuchian’ (QMF9533), the specimen referred to as *Gamosaurus lozovskii* (PIN 3361/14), and *Cuyosuchus huenei* (MCNAM PV 2669) and cervical ‘b’ of *Guchengosuchus shiguaiensis* [115] (figure 9*f*,*k*: pcdl). Cv8 of *Proterosuchus alexanderi* (NMQR 1484) has only an anterior centrodiapophyseal lamina and the probable eighth neural arch of *Erythrosuchus africanus* lacks prezygodiapophyseal and postzygodiapophyseal laminae (NHMUK PV R3592). The diapophysis is lateroventrally oriented and its distal end is missing on both sides of the vertebra. The anterior centrodiapophyseal lamina is anteroventrally oriented and ends at the anterodorsal corner of the centrum, being separated from the parapophysis by a dorsoventrally concave surface. There is no third facet for rib articulation, as in the probable Cv8 of *Cuyosuchus huenei* (MCNAM PV 2669), but contrasting with the presence of this structure in the probable Cv8 of *Chasmatosuchus rossicus* (PIN 2252/381), the ‘Arcadia proterosuchian’ (QMF9533), cervical ‘b’ of *Guchengosuchus shiguaiensis* [115], *Garjainia prima* [140] and probably *Proterosuchus alexanderi* (NMQR 1484) (figure 9*f*,*j*: 3rfa). The presence of an accessory rib facet cannot be determined in Cv8 of *Sarmatosuchus otschevi* (PIN 2865/68) or the probable Cv8 of the specimen referred to as *Gamosaurus lozovskii* (PIN 3361/14) because of damage.

The prezygapophyseal centrodiapophyseal fossa (*sensu* Wilson *et al.* [145]) is shallow. The posterior end of the base of the diapophysis merges with a short and thick, posteroventrally oriented tuberosity. Both structures meet in a gently acute angle in lateral view and roof a moderately deep, lateroventrally facing blind fossa that invades the base of the transverse process, as occurs in Cv7. A posterodorsally-to-anteroventrally oriented tuberosity is present immediately ventral to this fossa and is probably positioned at or close to the level of the neurocentral suture (figure 8*a*: tu), as occurs in more posterior vertebrae (see below) and resembling the condition in some other early diverging archosauriforms (e.g. the specimen referred to as *Gamosaurus lozovskii*: PIN 3361/14; *Garjainia prima*: [43]). The prezygapophysis is anterodorsally oriented and extends anteriorly beyond the level of the anterior margin of the centrum. The lateral surface of the prezygapophysis possesses a subtle anterodorsally-to-posteroventrally oriented, very thin ridge that extends posteriorly close to the mid-length of the base of the neural arch (figure 8*a*: ri), as also occurs in Cv3–7 of *Samsarasuchus pamelae* and the probable Cv8 of *Chasmatosuchus rossicus* (PIN 2252/381), *Erythrosuchus africanus* (NHMUK PV R3592), and the specimen referred to as *Gamosaurus lozovskii* (PIN 3361/14) (figure 9*b*,*c*,*k*: ri). By contrast, this ridge is absent in Cv8 of *Proterosuchus alexanderi* (NMQR 1484), *Sarmatosuchus otschevi* (PIN 2865/68), and *Garjainia prima* (PIN 951/64-8) and cervical ‘b’ of *Guchengosuchus shiguaiensis* [115]. The ridge defines dorsally a moderately deep subtriangular fossa in ISIR 1090, with a ventral apex, positioned at the base of the transverse process. The postzygapophysis extends posteriorly slightly beyond the level of the posterior margin of the centrum and there is a very shallow lateral fossa immediately ventral to its base (figure 8*a*: fo). The articular facets of both zygapophyses slant medially. There is no hyposphene-hypantrum or epipophysis, similar to the condition in the specimen referred to as *Gamosaurus lozovskii* (PIN 3361/14), *Garjainia prima* (PIN 951/64-8), and *Erythrosuchus africanus* (NHMUK PV R3592) (figure 9*b*,*c*), but contrasting with the presence of both hyposphene and epipophysis in cervical ‘b’ of *Guchengosuchus shiguaiensis* ([115]; IVPP V8808) and the presence of epipophyses in the probable Cv8 of *Chasmatosuchus rossicus* (PIN 2252/381; figure 9*k*: ep).

The prespinal fossa is moderately deep and restricted to the base of the neural spine. The postspinal fossa is transversely broad, deep at its base and invades the ventral three-quarters of the neural spine. There is no depression immediately lateral to the base of the neural spine, as in *Sarmatosuchus otschevi* (PIN 2865/68), but a fossa is present in this region in Cv8 of *Proterosuchus fergusi* (SAM-PK-11208) and *Proterosuchus alexanderi* (NMQR 1484), cervical ‘b’ of *Guchengosuchus shiguaiensis* [115] and the probable Cv8 of *Chasmatosuchus rossicus* (PIN 2252/381) and the specimen referred to as *Gamosaurus lozovskii* (PIN 3361/14) (figure 9*a*,*b*,*k*: fo). The neural spine of Cv8 of *Samsarasuchus pamelae* is vertical and approximately two times higher than anteroposteriorly long at its base. By contrast, the eighth cervical neural spines of *Proterosuchus alexanderi* (2.40: NMQR 1484), *Sarmatosuchus otschevi* (2.39: PIN 2865/68), *Garjainia prima* (3.42: PIN 951/64-8), *Erythrosuchus africanus* (*ca* 3.5: NHMUK PV R3592), and cervical ‘b’ of *Guchengosuchus shiguaiensis* (2.59: IVPP V8808) are proportionally taller (figure 9*a*,*c*). The neural spine expands anteroposteriorly towards its dorsal margin as a result of slightly divergent and concave anterior and posterior margins, resembling the condition in cervical ‘b’ of *Guchengosuchus shiguaiensis* [115]. The degree of anteroposterior expansion of the eighth neural spine of *Samsarasuchus pamelae* is lower than that of the more anterior cervical vertebrae of the species. The lateral surface of the neural spine is shallowly dorsoventrally concave as a result of a gradual transverse expansion of the spine. Both anterior and posterior surfaces of the neural spine possess a low, vertical and thick ridge that may have been the attachment area of interspinous ligaments (figure 8*c*,*d*: ri). The dorsal end of the neural spine possesses a pair of laterally well-developed and rounded mammillary processes, as occurs in Cv8 of *Proterosuchus fergusi* (SAM-PK-11208), *Proterosuchus alexanderi* (NMQR 1484; figure 9*a*: mp), ‘*Chasmatosaurus*’ *yuani* (IVPP V4067), *Garjainia prima* [140] and some isolated neural spines of the ‘Arcadia proterosuchian’ (QMF10125). Mammillary processes are absent in Cv8 of *Sarmatosuchus otschevi* (PIN 2865/68), the probable Cv8 of *Erythrosuchus africanus* (NMHUK PV R3592; figure 9*c*), and cervical ‘b’ of *Guchengosuchus shiguaiensis* [115]. The mammillary processes of Cv8 of *Samsarasuchus pamelae* are situated on the anterior two-thirds of the neural spine, not reaching its anterior margin, as in Cv8 of *Proterosuchus alexanderi* (NMQR 1484) and some isolated neural spines of the ‘Arcadia proterosuchian’ (QMF10125). By contrast, the mammillary processes of Cv8 of *Garjainia prima* are positioned at the anteroposterior mid-length of the neural spine (PIN 951/64-8). The mammillary processes are confluent with the distal margin of the neural spine at its anterior margin, but posteriorly they extend posteroventrally and are situated slightly ventral to the distal margin. The posterior end of the neural spine is transversely thick and squared in dorsal view, resembling the condition in cervical ‘b’ of *Guchengosuchus shiguaiensis* [115] and Cv8 of *Sarmatosuchus otschevi* (PIN 2865/68), whereas this margin tapers posteriorly in Cv8 of *Garjainia prima* (PIN 951/64-8) and is transversely concave posteriorly in *Erythrosuchus africanus* (NMHUK PV R3592). The dorsal surface of the neural spine is mostly flat, as in *Proterosuchus alexanderi* (NMQR 1484), *Sarmatosuchus otschevi* (PIN 2865/68), *Garjainia prima* (PIN 951/64-8), and *Erythrosuchus africanus* (NMHUK PV R3592), but the neural spine of cervical ‘b’ of *Guchengosuchus shiguaiensis* [115] has a strongly transversely convex distal surface.

##### 4.1.1.5. Cervical vertebra 9

A fairly complete vertebra (ISIR 1091, holotype; figure 10 and table 4) is interpreted to belong to the ninth cervical position because the parapophysis is still positioned on the anteroventral corner of the centrum, the diapophysis is placed dorsal to the level of the floor of the neural canal and posteriorly displaced from the anterior margin of the neural arch, and the anterior centrodiapophyseal lamina broadens ventrally and very likely harbours an accessory rib articular facet. This combination of features is present in Cv9 of *Proterosuchus alexanderi* (NMQR 1484) and *Sarmatosuchus otschevi* (PIN 2865/68). By contrast, the parapophysis of the tenth presacral vertebrae of *Sarmatosuchus otschevi* (PIN 2865/68) and *Garjainia prima* (PIN 951/64-9) is not adjacent to the anteroventral corner of the centrum, but more dorsally placed. The holotype of *Vonhuenia friedrichi* (PIN 1025/11; figure 9*e*,*i*) is interpreted as a probable Cv9 because the parapophysis is still adjacent to the anteroventral corner of the centrum and the diapophysis is positioned dorsal to the level of the floor of the neural canal and mainly laterally oriented. This combination of features also occurs in a vertebra of *Erythrosuchus africanus* (NHMUK PV R3592) that was interpreted as a posterior ‘pectoral’ or a possible ninth presacral vertebra by Gower ([141]: figure 22*b,c* and table 1). A vertebra from the Arcadia Formation (QMF9548) has a diapophysis slightly more dorsally positioned than the vertebra from the same unit interpreted as a probable Cv8 (QMF9533) and, as a result, is interpreted as a probable Cv9. The posteriormost preserved vertebra of the holotype of *Chasmatosuchus rossicus* (PIN 2252/381; figure 9*j*,*k*) is interpreted as a ninth presacral element (see above). Thus, these specimens are also compared with Cv9 of *Samsarasuchus pamelae*.

The centrum is 1.35 times longer than the height of its anterior articular surface, resembling the ratio present in the probable Cv9 of *Chasmatosuchus rossicus* (1.32: PIN 2252/381), whereas the centrum is slightly anteroposteriorly shorter in *Vonhuenia friedrichi* (1.16: PIN 1025/11) and the probable Cv9 of the ‘Arcadia proterosuchian’ (1.21: QMF9548), and more conspicuously shorter in *Garjainia prima* (1.00: PIN 951/64-9), *Sarmatosuchus otschevi* (0.72: PIN 2865/68) and *Erythrosuchus africanus* (0.43: Gower [141]: table 1). The centrum is moderately transversely compressed at mid-length and spool-shaped in ventral view. The centrum is slightly parallelogram-shaped in lateral view, with the anterior articular surface being situated dorsal to the posterior one, closely resembling the morphology in Cv8. The anterior and posterior articular facets of the centrum are shallowly concave and sub-circular. The ventral surface of the centrum possesses a very thin and low median longitudinal keel that extends along the anterior two-thirds of the centrum (figure 10*f*: vk); the posterior third of the ventral surface of the centrum is continuously transversely convex. The development of the ventral keel in ISIR 1091 (holotype) resembles the condition in the probable Cv9 of *Chasmatosuchus rossicus* (PIN 2252/381; figure 9*j*: vk), *Vonhuenia friedrichi* (PIN 1025/11; figure 9*i*: vk), the ‘Arcadia proterosuchian’ (QMF9548) and *Erythrosuchus africanus* (Gower [141]: figure 22*b*). By contrast, the ninth cervical centrum of *Proterosuchus fergusi* (SAM-PK-11208), *Proterosuchus alexanderi* (NMQR 1484), *Garjainia prima* (PIN 951/64-9) and *Bharitalasuchus tapani* [102] lacks a ventral keel. The parapophysis is positioned on the anteroventral corner of the centrum and its articular facet faces laterally. The parapophysis is low and extends laterally to the same point as the anterior articular facet of the centrum. The lateral surface of the centrum is flat, lacking a lateral fossa. There are multiple probable nutrient foramina piercing the lateral surface of the centrum, which are mainly clustered immediately posterodorsal to the parapophysis and close to the posterior margin of the centrum. The neurocentral suture is closed, but there are still traces of the suture on the posterior half of the vertebra.

The neural arch possesses anterior centrodiapophyseal, prezygodiapophyseal and postzygodiapophyseal laminae (figure 10*a*: acdl, prdl, podl), but not a posterior centrodiapophyseal lamina, as in *Vonhuenia friedrichi* (PIN 1025/11) and *Sarmatosuchus otschevi* (PIN 2865/68). The posterior centrodiapophyseal lamina is also absent in *Proterosuchus alexanderi* (NMQR 1484). By contrast, these four vertebral laminae are present in Cv9 of *Garjainia prima* (PIN 951/64-9) and *Chasmatosuchus rossicus* (PIN 2252/381; figure 9*k*), and at least the anterior and posterior centrodiapophyseal laminae are present in the probable Cv9 of the ‘Arcadia proterosuchian’ (QMF9548) and *Bharitalasuchus tapani* [102]. The diapophysis is lateroventrally oriented and its distal end is missing on both sides of the vertebra; thus, it cannot be determined if the diapophysis was as long as those of, for example, *Vonhuenia friedrichi* and *Garjainia prima*. The anterior centrodiapophyseal lamina is anteroventrally oriented and ends at the anterodorsal corner of the centrum, being separated from the parapophysis by a dorsoventrally concave, broad surface (figure 10*a*: acdl). The distal end of the anterior centrodiapophyseal lamina is anteroposteriorly expanded, as occurs in the posterior cervical vertebrae with an accessory articular rib facet of *Prolacerta broomi* (BP/1/2675) and several early archosauriforms (e.g. *Proterosuchus alexanderi*: NMQR 1484; *Sarmatosuchus otschevi*: PIN 2865/68; *Chasmatosuchus rossicus*: PIN 2252/381; *Vonhuenia friedrichi*: PIN 1025/11; *Erythrosuchus africanus*: [141]; *Garjainia prima*: [140]; figure 9: 3rfa). As a result, the distal end of the anterior centrodiapophyseal lamina very probably harboured an accessory rib articular facet on this vertebra (figure 10*a,c,d*: 3rfa*?*). The prezygapophyseal and postzygapophyseal centrodiapophyseal fossae (*sensu* Wilson *et al.* [145]) are shallow and the latter is subdivided by a broadly convex surface. A posterodorsally-to-anteroventrally oriented tuberosity is present immediately ventral to the postzygapophyseal centrodiapophyseal fossa and at or close to the level of the neurocentral suture (figure 10*a*: tu), resembling the condition of Cv9 of some other early archosauriforms (e.g. *Chasmatosuchus rossicus*: PIN 2252/381; *Sarmatosuchus otschevi*: PIN 2865/68; *Garjainia prima*: [43]), although it is absent in *Proterosuchus alexanderi* (NMQR 1484) and *Vonhuenia friedrichi* (PIN 1025/11). The prezygapophysis is anterodorsally oriented and extends anteriorly beyond the level of the anterior margin of the centrum. The postzygapophysis extends posteriorly slightly beyond the level of the posterior margin of the centrum, and there is a very shallow lateral fossa immediately ventral to its base (figure 10*a*: fo), as in Cv8 of *Samsarasuchus pamelae*. There is no epipophysis on the dorsal surface or dorsal to the postzygapophysis, contrasting with Cv5 of *Samsarasuchus pamelae* and the probable Cv9 of *Chasmatosuchus rossicus* (PIN 2252/381). The articular facets of both zygapophyses slant medially. There is no hyposphene-hypantrum, as occurs in *Chasmatosuchus rossicus* (PIN 2252/381), *Proterosuchus alexanderi* (NMQR 1484) and *Vonhuenia friedrichi* (PIN 1025/11). By contrast, a hyposphene is present in *Sarmatosuchus otschevi* (PIN 2865/68).

The prespinal fossa is moderately deep and restricted to the base of the neural spine. The postspinal fossa is transversely broad and deeply invades the base of the neural spine (figure 10*c*: posf). There is a very shallow depression immediately lateral to the base of the neural spine (figure 10*a*: fo), which is shallower than that of the anterior postaxial cervical vertebrae and those of the posterior cervical vertebrae of *Chasmatosuchus rossicus* (PIN 2252/381; figure 9*k*: fo), *Vonhuenia friedrichi* (PIN 1025/11; figure 9*e*: fo) and *Garjainia prima* [43]. The neural spine is vertical and 1.23 times longer than the height of the posterior articular surface of the centrum. By contrast, the neural spine is proportionally shorter in Cv9 of *Sarmatosuchus otschevi* (0.80: PIN 2865/68) and *Vonhuenia friedrichi* (0.91: PIN 1025/11), but taller in *Proterosuchus alexanderi* (1.44: NMQR 1484) and *Garjainia prima* (*ca* 1.45: PIN 951/64-9). The neural spine expands slightly anteroposteriorly towards its distal end as a result of slightly divergent and straight anterior and posterior margins, resembling the condition in *Garjainia prima* (PIN 951/64-9), but most other early archosauriforms have rather parallel anterior and posterior margins of the posteriormost cervical neural spine (e.g. *Proterosuchus alexanderi*: NMQR 1484; *Vonhuenia friedrichi*: PIN 1025/11; *Sarmatosuchus otschevi*: PIN 2865/68). The degree of anteroposterior expansion of the neural spine is lower than that of Cv8 of *Samsarasuchus pamelae*. The neural spine expands transversely towards its distal end and has flat lateral surfaces. The anterior surface of the neural spine is slightly transversely convex and most of the posterior surface is covered by a rugose, raised area that may have been the attachment area of interspinous ligaments. The distal end of the neural spine possesses a pair of laterally well developed and rounded mammillary processes (figure 10*a–e*: mp), as occurs in ‘*Chasmatosaurus*’ *yuani* (IVPP V4067) and *Garjainia prima* (PIN 951/64-9), but not in most early archosauriforms (e.g. *Vonhuenia friedrichi*: PIN 1025/11; *Sarmatosuchus otschevi*: PIN 2865/68). These processes are situated on the anterior two-thirds of the neural spine and project anteriorly slightly beyond it, resulting in a transversely concave anterior margin of the neural spine in dorsal view. This condition seems to be unique to Cv9 of *Samsarasuchus pamelae* because these structures do not extend anteriorly to the rest of the neural spine in the cervical series of the vast majority of archosauromorphs with mammillary processes (e.g. *Shringasaurus indicus*: [105], ISIR specimens; *Proterosuchus fergusi*: NMQR 1484; ‘*Chasmatosaurus*’ *yuani*: IVPP V4067; *Garjainia prima*: PIN 951/64-9). In particular, the mammillary processes of the tenth presacral vertebra of *Prolacerta broomi* extend anteriorly to the rest of the spine but each of them is separated from the spine by a distinct vertical cleft, resulting in a three-pointed anterior margin of the neural spine (BP/1/2675) rather than the continuously concave margin of *Samsarasuchus pamelae*. The mammillary processes are confluent with the distal margin of the neural spine at its anterior margin in *Samsarasuchus pamelae*, but posteriorly they curve distally and are situated slightly ventral to the distal margin. The posterior end of the neural spine also possesses a pair of low, rounded dorsal expansions, which are separated from each other by a median depression that extends slightly onto the posterior surface of the spine. As a result of the presence of two pairs of mammillary processes anteriorly and posteriorly on the spine, respectively, the distal surface of the neural spine acquires an X-shaped profile in dorsal view (figure 10*e*), which is not present in other vertebrae referred to *Samsarasuchus pamelae*, nor in other archosauromorph species of which we are aware.

#### 4.1.2. Dorsal vertebrae

##### 4.1.2.1. Anterior dorsal vertebrae

There are 11 preserved anterior dorsal vertebrae, which belong to at least five different positions in the trunk series. These different positions are described as follows as positions A−E, which correspond to sequentially more anterior to more posterior elements.

*Position A (probable D1)*. This position is represented by NHMUK PV R37583 (figure 11 and table 5). This specimen preserves most of the centrum, but with damaged anterior and posterior rims, which gives the vertebra an apparently dorsoventrally shorter appearance than in posterior cervical and other anterior dorsal vertebrae. NHMUK PV R37583 lacks most of the right diapophysis, the distal end of the left diapophysis, both prezygapophyses, most of the right postzygapophysis, the central portion of the neural arch, and the vast majority of the neural spine. The morphology of this vertebra is very similar to that of Cv9 of *Samsarasuchus pamelae*, sharing the presence of a median ventral keel on the centrum and a similar orientation of the diapophysis. However, NHMUK PV R37583 differs from Cv9 of *Samsarasuchus pamelae* in the presence of a more dorsally positioned parapophysis. Thus, NHMUK PV R37583 is interpreted as a more posterior element than Cv9 and probably corresponds to D1 of *Samsarasuchus pamelae*. Specimens referred to *Vonhuenia friedrichi* (PIN 1025/419; [41]), *Gamosaurus lozovskii* (PIN 3361/213; [41]) and the ‘Arcadia proterosuchian’ (QMF9530; [111]) are isolated vertebrae with a morphology of the rib facets consistent with that of NHMUK PV R37583 and also probably represent D1. Similarly, a vertebra of the holotype of *Cuyosuchus huenei* (MCNAM PV 2669) also has a similar morphology of the rib facets and probably represents the tenth presacral element. These specimens and the D1 of other early archosauriforms with more complete cervico-dorsal vertebral sequences (e.g. *Proterosuchus fergusi*, *Proterosuchus alexanderi*, *Garjainia prima*, *Bharitalasuchus tapani*) are compared with NHMUK PV R37583. General features that this specimen shares with Cv9 of *Samsarasuchus pamelae* are not described (e.g. diagonal tuberosity on the centrodiapophyseal fossa; an anterodorsally-to-posteroventrally oriented ridge on the lateral surface of the base of the prezygapophysis; fossa posteroventrally to the base of the postzygapophysis; the absence of hyposphene).

The centrum of NHMUK PV R37583 is less than 1.33 times longer than the height of its anterior articular surface, but this ratio is unlikely to be considerably lower than 1.33 because not much of the rims of the centrum are missing. A ratio of or close to one occurs in *Proterosuchus alexanderi* (1.00: NMQR 1484), *Garjainia prima* (0.97: PIN 951/64-10) and *Cuyosuchus huenei* (1:03: MCNAM PV 2669), whereas a considerably lower value occurs in *Sarmatosuchus otschevi* (0.78: PIN 2865/68) and more extremely in *Bharitalasuchus tapani* (0.58: [102]). By contrast, a proportionally longer centrum occurs in the specimen referred to as *Gamosaurus lozovskii* (1.69: PIN 3361/213). It cannot be determined if the centrum of NHMUK PV R37583 was parallelogram-shaped in lateral view because of damage. The centrum possesses a low median longitudinal keel (figure 11*f*: vk), resembling the condition of Cv9 of *Samsarasuchus pamelae* and the 10th presacral vertebra of *Garjainia prima* (PIN 951/64-10). A more subtle ventral keel is present in D1 of *Proterosuchus alexanderi* (NMQR 1484) and the specimen referred to *Gamosaurus lozovskii* (PIN 3361/213), whereas a well-developed ventral keel is present in the probable D1 of *Vonhuenia friedrichi* (PIN 1025/419) and, at least on the anteriormost region of the centrum, of the ‘Arcadia proterosuchian’ (QMF9530). The D1 of *Sarmatosuchus otschevi* (PIN 2865/68-20) and *Cuyosuchus huenei* (MCNAM PV 2669) both lack a ventral keel. The neurocentral suture of NHMUK PV R37583 is closed.

The neural arch possesses anterior centrodiapophyseal, prezygodiapophyseal, postzygodiapophyseal laminae, and a very short posterior centrodiapophyseal lamina. This condition differs from the absence of a posterior centrodiapophyseal lamina in the cervical vertebrae. The above-mentioned set of four laminae is also present in D1 of *Garjainia prima* (PIN 951/64-10) and *Cuyosuchus huenei* (MCNAM PV 2669), whereas *Sarmatosuchus otschevi* (PIN 2865/68) and the probable D1 of *Vonhuenia friedrichi* (PIN 1025/419) lack only the posterior centrodiapophyseal laminae. *Bharitalasuchus tapani* has anterior and posterior centrodiapophyseal laminae, but the presence of the zygodiapophyseal laminae cannot be determined in this species because of damage [102]. The presence of a third rib articular facet cannot be determined in D1 of *Samsarasuchus pamelae* because of damage, but it is present in D1 of *Garjainia prima* [140] and the probable D1 of *Vonhuenia friedrichi* (PIN 1025/419) and *Cuyosuchus huenei* (MCNAM PV 2669).

*Position B (probable D2)*. An anterior dorsal vertebra (GSI 2117; Huxley [58]: plate 2, figure 6; figure 12 and table 5) has a morphology similar to that of Cv8, Cv9 and probable D1 of *Samsarasuchus pamelae*, but the parapophysis is situated slightly more dorsally on the centrum and the transverse process is more horizontally projected, which are features consistent with a more posterior position in the dorsal series (probably D2). This position in the dorsal series is bolstered by the resemblance of GSI 2117 to the 11th presacral vertebra (D2) of *Garjainia prima* (PIN 951/64-11). GSI 2117 lacks most of the right transverse process, posteroventral corner of the right side of the neural arch, both postzygapophyses and neural spine.

The centrum of GSI 2117 is 0.88 times longer than the height of its anterior articular surface, whereas the centrum of D2 is slightly proportionally longer in *Garjainia prima* (1.03: PIN 951/64-11). The centrum is moderately transversely compressed at mid-length and spool-shaped in ventral view (figure 12*f*). The centrum is slightly parallelogram-shaped in lateral view, with the anterior articular surface being situated slightly dorsal to the posterior one (figure 12*a*,*b*). The anterior and posterior articular facets of the centrum are gently concave and sub-circular. There is no notochordal pit, but the deepest area of the facets is situated in the same position as the notochordal pit when it is present in other vertebrae of *Samsarasuchus pamelae*. The ventral surface of the centrum is continuously transversely convex, lacking a median keel or groove, as is the case in *Garjainia prima* (PIN 951/64-11). By contrast, the cervical and probable first dorsal centra of *Samsarasuchus pamelae* have a median keel. The parapophysis is situated on the anteroventral corner of the centrum, slightly ventral to the level of mid-height of the anterior articular surface. The articular facet of the parapophysis is oval, with a dorsoventral main axis, and mainly laterally facing, but with a slight ventral orientation (figure 12: pa). The parapophysis is low and extends laterally slightly beyond the level of the lateral margin of the anterior articular facet of the centrum. The lateral surface of the centrum possesses a shallow, poorly rimmed lateral fossa, which is situated immediately ventral to the boundary with the neural arch. There are multiple probable nutrient foramina piercing the lateral surface of the centrum, which are mainly positioned immediately posterodorsally to the parapophysis and close to the posterior margin of the centrum. The neurocentral suture is closed.

The neural arch possesses anterior centrodiapophyseal, prezygodiapophyseal and postzygodiapophyseal laminae (figure 12: acdl, podl, prdl), whereas the posterior centrodiapophyseal lamina is absent. By contrast, these four laminae are present in D2 of *Garjainia prima* [140]. The diapophysis is mainly laterally oriented, but with a slight ventral slant, and extends considerably beyond the level of the lateral margin of the centrum (figure 12: di). The articular facet of the diapophysis is flat and oval, with an anteroventrally-to-posterodorsally oriented main axis. The anterior centrodiapophyseal lamina is anteroventrally oriented and ends at the anterodorsal corner of the centrum, being separated from the parapophysis by a dorsoventrally concave, broad surface. The distal end of the anterior centrodiapophyseal lamina is not expanded, contrasting with the condition in Cv8 and Cv9 of *Samsarasuchus pamelae*. The prezygapophyseal centrodiapophyseal fossa is very shallow, being represented by a dorsoventrally concave surface. The postzygapophyseal centrodiapophyseal fossa is deep, small and mainly laterally facing. There is a second, considerably shallower, fossa placed immediately posterodorsal to the postzygapophyseal centrodiapophyseal fossa. This shallower fossa is subdivided by a low, anterodorsally-to-posteroventrally oriented strut. A posterodorsally-to-anteroventrally oriented tuberosity is present immediately ventral to the postzygapophyseal centrodiapophyseal fossa and probably placed at or close to the level of the neurocentral suture (figure 12*b*: tu), resembling the condition present in the other cervico-dorsal vertebrae of *Samsarasuchus pamelae* and *Garjainia prima* [43].

The prezygapophysis is anterodorsally oriented and extends anteriorly slightly beyond the level of the anterior margin of the centrum. There is a moderately deep lateral fossa immediately ventral to the base of the postzygapophysis (figure 12*b*,*d*: fo), as in Cv8 and Cv9 of *Samsarasuchus pamelae*. The articular facets of both zygapophyses slant medially. The articular facet of the prezygapophysis is oval, with a transversely oriented main axis. There is no hyposphene-hypantrum, but the presence of an epipophysis cannot be determined because of breakage. The prespinal fossa is moderately deep and restricted to the base of the neural spine (figure 12*c*: prsf). The postspinal fossa is transversely broad and invades the base of the neural spine towards its distal end as far as it is preserved (figure 12*d*: posf). There is a very shallow depression immediately lateral to the base of the neural spine, resembling the condition of Cv9 of *Samsarasuchus pamelae*. By contrast, this fossa is considerably deeper in D2 of *Proterosuchus alexanderi* (NMQR 1484) and *Proterosuchus fergusi* (SAM-PK-K140). The anterior and posterior surfaces of the neural spine are covered by a rugose, raised area that may have been the attachment area of interspinous ligaments.

*Position C (probable D3)*. This position in the anterior dorsal series is represented by two vertebrae of congruent morphology. GSI 2116 (Huxley [58]: plate II, figure 5; figure 13 and table 5) is fairly complete, only missing the right posterolateral margin of the centrum, right postzygapophysis, most of the right diapophysis, the distal end of the left diapophysis and the right side of the distal end of the neural spine. The other vertebra, ISIR 1092, lacks most of its neural arch and right side of the centrum (table 5). These vertebrae possess a parapophysis situated at a very similar level on the anterior margin of the centrum as in the probable D2 of *Samsarasuchus pamelae* (GSI 2117), but they are more laterally developed than in the latter specimen. There is a paradiapophyseal lamina in both probable D3 vertebrae, contrasting with the presence of an anterior centrodiapophyseal lamina, which does not reach the parapohysis, in Cv8, Cv9, and the probable D2. As a result, GSI 2116 and ISIR 1092 are interpreted as more posterior elements than D2, probably representing D3.

The centrum is 1.14 times longer than the height of its anterior articular surface in GSI 2116 and greater than 1.26 in ISIR 1092, resembling the condition in *Proterosuchus fergusi* (1.13: SAM-PK-11208, an anterior dorsal vertebra posterior to D2). By contrast, a proportionally shorter centrum occurs in the 12 presacral vertebra (probable D3) of *Garjainia prima* (1.06: PIN 951/64-12) and more conspicuously in *Erythrosuchus africanus* (0.48: Gower [141]: table 1, estimated presacral 11 of BP/1/4680). The centrum is moderately transversely compressed at mid-length and spool-shaped in ventral view. The centrum is very slightly parallelogram-shaped in lateral view at least in ISIR 1092, but the condition cannot be determined in GSI 2116 because of breakage. The anterior and posterior articular facets of the centrum are gently concave and sub-circular. There is a moderately deep notochordal pit on the anterior and posterior articular surfaces of both vertebrae, which are slightly displaced dorsally from the centre of the facet (figure 13*c,d*). The ventral surface of the centrum is continuously transversely convex and lacks a median keel or groove, as in the probable D2, but a very low ventral keel is present in the anterior dorsal vertebrae of *Proterosuchus fergusi* (SAM-PK-11208) and *Garjainia prima* (PIN 951/64-12). The parapophysis is situated on the anteroventral corner of the centrum and its articular facet is mainly laterally facing, but with a ventral slant (figure 13: pa). The parapophysis is laterally projected on a low peduncle and extends laterally slightly beyond the level of the lateral margin of the anterior articular facet of the centrum. The lateral surface of the centrum possesses a shallow, not well-defined, lateral fossa, which is placed immediately ventral to the boundary with the neural arch. The neurocentral suture is closed in GSI 2116, but it is still clearly visible on the lateral surface and internal wall of the neural canal of ISIR 1092. The latter condition indicates that ISIR 1092 was probably not a skeletally mature individual at the time of its death. This interpretation is bolstered by the fact that ISIR 1092 is 62–73% the size of GSI 2116.

The neural arch (mostly absent in ISIR 1092) possesses paradiapophyseal, posterior centrodiapophyseal, prezygodiapophyseal and postzygodiapophyseal laminae (figure 13: pcdl, pdl, podl, prdl), as occurs in *Garjainia prima* [140]. The diapophysis is mainly laterally oriented, but with a low ventral slant (figure 13: di). The paradiapophyseal lamina is mainly vertically oriented, with a slight anterior slanting. The prezygapophyseal and postzygapophyseal centrodiapophyseal fossae are shallow, whereas the centrodiapophyseal fossa is deeper and lateroventrally facing. A posterodorsally-to-anteroventrally oriented tuberosity, but with a nearly vertical main axis, is present immediately posteroventral to the centrodiapophyseal fossa (figure 13*a*: tu), resembling the condition present in some other early archosauriforms (e.g. *Garjainia prima*: [43]). The prezygapophysis is anterodorsally oriented and extends anteriorly beyond the level of the anterior margin of the centrum. The lateral surface of the prezygapophysis possesses an anterodorsally-to-posteroventrally oriented and very thin ridge that is very rugose on its anterior third (figure 13*b*: ri). This ridge is homologous to that present in more anterior vertebrae of *Samsarasuchus pamelae*, but the rugose anterior portion of the ridge is not present in other vertebrae of the species. The postzygapophysis is short and does not extend posteriorly beyond the level of the posterior margin of the centrum. There is a very shallow lateral fossa immediately ventral to the base of the postzygapohysis (figure 13*a*: fo), as in posterior cervical and more anterior dorsal vertebrae. The articular facets of both zygapophyses slant medially and are oval, with a transverse main axis. There is no hyposphene-hypantrum, nor an epipophysis. The D2 of *Proterosuchus alexanderi* (NMQR 1484), *Proterosuchus fergusi* (SAM-PK-K140) and *Garjainia prima* (PIN 951/64-12) also lack epipophyses.

The prespinal fossa is moderately deep and restricted to the base of the neural spine (figure 13*c*: prsf). The postspinal fossa is transversely broad and invades the ventral half of the neural spine, being deeper towards the base of the neural spine (figure 13*d*: posf). There is a very shallow depression immediately lateral to the base of the neural spine, contrasting with the presence of a deeper fossa in *Proterosuchus fergusi* (SAM-PK-11208) and *Proterosuchus alexanderi* (NMQR 1484). The neural spine is vertical and slightly less than two times higher than it is anteroposteriorly long at its base. The neural spine expands slightly anteroposteriorly towards its distal end as a result of slightly divergent and straight anterior and posterior margins, resembling the condition in Cv7 of *Samsarasuchus pamelae*. The lateral surface of the neural spine is flat. The anterior and posterior surfaces of the neural spine are covered by a rugose, raised area that may have been the attachment area of interspinous ligaments. The neural spine expands transversely towards its distal end and the distal end possesses a pair of laterally well-developed and rounded mammillary processes (figure 13*b–e*: mp), as in Cv7–9 of *Samsarasuchus pamelae* (the condition in Cv6 and probable D1 and D2 is unknown) and D3 of *Proterosuchus alexanderi* (NMQR 1484) and ‘*Chasmatosaurus*’ *yuani* (IVPP V4067), whereas incipient mammillary processes occur in the probable D3 (12 presacral vertebra) of *Garjainia prima* [140]. These processes are slightly anteriorly displaced from the mid-length of the neural spine, but well separated from its anterior margin, resembling the condition in D3 of *Proterosuchus alexanderi* (NMQR 1484). Cv8 of *Samsarasuchus pamelae* has more anteriorly positioned mammillary processes but resembles the probable D3 in the presence of processes well separated from the anterior margin of the neural spine. By contrast, the mammillary processes of Cv9 of *Samsarasuchus pamelae* extend anteriorly beyond the level of the rest of the neural spine. The mammillary processes of the probable D3 are distinctly separated anteriorly from the rest of the spine in dorsal view, but posteriorly they merge gradually with the rest of the spine. The mammillary processes are placed slightly ventral to the distal margin of the neural spine. The distal surface of the neural spine is flat and sub-rectangular.

*Position D (probable D4)*. This position is probably represented by two vertebrae. ISIR 1098 lacks the posteroventral corner of the centrum, both diapophyses and prezygapophyses, left postzygapophysis and neural spine (figure 14 and table 5). This vertebra possesses a parapophysis more dorsally placed than the previously described anterior dorsal vertebrae, being situated at mid-height in the centrum, and connected with the diapophysis by a long, slightly posteriorly curved paradiapophyseal lamina. The other vertebra, ISIR 1099 (table 5), is interpreted to belong to an element approximately of the same position as ISIR 1098 because of a similar position of the base of the parapophysis and because it also possesses a median longitudinal keel, contrasting with the probable D2 and D3. ISIR 1099 lacks most of its neural arch and anteroventral end of the centrum. The centrum of ISIR 1099 and of another relatively large, but considerably smaller than ISIR 1099, anterior dorsal vertebra (GSI 2117) are proportionally shorter than smaller anterior dorsal vertebrae and possess a deeper fossa on the lateral surface of the centrum. These differences are likely related to ontogenetic variation.

The length of the centrum is 1.25 times the height of its anterior articular surface in ISIR 1098 (figure 14) and less than 1.11 in ISIR 1099, resembling the condition in the posterior anterior dorsal vertebrae of *Proterosuchus fergusi* (0.96–1.19: SAM-PK-K140), a vertebra assigned to cf. *Proterosuchus* from Brazil (UNIPAMPA 271; [110]), and *Garjainia prima* (1.19: PIN 951/64-13). By contrast, the posterior centra of the anterior dorsal region are proportionally shorter in *Bharitalasuchus tapani* (0.84–0.86: [102]) and *Erythrosuchus africanus* (0.55: Gower [141]: table 1, figure 23, estimated presacral 12 of NHMUK PV R3592 large), and proportionally longer in a probable D4 or D5 of *Cuyosuchus huenei* (*ca* 1.4: MCNAM PV 2669). The ventral surface of the centrum possesses a very low median longitudinal keel that extends along the entire preserved portion (figure 14*f*: vk), as in *Garjainia prima* [140] and *Bharitalasuchus tapani* [102], and some specimens of *Proterosuchus fergusi* have a keel and others do not (SAM-PK-K140; SAM-PK-11208). The lateral surface of the centrum is anteroposteriorly concave and possesses a shallow lateral fossa lacking a pronounced rim. There are multiple small, circular to oval nutrient foramina on the lateral surface of the centrum, which are mainly grouped anteroventral to the parapophysis or close to the posterior margin, as occurs in several more anterior presacral vertebrae. The articular surfaces of both parapophyses are damaged (figure 14*a*: pa). The neurocentral suture is closed in ISIR 1098.

The posteroventral corner of the neural arch possesses a broadly anteroposteriorly convex surface with multiple striations on its lateral surface. The neural arch possesses paradiapophyseal and posterior centrodiapophyseal laminae (figure 14*a*,*d*: pdl, pcdl), as in *Garjainia prima* (PIN 951/64-13) and *Cuyosuchus huenei* (MCNAM PV 2669), but the latter lamina is absent in *Proterosuchus fergusi* (SAM-PK-K140) and the cf. *Proterosuchus* from Brazil (UNIPAMPA 271; [110]). The centrodiapophyseal fossa is subdivided by three anterodorsally-to-posteroventrally oriented and thin internal ridges that are parallel to each other (figure 14*a*: ri). The cortical surface of the centrodiapophyseal fossa between the most posterior internal ridge and the posterior centrodiapophyseal fossa seems to be lost and exposes a honeycomb-like internal structure. It is not possible to determine the presence of a prezygodiapophyseal lamina because of breaks. The postzygapophysis is mainly laterally oriented in dorsal view, with the main axis forming an angle of 60° with the sagittal axis of the trunk, resembling the condition present in dorsal vertebrae of a probable similar position in *Proterosuchus alexanderi* (NMQR 1484) and ‘*Chasmatosaurus*’ *yuani* (IVPP V4067). As a result, the postzygapophysis extends posteriorly only slightly beyond the posterior margin of the centrum in lateral view. The articular facet of the postzygapophysis is lateroventrally facing and kidney-shaped, with a concave anterior margin and a transverse main axis. The base of the postzygapophysis is delimited ventrally by a shallow, subtriangular fossa (figure 14*a*: fo). The postzygapophysis extends medially as a medioventrally oriented shelf, but it is not possible to determine if it reaches the median line because it is broken off. There is no epipophysis.

*Position E (probable D5–D6)*. This position is represented by five specimens: ISIR 1094 (figure 15*i*–*m*,*q* and table 6), ISIR 1096 (figure 15*a*–*c*,*e*–*g* and table 6), ISIR 1097 (figure 15*d*,*h*), GSI 2260 (Huxley, 1865: figure 5; figure 15*p* and table 5) and NHMUK PV R37577 (figure 15*n*,*r* and table 5). These vertebrae possess a parapophysis situated on the dorsal half of the centrum (not preserved in ISIR 1094) and a transverse process placed level with the roof of the neural canal (not preserved in ISIR 1097 and NHMUK PV R37577), contrasting with the more anterior vertebrae of *Samsarasuchus pamelae*. ISIR 1094 lacks the parapophyses, distal ends of the transverse processes, prezygapophyses and neural spine, and the anterior and posterior surfaces of the centrum are damaged. ISIR 1096 lacks the right diapophysis, distal ends of both postzygapophyses, and most of the neural spine. ISIR 1097 lacks both prezygapophyses, right postzygapophysis and both diapophyses. GSI 2260 lacks most of the transverse processes, right prezygapophysis, postzygapophyses and neural spine. Finally, NHMUK PV R37577 lacks the right anterolateral margin of the centrum (including the right parapophysis) and most of the neural arch.

The length of the centrum is 1.26 times the height of its anterior articular surface in GSI 2260 and *ca* 1.3 times in NHMUK PV R37577, whereas it is proportionally longer in *Cuyosuchus huenei* (*ca* 1.5: MCNAM PV 2669). Proportionally shorter centra occur in the D5 and D6 (14th and 15th presacral vertebrae) of *Garjainia prima* (1.09–1.16: PIN 951/64-14, 64-15) and dorsal vertebrae of probable similar position in *Bharitalasuchus tapani* (0.84–0.97: [102]) and *Erythrosuchus africanus* (0.65–0.70: Gower [141]: table 1, anterior–middle dorsal of NHMUK PV R3592 large). The anterior and posterior articular surfaces of the centrum are concave and slightly taller than broad. The centrum is moderately transversely compressed at mid-length and, as a result, spool-shaped in ventral view. The ventral surface of the centrum is continuously transversely convex and most specimens lack a median keel (ISIR 1094, ISIR 1096, ISIR 1097, GSI 2260), but NHMUK PV R37577 has a very low keel (figure 15*r*: vk). Dorsal vertebrae of the probable same position have a ventral keel in *Bharitalasuchus tapani* [102] and *Erythrosuchus africanus* (NHMUK PV R3592), but not in *Proterosuchus fergusi* (SAM-PK-K140) or *Cuyosuchus huenei* (MCNAM PV 2669). The lateral surface of the centrum is anteroposteriorly concave and lacks a lateral fossa. This surface is pierced by a circular foramen placed posteroventrally to the base of the parapophysis and by some other more posterior, randomly distributed foramina in ISIR 1094, ISIR 1096 and ISIR 1097. The parapophysis is slightly raised, projecting laterally only slightly beyond the lateral margin of the anterior articular surface of the centrum. The ventral margin of the parapophysis is placed level with the mid-height of the centrum and dorsally extends dorsal to the level of the floor of the neural canal (figure 15: pa). The articular surface of the parapophyses is slightly anteriorly bowed in lateral view, lateroventrally facing, and dorsoventrally concave. There is no centroparapophyseal lamina. The neurocentral suture is closed in all five specimens.

A well-developed posterodorsally-to-anteroventrally oriented tuberosity is present posterior to the parapophysis in all the vertebrae of this position (figure 15: tu). This tuberosity and the broadly convex posteroventral corner of the neural arch form a distinct inverted V-shaped boundary between the neural arch and centrum. The neural arch possesses paradiapophyseal, posterior centrodiapophyseal and prezygodiapophyseal laminae (figure 15: pdl, pcdl, prdl), as occurs in vertebrae of a similar position in *Cuyosuchus huenei* (MCNAM PV 2669), *Garjainia prima* [140] and *Erythrosuchus africanus* (NHMUK PV R3592), whereas the latter lamina is absent in the posterior anterior dorsal vertebrae of *Proterosuchus fergusi* (SAM-PK-K140). The posterior centrodiapophyseal lamina of *Samsarasuchus pamelae* is posteroventrally oriented and very short, finishing well dorsal to the boundary between the neural arch and the centrum. As a result, the postzygapophyseal centrodiapophyseal and centrodiapophyseal fossae are small and shallow. The diapophysis is situated immediately dorsal to the level of the dorsal border of the neural canal and on the anterior half of the neural arch (figure 15: di). The diapophysis is posterolaterally oriented in dorsal view, resembling the condition in *Garjainia prima* (PIN 951/64-14, 64-15) and *Erythrosuchus africanus* (NHMUK PV R3592). The diapophysis of *Samsarasuchus pamelae* extends posteriorly as a horizontal shelf but fails to reach the base of the postzygapophysis, contrasting with the presence of a postzygodiapophyseal lamina in the probable D5–D6 of *Cuyosuchus huenei* (MCNAM PV 2669), *Garjainia prima* [140] and *Erythrosuchus africanus* (NHMUK PV R3592). The prezygapophysis is anterodorsally oriented in lateral view and extends anteriorly to the level of the anterior margin of the centrum. The articular facet of the prezygapophysis is oval, with a transverse main axis and slants medially. The postzygapophysis is poorly posteriorly projected and would have extended posteriorly approximately to the same level as the posterior margin of the centrum. Its articular facet is oval, with a transverse main axis, and slants medially. There is no hyposphene, nor an epipophysis. There is a shallow, circular depression immediately ventral to the base of the postzygapophysis (figure 15*a*,*i*: fo), as in the posterior cervical and more anterior dorsal vertebrae. Similarly, a shallow depression is present immediately lateral to the base of the neural spine (figure 15*f*,*m*: fo), as in ‘*Chasmatosaurus*’ *yuani* (IVPP V4067), whereas this depression is deeper in *Proterosuchus alexanderi* (NMQR 1484) and *Proterosuchus fergusi* (SAM-PK-11208), and even deeper and posteriorly well-defined in *Erythrosuchus africanus* (NHMUK PV R3592).

The prespinal and postspinal fossae are very deep, transversely broad and extend along most of the neural spine, but they do not reach the distal end of the process (figure 15: posf, prsf). The neural spine is trapezoidal in lateral view, with gently divergent anterior and posterior margins, and its main axis slants slightly posteriorly in lateral view. Complete mammillary processes are preserved in ISIR 1097 (figure 15*d*,*h*: mp) and their bases are preserved in ISIR 1094. These processes are broadly anteroposteriorly convex, slightly anteriorly displaced from the anteroposterior mid-length of the distal end of the neural spine, and slightly ventrally displaced from the distal margin of the neural spine, resembling the position of, but being distinctly less laterally developed than, the mammillary processes in the probable D3 of *Samsarasuchus pamelae*. The mammillary processes are also well developed in D5–D6 of *Proterosuchus alexanderi*, but positioned at mid-length on the distal end of the spine in D5 and posteriorly displaced from mid-length in D6 (NMQR 1484). By contrast, mammillary processes are absent in D5–D6 of *Proterosuchus fergusi* (SAM-PK-11208), ‘*Chasmatosaurus*’ *yuani* (IVPP V4067), *Garjainia prima* [140] and *Cuyosuchus huenei* (MCNAM PV 2669). The distal surface of the neural spine of *Samsarasuchus pamelae* is broad and flat.

##### 4.1.2.2. Middle dorsal vertebrae (probable D7–D11)

**Figure 17.**
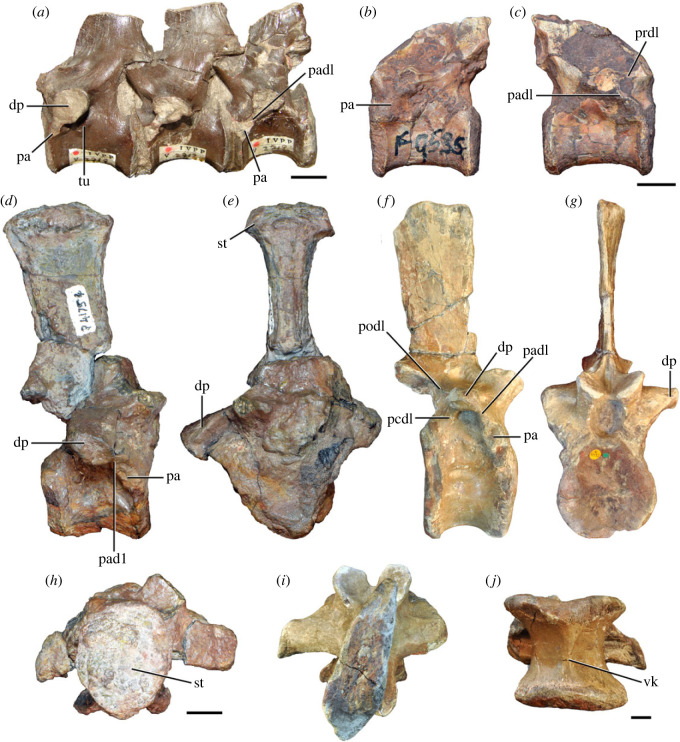
Comparison between middle dorsal vertebrae of selected Early and Middle Triassic non-eucrocopodan archosauriforms. (*a*) ‘*Chasmatosaurus*’ *yuani* (IVPP V2719), (*b, c*) ‘Arcadia proterosuchian’ (QMF9535), (*d, e, h*) ‘Long Reef proterosuchian’ (SAM P41754), and (*f, g, i, j*) *Erythrosuchus africanus* (NHMUK PV R3592) in (*a, b*) left lateral, (*c, d, f*) right lateral, (*e, g*) anterior, (*h, i*) dorsal, and (*j*) ventral views. dp, diapophysis; pa, parapophysis; padl paradiapophyseal lamina; pcdl, posterior centrodiapophyseal lamina; podl, postzygodiapophyseal lamina; prdl, prezygodiapophyseal lamina; st, spine table; tu, tuberosity; vk, ventral keel. Scale bars equal 1 cm in (*a, d, e, h*), 5 mm in (*b, c*), and 2 cm in (*f, g, i, j*).

There are four dorsal vertebrae (GSI 2261, ISIR 1101–1103; table 6) with the parapophysis and diapophysis situated level with the floor and roof of the neural canal, respectively (figure 16). As a result, these vertebrae are interpreted to belong to the middle dorsal series. In particular, ISIR 1101 possesses more strongly developed mammillary processes than ISIR 1102 (the neural spine is not preserved in GSI 2261 and ISIR 1103) and, therefore, the former vertebra is interpreted to belong to a more anterior position in the middle dorsal series. Nevertheless, all these vertebrae are described together because of their similar morphology. In addition, an isolated centrum (ISIR 1104) may also represent a middle dorsal vertebra. The position of the parapophyses and diapophyses of these vertebrae resembles that in the two vertebrae of the ‘Long Reef proterosuchian’ [114] (figure 17*d*,*e*,*h*). Thus, the latter vertebrae are interpreted here as middle dorsal elements and are compared here with those of *Samsarasuchus pamelae*.

GSI 2261 lacks the left posterolateral corner of the centrum, right side of the neural arch, all the zygapophyses, most of the left diapophysis and the neural spine (figure 16*m*,*n*,*r*). ISIR 1102 lacks the right lateral border of the posterior end of the centrum, right diapophysis and postzygapophysis, most of the left diapophysis and the posterodistal tip of the neural spine (figure 16*g*–*l*). The parapophyses and right lateral surface of the neural spine are damaged. ISIR 1101 is a transversely compressed vertebra that lacks part of the centrum at mid-length, both prezygapophyses, left transverse process and most of the right transverse process (figure 16*a*–*f*). The anterior and posterior surfaces of the centrum and anterior margin of the neural spine are damaged. Finally, ISIR 1103 and ISIR 1104 are complete centra, but lack almost all of their neural arches (figure 16*o*–*q*).

The length of the centrum is 1.33 times the height of its anterior articular surface in GSI 2261, 1.34 in ISIR 1102, 1.43 in ISIR 1103 and approximately 1.30 in ISIR 1101. This variation falls within the range observed in the middle dorsal vertebrae of ‘*Chasmatosaurus*’ *yuani* (1.32–1.48: IVPP V2719; figure 17*a*) and *Garjainia prima* (1.27–1.55: PIN 951/64), whereas the centrum is slightly proportionally shorter in the ‘Long Reef proterosuchian’ (0.94 and 1.23: SAM P41754; figure 17*d*) and the probable middle dorsal vertebrae of *Proterosuchus fergusi* (1.19: SAM-PK-K140), *Cuyosuchus huenei* (1.17: MCNAM PV 2669) and *Bharitalasuchus tapani* (0.94–1.03: [102]). By contrast, the middle dorsal centra are drastically shorter in *Erythrosuchus africanus* (0.67–0.76: Gower [141]: table 1, middle dorsal vertebrae; figure 17*f*) and *Shansisuchus shansisuchus* (0.70–0.85: [146]: table 6, dorsal 21d, dorsal 22, 16). The centrum of a probable middle dorsal vertebra of the ‘Arcadia proterosuchian’ is proportionally longer than the above-mentioned taxa, but this could be a result of its small size in comparison with other specimens of the same assemblage (1.60: QMF9535; figure 17*b*,*c*). The centrum of the middle dorsal vertebrae of *Samsarasuchus pamelae* is slightly parallelogram-shaped in lateral view, with the anterior articular surface situated more dorsally than the posterior one. The anterior articular surface of the centrum is sub-circular (GSI 2261) to slightly sub-oval, being dorsoventrally taller than broad (ISIR 1103). The posterior articular surface is slightly dorsoventrally taller than broad (ISIR 1103), and both anterior and posterior surfaces are shallowly concave. The notochordal pit is circular, shallow and slightly displaced dorsally from the centre of both articular surfaces of the centrum. The centrum is slightly constricted transversely at mid-length and, as a result, is spool-shaped in ventral view. The ventral surface of the centrum possesses a low and sharp median longitudinal keel, resembling the condition in *Garjainia prima* [43] and *Erythrosuchus africanus* (NHMUK PV R3592; figure 17*j*: vk). This keel is less developed in the probable middle dorsal centra of *Bharitalasuchus tapani* [102] and the ‘Arcadia proterosuchian’ (QMF9535), and it is absent in *Proterosuchus fergusi* (SAM-PK-K140). The ventral keel of *Samsarasuchus pamelae* extends along the majority of the bone, but disappears before reaching the anterior and posterior margins of the centrum. The lateral surface of the centrum has a very shallow and poorly rimmed lateral fossa that is pierced by multiple, small foramina on both sides in ISIR 1102 and ISIR 1103. These foramina do not form a distinct cluster, contrasting with the condition in cervical and anterior dorsal vertebrae. By contrast, ISIR 1101 possesses a fossa, but not foramina, and GSI 2261 lacks a lateral fossa and foramina, with the lateral surface of the centrum being dorsoventrally convex. The neurocentral suture is closed in the four middle dorsal vertebrae, including ISIR 1104.

The parapophysis is placed on a low peduncle positioned on the anteroventral corner of the neural arch and extends laterally beyond the level of the lateral margin of the centrum, as is the case in the middle dorsal vertebrae of other early archosauriforms (e.g. ‘*Chasmatosaurus*’ *yuani*: IVPP V2719; the ‘Long Reef proterosuchian’: SAM P41754; *Bharitalasuchus tapani*: [102]). The articular facet of the parapophysis is anteroventrally-to-posterdorosally oriented, anteroposteriorly narrow, and gently convex. The parapophysis is connected with the diapophysis by a well-developed paradiapophyseal lamina, as in *Proterosuchus fergusi* (SAM-PK-K140), ‘*Chasmatosaurus*’ *yuani* (IVPP V2719; figure 17*a*: padl), the ‘Long Reef proterosuchian’ (SAM P41754: figure 17*d*: padl), the ‘Arcadia proterosuchian’ (QMF9535; figure 17*c*: padl) and erythrosuchids (e.g. *Erythrosuchus africanus*: [141], figure 17*f*: padl; *Bharitalasuchus tapani*: [102]; *Shansisuchus shansisuchus*: [146]). It is not possible to determine the presence of a prezygodiapophyseal lamina in any specimen, but the posterior centrodiapophyseal and postzygodiapophyseal laminae are absent, as is the case in the ‘Arcadia proterosuchian’ (QMF9535; figure 17*b*,*c*), the ‘Long Reef proterosuchian’ (SAM P41754; figure 17*d*) and ‘*Chasmatosaurus*’ *yuani* (IVPP V2719) (figure 17*a*). The posterior centrodiapophyseal lamina is also absent in *Proterosuchus fergusi* (SAM-PK-K140). However, the prezygodiapophyseal lamina is present in the ‘Arcadia proterosuchian’ (QMF9535; figure 17*c*: prdl), and all these laminae are present in *Erythrosuchus africanus* ([141]; figure 17*f*) and *Shansisuchus shansisuchus* [146]. A thick, low and anteroventrally-to-posterodorsally oriented tuberosity is present level with and posterior to the parapophysis, as occurs in the posterior cervical and anterior dorsal vertebrae and in the middle dorsal vertebrae of ‘*Chasmatosaurus*’ *yuani* (IVPP V2719; figure 17*a*: tu) and *Garjainia prima* [43,140]. The prezygapophysis extends anteriorly beyond the level of the anterior margin of the centrum. This process also possesses a very low dorsal orientation in lateral view, but it is considerably lower than in the posterior cervical vertebrae. The prezygapophyseal facet is sub-circular and mainly dorsally facing. The postzygapophysis extends posteriorly up to the same level as the posterior margin of the centrum and possesses a posterolaterally oriented main axis. The postzygapophyseal facet is oval and faces lateroventrally. There is no hyposphene, nor an epipophysis.

The neural spine is 1.25 times taller than its length at the base in ISIR 1102 and 1.57 times in ISIR 1101, falling within the range observed in *Proterosuchus alexanderi* (1.34–1.50: NMQR 1484) and *Cuyosuchus huenei* (1.50: MCNAM PV 2669), and being slightly taller than in *Proterosuchus fergusi* (1.10: SAM-PK-K140). By contrast, the middle dorsal neural spines are considerably taller than those of *Samsarasuchus pamelae* in the ‘Long Reef proterosuchian’ (1.70: SAM P41754; figure 17*d*) and particularly erythrosuchids (e.g. *Erythrosuchus africanus*: 2.52, NHMUK PV R3592, figure 17*f*; *Garjainia prima*: 1.92–2.27, PIN 951/64). The main axis of the middle dorsal neural spines of *Samsarasuchus pamelae* slants slightly posteriorly in lateral view in both ISIR 1101 and ISIR 1102, resembling the condition in the ‘Long Reef proterosuchian’ (SAM P41754), *Erythrosuchus africanus* [141], *Garjainia prima* [140] and *Shansisuchus shansisuchus* [146]. The posterior margin of the neural spine is straight, but the shape and orientation of the anterior margin cannot be determined because it is covered with matrix that cannot be removed without compromising the rest of the bone in ISIR 1102 and is damaged in ISIR 1101. The neural spine possesses a well-developed and laterally rounded mammillary process in ISIR 1101. This process is positioned slightly ventral to the distal margin of the neural spine and was probably anteriorly displaced from the mid-length of the spine. By contrast, ISIR 1102 possesses a considerably lower mammillary process that is restricted to the anterior half of the distal end and is confluent with the distal margin of the spine. The last mammillary process of *Proterosuchus alexanderi* occurs in D8; thus, these structures seem to be more posteriorly extended in the dorsal series in *Samsarasuchus pamelae*. In addition, another difference between both species is that in *Proterosuchus alexanderi* the last mammillary process is restricted to the posterior half of the neural spine (NMQR 1484). Mammillary processes are absent in the middle dorsal vertebrae of *Proterosuchus fergusi* (SAM-PK-K140), erythrosuchids (e.g. *Garjainia prima*: [140]; *Erythrosuchus africanus*: [141]), *Cuyosuchus huenei* (MCNAM PV 2669) and in the ‘Long Reef proterosuchian’ (SAM P41754) (figure 17*d*–*i*). The distal surface of the neural spine is transversely convex and very rugose in ISIR 1101, whereas in ISIR 1102 it is mostly flat and less rugose. The distal end of the neural spine of the ‘Long Reef proterosuchian’ (SAM P41754) has an archosaur-like spine table (figure 17*e*,*h*: st), which is absent in proterosuchids, *Cuyosuchus huenei*, erythrosuchids and *Samsarasuchus pamelae* [9,18]. The posterodistal corner of the neural spine of *Samsarasuchus pamelae* is only incipiently posteriorly projected, contrasting with the conspicuous anteroposterior expansion of the distal end of the neural spine of the ‘Long Reef proterosuchian’ (SAM P41754). The presence of a prespinal fossa cannot be determined because of breaks. The postspinal fossa is very deep and restricted to the very base of the neural spine. The damaged right side of the neural arch of ISIR 1102 exposes a large, oval and very deep pocket situated on the medial surface of the base of the postzygapophysis. It cannot be determined if this pocket opens into the postspinal fossa or was a completely internal feature of the neural arch.

## 5. Systematic palaeontology

Diapsida Osborn, 1903 [127] [Gauthier & de Queiroz (2020)] [128]

Archosauromorpha von Huene, 1946 [129] [Gauthier (2020)] [130]

Archosauriformes Gauthier, Kluge & Rowe, 1988 [131] [Gauthier (2020)] [132]

cf. Proterosuchidae von Huene, 1908 [133] *sensu* Ezcurra, Butler & Gower, 2013 [11]

Genus and species indeterminate

**Material.** GSI 2190, portion of horizontal process of the right maxilla; GSI 2259 (holotype of ‘*Ankistrodon indicus*’; Huxley [58]: figure 4), portion of distal end of horizontal process of right maxilla with two teeth; ISIR 1075, portion of the anterior process of left maxilla with four teeth; ISIR 1076, anterior tip of right maxilla with one tooth; GSI 18123, anterior half of left dentary with three teeth *in situ*; GSI 18124, anterior half of right dentary with four partial teeth *in situ*; ISIR 1078, ventral end of left quadrate; ISIR 1079, NHMUK PV R37582, axes; NHMUK PV R37584, NHMUK PV R37586, ISIR 1105–1107, ISIR 1112, posterior dorsal vertebrae; GSI 2118 (Huxley [58]: plate II, figure 7), GSI 2120 (Huxley [58]: plate III, figure 1), first sacral vertebra with ribs; ISIR 1109, ISIR 1110, second sacral vertebra with base of ribs; ISIR 1111, second sacral vertebra with ribs; NHMUK PV R37579, second sacral vertebra with ribs and fused intercentrum; ISIR 1116, partial second sacral vertebra with the bases of the ribs; NHMUK PV R37585, second sacral rib; GSI 2124 (Huxley [58]: plate III, figure 5), GSI 2125 (Huxley [58]: plate III, figure 6), GSI 2119 (Huxley [58]: plate II, figure 8), ISIR 1118–1120, middle caudal vertebrae; GSI 2126 (Huxley [58]: plate III, figure 7), ISIR 1121–1123, PGRU/GL/M/VF-003, posterior caudal vertebrae; ISIR 1124–1126, partial centra; PGRU/GL/M/VF-001, distal portion of left humerus; ISIR 1129, proximal half of right humerus; ISIR 1130, distal end of left fourth metatarsal; ISIR 1131, pedal ungual phalanx.

**Geographic occurrence.** All GSI specimens, ISIR 1079, ISIR 1112 and ISIR 1118 were collected in the Deoli locality, close to Deoli village; ISIR 1105, ISIR 1109–1111, ISIR 1116, ISIR 1119, ISIR 1121, ISIR 1123, NHMUK PV R37579, NHMUK PV R37582 and all PGRU/GL/M/VF specimens were collected in the Dumdumi locality, close to Dumdumi village; and NHMUK PV R37584–37586 were collected in the riverbank of the Damodar River, besides the Railway Bridge. Locality data for the GSI specimens are from Satsangi [45] and Blanford (in Huxley [58]), data from PGRU/GL/M/VF specimens is from S. Pal (personal communication 2021), data from the NHMUK specimens is from the records of that institution and data from the ISI specimens is from our own field records. Specimens not mentioned here lack precise locality data. All the localities occur in the west of West Bengal, northeast India.

**Stratigraphic occurrence.** All the specimens were collected in the yellow-brownish conglomeratic sandstones of the upper Panchet Formation (middle–late Induan), Damodar Basin.

Proterosuchidae von Huene, 1908 [133] *sensu* Ezcurra, Butler & Gower, 2013 [11]

Genus and species indeterminate

**Material.** ISIR 1077, right quadrate; ISIR 1127, ISIR 1128, left humeri; GSI 18125, left ilium; NHMUK PV R10149, left ilium (cast of an unknown specimen).

**Geographic occurrence.** GSI 18125, ISIR 1077 and ISIR 1127 were collected in the Deoli locality, close to Deoli village; and NHMUK PV R10149 were collected in the riverbank of the Damodar River, besides the Railway Bridge. Locality data for the GSI specimens are from Satsangi [45] and Blanford (in Huxley [58]), and data from the NHMUK specimens are from the records of that institution. All the localities occur in the west of West Bengal, northeast India.

**Stratigraphic occurrence.** Yellow-brownish conglomeratic sandstones of the upper Panchet Formation (middle–late Induan), Damodar Basin.

Proterosuchidae von Huene, 1908 [133] *sensu* Ezcurra, Butler & Gower, 2013 [11]

Chasmatosuchinae nov.

Genus and species indeterminate

**Material.** GSI 2121 (Huxley [58]: plate III, figure 2), GSI 2122 (Huxley [58]: plate III, figure 3), GSI 2123 (Huxley [58]: plate III, figure 4), ISIR 1113–1115, ISIR 1117, NHMUK PV R37576, NHMUK PV R37581, anterior caudal vertebrae.

**Geographic occurrence.** All GSI specimens and ISIR 1115 were collected in the Deoli locality, close to Deoli village; ISIR 1114, ISIR 1117 and NHMUK PV R37581 were collected in the Dumdumi locality, close to Dumdumi village; and NHMUK PV R37576 was collected in the Banspatali locality (=Banspetali locality in Das & Gupta [73]: figure 1). Locality data for the GSI specimens are from Satsangi [45] and Blanford (in Huxley [58]), data from the NHMUK specimens are from the records of that institution, and data from the ISI specimens are from our own field records. Specimens not mentioned here lack precise locality data. All the localities occur in the west of West Bengal, northeast India.

**Stratigraphic occurrence.** Yellow-brownish conglomeratic sandstones of the upper Panchet Formation (middle–late Induan), Damodar Basin.

**Taxonomic remarks.** These specimens are not referred to *Samsarasuchus pamelae* because we cannot identify the unique combination of character states listed in the diagnosis of the species. However, the morphology of these specimens is very similar to that present in other proterosuchids and their size matches that are expected for the inferred intraspecific ontogenetic range if a single proterosuchid species were present in the upper Panchet Formation. Thus, these specimens are referred to cf. Proterosuchidae, but with the following exceptions. The most complete quadrate has an angle between the posterior margins of the dorsal and ventral ends of 143°–158°. This feature is recovered as a synapomorphy of Proterosuchidae in one of our analyses (Analysis 6; see below). Similarly, both complete humeri have a transverse width of the proximal end versus total length of the bone of 0.48–0.70, which is recovered as a synapomorphy of Proterosuchidae in the same analysis (Analysis 6; see below). Both ilia have a main axis of the articular surface of the ischiadic peduncle posteroventrally oriented in ventral view as a result of a strong lateral projection of the peduncle, in which its lateralmost point exceeds that of the supraacetabular crest. This feature is only shared with *Proterosuchus alexanderi* (NMQR 1484) and a specimen referred to the proterosuchid *Vonhuenia friedrichi* (PIN 1025/406) among non-archosaurian archosauriforms. Thus, these character states, respectively, are used to assign the most complete quadrate and humeri and both available ilia to Proterosuchidae. In the case of the anterior caudal vertebrae, the presence of a very deep and well-defined fossa on the surface lateral to the base of the neural spine is only shared with anterior caudal vertebrae referred to the chasmatosuchine proterosuchid *Chasmatosuchus rossicus* (PIN 2243/167, 2252/384) among non-eucrocopodan archosauriforms. Thus, these elements of the Panchet archosauriform assemblage are referred to chasmatosuchine proterosuchids.

Some of the cf. proterosuchid and proterosuchid specimens (e.g. quadrate, axis, humerus) differ morphologically from those of *Proterosuchus* spp. and ‘*Chasmatosaurus*’ *yuani*, supporting their assignment to a non-*Proterosuchus*/‘*Chasmatosaurus*’ proterosuchid archosauriform. As a result, there is no information against the assignment of these specimens to *Samsarasuchus pamelae*, but nor can it be supporting on the basis of autapomorphies. Information from articulated specimens would allow determining the referral of the cf. proterosuchid and proterosuchid bones of the Panchet Formation to the new species.

## 6. Description of cf. proterosuchid and proterosuchid specimens

### 6.1. Skull

#### 6.1.1. Maxilla

**Figure 18.**
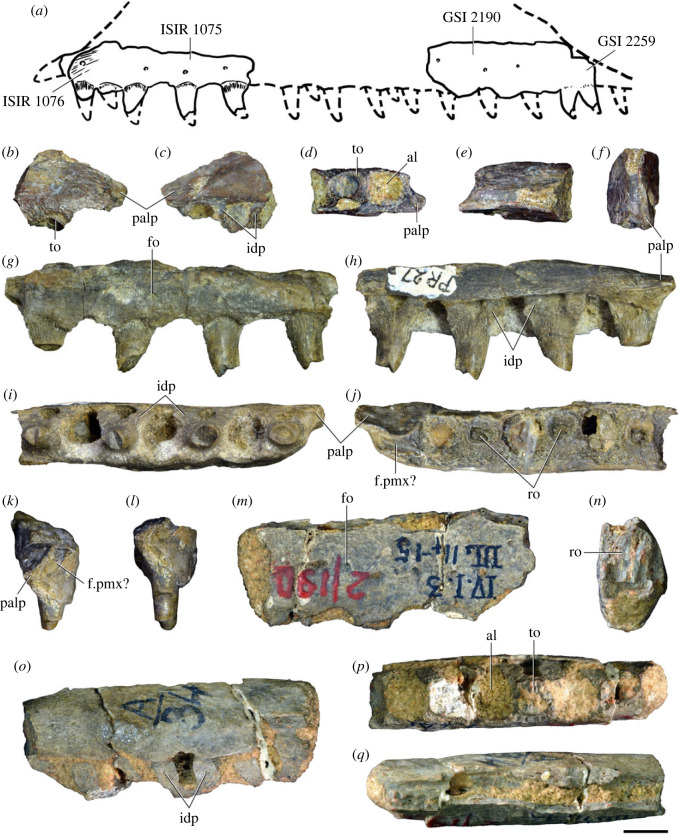
Panchet cf. proterosuchid maxillae. (*a*) Drawing of composite maxilla based on available specimens, (*b–f*) anterior end (ISIR 1076), (*g–l*) anteroventral portion (ISIR 1075), and (*m–q*) posteroventral portion (GSI 2190) of (*b–f, m–q*) right and (*g–l*) left maxillae in (*a, b, g, m*) lateral, (*c, h, o*) medial, (*d, i, p*) ventral, (*e, j, q*) dorsal, (*f, k, n*) anterior, and (*l*) posterior views. al, alveolus; f.pmx?, probable facet for premaxilla; fo, foramen; idp, interdental plate; palp, palatal process; ro, root; to, tooth. Scale bar equals 5 mm.

**Figure 19.**
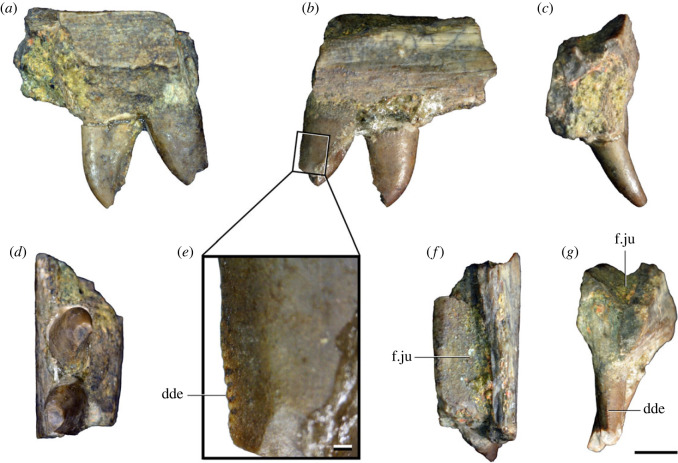
Panchet cf. proterosuchid partial left maxilla (GSI 2259, holotype of ‘*Ankistrodon indicus*’) in (*a*) lateral, (*b*) medial, (*c*) anterior, (*d*) ventral, (*f*) dorsal, and (*g*) posterior views. (*e*) Close-up of distal denticles in lingual view. dde, distal denticles; f.ju, facet for jugal. Scale bar equals 5 mm in (*a–d, f, g*) and 2 mm in (*e*).

This region of the skull is represented by four specimens, all of which preserve the alveolar margin of the bone (figure 18*a* and table 7). ISIR 1076 is the anterior tip of a right maxilla and preserves one tooth in the second tooth position, broken slightly apical to the base of the crown (figure 18*b–f* and table 7). ISIR 1075 is the ventral portion of the anterior process of a left maxilla and preserves seven tooth positions with four teeth *in situ* in the first, third, fifth and seventh alveoli (figure 18*g–l* and table 7). The crowns of these teeth lack their apices. The absence of a facet for articulation with the palatine in ISIR 1075 indicates that it should correspond mostly to the anterior portion of the maxilla. A small fragment of bone, bearing three tooth positions with two teeth preserved *in situ*, is the holotype of ‘*Ankistrodon indicus*’ (GSI 2259; figure 19 and table 7). This specimen represents a fragment of the posterior end of the horizontal process of a right maxilla. Finally, GSI 2190 represents a portion of the horizontal process of a right maxilla. It has seven tooth positions preserved, with three teeth *in situ* that are broken slightly apical to the base of the crown (figure 18*m–q*).

**Table 7.** Measurements in millimetres of Panchet cf. proterosuchid tooth-bearing bones. MxA (GSI 2190), MxB (GSI 2259), MxC (ISIR 1075), MxD (ISIR 1076), DtR (GSI 18123), and DtL (GSI 18124). DtL, left dentary; DtR, right dentary; Mx, maxilla. Values with an asterisk indicate incomplete measurements (owing to post-mortem damage) and the value given is the maximum measurable. The maximal deviation of the callipers is 0.02 mm, but measurements were rounded to the nearest 0.1 mm.

	MxA	MxB	MxC	MxD	DtR	DtL
length	34.0*	9.5*	38.7*	13.4*	33.5*	33.3*
maximum height	13.2*	5.3*	7.8*	8.9*	11.3*	14.1
minimum height	—	—	—	—	8.5*	12.6*
height of largest crown	—	4.7*	6.6*	—	8.9	—
length at base of largest crown	—	2.8	4.7	3.2	3.7	3.1

ISIR 1075 preserves the first seven alveoli and ISIR 1076 the first three, in which the third one is only partially preserved. The lateral surface of the anterior process is slightly anteroposteriorly and dorsoventrally convex and lacks ornamentation, but there is a series of primarily anteroventrally oriented striations on the lateral surface of the anterior tip of the process. There is a horizontal row of small, circular neurovascular foramina positioned approximately 4 mm dorsal to the alveolar margin (figure 18*g*). These foramina are present dorsal to the first, third, fifth and seventh tooth positions in ISIR 1075, which are also the alveoli that bear erupted teeth. The only foramen preserved in ISIR 1076 occurs dorsal to the second tooth position, which is also the only alveolus in this specimen to bear an erupted tooth. As a result, the different positions of the neurovascular foramina in the two specimens could result from the out-of-phase sequence of their tooth replacement (see below). The most anterior neurovascular foramen opens anterolaterally in both specimens, but is similar in size to more posterior foramina, contrasting with the proportionally larger anterior maxillary foramen commonly present in most Permo-Triassic non-archosauriform saurians (e.g. *Planocephalosaurus robinsonae*: [147]; *Protorosaurus speneri*: [30]; *Macrocnemus bassanii*: [148]; *Azendohsaurus madagaskarensis*: [149]; *Mesosuchus browni*: [150]; *Prolacerta broomi*: [151]) and some early archosauriforms (e.g. *Proterosuchus fergusi*: RC 846; *Osmolskina czatkoviensis*: [152]). However, it is possible that a larger anterior maxillary foramen was originally present dorsal to the preserved portion of the anterior process of both specimens. The more posterior foramina of the horizontal row open laterally to posteroventrally. There is at least one other foramen present dorsal to the horizontal row in ISIR 1075. This foramen is placed dorsal to the fifth tooth position and opens lateroventrally. None of the neurovascular foramina are confluent with ventrally extending grooves, contrasting with the condition in erythrosuchids (e.g. *Erythrosuchus africanus*, *Garjainia prima*; [18]).

The medial surface of the preserved portion of the anterior process is slightly concave anteroposteriorly at its anterior end and becomes anteroposteriorly convex more posteriorly (figure 18*c,h*). This surface possesses multiple primarily anteroposteriorly oriented thin striations. The base of the palatal process of the maxilla is preserved in both specimens and is placed immediately dorsal to the alveolar margin of the bone (figure 18: palp), as occurs in *Proterosuchus goweri* (NMQR 880), *Proterosuchus fergusi* (RC 846), ‘*Chasmatosaurus*’ *yuani* (IVPP V36315), *Kalisuchus rewanensis* (QMF8998), *Guchengosuchus shiguaiensis* [115], *Garjainia* spp. [153,154], *Chalishevia cothurnata* [155], *Euparkeria capensis* (SAM-PK-6048, SAM-PK-13666; [156]) and *Osmolskina czatkoviensis* [152]. This process projects only very weakly medially and, as a result, likely did not contact its counterpart along the midline of the palate, as is also the case in several non-archosaurian archosauriforms—a median contact between maxillae occurs at least in *Euparkeria capensis* [156]—and phytosaurs [9]. The base of the palatal process possesses a horizontally oriented main axis and two surfaces separated by a distinct change in slope. The first surface faces dorsomedially and is traversed by three to four thick longitudinal ridges. This surface would have received the palatal process of the premaxilla when in articulation. The second surface faces ventromedially, with a stronger ventral orientation in ISIR 1076. The palatal process and the lateral surface of the bone are separated dorsally by a transversely concave surface, which probably received the postnarial process of the premaxilla when in articulation. The alveolar margin of the anterior process of the maxilla is mainly straight in lateral view (figure 18), as in most early archosauriforms, but contrasting with the concave margin present in *Proterosuchus goweri* [49] and the upturned anterior end present in erythrosuchids (e.g. *Erythrosuchus africanus*, *Garjainia prima*; [18,141,154]).

The lateral surface of the horizontal process is slightly convex dorsoventrally and the medial surface is strongly convex dorsoventrally. As a result of this asymmetry, the horizontally oriented process becomes transversely narrower towards its dorsal margin. The lateral surface of GSI 2190 possesses a row of circular foramina positioned dorsal to the alveolar margin. These foramina open directly laterally and do not extend onto the lateral surface of the bone as grooves, similar to the condition in the anterior process of the bone. There is no facet for articulation with a palatal bone on the preserved medial surface of GSI 2190. The dorsal surfaces of GSI 2190 and GSI 2559 possess a mainly dorsally facing and gently transversely concave facet. This facet may have received the anterior process of the jugal (figure 19: f.ju). The medial border of this facet is raised dorsally above the lateral one in GSI 2259.

Tooth implantation is ankylothecodont in all the specimens, with bony ridges connecting the bone with the base of the crown (figure 18*a,g*), as in the maxillae of other proterosuchids [18] and *Prolacerta broomi* [151], *Teyujagua paradoxa* [157], *Tasmaniosaurus triassicus* [158], some early erythrosuchids (e.g. *Fugusuchus hejiapanensis*, *Guchengosuchus shiguaiensis*, some specimens of *Garjainia*; [43,115,154]) and *Kalisuchus rewanensis* [18]. There are interdental plates between each alveolus in ISIR 1075, ISIR 1076 and GSI 2190 (figure 18: idp), but they seem to be absent in GSI 2259. However, their absence in the latter specimen may be because of lack of preservation or variation of this feature along the alveolar margin, as is observed in some other proterosuchids (e.g. dentary of ‘*Chasmatosaurus*’ *yuani*: IVPP V36315). The interdental plates are pentagon-shaped in medial view, with a dorsoventral main axis, and are vertical in ISIR 1075 and slant gently posteriorly in GSI 2190. The interdental plates are anteroposteriorly short, leaving room between them for a large reabsorption pit on the lingual surface of the boundary between the crown and root. A reabsorption pit is well preserved in only one tooth position of GSI 2190, but pits are very well preserved in ISIR 1075 and the dentary GSI 18123 (see below), resembling the condition in ‘*Chasmatosaurus*’ *yuani* (IVPP V36315). The presence of interdental plates is an apomorphy of Archosauriformes, but convergently acquired within Tanystropheidae [9,18,57]. The Panchet cf. proterosuchid maxillae share with other proterosuchids the presence of interdental plates well separated from each other, whereas they closely approach or contact each other in the maxillae of *Kalisuchus rewanensis*, erythrosuchids, and eucrocopods [18]. The interdental plates extend dorsally to the same level as the lateral wall of the alveolar margin and possess multiple, small pits on their medial surface, which are more conspicuous in GSI 2190.

The broken surfaces of the available maxillae show that the teeth are deeply implanted in the alveoli (figure 18*j*,*n*: ro). The pattern of empty alveoli in GSI 2190, ISIR 1075 and ISIR 1076 (and also in two dentaries described below) strongly suggests an alternate tooth implantation. The concave lateral margins of the empty alveoli indicate that the replacement of the teeth involved the loss of part of the bony margin of the socket that was subsequently regenerated by growth of bone that later fused again to the base of the crown, as occurs in other archosauromorphs with ankylothecodont tooth implantation (e.g. *Proterosuchus fergusi*: RC 846). The interdental plates are also fused to the lingual surfaces of the teeth.

#### 6.1.2. Quadrate

**Figure 20.**
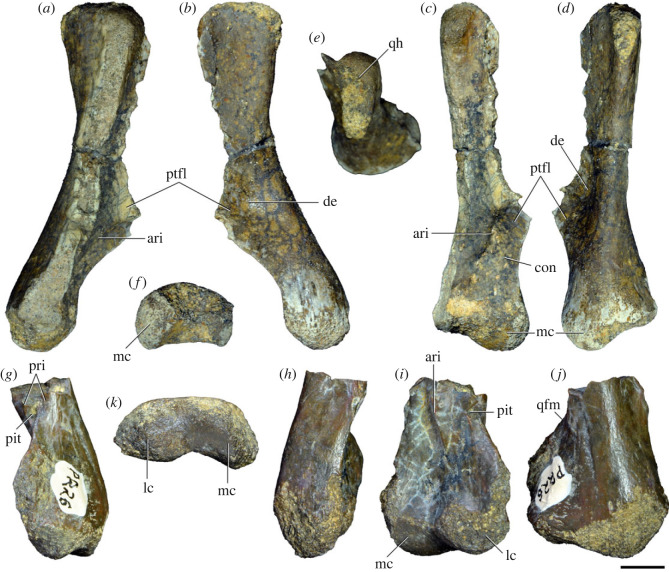
Panchet cf. proterosuchid (*g–k*) and proterosuchid (*a–f*) quadrates. (*a–f*) Right partial (ISIR 1077) and (*g–k*) left ventral region (ISIR 1078) of quadrates in (*a, g*) lateral, (*b, h*) medial, (*c, i*) anterior, (*d, j*) posterior, (*e*) dorsal, and (*f, k*) ventral views. ari, anterior ridge; con, concave surface; de, depression; lc, lateral condyle; mc medial condyle; pri, paired ridges; ptfl, pterygoid flange; qfm, quadrate foramen margin; qh, quadrate head. Scale bar equals 5 mm.

Two quadrates that are assigned to cf. Proterosuchidae (ISIR 1078) and Proterosuchidae (ISIR 1077) have been collected from the upper Panchet Formation (figure 20 and table 8). ISIR 1077 is a right quadrate that lacks most of its lateral surface, including part of the lateral ventral condyle (figure 20*a–f*). Most of the pterygoid flange is also missing and the quadrate head is damaged. ISIR 1078 is the ventral end of a left quadrate that has suffered damage to both the lateral edge and the ventral condyles (figure 20*g–k*). The morphologies of the two quadrates are consistent and they are described together.

**Table 8.** Measurements in millimetres of Panchet proterosuchid right quadrate (ISIR 1077) and cf. proterosuchid ventral end of left quadrate (ISIR 1078). Values with an asterisk indicate incomplete measurements (owing to post-mortem damage) and the value given is the maximum measurable. The maximal deviation of the callipers is 0.02 mm, but measurements were rounded to the nearest 0.1 mm.

	ISIR 1077	ISIR 1078
height	41.2	22.1*
proximal transverse width	5.2	—
proximal anteroposterior depth	9.6	—
distal transverse width	11.1*	18.5*
medial condyle transverse width	7.5	10.0*
medial condyle anteroposterior depth	7.5	8.7*

The quadrate is anteriorly bowed in lateral view, with an angle of 143° between the main axes of the dorsal and ventral ends of the bone. This angle resembles the condition in *Proterosuchus alexanderi* (angle: 149°), *Proterosuchus goweri* (angle: 149°) and ‘*Chasmatosaurus*’ *yuani* (angle: 155°), but differs from the considerably lower angles present in *Proterosuchus fergusi* (angle: 120°–127°) [49], *Sarmatosuchus otschevi* (angle: 121°, PIN 2865/68), *Erythrosuchus africanus* (angle: 128°, BP/1/5207) and *Euparkeria capensis* (angle: 129°, SAM-PK-6047a). An intermediate condition occurs in *Garjainia prima* (angle: 136°, PIN 2394/5). The articular surface of the quadrate head is convex. The quadrate has a semilunate outline in dorsal view, with an anteroposterior long axis and a slightly concave medial margin (figure 20*e*: qh), resembling the condition in *Garjainia prima* (PIN 951/57) and *Chalishevia cothurnata* [155]. By contrast, in *Sarmatosuchus otschevi* (PIN 2865/68) and *Erythrosuchus africanus* (NHMUK PV R3592) the quadrate head is subtriangular in dorsal view, with anteromedially, laterally and posteriorly oriented apices. The quadrate head becomes gradually transversely broader towards its anterior margin. The posterior surface of the proximal two-thirds of the bone is strongly convex transversely, but becomes less convex towards the ventral end as a result of transverse expansion of the bone. The medial surface of the proximal end of the bone is slightly concave anteroposteriorly.

The pterygoid flange extends along most of the anteromedial margin of the bone, contacting the margin of the dorsal articular surface and ending at the base of the medial ventral condyle (figure 20: ptfl). The medial surface of the base of the pterygoid flange possesses a depression close to its ventral margin (figure 20: de), as occurs in other early diverging archosauromorphs (e.g. *Proterosuchus goweri*: NMQR 880; *Sarmatosuchus otschevi*: PIN 2865/68; *Garjainia prima*: [154]). A low, thin ridge is present on the lateral surface of the base of the pterygoid flange and extends onto the anterior surface of the bone (figure 20*a,c,i*: ari). This ridge is anterodorsally-to-posteroventrally oriented, dorsally merges with the anteroventral margin of the pterygoid flange, and ventrally fades out gradually and does not reach the ventral end of the bone. The medial surface of the quadrate is flat close to its mid-length. The anterior surface of the quadrate is flat to slightly transversely convex on its dorsal two-thirds, with this surface being delimited medially by the pterygoid flange. The pterygoid flange is laminar and directed mainly anteriorly and slightly medially. The ventral edge of the pterygoid flange merges with the dorsal margin of the medial ventral condyle and it delimits with the anterior ridge a deeply concave transverse surface (figure 20*c*: con). This bifurcation also occurs in *Erythrosuchus africaus* (NHMUK PV R3592), but is absent in *Proterosuchus goweri* (NMQR 880), *Sarmatosuchus otschevi* (PIN 2865/68) and *Garjainia prima* (PIN 951/57). The medial wall of the quadrate foramen is preserved in ISIR 1078 (figure 20*j*: qfm) and possesses a ventrolaterally opening pit (figure 20*g*,*i*: pit), as occurs in *Sarmatosuchus otschevi* and *Erythrosuchus africanus* [139,141]. Only the ventral end of this pit is preserved, but it is well defined by a pair of thick vertical ridges (figure 20*g*: pri). The wall of the quadrate foramen ventral to the pit is formed by a sharp edge.

The ventral end of the bone possesses two articular condyles that participated in the cranio-mandibular joint (figure 20*f*,*k*: lc, mc). The orientation of the articular surface of the condyles indicates that the main axis of the quadrate was anterodorsally-to-posteroventrally oriented in lateral view, as occurs in other early diverging archosauromorphs (e.g. *Prolacerta broomi*: [151]; *Proterosuchus fergusi* and *Proterosuchus alexanderi*: [49]; ‘*Chasmatosaurus*’ *yuani*: IVPP V4067; *Erythrosuchus africanus*: [141]; *Euparkeria capensis*: [156]). The ventral end of the quadrate possesses a semilunate outline in distal view (figure 20*k*), with a concave anterior margin and a transverse main axis, as is the case in other early diverging archosauriforms (e.g. *Proterosuchus goweri*, NMQR 880; *Sarmatosuchus otschevi*: PIN 2865/68; *Garjainia prima*: [154]). The medial ventral condyle possesses a ball-like articular surface and is separated from the lateral ventral condyle by a deep cleft. The medial ventral condyle projects further ventrally than the lateral condyle, resembling the condition in *Proterosuchus goweri* (NMQR 880) and *Sarmatosuchus otschevi* (PIN 2865/68). The medial surface of the ventral end of the quadrate is anteroposteriorly convex. The posterior margin of the ventral end of the quadrate is dorsoventrally convex in lateral view, as occurs in several early diverging archosauromorphs (e.g. *Azendohsaurus madagaskarensis*: [159]; *Prolacerta broomi*: BP/1/471; *Proterosuchus goweri*: NMQR 880; *Proterosuchus alexanderi*: NMQR 1484). The ventral end of the quadrate is asymmetric in posterior view, being more expanded laterally than medially. The lateral surface of the distal end of the quadrate is not preserved in any specimen. As a consequence, the nature of the facet for articulation with the quadratojugal is unknown.

#### 6.1.3. Dentary

**Figure 21.**
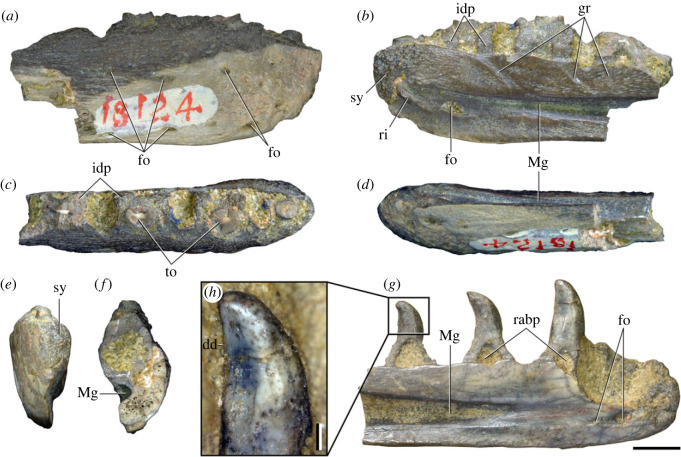
Panchet cf. proterosuchid dentaries. (*a–f*) Right (GSI 18124) and left (GSI 18123) anterior portions of dentaries in (*a*) lateral, (*b, g*) medial, (*c*) dorsal, (*d*) ventral, (*e*) anterior, and (*f*) posterior views. (*h*) Close-up of seventh dentary tooth in lingual view. dd, distal denticles; fo, foramen; gr, groove; idp, interdental plate; Mg, Meckelian groove; rabp, reabsorption pit; ri, ridge; sy, symphysis; to, tooth. Scale bar equals 5 mm in (*a–f, g*) and 1 mm in (*h*).

Anterior regions of a left and a right dentary are preserved and are similar in size and both broken off posteriorly at approximately the same point (GSI 18123, 18124; figure 21 and table 7). The exposed surfaces of the two dentaries are consistent in morphology. However, it is not possible to determine if they belong to the same individual because this information was not provided by Satsangi [45]. Nevertheless, the medial surfaces of the dentaries are slightly different from one another, being slightly striated on the right dentary (figure 21*b*) and smooth on the left dentary (figure 21*g*). The anterodorsal corner of the medial surface is missing in the left dentary and all the erupted crowns of the right dentary are broken off close to their bases. All surfaces of the right dentary are exposed, whereas the lateral surface of the left dentary is covered by matrix.

The anterior end of the dentary is distinctly transversely expanded, resembling the condition in ‘*Chasmatosaurus*’ *yuani* (IVPP V36315), *Proterosuchus alexanderi* (NMQR 1484), *Garjainia madiba* (NMQR 3051) and a dentary referred to *Archosaurus rossicus* (PIN 1100/78). By contrast, the dentary maintains a more similar transverse width along its length in *Prolacerta broomi* (BP/1/2675), *Teyujagua paradoxa* [157], *Sarmatosuchus otschevi* (PIN 2865/68-11), *Garjainia prima* [154], *Erythrosuchus africanus* [141] and *Euparkeria capensis* (SAM-PK-5867, SAM-PK-6050). The lateral surface of the preserved portion of the bone is dorsoventrally and anteroposteriorly convex and possesses large neurovascular foramina that are aligned in two longitudinal rows (figure 21*a*: fo), as occurs in *Prolacerta broomi*, *Proterosuchus fergusi*, *Proterosuchus alexanderi*, *‘Chasmatosaurus*’ *yuani*, *Sarmatosuchus otschevi* and the specimen referred to *Archosaurus rossicus* [18]. The more dorsal row is located approximately at mid-height of the anterior end of the bone and contains six preserved foramina, with the four more anterior being sub-circular and opening mainly laterally, whereas the two posterior foramina are larger, sub-oval, open posterolaterally and extend posteriorly as a short, shallow groove. Six openings are also preserved in the lower row of foramina, with the two more anterior foramina being larger than the more posterior ones. The foramina of the more ventral row open lateroventrally. The medial surface of the dentary possesses a Meckelian groove that deepens posteriorly and is positioned just ventral to the mid-height of the bone (figure 21: Mg). The Meckelian groove does not reach the symphysis and at its anterior end is clearly delimited by a low anterodorsally-to-posteroventrally oriented ridge (figure 21*b*: ri). Immediately posterior to this ridge and within the Meckelian groove, there is a large sub-oval foramen with an anteroposteriorly oriented main axis (figure 21*b,g*: fo), as in *'Chasmatosaurus*’ *yuani* (IVPP V36315), *Garjainia prima* [154], *Erythrosuchus africanus* [141] and the referred specimen of *Archosaurus rossicus* (PIN 1100/78). The foramen of *Erythrosuchus africanus* is positioned more anteriorly than in GSI 18123, GSI 18124 and the previously mentioned taxa, being placed at the level of the second tooth position (NHMUK PV R3582). This foramen in GSI 18123 and GSI 18124 extends posteriorly as a short, shallow groove parallel to the ventral margin of the Meckelian groove. There is a second, considerably smaller, foramen situated posterior to the large medial foramen in the left dentary only (figure 21*g*). The symphysis is restricted to the anteriormost end of the bone and faces mainly anteromedially, with a rugose surface (figure 21*b*: sy). This morphology corresponds to the type II morphotype of Holliday & Nesbitt [159] and is also present in other proterosuchids (‘*Chasmatosaurus*’ *yuani*: IVPP V36315; referred specimen of *Archosaurus rossicus*: PIN 1100/78). The medial surface of the bone dorsal to the Meckelian groove is slightly anteroposteriorly convex anteriorly, becoming flat posteriorly. There is a group of very shallow anterodorsally-to-posteroventrally oriented grooves on the medial surface of the bone dorsal to the Meckelian groove (figure 21*b*: gr). There are at least three of these grooves on the right dentary and two on the left, but one additional anterior groove may be missing in the broken area of the latter bone. Similar diagonal grooves dorsal to the Meckelian groove are also present in ‘*Chasmatosaurus*’ *yuani* (IVPP V36315), *Erythrosuchus africanus* [141], *Garjainia prima* (PIN 951/54), and the referred specimen of *Archosaurus rossicus* (PIN 1100/78). The medial surface of the dentary ventral to the Meckelian groove faces laterally at the anterior end of the bone, but gradually changes posteriorly to face dorsomedially. The ventral margin of the bone has a sharp medial edge that would have articulated with the ventral margin of the splenial.

There are eight tooth positions preserved in the right dentary and there is potentially room for the same number in the left dentary, but only seven alveoli are clearly exposed in the latter bone. The alveoli face dorsally and the tooth implantation is ankylothecodont, with thin bony ridges linking the tooth with the tooth-bearing bone, as occurs in other proterosuchids and some other early archosauriforms [9,18]. The first alveolus is set back from the anterior margin of the bone by an edentulous region, which is however shorter than the length of the subsequent tooth positions. At least five distinct interdental plates are preserved medial to tooth positions 2–7. The morphology of the interdental plates is identical to those of the maxilla. Teeth are erupted in the same alternate pattern in both dentaries, suggesting the presence of an alternate tooth replacement through, at least, the ontogenetic stages documented by these specimens and the larger portion of maxilla (GSI 2190).

#### 6.1.4. Dentition

Teeth are preserved in six specimens, four partial maxillae (GSI 2190, 2259, ISIR 1075, ISIR 1076; figures 18 and 19 and table 7) and two anterior ends of dentaries (GSI 18123, 18124; figure 21 and table 7). The bases of the crowns exposed in cross-section in the maxillae are slightly labiolingually compressed. Most of the preserved maxillary crowns of GSI 2259 and ISIR 1075 are fairly complete but lack their apices. The mesial margin is strongly convex in labial view in all the preserved crowns. The distal margin of the crowns is concave in the mesial maxillary teeth of ISIR 1075 and mesial dentary teeth of GSI 18123 (figures 18 and 21), but straight in the distal maxillary teeth of GSI 2259 (figure 19). The presence of straight to incipiently recurved distal maxillary teeth occurs in juvenile specimens of *Proterosuchus fergusi* [49]. All these maxillary and dentary teeth preserved *in situ* in their tooth-bearing bone lack carinae and denticles on their preserved mesial margins, but the tip of all the crowns is missing, whereas the distal margin possesses a serrated carina (figures 19*e* and 21*h*). An isolated tooth crown from the upper Panchet Formation, originally described as the new species *Teratosaurus*(?) *bengalensis*, preserves its tip and has mesial denticles, smaller than the distal ones, restricted to the upper third of the crown [86]. This latter condition is the same that occurs in *Proterosuchus* (e.g. *Proterosuchus fergusi*: SAM-PK-11208), ‘*Chasmatosaurus*’ *yuani* (IVPP V36315), and a dentary that has been referred to *Chasmatosuchus rossicus* (PIN 2354/26; [40]). Thus, *Teratosaurus*(?) *bengalensis* and the other Panchet cf. proterosuchid tooth-bearing bones may belong to a single species and the apparent absence of mesial denticles could be a result of lack of preservation. By contrast, *Teyujagua paradoxa* lacks denticles on the mesial margin of the crowns [57,157]. One of the isolated tooth crowns referred to as *Archosaurus rossicus* (PIN 1100/85a) has small, poorly mesiodistally developed denticles that extend through at least more than the apical half of both mesial and distal margins. The best-preserved tooth crown of the dentary of *Sarmatosuchus otschevi* has mesial denticles as large as the distal ones, but it cannot be determined how far they extend basally (PIN 2865/68-11). Similarly, an erupting tooth in the maxilla of *Kalisuchus rewanensis* has mesial denticles but it cannot be determined how far basally they extend on the crown (QMF8998). In most erythrosuchids (e.g. *Guchengosuchus shiguaiensis*: [115]; *Garjainia prima*: [43,154]; *Erythrosuchus africanus*: [141]; *Chalishevia cothurnata*: [155]) and early eucrocopods (e.g. *Euparkeria capensis*: [156]; *Rhadinosuchus gracilis*: [160]) the mesial denticles extend basally to the apical third of the crown and are similar or slightly less mesiodistally developed that the distal denticles. The denticles of the Panchet cf. proterosuchid tooth-bearing bones are very small, eight serrations per millimetre in GSI 2259, with a rounded margin and do not reach the base of the crown on the distal edge. The preserved regions of the crowns lack ornamentation or wear facets, contrasting with the presence of enamel wrinkles in the erythrosuchid *Chalishevia cothurnata* [155].

The bases of the crowns of the first and third tooth positions of the dentaries are circular in cross-section, but more distal crowns are labiolingually compressed at their bases, as is the case in *Prolacerta broomi*, *Teyujagua paradoxa*, *Tasmaniosaurus triassicus*, and early diverging archosauriforms [9,18,57,158]. The labiolingual compression of the crowns increases toward their apices. There is no mesiodistal or labiolingual expansion at the base of the crown. The tooth crown of the third tooth position of the left dentary is only slightly distally curved, showing a distinctly convex mesial margin but an almost straight distal margin. The mesial margin of this third crown is more labiolingually convex than that of the more distal crowns. The crowns of the fifth and seventh tooth positions of this same dentary are distinctly distally curved, with a concave distal margin. The pattern of serrations in the crowns is very similar to that of the maxillary teeth, with seven serrations per millimetre in the third dentary tooth of GSI 18123. The lingual surface of the crown lacks ornamentation or wear facets on the surface of the enamel.

### 6.2. Cervical vertebrae

#### 6.2.1. Axis

**Figure 22.**
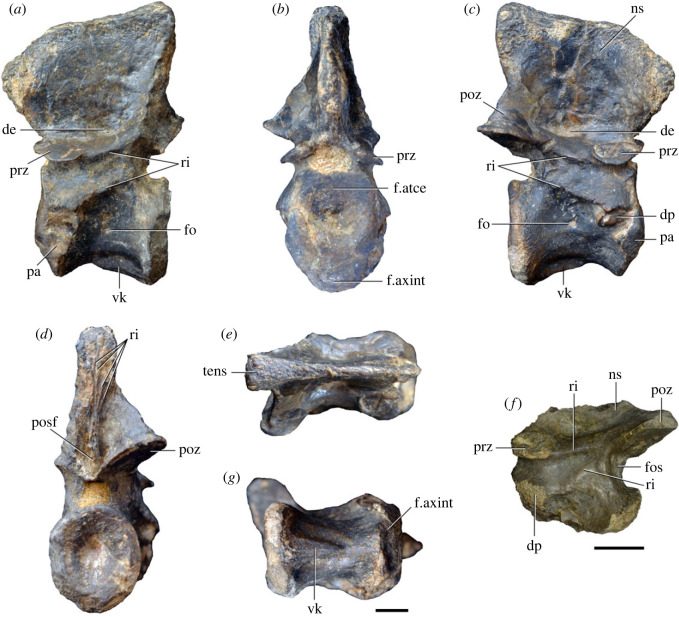
Panchet cf. proterosuchid axes. (*a–e, g*) Almost complete (NHMUK PV R37582) and (*f*) partial (ISIR 1079) left axes in (*a, f*) left lateral, (*c*) right lateral, (*b*) anterior, (*d*) posterior, (*e*) dorsal, and (*g*) ventral views. de, depression; dp, diapophysis; f.atce, facet for atlantal centrum; f.axint, facet for axial intercentrum; fo, foramen; fos, fossa; ns, neural spine; pa, parapophysis; posf, postspinal fossa; poz, postzygapophysis; prz, prezygapophysis; ri ridge; tens, transverse expansion of neural spine; vk, ventral keel. Scale bar equals 1 cm.

The axes are represented by a fairly complete element, lacking only the left postzygapophysis (NHMUK PV R37582; figure 22*a–e,g* and table 2), and a less complete element that lacks the right zygapophyses and most of its centrum, right diapophysis and neural spine (ISIR 1079; figure 22*f* and table 2). The centrum of the axis is 1.25 times longer than the height of its posterior articular surface. The centrum is moderately transversely compressed around mid-length and the anterior and posterior articular surfaces are concave. The anteroventral edge of the centrum is distinctly bevelled and possesses a broad semilunate facet for reception of the unfused axial intercentrum (figure 22*b*: f.axint). The atlantal centrum (=odontoid process) is also not fused to the axial centrum. The posterior articular surface is approximately 1.2 times higher than broad. The ventral surface of the centrum has a well-developed longitudinal keel restricted to its posterior two-thirds (figure 22: vk). This keel is subtriangular in ventral view, being considerably broader posteriorly, and has a straight ventral margin in lateral view. The ventral keel and the lateral surface of the centrum are separated by a distinct, dorsally arched change in slope. The lateral surface of the centrum possesses a low parapophysis on its anteroventral corner (figure 22*a*,*c*: pa) and a more laterally developed diapophysis on its anterodorsal corner (figure 22*c*: dp). The diapophysis is anterolateroventrally directed and positioned slightly posterior to the parapophysis. A conspicuous ridge extends posterodorsally from the diapophysis towards the posteroventral corner of the peduncle of the neural arch (figure 22*a*,*c*: ri). This ridge is mound-like in ISIR 1079, but better developed and with a sharper edge in NHMUK PV R37582. The lateral surface of the centrum ventral to this ridge is concave and has a relatively large, sub-oval foramen, with an anteroposterior main axis, placed approximately centrally on both sides of NHMUK PV R37582 (figure 22*a*,*c*: fo). This lateral surface harbours four small, oval foramina on the right side of ISIR 1079; the condition on the left side of this specimen cannot be determined because of damage. The neurocentral suture is closed in both available axes.

**Figure 23.**
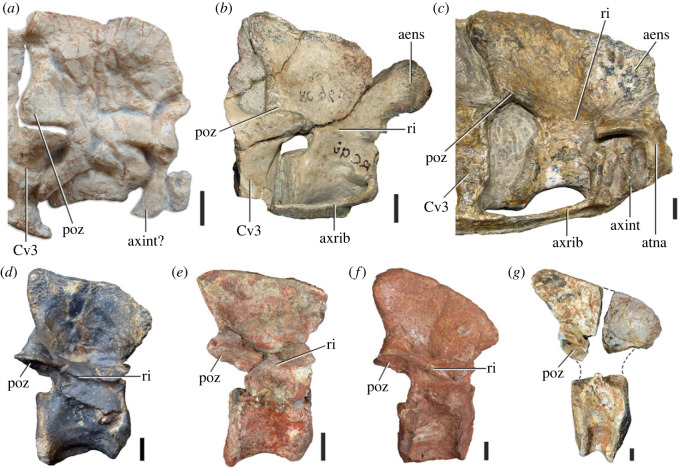
Comparison between axes of selected Early to Middle Triassic non-eucrocopod crocopods. (*a*) *Teyujagua paradoxa* (modified and reversed from Pinheiro *et al.* [157], holotype), (*b*) *Proterosuchus fergusi* (RC 846, proposed neotype, reversed), (*c*) *Proterosuchus fergusi* (SNSB-BSPG 1934 VIII 514, reversed), (*d*) Panchet cf. proterosuchid specimen (NHMUK PV R37582), (*e*) *Sarmatosuchus otschevi* (PIN 2865/68-22, holotype, reversed), (*f*) *Garjainia prima* (PIN 2394/5-10, holotype), and (*g*) *Bharitalasuchus tapani* (ISIR 1156, 1158, 1158A, holotype) in lateral view. aens, anterior extension of the neural spine; atna, atlantal neural arch; axint, axial intercentrum; axrib, axial rib; Cv3, third cervical vertebra; poz, postzygapophysis; ri, ridge. Scale bars equal 2 mm in (*a*) and 1 cm in (*b–g*).

In the neural arch, the prezygapophysis is very short, projects anteriorly and is positioned dorsal to the level of the diapophysis (figure 22: prz). The base of the prezygapophysis extends posteriorly as a thick, posterodorsally oriented ridge that reaches the base of the postzygapophysis in NHMUK PV R37582 but fails to contact the postzygapophysis in ISIR 1079 (figure 22*a,c,f*: ri). This ridge forms a distinct shelf lateral to the base of the neural spine and resembles the condition in *Proterosuchus fergusi* (RC 846, SNSB-BSPG 1934 VIII 514), *Garjainia prima* [43], *Sarmatosuchus otschevi* (PIN 2865/68) and a specimen referred to as *Tsylmosuchus jakovlevi* (PIN 4339/1) (figure 23*b,c,e,f*). The lateral surface between the prezygapophysis and diapophysis is anteroposteriorly convex and the surface between the ridges that project posteriorly from both structures is gently concave. The postzygapophysis is considerably longer than the prezygapophysis and extends posterior to the posterior border of the centrum (figure 22: poz), as occurs in the axis of other early archosauriforms (e.g. *Proterosuchus fergusi*: RC 846; *Sarmatosuchus otschevi*: PIN 2865/68). The postzygapophysis is posterolaterally oriented at an angle of 50° with respect to the sagittal axis of the neck. The postzygapophysis is also slightly dorsally oriented, being positioned immediately dorsal to the level of the prezygapophysis. The articular facet of the postzygapophysis is oval, faces lateroventrally, and is well defined anteriorly by a ridge. The ventral surface of the base of postzygapophysis is covered by a moderately deep fossa that extends onto the posterolateral surface of the neural arch (figure 22*f*: fos). This surface possesses multiple, mainly dorsoventrally oriented striations that extend onto the posterior surface of the neural arch immediately lateral to the neural canal. The base of the postzygapophysis extends medially as a short shelf, but it does not contact its counterpart at the midline. There is no hyposphene. The dorsal surface of the postzygapophysis lacks an epipophysis. The posterior margin of the neural arch has a sharp inflexion between the postzygapophysis and neural spine in lateral view. This as a result of the strong posterior development of the postzygapophysis, as occurs in *Prolacerta broomi* (BP/1/2675), *Teyujagua paradoxa* [157], *Sarmatosuchus otschevi* [139], *Garjainia prima* [43,140], and specimen referred to as *Tsylmosuchus jakovlevi* (PIN 4339/1) (figure 23*a,d–f*). By contrast, the postzygapophysis is poorly posteriorly developed and confluent with the posterior margin of the neural spine in dorsal view in *Proterosuchus fergusi* (RC 846, SNSB-BSPG 1934 VIII 514), *Proterosuchus alexanderi* (NMQR 1484), *Shansisuchus shansisuchus* [146], *Bharitalasuchus tapani* [102] and *Euparkeria capensis* (SAM-PK-5867) (figure 23*b,c,g*).

The base of the neural spine of the axis is transversely thick and extends along the entire midline of the neural arch (figure 22: ns). There is a depression present on the point of transition between the base of the neural spine and the lateral surface of the neural arch in both NHMUK PV R37582 and ISIR 1079 (figure 22*a,c*: de). The neural spine is taller than the centrum and blade-like, with an anteroventrally sloping dorsal margin in lateral view. This margin is formed by two straight edges separated by a distinct inflexion point at the anterior third of the spine, a condition that contrasts with the continuously straight or slightly convex dorsal margin of the axial neural spine in *Proterosuchus fergusi* (SNSB-BSPG 1934 VIII 514), *Sarmatosuchus otschevi* [139] and *Garjainia prima* [43] (figure 23). In dorsal view, the posterior half of the neural spine of NHMUK PV R37582 expands gradually transversely, acquiring a subtriangular profile, whereas the anterior region of the neural spine is slightly thicker than at mid-length (figure 22*e*). The anterior portion of the neural spine is subtriangular in lateral view, with a distinct anteriorly directed apex, as is the case in most early archosauriforms. By contrast, the anterior end of the neural spine is more strongly anteriorly projected and rounded in lateral view in *Proterosuchus fergusi* (RC 846, SNSB-BSPG 1934 VIII 514; figure 23*b,c*), but the condition is unknown in other species of *Proterosuchus*. Thus, this condition may represent an autapomorphy of *Proterosuchus fergusi* or an apomorphy at some level within Proterosuchidae. The axes have a deep postspinal fossa between the postzygapophyses, but restricted to the base of the neural spine (figure 22*d*: posf). The posterior surface of the neural spine possesses a series of longitudinal ridges, which diverge from each other at the distal end of the spine, and probably acted as the anchor of interspinosous ligaments (figure 22*d*: ri). The posterodorsal corner of the neural spine does not extend posteriorly beyond the level of the postzygapophysis in lateral view, resembling similarly short posterior developments of the neural spines of *Prolacerta broomi* (BP/1/2675), *Teyujagua paradoxa* [157], *Proterosuchus alexanderi* (NMQR 1484), *Sarmatosuchus otschevi* [139], *Garjainia prima* [43,140], a specimen referred to as *Tsylmosuchus jakovlevi* (PIN 4339/1) and *Euparkeria capensis* (SAM-PK-5867). By contrast, the neural spine of the axis extends posteriorly beyond the postzygapophyses for a distance equal to or longer than the length of the latter structures in *Proterosuchus fergusi* (RC 846, SNSB-BSPG 1934 VIII 514), *Shansisuchus shansisuchus* [146] and *Bharitalasuchus tapani* [102] (figure 23*b,c,g*).

### 6.3. Dorsal vertebrae

#### 6.3.1. Posterior dorsal vertebrae (probable D12–D16)

**Figure 24.**
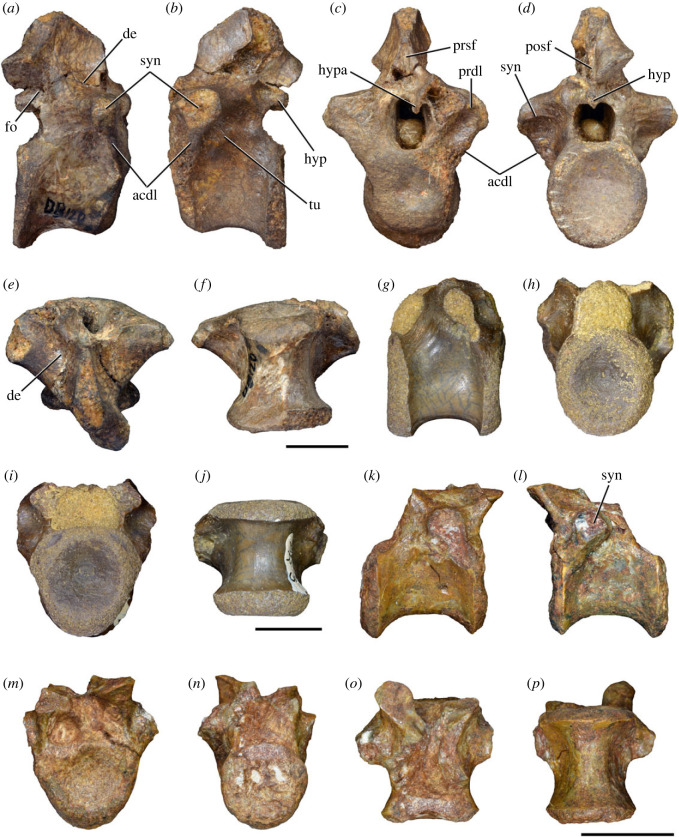
Panchet cf. proterosuchid posterior dorsal (probable twelveth to sixteenth) vertebrae. (*a–f*) NHMUK PV R37586, (*g–j*) ISIR 1105, and (*k–p*) ISIR 1107 in (*a, g, k*) right lateral, (*b, l*) left lateral, (*c, h, m*) anterior, (*d, i, n*) posterior, (*e, o*) dorsal, and (*f, j, p*) ventral views. acdl, anterior centrodiapophyseal lamina; de, depression; fo, fossa; hyp, hyposphene; hypa, hypantrum; posf, postspinal fossa; prdl, prezygodiapophyseal lamina; prsf, prespinal fossa; syn, synapophysis; tu, tuberosity. Scale bars equal 1 cm.

**Figure 25.**
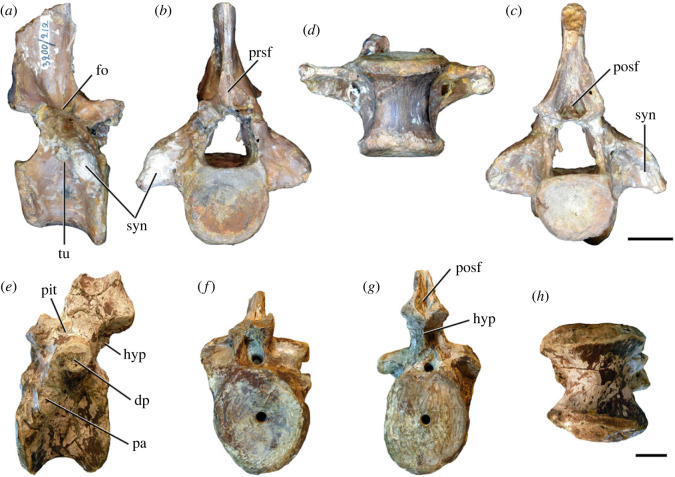
Comparison between posterior dorsal vertebrae of selected Early and Middle Triassic non-eucrocopodan archosauriforms. (*a–d*) *Vonhuenia friedrichi* (PIN 3200/212) and (*e–h*) *Erythrosuchus africanus* (NHMUK PV R3592) in right lateral (*a*), anterior (*b,f*), posterior (*c,g*), ventral (*d,h*), and left lateral (*e*) views. Abbreviations: dp, diapophysis; fo, fossa; hyp, hyposphene; pa, parapophysis; posf, postspinal fossa; prsf, prespinal fossa; syn, synapophysis; tu, tuberosity. Scale bars equal 1 cm in (*a–d*) and 2 cm in (*e–h*).

This region of the trunk is represented by two more complete vertebrae and four additional specimens mainly represented by their centra (figure 24 and table 9). NHMUK PV R37586 is the most complete posterior dorsal vertebra, lacking the distal ends of the transverse processes and neural spine, right prezygapophysis, most of the left prezygapophysis and left postzygapophysis (figure 24*a*–*f*). In addition, the ventral margin of the anterior articular surface of the centrum is damaged. ISIR 1107 lacks the distal end of the right prezygapophysis, both postzygapophyses, neural spine and most of the transverse processes (figure 24*k*–*p*). Additional posterior dorsal vertebrae that are represented by an isolated centrum and, in some cases, a portion of the base of the neural canal are ISIR 1105 (figure 24*g*–*j*), ISIR 1106, NHMUK PV R37584 and ISIR 1112. The description of the posterior dorsal vertebrae is mostly based on NHMUK PV R37586, which has a consistent morphology with the other posterior dorsal vertebrae. Isolated vertebrae that have been referred to as *Chasmatosuchus rossicus* (PIN 3200/212; [40]; figure 25*a*–*d*) and cf. *Proterosuchus* from Brazil [110] resemble the above-mentioned specimens, including the presence of a synapophysis, and very probably represent posterior dorsal elements; thus, they are compared with the Panchet cf. proterosuchid posterior dorsal vertebrae.

**Table 9.** Measurements in millimetres of Panchet cf. proterosuchid posterior dorsal vertebrae. PDa (NHMUK PV R37586), PDb (ISIR 1112), PDc (ISIR 1105), PDd (ISIR 1106), and PDe (ISIR 1107). Abbreviation: PD, posterior dorsal vertebra. Values with an asterisk indicate incomplete measurements (owing to post-mortem damage) and the value given is the maximum measurable. The maximal deviation of the callipers is 0.02 mm, but measurements were rounded to the nearest 0.1 mm.

	PDa	PDb	PDc	PDd	PDe
length of centrum	18.0	25.2	18.0	13.8	13.2
anterior height of centrum	13.4*	23.0	16.3*	11.3*	10.7
anterior width of centrum	12.9	23.4	16.7*	12.2	10.3
posterior height of centrum	16.4	22.3*	15.9*	12.9	9.6*
posterior width of centrum	15.4	[24.6]	14.9*	12.5	10.7
length across zygapophyses	20.7*	—	—	—	—
height neural spine	12.5*	—	—	—	—
length neural spine at base	14.3	—	—	—	—
maximum height	37.9*	—	25.3*	15.0*	19.8*

The length of the centrum is lower than 1.35 times the height of the damaged anterior articular surface in NHMUK PV R37586 (figure 24*a*,*b*), 1.23 times in ISIR 1107 (figure 24*k*,*l*), and approximately 1.10 times in ISIR 1112 and ISIR 1105 (figure 24*g*). This ratio falls within the range in the referred specimen of *Chasmatosuchus rossicus* (1.16: PIN 3200/212; figure 25*a*), *Garjainia prima* (0.90–1.30: PIN 951/64) and *Cuyosuchus huenei* (1.21: MCNAM PV 2669), resembles the ratio present in the cf. *Proterosuchus* from Brazil (1.37: UNIPAMPA 684; [110]), but is slightly higher than that of *Erythrosuchus africanus* (0.79–0.92: NHMUK PV R3592, SAM-PK-905; figure 25*e*) and *Shansisuchus shansisuchus* (1.00–1.06: Young [146]: table 6 presacrals 24 and 25). The centrum is very slightly parallelogram-shaped in lateral view, with the anterior articular surface offset dorsally to the posterior one. Both anterior and posterior articular surfaces of the centrum are shallowly concave and slightly dorsoventrally taller than broad. The notochordal pit is circular, shallow and slightly displaced dorsally from the centre of the centrum. The centrum is slightly constricted transversely at mid-length and, as a result, is spool-shaped in ventral view. The ventral surface of the centrum lacks a median keel (ISIR 1107, ISIR 1112, ISIR 1105) and its lateral surface has a very shallow and poorly rimmed lateral fossa, as is the case in the referred specimen of *Chasmatosuchus rossicus* (PIN 3200/212) and posterior dorsal vertebrae of *Garjainia prima* [140], *Erythrosuchus africanus* (NHMUK PV R3592) and *Cuyosuchus huenei* (MCNAM PV 2669). By contrast, the cf. *Proterosuchus* from Brazil has a ventral median keel [110]. The neurocentral suture is closed in ISIR 1107, NHMUK PV R37586 and ISIR 1105, but is open in ISIR 1112.

The parapophysis and diapophysis are merged into a single synapophysis, but its distal articular surface is not preserved on either side of ISIR 1107, NHMUK PV R37586 or ISIR 1105. As a result, it cannot be determined if one of the more posterior dorsal ribs was fused to its respective vertebra, which is a condition that occurs in some archosauromorphs (e.g. *Azendohsaurus madagaskarensis*: [17]; *Proterosuchus alexanderi*: NMQR 1484; *Garjainia prima*: [140]). A similar dorsoventrally tall synapophysis with a poor distinction between parapophyseal and diapophyseal regions occurs in the referred specimen of *Chasmatosuchus rossicus* (PIN 3200/212; figure 25*a*: syn) and the posterior dorsal vertebrae of *Cuyosuchus huenei* (MCNAM PV 2669). By contrast, the posterior dorsal vertebrae of erythrosuchids (e.g. *Erythrosuchus africanus*: NHMUK PV R3592, figure 25*e*; *Garjainia prima*: PIN 951/64; *Shansisuchus shansisuchus*: Young [146]: figure 22*b*) and most eucrocopods retain a clear distinction between both regions, with separate articular facets or a single facet with an L-shaped profile [18]. The transverse process is placed on the anterior half of the neural arch and level with the dorsal half of the neural canal, but in *Erythrosuchus africanus* the dorsal region of the synapophysis is placed posteriorly to the mid-length of the centrum (NHMUK PV R3592, figure 25*e*). There is a well-developed anterior centrodiapophyseal lamina that extends onto the anterodorsal corner of the centrum (figure 24*a*,*b*: acdl). This lamina anteriorly delimits a moderately deep centrodiapophyseal fossa. A thick, low and anteroventrally-to-posterodorsally oriented tuberosity is present ventral to the transverse process in NHMUK PV R37586 (figure 24*b*: tub), resembling the condition in more anterior dorsal vertebrae and the posterior dorsal vertebra referred to *Chasmatosuchus rossicus* (PIN 3200/212; figure 25*a*: tu). In NHMUK PV R37586, this tuberosity delimits posteroventrally the centrodiapophyseal fossa, but it is absent in ISIR 1107. There is a short prezygodiapophyseal lamina that is only preserved on the left side of NHMUK PV R37586 (figure 24*c*: prdl) and is incipient in ISIR 1105, but absent in ISIR 1107. The absence of the lateral tuberosity and a prezygodiapophyseal lamina probably indicates that ISIR 1107 is a more posterior element than NHMUK PV R37586 and ISIR 1105. An intermediate condition occurs in the posterior dorsal vertebra referred to *Chasmatosuchus rossicus*, in which there is a diagonal tuberosity but no laminae on the neural arch (PIN 3200/212). The posterior centrodiapophyseal and postzygodiapophyseal laminae are absent in all the posterior dorsal vertebrae of the Panchet cf. proterosuchids.

The postzygapophysis extends posteriorly slightly beyond the level of the posterior margin of the centrum and possesses a posterolaterally oriented main axis. There is no epipophysis and the postzygapophyseal facet is oval and faces lateroventrally. There is a sub-circular and moderately deep fossa immediately ventral to the base of the postzygapophysis (figure 24*a*: fo). In NHMUK PV R37586, a well-developed and distinct hyposphene and hypantrum are preserved immediately ventral to the base of the prezygapophyses and postzygapophyses, respectively (figure 24*b*–*d*: hyp, hypa), contrasting with the condition in more anterior presacral vertebrae. These accessory articular structures are also present in the posterior dorsal vertebrae of *Erythrosuchus africanus* (NHMUK PV R3592). The hyposphene is represented by a diamond-shaped structure in posterior view that possesses a short articular surface on its dorsolateral margin. The ventral margin of the hyposphene extends onto the roof of the neural canal as a sharp and narrow median longitudinal flange. The hypantrum possesses a very short, peg-like process on its medial surface to articulate with the hyposphene of the preceding vertebra. However, a hypantrum is absent in ISIR 1107, resembling the condition in the posterior dorsal vertebra referred to *Chasmatosuchus rossicus* (PIN 3200/212). There is a very shallow, concave depression on the lateral surface of the base of the neural spine (figure 24*a*,*e*: de), contrasting with the deeper fossa or pit present in this region in the posterior dorsal vertebra referred to *Chasmatosuchus rossicus* (PIN 3200/212; figure 25*a*: fo), *Garjainia prima* [140], and *Erythrosuchus africanus* ([141]; figure 25*e*: pit). As far as it is preserved, the neural spine is mainly dorsally oriented in lateral view. There is a moderately deep prespinal fossa that is restricted to the base of the neural spine. The postspinal fossa is very deep and its distal end is not preserved. The neural spine is subtriangular in cross-section, with an anterior apex, where it is broken in NHMUK PV R37586.

### 6.4. Sacral vertebrae

There are two different morphologies of sacral vertebrae and ribs in the early archosauriform assemblage from the Panchet Formation and the medial surface of the ilium with a complete iliac blade (NHMUK PV R10149) possesses only two scars for the attachment of sacral ribs. As a result, it is interpreted that the sacrum of the Panchet cf. proterosuchid taxon was composed of only two vertebrae, as occurs in other non-archosaurian archosauromorphs [9,18].

#### 6.4.1. First sacral vertebra and rib

**Figure 26.**
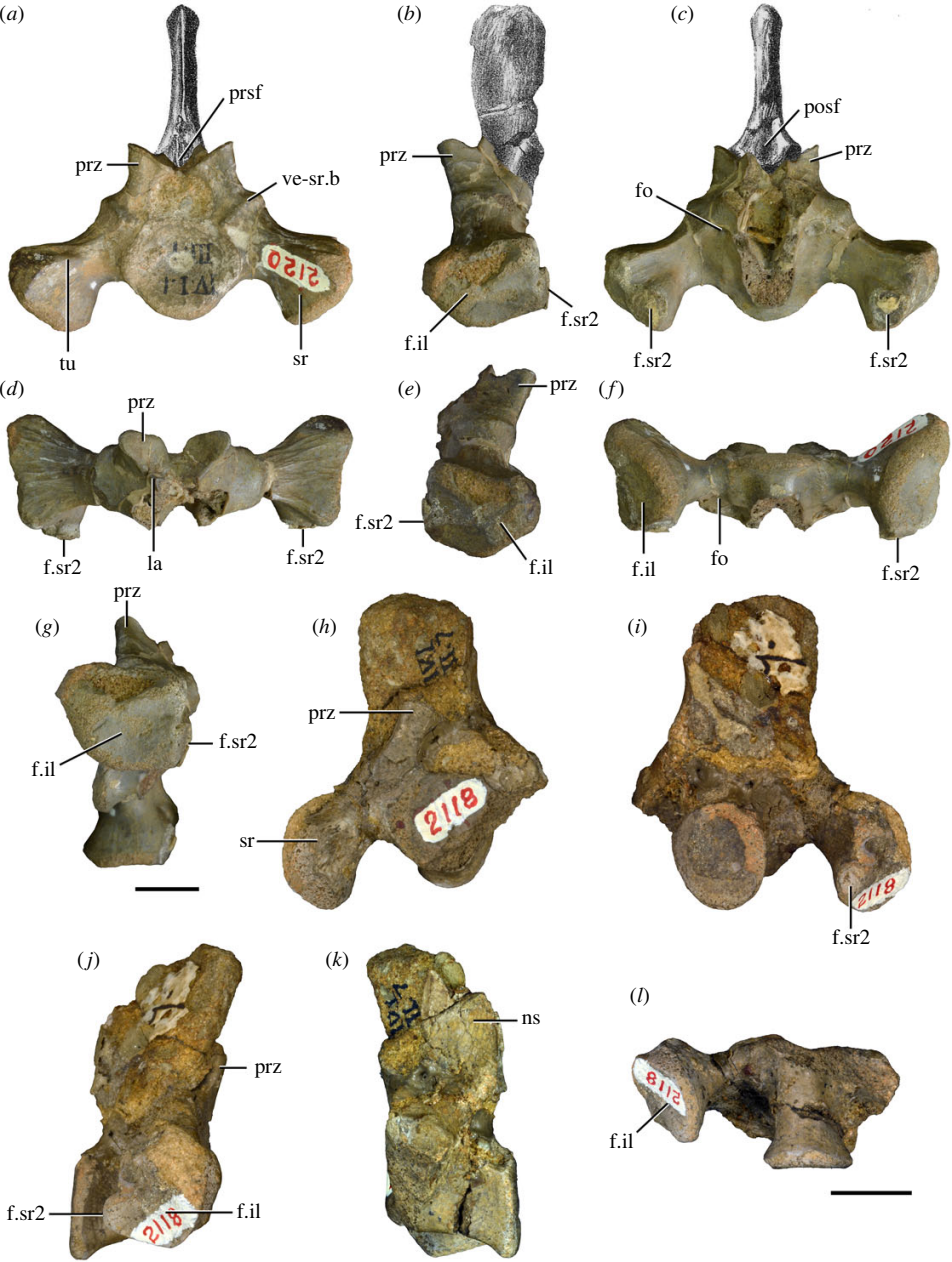
Panchet cf. proterosuchid first sacral vertebrae and ribs. (*a–g*) GSI 2120 and (*h–l*) GSI 2128 in (*a, h*) anterior, (*b, k*) left lateral, (*c, i*) posterior, (*d*) dorsal, (*e, j*) right lateral, (*f, l*) ventral, and (*g*) left ventrolateral views. f.il, facet for ilium; f.sr2, facet for sacral rib 2; fo, fossa; la, lamina; ns, neural spine; posf, postspinal fossa; prsf, prespinal fossa; prz, prezygapophysis; sr, sacral rib; tu, tuberosity; ve-sr.b, vertebra-sacral rib boundary. Scale bars equal 1 cm.

This element is represented by two specimens. GSI 2118 (Huxley [58]: plate II, figure 7; figure 26*h*–*l* and table 10) lacks the left prezygapophysis, both postzygapophyses, the distal end of the neural spine and the left rib. The anterior and posterior surfaces of the centrum are damaged and the posterior end and right posterolateral corner of the base of the neural arch are reconstructed. GSI 2120 (Huxley [58]: plate III, figure 1; figure 26*a*–*g* and table 10) is better preserved, missing the posterior half of the centrum, postzygapophyses, neural spine and the posteroventral corner of the distal end of the sacral ribs. However, the neural spine of this specimen was originally complete and its description is based on the illustration of Huxley ([58]: plate III, figure 1; figure 26*a*–*c*).

**Table 10.** Measurements in millimetres of Panchet cf. proterosuchid sacral vertebrae. S1a (GSI 2118), S1b (GSI 2120), and S2b (NHMUK PV R37579). S, sacral vertebra. Values with an asterisk indicate incomplete measurements (owing to post-mortem damage), values between square brackets indicate estimated measurements, and the value given is the maximum measurable. The maximal deviation of the callipers is 0.02 mm, but measurements were rounded to the nearest 0.1 mm.

	S1a	S1b	S2b
length of centrum	15.7	9.3*	23.9
anterior height of centrum	12.6*	16.0	18.5
anterior width of centrum	13.1*	18.7	17.1
posterior height of centrum	12.7*	—	20.0
posterior width of centrum	11.2*	—	18.8
length across zygapophyses	—	14.9*	19.9*
height neural spine	—	—	17.0*
length neural spine at base	—	—	13.8
maximum height	33.8*	28.1*	43.5*
width vertebra + ribs	[41.8]	53.2	[57.4]

The centrum is slightly transversely compressed around its mid-length, acquiring a spool-shape in ventral view. The ventral surface of the centrum is continuously transversely convex, without keel or groove. The anterior articular surface is shallowly concave and slightly transversely broader than tall, whereas the posterior surface is mostly flat and its contour cannot be determined because of breakage in both specimens. The lateral surface of the centrum lacks a lateral fossa. The transverse process extends from the dorsal half of the centrum onto the lateral surface of the neural arch and is restricted to the anterior half of the vertebra. A moderately deep, circular and posteriorly facing fossa is present on the posterior surface of the transverse process and adjacent to the boundary with the sacral rib (figure 26*c*,*f*: fo). The prezygapophysis is short, mainly dorsally oriented, and extends slightly anterior to the level of the anterior margin of the centrum (figure 26: prz). The articular facet of the prezygapophysis is oval, with a transverse main axis. There is a thick, well-developed lamina that extends from the lateral margin of the base of the prezygapophysis towards the neural spine (figure 26*d*: la), but it is not possible to determine if it reached the latter structure because of damage. The prespinal and postspinal fossae are restricted to the base of the neural spine, with the latter fossa being the deepest (figure 26*a*,*c*: posf, prsf). The neural spine is tall and mostly vertical, with parallel anterior and posterior margins (figure 26*a*–*c*), resembling the condition in *Erythrosuchus africanus* (NMHUK PV R3592). By contrast, the first sacral neural spine of *Proterosuchus alexanderi* is slightly posterodorsally oriented (NMQR 1484). The anterior margin of the neural spine is concave along its proximal half in lateral view and, as a result, the distal half of the spine is more anteriorly projected than its base, contrasting with the continuously straight anterior margin of the neural spine of *Erythrosuchus africanus* (NMHUK PV R3592). The neural spine lacks a distal transverse expansion, but it is slightly and continuously transversely expanded in *Erythrosuchus africanus* (NMHUK PV R3592). The suture between the vertebra and the sacral ribs is closed, but a laterally inflated and striated surface on both sides of the bone is a vestige of this suture in GSI 2120 (figure 26*a*: ve-sr.b). This sutural vestige cannot be observed in GSI 2118, but it is probably a result of preservation rather than genuine absence.

The proximal end of the sacral rib is dorsoventrally and anteroposteriorly expanded, with a ventral margin finishing well dorsal to the anteroventral margin of the centrum. This condition resembles that of *Garjainia prima* [140], *Garjainia madiba* [153] and *Cuyosuchus huenei* (MCNAM PV 2669). By contrast, the proximal end of the first sacral rib of *Erythrosuchus africanus* (NHMUK PV R3592) and *Shansisuchus shansisuchus* [146] is proportionally dorsoventrally taller, reaching the anteroventral margin of the centrum. In addition, the anterior end of the proximal region of the sacral rib of *Erythrosuchus africanus* extends anteriorly to the anterior surface of the centrum and would have contacted extensively the last dorsal centrum (NHMUK PV R3592). In the Panchet cf. proterosuchid specimens and other early archosauriforms (e.g. *Garjainia prima*: [140]; *Garjainia madiba*: [153]; *Cuyosuchus huenei*: MCNAM PV 2669), the sacral rib extends only incipiently anterior to the centrum. The cross-section of the sacral rib at mid-length is subtriangular, with a ventrally oriented apex, and slightly dorsoventrally taller than anteroposteriorly long. The anterior surface of the base of the rib possesses a shallow fossa, which is adjacent to the dorsolateral margin of the vertebral centrum. There is thick tuberosity that extends transversely along the anterodorsal surface of the rib (figure 26*a*: tu). This tuberosity defines dorsally a shallowly dorsoventrally concave surface on the anterior surface of the rib that becomes dorsoventrally convex on the anteroventral surface. The dorsal surface of the rib is anteroposteriorly flat and possesses multiple, mainly transversely oriented striations, as occurs in other early archosauriforms (e.g. *Proterosuchus alexanderi*: NMQR 1484; ‘*Chasmatosaurus*’ *yuani*: IVPP V2719, V4067; *Erythrosuchus africanus*: NHMUK PV R3592). The striations that are closer to the anterior margin are anterolaterally oriented and those closer to the posterior margin are posterolaterally oriented. The ventral surface of the rib is strongly anteroposteriorly convex and the posterior surface is dorsoventrally concave. The posterior and dorsal surfaces of the rib are separated from each other by a sharp edge that extends along the posterodorsal surface of the bone.

The distal end of the rib is well-expanded anteroposteriorly and ventrally, whereas the dorsal expansion is only incipient. The distal articular facet of the rib for the ilium is anteroposteriorly longer than tall and L-shaped in contour, with an anterior main surface and a small posterior projection on its posteroventral corner (figure 26: f.il). The distal end of the sacral rib is not subdivided by a deep notch, contrasting with the condition in *Proterosuchus alexanderi* (NMQR 1484), but resembling other early archosauriforms (‘*Chasmatosaurus*’ *yuani*: IVPP V2719, V4067; *Garjainia prima*: [140]; *Erythrosuchus africanus*: NHMUK PV R3592). The anterior portion of the articular facet is subdivided into four subtriangular areas, the apices of which converge close to the centre of the facet (figure 26*g*). This subdivision of the articular surface does not occur in ‘*Chasmatosaurus*’ *yuani* (IVPP V2719). The dorsal area is deeply concave, well defined ventrally and mostly restricted to the dorsal third of the facet. The anterior area is convex and restricted to the anterior third of the facet. The posterior and ventral areas occupy most of the surface of the facet, being shallowly concave and separated from each other by an anterodorsally-to-posteroventrally oriented change in slope. The facet for articulation with the second sacral rib is raised on a posteriorly oriented peduncle placed on the posteroventral surface of the rib and is oval in cross-section, being slightly dorsoventrally taller than broad (figure 26: f.sr2).

#### 6.4.2. Second sacral vertebra and rib

**Figure 27.**
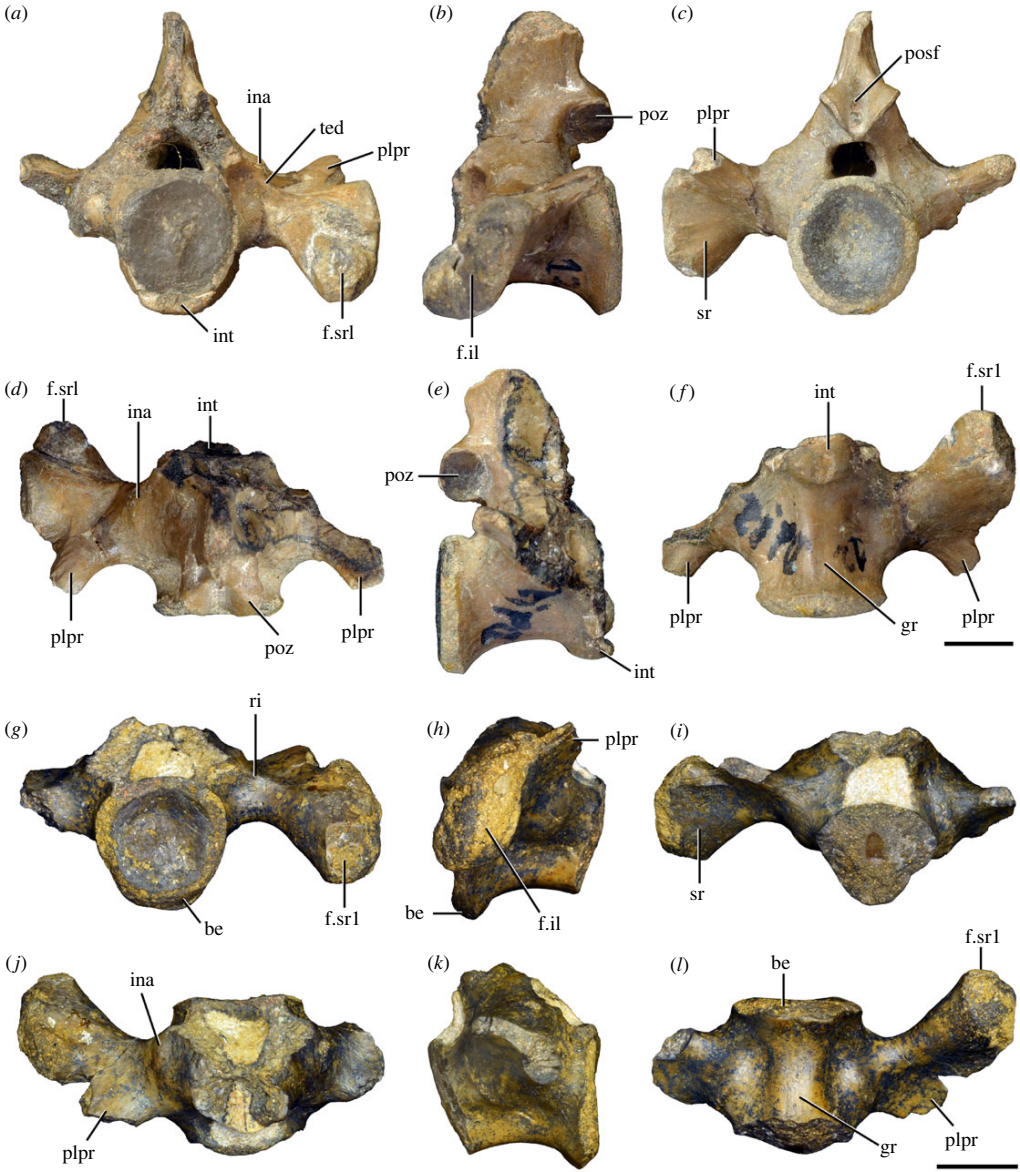
Panchet cf. proterosuchid second sacral vertebrae and ribs. (*a–f*) NHMUK PV R37579 and (*g–l*) ISIR 1111 in (*a, h*) anterior, (*b, k*) left lateral, (*c, i*) posterior, (*d*) dorsal, (*e, j*) right lateral, (*f, l*) ventral, and (*g*) left ventrolateral views. be, bevelling; f.sr1, facet for sacral rib 1; gr, groove; ina, inflated area; int, intercentrum; plpr, posterolateral process; posf, postspinal fossa; poz, postzygapophysis; ri, ridge; sr, sacral rib. Scale bars equal 1 cm.

Four or five partial second sacral vertebrae fused to their respective ribs (NHMUK PV R37579, ISIR 1109, ISIR 1111, ISIR 1116 and probably ISIR 1110) and one left second sacral rib (NHMUK PV R37585) are preserved. These vertebrae and ribs possess a congruent morphology between each other. The most complete vertebra (NHMUK PV R37579; figure 27*a*–*f* and table 10) lacks the prezygapophyses and distal end of the neural spine, whereas its left rib lacks the tip of the posterolateral process and the right rib is severely damaged. ISIR 1111 (figure 27*g*–*l*) is a more incomplete specimen, lacking the posterior end of the centrum, zygapophyses and neural spine. The other second sacral vertebrae lack most of their neural arches, including the zygapophyses and neural spine, and their ribs are strongly damaged (ISIR 1109, ISIR 1110, ISIR 1116, NHMUK PV R37585). These elements are interpreted as second sacral vertebrae and ribs because the main axis of the articular facet of the sacral rib is oblique, anteroventrally-to-posterodorsally oriented, and extends dorsally well dorsal to the level of the dorsal margin of the centrum, as occurs in other early archosauromorphs.

The centrum is slightly transversely compressed around its mid-length, acquiring a spool-shape in ventral view. The ventral surface of the centrum is continuously transversely convex and in NHMUK PV R37579 and ISIR 1111 possesses a subtle median groove (figure 27: gr), which is absent in the other second sacral centra. The anterior articular surface of the centrum is sub-circular in some specimens (ISIR 1111), but dorsoventrally taller than broad in others (NHMUK PV R37579, ISIR 1110). In all the specimens, this articular surface is shallowly concave. The ventral margin of the anterior articular facet of NHMUK PV R37579 is fused to an intercentrum (figure 27: int; see below), whereas the anteroventral margin of the centrum is bevelled in ISIR 1111 and very likely also received an intercentrum (figure 27: be). The posterior surface of the centrum is sub-circular, but more deeply concave than the anterior one. The lateral surface of the centrum is anteroposteriorly concave, lacks a lateral fossa, and possesses either a pair of or multiple small, oval foramina depending on the specimen. A rounded, inflated area that runs anteromedially-to-posterolaterally is a remnant of the suture between the vertebra and its sacral ribs, showing that the transverse process is poorly laterally developed. The transverse process extends from the dorsal half of the centrum onto the lateral surface of the neural arch. The anterior surface of the transverse process is transversely concave and delimited dorsally by a broad, low ridge that connects the base of the prezygapophysis with the sacral rib (figure 27*a*,*g*: ri). There is a low, rounded inflated area on the dorsal surface of the transverse process (figure 27*d*,*j*: ina), which is adjacent to the aforementioned ridge. This subtle expansion possibly indicates the position of the suture between the transverse process and the rib on the dorsal surface. The postzygapophyses extend posteriorly up to the same level as the posterior margin of the centrum and weakly diverge from each other in dorsal view. The postzygapophyseal facet is sub-oval, with a transverse main axis, and there is no hyposphene. The postspinal fossa is dorsoventrally long and moderately deep, invading the very base of the neural spine dorsal to the postzygapophyses (figure 27*c*: posf). By contrast, a more dorsally developed postspinal fossa, extending well dorsal to the level of the postzygapophyses, occurs in *Garjainia prima* (PIN 951/37-1, 2), and *Erythrosuchus africanus* (NHMUK PV R3592). The base of the neural spine is anteroposteriorly long and its posterior margin is posterodorsally oriented, resembling the condition in *Proterosuchus alexanderi* (NMQR 1484), ‘*Chasmatosaurus*’ *yuani* (IVPP V4067), *Garjainia prima* (PIN 951/37-1, 2) and *Erythrosuchus africanus* (NHMUK PV R3592).

The main axis of the base of the sacral rib is oriented mainly in a sagittal plane and gradually becomes more oblique, being posterodorsally-to-anteroventrally oriented towards the distal end of the bone. As a result, the main axis of the distal articular facet of this rib is more obliquely oriented than that of the first sacral rib (figure 27*b*,*h*). The second sacral rib is strongly anteriorly expanded and, as a result, the distal articular facet for articulation with the pelvic girdle extends anteriorly beyond the level of the anterior margin of the centrum. The anterior surface of the distal end of the sacral rib possesses a sub-oval to sub-rectangular, dorsoventrally taller than transversely broad, articular facet for contact with the posterolateral region of the first sacral rib (figure 27: f.sr1). The distal end of the second sacral rib of the Panchet cf. proterosuchid specimens is distinctly notched, resulting in anterolateral and posterolateral projections (figure 27: plpr), as occurs in some lepidosauromorphs and several non-eucrocopodan archosauromorphs (e.g. *Macrocnemus bassanii*, *Pamelaria dolichotrachela*, *Mesosuchus browni*, *Prolacerta broomi*, *Proterosuchus alexanderi*, *Cuyosuchus huenei*, *Garjainia prima*; [9,18,142,150]). However, the second sacral rib lacks this notch in ‘*Chasmatosaurus*’ *yuani* (IVPP V4067), most erythrosuchids and non-avemetatarsalian eucrocopods [18]. The posterolateral process of the Panchet cf. proterosuchid specimens is oriented at an angle of *ca* 35° with respect to the sagittal plane and forms an obtuse angle with the anterolateral portion of the rib in dorsal view. By contrast, this process is more laterally oriented and forms an acute angle with the anterolateral portion of the rib in *Noteosuchus colletti* (AM 3591: *ca* 60°), *Mesosuchus browni* (SAM-PK-6046: *ca* 45°), *Proterosuchus alexanderi* (NMQR 1484: *ca* 50°), *Prolacerta broomi* (Gow [161]: figure 22: *ca* 55°) and *Garjainia prima* (PIN 951/37-1, 2: *ca* 55°). A different condition occurs in *Pamelaria dolichotrachela* (ISIR 333/1) and *Cuyosuchus huenei* (Rusconi [162]: figure 17), in which the posterolateral process is mainly posteriorly oriented (*ca* 0°) in dorsal view. The anterolateral projection is dorsoventrally thicker than the posterolateral one and harbours the distal articular facet of the rib. The shape of the posterior margin of the posterolateral projection (i.e. whether it is squared or tapers distally) cannot be determined because of lack of preservation. The dorsal surface of the base of the sacral rib is flat and its anterior surface is dorsoventrally convex. Both surfaces are separated from each other by a sharp change in slope. The dorsal surface of the rib, adjacent to the distal articular surface, possesses two short grooves delimited by three posteromedially-to-anterolaterally oriented ridges. The ventral surface of the rib is anteroposteriorly concave at its base and along the preserved portion of the posterolateral projection and becomes convex at its anterolateral end.

### 6.5. Caudal vertebrae

#### 6.5.1. Anterior caudal vertebrae

**Figure 28.**
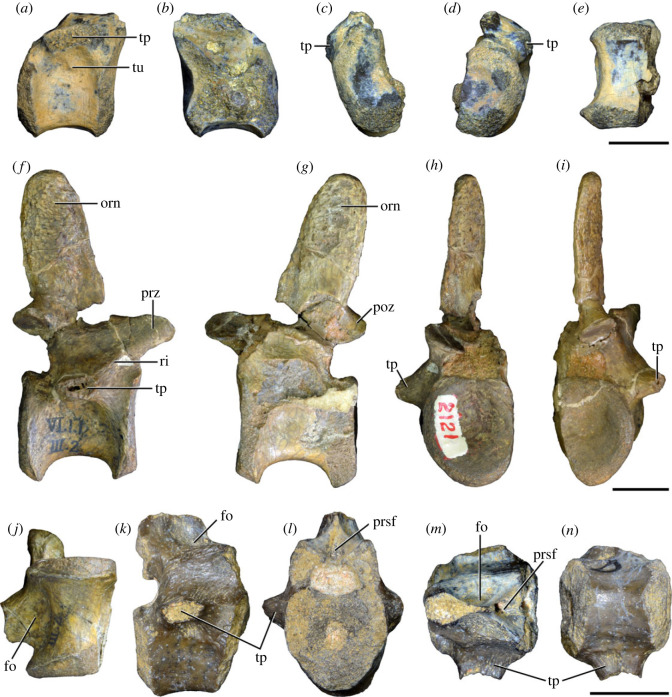
Panchet proterosuchid anterior caudal vertebrae of ‘position A’. (*a–e*) ISIR 1117, (*f–j*) GSI 2121, and (*k–n*) ISIR 1113 in (*a, f, k*) right lateral, (*b, g*) left lateral, (*c, h, l*) anterior, (*d, i*) posterior, (*e, j, n*) ventral, and (*m*) dorsal views. fo, fossa; orn, ornamentation; poz, postzygapophysis; prsf, prespinal fossa; prz, prezygapophysis; ri, ridge; tp, transverse process; tu, tuberosity. Scale bars equal 1 cm.

Seven anterior caudal vertebrae belonging to at least four positions/sectors in the anterior region of the tail are preserved. The two more anterior positions are described together as ‘position A’ (figure 28) and the two more posterior positions are described as ‘position B’ (figures 30 and 31) because of their similarities.

**Figure 29.**
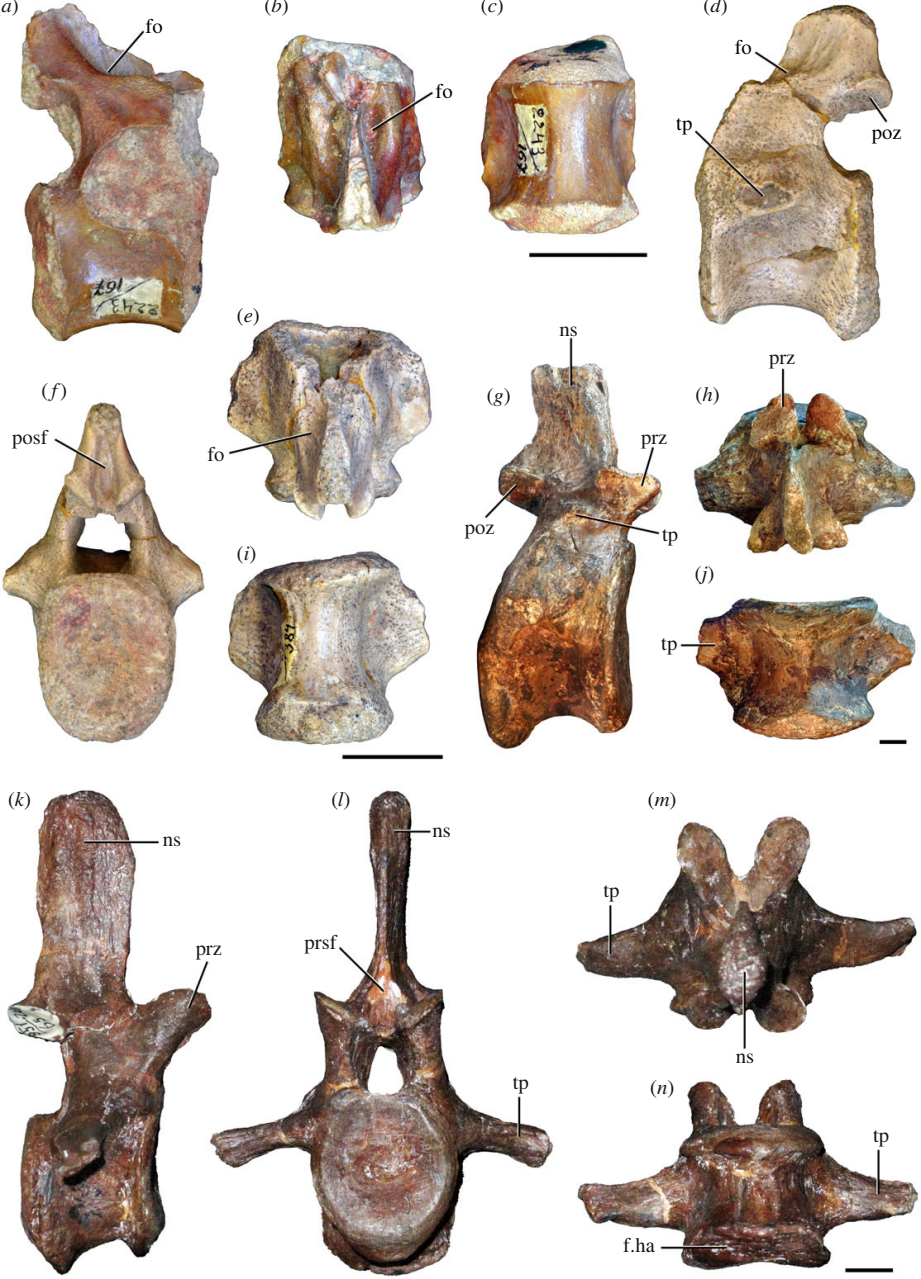
Comparison between anterior caudal vertebrae of selected Early and Middle Triassic non-eucrocopodan archosauriforms. (*a–c*) *Chasmatosuchus rossicus* (PIN 2243/167), (*d–f, i*) *Chasmatosuchus rossicus* (PIN 2252/384), (*g, h, j*) *Erythrosuchus africanus* (NHMUK PV R3592), and (*k–n*) *Garjainia prima* (PIN 951/65) in (*a, g, k*) right lateral, (*b, e, h, m*) dorsal, (*c, i, j, n*) ventral, (*d*) left lateral, (*f*) posterior, and (*l*) anterior views. f.ha, facet for haemal arch; fo, fossa; ns, neural spine; posf, postspinal fossa; poz, postzygapophysis; prsf, prespinal fossa; prz, prezygapophysis; tp, transverse process. Scale bars equal 1 cm.

**Figure 30.**
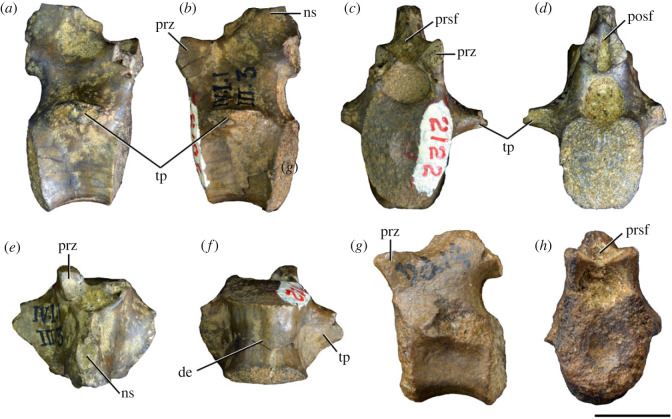
Panchet proterosuchid anterior caudal vertebrae of ‘position B’. (*a–f*) GSI 2122 and (*g, h*) NHMUK PV R37581 in (*a*) right lateral, (*b, g*) left lateral, (*c, h*) anterior, (*d*) posterior, (*e*) dorsal, and (*f*) ventral views. de, depression; ns, neural spine; posf, postspinal fossa; prsf, prespinal fossa; prz, prezygapophysis; tp, transverse process. Scale bar equals 1 cm.

**Figure 31.**
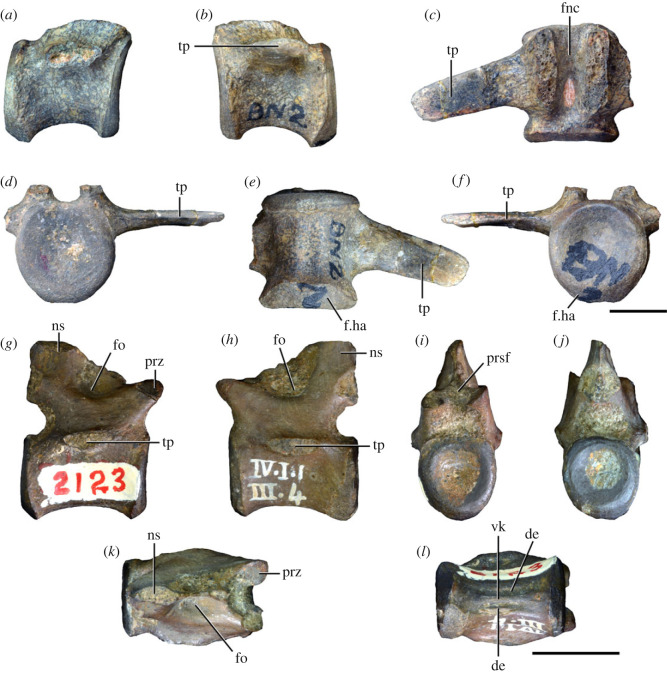
Panchet proterosuchid anterior caudal vertebrae of ‘position B’. (*a–f*) NHMUK PV R 37576 and (*g–l*) GSI 2123 in (*a, g*) right lateral, (*b, h*) left lateral, (*c, k*) dorsal, (*d, i*) anterior, (*e, l*) ventral, and (*f, l*) posterior views. de, depression; f.ha, facet for haemal arch; fnc, floor of the neural canal; fo, fossa; ns, neural spine; prsf, prespinal fossa; prz, prezygapophysis; tp, transverse process; vk, ventral keel. Scale bars equal 1 cm.

##### 6.5.1.1. Position A

ISIR 1117 (figure 28*a*–*e*) is one of the first caudal vertebrae because its centrum is proportionally tall and the anterior articular surface is dorsoventrally asymmetric, being distinctly more extended anteriorly towards the dorsal margin than ventrally. This condition characterizes the most anterior caudal vertebrae of other archosauromorph species (e.g. *Elorhynchus carrolli*: [103]; *Garjainia prima*: PIN 951/64; *Erythrosuchus africanus*: [141]). GSI 2121 (Huxley [58]: plate III, figure 2; figure 28*f*–*j* and table 11) is a more posterior caudal vertebrae because the anterior surface of its centrum is more asymmetric than ISIR 1117. The identification of GSI 2121 as an anterior caudal vertebra is based on the presence of a posterolaterally oriented transverse process, almost horizontal prezygapophysis and a tall neural spine. This vertebra is fairly complete, lacking most of the right transverse process and the left zygapophyses and transverse process. The lateral surfaces of the anterior and posterior margins of the centrum are damaged. However, at least the left prezygapophysis was lost subsequently to the description of Huxley [58]. ISIR 1113 (figure 28*k*–*n*) is a partial vertebra that possesses a morphology consistent with that of GSI 2121 and lacks most of the transverse processes, zygapophyses and neural spine. The anterior and posterior surfaces of the centrum of this vertebra are damaged. GSI 2121 is considerably more complete than ISIR 1113 and ISIR 1117 and, thus, the description of ‘position A’ is mostly based on the former specimen.

**Table 11.** Measurements in millimetres of Panchet proterosuchid anterior caudal vertebrae. ACa (GSI 2121), ACb (ISIR 1115), ACc (GSI 2123), ACd (NHMUK PV R37576) and ACe (NHMUK PV R37581). AC, anterior caudal vertebra. Values with an asterisk indicate incomplete measurements (owing to post-mortem damage) and the value given is the maximum measurable. The maximal deviation of the callipers is 0.02 mm, but measurements were rounded to the nearest 0.1 mm.

	ACa	ACb	ACc	ACd	ACe
length of centrum	20.6	15.9	14.4	20.2	13.4*
anterior height of centrum	19.3	—	10.5	18.1	12.7
anterior width of centrum	15.6*	—	9.2	17.3	12.6
posterior height of centrum	19.4	14.6	9.6	17.8	11.8*
posterior width of centrum	13.5*	12.9	8.8	17.2	11.0*
length across zygapophyses	27.4	—	15.8*	—	17.0*
height neural spine	28.2	—	6.5*	—	—
length neural spine at base	—	—	11.2	—	—
maximum height	53.8	20.7*	20.6	22.7*	23.4*

The centrum of GSI 2121 is 1.07 times longer than its anterior height and very slightly parallelogram-shaped in lateral view, with the anterior articular surface positioned more dorsally than the posterior one. This condition closely resembles that in an anterior caudal vertebra that has been referred to *Chasmatosuchus rossicus* (1.07: PIN 2243/167; [40]), whereas the anteriormost caudal centra are approximately as long as tall and proportionally shorter in *Garjainia prima* (0.83–1.04: PIN 951/64-28, 30, 31: Ca1, 3 and 4) and an anterior caudal vertebra of the ‘Arcadia proterosuchian’ (0.97: QMF9534), and considerably shorter in *Erythrosuchus africanus* (0.72–0.81: Gower [141]: table 2, Ca1, 2 and 5) (figure 29). The anterior caudal vertebrae of *Cuyosuchus huenei* are proportionally longer than in the above-mentioned specimens and species (1.29: MCNAM PV 2669). Both anterior and posterior articular surfaces of the centrum are dorsoventrally taller than broad and concave in the Panchet proterosuchid specimens, as occurs in the anteriormost caudal vertebrae of several other early archosauriforms (e.g. specimens referred to *Chasmatosuchus rossicus*: PIN 2243/167, 2252/384; *Garjainia prima*: PIN 951/64-29; *Erythrosuchus africanus*: [141]). These surfaces lack a notochordal pit, but their deepest area is situated dorsal to the centre of the facet, resembling the position of the pit in the presacral vertebrae that possess this feature. However, a small, circular notochordal pit is present on the posterior surface of the centrum of ISIR 1113. The centrum is constricted transversely at mid-length and, as a result, is spool-shaped in ventral view. The ventral surface of the centrum is transversely flat and lacks a ventral groove or keel. The lateral surface of the centrum has a very shallow lateral fossa lacking a pronounced rim that is placed immediately ventral to the base of the transverse process. ISIR 1117 possesses a small tuberosity positioned ventral to the base of the transverse process and approximately at mid-length on the centrum (figure 28*a*: tu), resembling the condition in the posterior cervical and dorsal vertebrae of *Samsarasuchus pamelae*. This tuberosity is absent in ISIR 1113 and GSI 2121. The neurocentral suture is closed in the three specimens.

The transverse process is positioned level with the dorsal margin of the centrum and its anterior margin slants posteriorly in dorsal view (figure 28*h*–*j*,*m*,*n*), as in other early archosauriforms (e.g. ‘*Chasmatosaurus*’ *yuani*: IVPP V4067; *Proterosuchus alexanderi*: NMQR 1484; *Cuyosuchus huenei*: MCNAM PV 2669; *Garjainia prima*: PIN 951/64; *Erythrosuchus africanus*: NHMUK PV R3592) (figure 29). However, the overall orientation of the transverse processes cannot be determined in these anteriormost caudal vertebrae of the Panchet proterosuchid specimens. The ventral surface of the base of the transverse process possesses a shallow fossa that is delimited by a pair of thick tuberosites that converge laterally. The base of the transverse process is oval in cross-section, being considerably anteroposteriorly longer than tall. There is a low ridge that extends anterodorsally from the anterior margin of the transverse process and reaches the lateroventral surface of the prezygapophysis. This ridge defines dorsally a shallow, anterolateroventrally facing depression. The prezygapophysis slants slightly dorsally in lateral view and are almost parallel to the sagittal axis in dorsal view (figure 28*f*–*j*). The prezygapophysis extends anteriorly considerably beyond the level of the centrum. The articular facet of the prezygapophysis is oval, with an anterolaterally-to-posteromedially oriented main axis and slants medially. The postzygapophysis is short and extends posteriorly only slightly beyond the level of the centrum. The articular facet of the postzygapophysis is circular and slants medially. It is not possible to determine the presence of a hyposphene because of breakage.

The lateral surface of the base of the neural spine of ISIR 1113 is invaded by a deep fossa on its anterior half (figure 28*k*: fo), closely resembling the condition in anterior caudal vertebrae referred to *Chasmatosuchus rossicus* (PIN 2243/167, 2252/384, 2252/386; [40]; figure 29*a*,*d*,*e*: fo). By contrast, this depression is considerably shallower to absent in ‘*Chasmatosaurus*’ *yuani* (IVPP V4067), *Proterosuchus alexanderi* (NMQR 1484), *Cuyosuchus huenei* (MCNAM PV 2669), *Garjainia prima* (PIN 951/64-29) and *Erythrosuchus africanus* (NHMUK PV R3592) (figure 29). In ISIR 1113, the prespinal fossa is deep and dorsoventrally short, invading the very base of the neural spine, as in *Cuyosuchus huenei* (MCNAM PV 2669), but contrasting with the presence of a more dorsally extended fossa in *Erythrosuchus africanus* [141]. The latter two features are not preserved in GSI 2121. The neural spine is considerably taller than the centrum and slants slightly posteriorly in lateral view (figure 28*f*–*i*), resembling the condition in ‘*Chasmatosaurus*’ *yuani* (IVPP V4067) and *Garjania prima* (PIN 951/64-29; figure 29*k*,*l*: ns). A similarly tall, but vertical, neural spine is present in *Cuyosuchus huenei* (MCNAM PV 2669). The anterior and posterior margins of the neural spine are convex in lateral view and converge gradually upon a rounded distal margin. Both margins of the neural spine are very sharp. The distal two-thirds of the lateral surface of the neural spine are slightly inflated and possess striations and pits (figure 28*f*,*g*: orn), resembling the condition in ‘*Chasmatosaurus*’ *yuani* (IVPP V4067) and *Garjania prima* (PIN 951/64-29; figure 29*k*,*l*), whereas the anterior caudal vertebrae of *Proterosuchus alexanderi* (NMQR 1484) seem to lack conspicuous ornamentation. The distal end of the neural spine is not transversely expanded (figure 28*h*,*i*), as in other early archosauriforms (e.g. ‘*Chasmatosaurus*’ *yuani*: IVPP V4067; *Proterosuchus alexanderi*: NMQR 1484; *Cuyosuchus huenei*: MCNAM PV 2669; *Garjania prima*: PIN 951/64-29; figure 29*l*).

##### 6.5.1.2. Position B

This position is represented by four isolated caudal vertebrae that probably belong to at least two different regions of the anterior portion of the tail because of differences in their proportional centrum elongation. These vertebrae differ from those of ‘position A’ in a more anterodorsal orientation of the prezygapophysis (unknown in NHMUK PV R37576) and the absence of a ridge connecting the transverse process with the prezygapophysis. The two more anterior vertebrae of ‘position B’ are GSI 2122 (Huxley [58]: plate III, figure 3; figure 30*a*–*f*) and NHMUK PV R37581 (figure 30*g*,*h* and table 11). The former specimen lacks most of the transverse processes, postzygapophyses, right prezygapophysis and neural spine, and the distal end of the left prezygapophysis, and the anterior and posterior surfaces of the centrum are damaged. NHMUK PV R37581 lacks most of the transverse processes, postzygapophyses, and neural spine. The more elongated, and probably more posterior, vertebrae of ‘position B’ are GSI 2123 (Huxley [58]: plate III, figure 4; figure 30*a*–*f* and table 11) and NHMUK PV R37576 (figure 30*g*,*h* and table 11). GSI 2123 lacks most of the transverse processes, postzygapophyses and neural spine, and the distal end of the right prezygapophysis. NHMUK PV R37576 lacks most of the right transverse process and all the neural arch with exception of part of the walls of the neural canal.

The length versus anterior height ratio of the centrum is 1.05 in NHMUK PV R37581, 1.12 in NHMUK PV R37576 and 1.37 in GSI 2123 (figures 30 and 31). This ratio cannot be estimated in GSI 2122 because of damage. This ratio resembles that of a specimen referred to *Chasmatosuchus rossicus* (PIN 2243/384; figure 29*d*) and some anterior caudal vertebrae of *Cuyosuchus huenei* (MCNAM PV 2669) and *Garjainia prima* (PIN 951/64; figure 29*k*), but it is higher than the ratio of the anterior caudal vertebrae of the ‘Arcadia proterosuchian’ (QMF9534) and *Erythrosuchus africanus* ([141]; figure 29*g*). The centrum is slightly-to-moderately parallelogram-shaped in lateral view, with the anterior articular surface positioned more dorsally than the posterior one. Both anterior and posterior articular surfaces of the centrum are dorsoventrally taller than broad and concave. When the articular surface of the centrum is exposed, there is a notochordal pit slightly dorsally displaced from its centre. The centrum is slightly constricted transversely at mid-length and, as a result, is spool-shaped in ventral view. The ventral surface of the centrum possesses a pair of shallow longitudinal depressions situated immediately lateral to the median line (GSI 2122, 2123; figures 30*f* and 31*l*: de) or a single median longitudinal depression (NHMUK PV R37581, R37576; figure 31*e*). In addition, GSI 2123 has a low median longitudinal keel that is bordered collaterally by the pair of depressions (figure 31*l*: vk). This keel is absent in the other three vertebrae and those of ‘position A’, which, instead, have a strongly transversely convex ventral surface. The posteroventral surface of the centrum is strongly bevelled for reception of its respective haemal arch, a condition that it is better preserved in NHMUK PV R37576 (figure 31*e*,*f*: f. ha). The lateral surface of the centrum is slightly dorsoventrally convex and lacks a lateral fossa in GSI 2122, but possesses a shallow fossa in the other three specimens immediately ventral to the base of the transverse process. The neurocentral suture is closed in all the specimens.

The transverse process is situated level with the dorsal margin of the centrum. Its anterior and posterior margins are almost parallel to each other in dorsal view, acquiring a trapezoidal profile (NHMUK PV R37576; figure 31*c*,*e*: tp). The complete transverse process of NHMUK PV R37576 is 1.07 times the length of the centrum and almost parallel to the transverse plane in this specimen and GSI 2122. The transverse process is posteroventrally oriented at an angle of *ca* 17° with respect to the sagittal plane in NHMUK PV R37576 (figure 31*c*,*e*), resembling the condition in ‘*Chasmatosaurus*’ *yuani* (IVPP V4067) and some anterior caudal vertebrae of *Garjainia prima* (PIN 951/65-26), *Proterosuchus alexanderi* (NMQR 1484) and *Cuyosuchus huenei* (MCNAM PV 2669), but the transverse process is almost orthogonal to the sagittal plane in other vertebrae of the same specimen of the latter three species. The ventral surface of the transverse process of the Panchet proterosuchid specimens is anteroposteriorly convex and lacks a fossa. The base of the transverse process is oval in cross-section, being considerably anteroposteriorly longer than tall.

The prezygapophysis is anterodorsally oriented in lateral view and mainly anteriorly to anterolaterally oriented in dorsal view. The prezygapophysis extends anteriorly beyond the level of the centrum, as in other early archosauriforms (e.g. *Garjainia prima*: PIN 951/65-26, 28; *Erythrosuchus africanus*: NHMUK PV R3592). The articular facet of the prezygapophysis is oval and slants medially. The lateral margin of the prezygapophysis is connected with the anterior margin of the base of the neural spine by a thick, medially curved ridge. This ridge defines a shallow and transversely broad prespinal fossa that is restricted to the very base of the neural spine. Another, thicker ridge extends posteriorly from the prezygapophysis to slightly posterior to the mid-length of the neural spine. This ridge is slightly laterally bowed and defines laterally a depression on the lateral surface of the base of the neural spine, immediately posterior to the base of the prezygapophysis (figure 31*g*,*h*,*k*: fo). This is the same condition as the anterior caudal vertebrae of ‘position A’ (figure 28*k*,*m*: fo), but the fossa is considerably shallower in GSI 2122 and NHMUK PV R37581. This fossa lateral to the neural spine is absent in ‘*Chasmatosaurus*’ *yuani* (IVPP V4067), *Proterosuchus alexanderi* (NMQR 1484), *Garjainia prima* (PIN 951/65-26, 28) and *Erythrosuchus africanus* (NHMUK PV R3592). The postspinal fossa is moderately deep, transversely narrow and restricted to the base of the neural spine. The anterior margin of the base of the neural spine is posterodorsally-to-anteroventrally oriented and curves gently dorsally in NHMUK PV R37581 and more abruptly in GSI 2122. There is no anterior spur on the neural spine, as occurs in Ca1−5 of *Proterosuchus alexanderi* (NMQR 1484), Ca1−Ca8 of *Proterosuchus yauni* (IVPP V4067) and anterior caudal vertebrae of *Cuyosuchus huenei* (MCNAM PV 2669), *Garjainia prima* (PIN 951/65-26, 28), *Erthrosuchus africanus* [141] and specimens referred to *Chasmatosuchus rossicus* (PIN 2243/384). The posterior margin of the base of the neural spine possesses a very sharp edge in GSI 2123, which is not preserved in the other specimens.

#### 6.5.2. Middle caudal vertebrae

**Figure 32.**
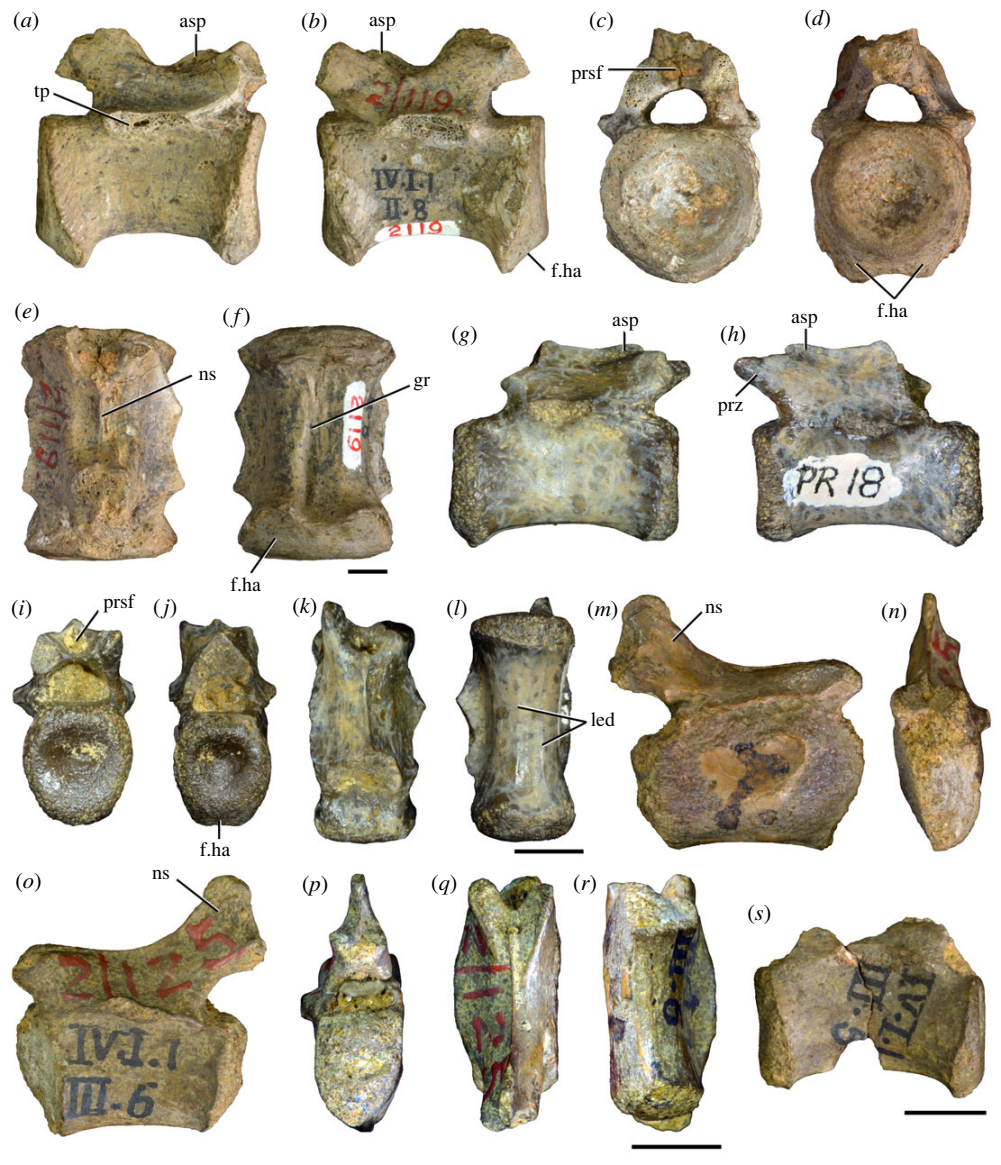
Panchet cf. proterosuchid middle caudal vertebrae. (*a–f*) GSI 2119, (*g–l*) ISIR 1120, (*m–r*) GSI 2125, and (*s*) GSI 2124 in (*a, g, m, s*) right lateral, (*b, h, o*) left lateral, (*c, i, n, p*) anterior, (*d, j*) posterior, (*e, k, q*) dorsal, and (*f, l, r*) ventral views. asp, anterior spur; f.ha, facet for haemal arch; gr, groove; led, longitudinal edge; ns, neural spine; prsf, prespinal fossa; prz, prezygapophysis; tp, transverse process. Scale bars equal 5 mm.

There are six middle caudal vertebrae preserved in the Panchet Formation early archosauriform sample (ISIR 1118−1120, GSI 2119, 2124, 2125; figure 32 and table 12). All these elements have a proportionally longer centrum than that of the anterior caudal vertebrae and possess differences in their morphology that are consistent with changes in the caudal series observed in other early archosauriforms (e.g. ‘*Chasmatosaurus*’ *yuani*: IVPP V4067; *Proterosuchus alexanderi*: NMQR 1484). As a result, the middle caudal vertebrae of the Panchet cf. proterosuchid specimens are described in three successively more posterior positions/sections. ISIR 1119 is represented only by a centrum and may belong to either position A or B.

**Table 12.** Measurements in millimetres of Panchet cf. proterosuchid middle and posterior caudal vertebrae. MCa (GSI 2119), MCb (ISIR 1118), MCc (GSI 2124), MCd (ISIR 1120), MCe (ISIR 1119), PCa (GSI 2126), PCb (ISIR 1121), and PCc (ISIR 1123) caudal vertebrae. MC, middle caudal vertebra; PC, posterior caudal vertebrae. Values with an asterisk indicate incomplete measurements (owing to post-mortem damage) and the value given is the maximum measurable. The maximal deviation of the callipers is 0.02 mm, but measurements were rounded to the nearest 0.1 mm.

	MCa	MCb	MCc	MCd	MCe	PCa	PCb	PCc
length of centrum	28.6	15.3	14.1	17.4	13.2	12.7	12.9	[15.0]
anterior height of centrum	19.6	9.7*	8.1	9.1	7.8	5.1	4.8	5.4*
anterior width of centrum	19.3	8.7*	7.5	8.2	7.6	4.6	5.0	4.9
posterior height of centrum	18.4	10.8	8.0	9.4	7.2	5.0	4.6	5.9
posterior width of centrum	19.1	[9.6]	7.2	8.1	7.3	4.6	4.9	5.8
length across zygapophyses	26.4*	—	—	15.3*	—	9.1*	—	—
height neural spine	—	—	—	—	—	—	—	2.8*
length neural spine at base	16.7	—	—	8.3*	—	—	—	12.3
maximum height	30.5*	13.5*	12.0*	16.8*	8.6*	8.0*	5.6*	10.7*

##### 6.5.2.1. Position A

This region of the middle caudal series is represented by GSI 2119 (Huxley [58]: plate II, figure 8; figure 32*a*–*f* and table 12) and ISIR 1118 (table 12). GSI 2119 lacks most of the transverse processes, the right prezygapophysis, both postzygapophyses and neural spine, and the distal end of the left prezygpophysis. ISIR 1118 lacks part of the anterior surface of the centrum and most of its neural arch, with exception of the right transverse process. These two vertebrae are described together because of their congruent morphology, but the description is mostly based on GSI 2119.

**Figure 33.**
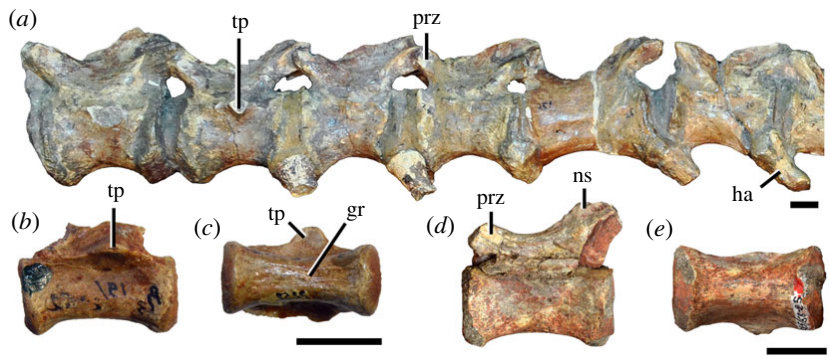
Comparison between middle caudal vertebrae of selected Early and Middle Triassic non-eucrocopodan archosauriforms. (*a*) *Erythrosuchus africanus* (NHMUK PV R3592), (*b, c*) *Chasmatosuchus rossicus* (PIN 2252/383, (*b*) reversed), and (*d, e*) *Sarmatosuchus otschevi* (PIN 2865/68-25, holotype) in (*a, b, d*) lateral and (*c, e*) ventral views. gr, groove; ha, haemal arch; ns, neural spine; prz, prezygapophysis; tp, transverse process. Scale bars equal 5 mm.

The centrum is 1.46 times longer than its anterior height, which is a ratio that falls within the range observed in the middle caudal vertebrae of other early archosauriforms (e.g. *Erythrosuchus africanus*: NHMUK PV R3592). The anterior and posterior articular surfaces of the centrum are positioned approximately at the same dorsoventral level and are sub-circular, concave and lack a distinct notochordal pit. The centrum is slightly constricted transversely at mid-length and, as a result, is spool-shaped in ventral view. The ventral surface of the centrum possesses a transversely broad longitudinal groove that becomes shallower anteriorly (figure 32*f*: gr), resembling the condition in *Garjainia prima* (PIN 951/65-29, 30), *Erythrosuchus africanus* (NHMUK PV R3592) and a specimen referred to *Chasmatosuchus rossicus* (PIN 2252/383; [40]; figure 33*b*: gr). By contrast, the preserved middle caudal vertebra of *Sarmatosuchus otschevi* lacks a ventral groove on its centrum (PIN 2865/68-25; figure 33*e*). The ventral surface of the posterior margin of the centrum is bevelled and has a pair of very well developed, posteroventrally facing facets for articulation with the haemal arch. These facets are separated from each other by a ventrally concave median notch. The lateral surface of the centrum is flat and lacks a lateral fossa. The neurocentral suture is closed in both specimens.

The transverse process (figure 32*a*: tp) is situated level with the dorsal margin of the centrum and lacks a ventral fossa at its base. The base of the transverse process is dorsoventrally compressed. The prezygapophysis is anterodorsally oriented in lateral view and possesses a distinct lateral slant in dorsal view. The lateral margin of the prezygapophysis is connected with the anterior margin of the base of the neural spine by a thick, medially curved ridge, as occurs in the anterior caudal vertebrae of position B. This ridge also defines a shallow and transversely broad prespinal fossa (figure 32: prsf). The presence of a postspinal fossa cannot be determined in any of the specimens because of breakage. The main portion of the neural spine is placed on the posterior half of the neural arch. The anterior surface of the main portion of the neural spine is anteroventrally-to-posterodorsally oriented and possesses three thin ridges, one median and two that are anteromedially oriented. These three ridges define a pair of transversely concave surfaces. The median ridge is connected with the anterior portion of the neural spine, which consists of a flange-like spur with an anterodorsally oriented dorsal margin in lateral view (figure 32*a*,*b*: asp), resembling the condition in ‘*Chasmatosaurus*’ *yuani* (IVPP V4067) and *Proterosuchus alexanderi* (NMQR 1484). By contrast, this anterior spur is absent in the middle caudal vertebra of *Sarmatosuchus otschevi* (PIN 2865/68-25; figure 33*d*), the middle and posterior caudal vertebrae of *Garjainia prima* (PIN 951/65), and *Erythrosuchus africanus* (NHMUK PV R3592; figure 33*a*). In the Panchet cf. proterosuchid specimen, there is a shallow, concave longitudinal depression adjacent to the base of the anterior spur of the neural spine.

##### 6.5.2.2. Position B

This position is represented by ISIR 1120, which lacks most of the right prezygapophysis, both postzygapophyses and the posterior end (main part) of the neural spine (figure 32*g*–*l* and table 12). Only those characters that differ from or cannot be determined in the middle caudal vertebrae of position A are described here. The neurocentral suture is also closed.

The centrum is 1.91 times longer than its anterior height, resembling the condition in the middle caudal vertebra of *Sarmatosuchus otschevi* (1.88: PIN 2865/68-25; figure 33*d*), *Cuyosuchus huenei* (1.76: MCNAM PV 2669), and a specimen referred to *Chasmatosuchus rossicus* (2.0: PIN 2252/383; [40]; figure 33*b*). By contrast, the preserved middle caudal vertebrae of *Garjainia prima* and *Erythrosuchus africanus* have ratios less than 1.25 (PIN 951/65; [141]; figure 33*a*). Both anterior and posterior articular surfaces of the centrum are slightly dorsoventrally taller than broad and possess a notochordal pit. The ventral surface of the centrum is flat and separated from the lateral surfaces by a distinct change in slope that forms a sharp longitudinal edge along the ventrolateral surface of the centrum (figure 32*l*: led), contrasting with the continuously transversely convex ventral surface of the centrum of *Sarmatosuchus otschevi* (PIN 2865/68-25; figure 33*e*). The prezygapophysis is relatively short, being anteriorly extended slightly beyond the level of the anterior margin of the centrum (figure 32*h*). The ridges collateral to the anterior spur of the neural spine that are present in GSI 2119 are absent in ISIR 1120. The anterior spur of the neural spine of ISIR 1120 (figure 32*g*,*h*: asp) is considerably lower than that of GSI 2119.

##### 6.5.2.3. Position C

This region of the middle caudal series is represented by GSI 2124 (Huxley [58]: plate III, figure 5; figure 32*s* and table 12) and GSI 2125 (Huxley [58]: plate III, figure 6; figure 32*m*–*r*). GSI 2124 currently lacks most of its neural arch, but it seems that at least the prezygapophyses were originally more complete. GSI 2125 lacks most of the left transverse process and most of the right postzygapophysis, the right side of the centrum and base of neural arch, and the anterior and posterior surfaces of the centrum and distal end of the neural spine are damaged. These two vertebrae are described together because of their consistent morphology. Only those characteristics that differ from or cannot be determined in the middle caudal vertebrae of positions A and B are described. The neurocentral suture is closed in both specimens.

The centrum is 1.74 times longer than its anterior height in GSI 2124. The ventral surface of the centrum possesses the same morphology as in ISIR 1120. The facets for the haemal arches are poorly developed and not subdivided. The prezygapophysis is almost horizontal and its base is not connected to the neural spine. The prespinal fossa is reduced to a deep, sub-circular opening. The postspinal fossa is absent. These features of the neural arch may indicate that GSI 2125 is a more posterior vertebra than GSI 2119. The anterior margin of the neural spine is continuously concave in lateral view and finishes anteriorly between both prezygapophyses. There is no anterior spur and the posterior surface of the neural spine possesses a sharp edge (figure 32*m*,*o*).

#### 6.5.3. Posterior caudal vertebrae

**Figure 34.**
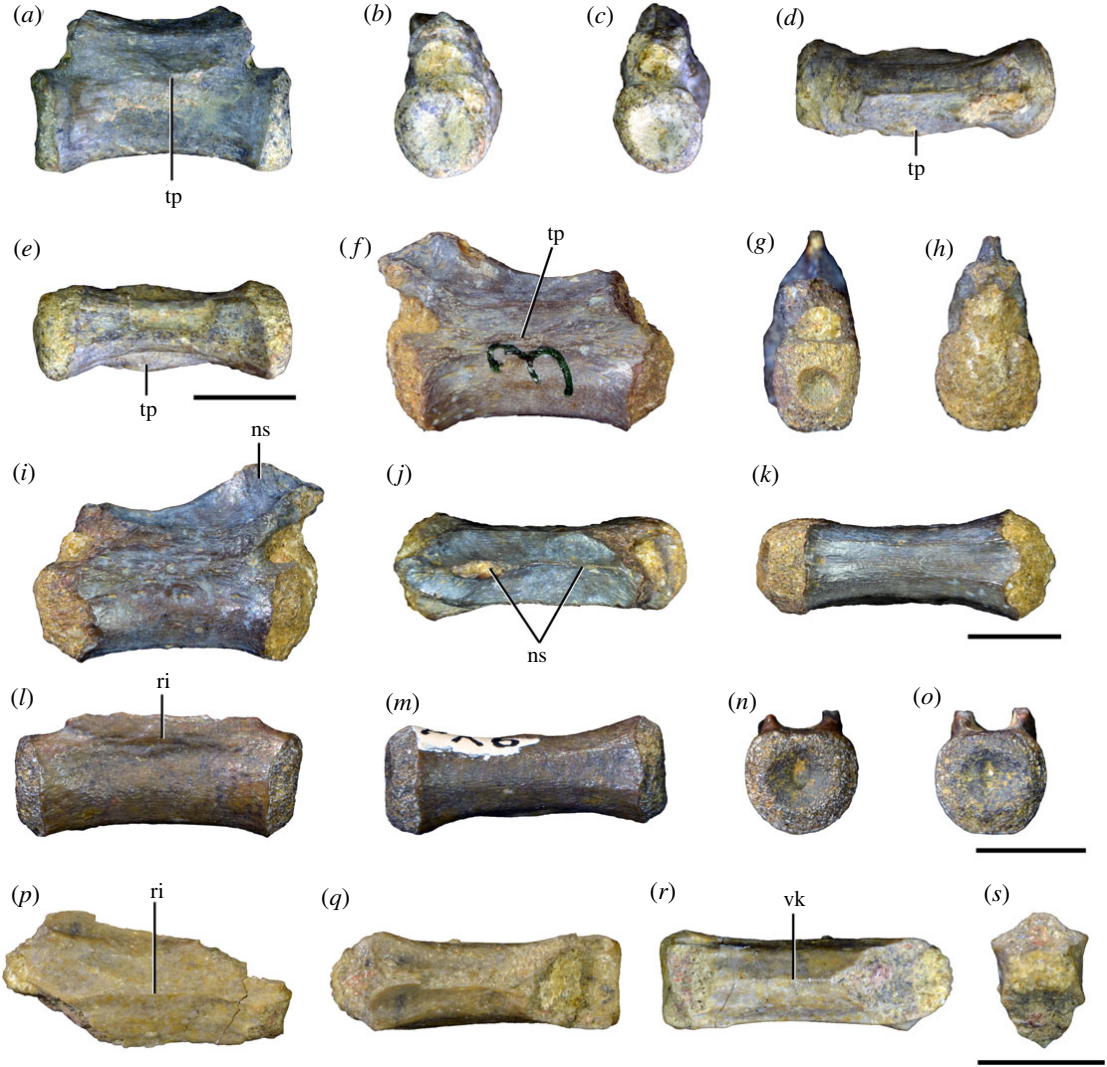
Panchet cf. proterosuchid posterior caudal vertebrae. (*a–e*) GSI 2126, (*f–k*) ISIR 1123, (*l–o*) ISIR 1121, and (*p–s*) ISIR 1122 in (*a, f, l, p*) right lateral, (*b, g, n, s*) anterior, (*c, h, o*) posterior, (*d, j, q*) dorsal, (*e, k, r*) ventral, (*i*) left lateral views. ns, neural spine; ri, ridge; tp, transverse process; vk, ventral keel. Scale bars equal 5 mm.

This region of the tail is represented by five vertebrae. GSI 2126 (Huxley [58]: plate III, figure 7; figure 34*a*–*e* and table 12) lacks the right prezygapophysis, both postzygapophyses, and most of the left prezygapophysis. The distal end of the neural spine is damaged. ISIR 1123 lacks the zygapophyses and the distal end of the neural spine (figure 34*f*–*k* and table 12). ISIR 1121 lacks most of the neural arch (figure 34*l*–*o* and table 12) and ISIR 1122 lacks the zygapophyses, the anteroventral end of the centrum, and the posterior end of the centrum and the distal margin of the neural spine is damaged (figure 34*p*–*s*). PGRU/GL/M/VF-003 is almost complete, lacking the distal end of the neural spine and having some damage on the tip of the zygapophyses and anterior and posterior margins of the centrum [46].

The centrum is 2.49 times longer than its anterior height in GSI 2126, 2.69 times in ISIR 1121, and *ca* 2.25 in PGRU/GL/M/VF-003, being considerably more elongated than the middle caudal vertebrae. These ratios resemble those of the posterior caudal vertebrae of the ‘Arcadia proterosuchian’ (2.34: QMF9536; 3.00: QMF9537) and ‘*Chasmatosaurus*’ *yuani* (2.15: IVPP V2719). By contrast, as occurs in other regions of the vertebral column, the posterior caudal vertebrae of *Erythrosuchus africanus* are proportionally shorter (1.53: NHMUK PV R3592). The anterior and posterior articular surfaces of the centrum are placed at the same dorsoventral level and are concave. These surfaces are slightly dorsoventrally taller than broad in GSI 2126 and PGRU/GL/M/VF-003, and the opposite is the case in ISIR 1121. Both surfaces possess a centrally placed notochordal pit. The centrum is slightly constricted transversely at mid-length. The ventral surface of the centrum is mainly flat and separated from the dorsoventrally convex lateral surface by a longitudinal change in slope in GSI 2126 (figure 34*k*), ISIR 1121 (figure 34*m*) and PGRU/GL/M/VF-003, resembling the condition in the ‘Arcadia proterosuchian’ (QMF9536, 9537), but in the latter specimens the median surface between the changes in slope is transversely concave. By contrast, ISIR 1122 possesses a low and sharp median ventral keel (figure 34*r*: vk), as occurs in a posterior caudal vertebra of ‘*Chasmatosaurus*’ *yuani* (IVPP V2719). The facets for articulation with the haemal arch are low. The neurocentral suture is closed in all the specimens. The transverse process is strongly reduced or absent. When the transverse process is absent there is instead a horizontal, rugose ridge positioned level with the dorsal margin of the centrum (figure 34: tp, ri). The base of the prezygapophysis is mainly parallel to the sagittal axis and slightly anterodorsally oriented in lateral view (PGRU/GL/M/VF-003). There is a thin ridge that extends posteriorly from the base of the prezygapophysis along the lateral surface of the neural arch in ISIR 1123. This ridge finishes before reaching the base of the postzygapophysis and is absent in ISIR 1122, but is present in the ‘Arcadia proterosuchian’ (QMF9536, 9537). The neural spine is very narrow and low along its entire preserved length. The dorsal margin is concave in lateral view and the highest point of the spine is placed at its posterior end. PGRU/GL/M/VF-003 possesses a low and rounded spur on the anterior region of the neural spine.

### 6.6. Intercentra

The presence of intercentra cannot be determined in *Samsarasuchus pamelae*, nor in the Panchet cf. proterosuchid specimens, because the axial elements are preserved in isolation. However, in one second sacral vertebra there is an intercentrum fused to the anteroventral margin of its centrum (NHMUK PV R37579; figure 27*a*,*d*–*f*: int). There is no trace of separation between the intercentrum and centrum in this specimen, but the identification of the former is based on its shape and anterior extension distinctly beyond the anterior margin of the centrum. The intercentrum is oval in ventral view, with a transverse main axis and crescent-shaped in anterior view, with a transversely convex ventral surface (figure 27*a*).

### 6.7. Appendicular skeleton

#### 6.7.1. Humerus

**Figure 35.**
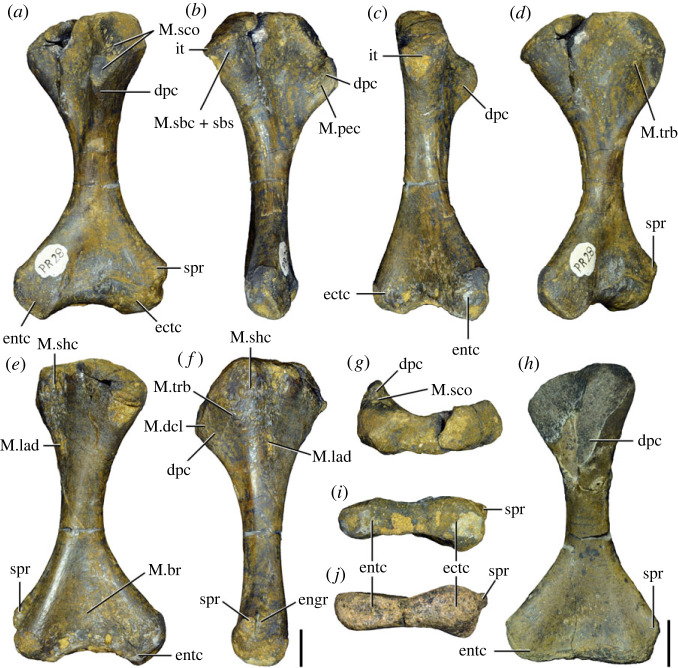
Panchet proterosuchid left humeri. (*a–g*) ISIR 1128 and (*j, h*) ISIR 1127 in (*a, h*) ventral, (*b*) medial, (*c*) dorsomedial, (*d*) ventromedial, (*e*) dorsal, (*f*) lateral, (*g*) proximal, and (*i, j*) distal views. dpc, deltopectoral crest; ectc, ectepicondyle; engr, entepicondylar groove; entc, entepicondyle; it, internal tuberosity; M.br, *M. brachialis*; M.dcl, *M. deltoideus clavicularis*; M.lad, *M. latissimus dorsi*; M.pec, *M. pectoralis*; M.sbc + sbs, *Mm. subcoracoideus* and *subscapularis*; M.sco, *M. supracoracoideus* complex; M.shc, *M. scapulohumeralis caudalis*; M.trb, *M. triceps brevis*; spr, supinator process. Scale bars equal 1 cm.

The Panchet proterosuchid humerus is represented by one complete (ISIR 1128; figure 35*a*–*i* and table 13) and one partial (ISIR 1127; figure 35*j*,*h* and table 13) left humerus, whereas a partial proximal half of a right humerus (ISIR 1129) and a distal half of a left humerus (PGRU/GL/M/VF-001; [46]) are assigned to cf. Proterosuchidae. Only some portions of the medial edge of the proximal end present some damage in ISIR 1128. ISIR 1127 lacks most of the deltopectoral crest and the distal surface of the bone is damaged. ISIR 1129 lacks most of the deltopectoral crest and the proximal surface is damaged. The four specimens possess a consistent morphology. ISIR 1128 is approximately 30% bigger than ISIR 1127 and the muscle scars of the former specimen are more developed. PGRU/GL/M/VF-001 has a size intermediate between the latter two specimens.

**Table 13.** Measurements in millimetres of Panchet proterosuchid left humeri. Values with an asterisk indicate incomplete measurements (owing to post-mortem damage) and the value given is the maximum measurable. The maximal deviation of the callipers is 0.02 mm, but measurements were rounded to the nearest 0.1 mm.

	ISIR 1127	ISIR 1128
length	63.8*	82.7
proximal width	31.6	39.6
proximal depth	10.7	14.6
length deltopectoral crest	26.2	37.2
minimum width of shaft	9.1	11.6
distal width	31.2	42.3
distal depth	11.4	15.3

**Figure 36.**
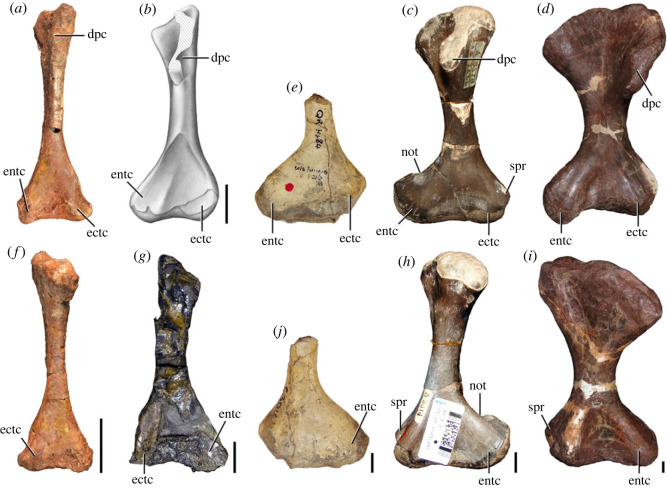
Comparison between humeri of selected Early Triassic non-eucrocopodan crocopods. (*a, f*) *Prolacerta broomi* (BP/1/2675), (*b*) *Antarctanax shackletoni* (modified from Peecook *et al.* [109]: figure 7), (*e, j*) *Proterosuchus alexanderi* (NMQR 1484, holotype), (*c, h*) ‘*Chasmatosaurus*’ *yuani* (IVPP V2719, reversed), (*d, i*) *Garjainia prima* (PIN 951/37, reversed), and (*g*) *Tasmaniosaurus triassicus* (UTGD 54655, holotype) in (*a–d*) ventral and (*f–i*) dorsal views. dpc, deltopectoral crest; ectc, ectepicondyle; entc, entepicondyle; not, notch; spr, supinator ridge. Scale bars equal 1 cm.

The proximal and distal ends of the humerus are strongly transversely expanded, being subequal in transverse width in the smaller specimen (ISIR 1127) and the distal end proportionally more expanded in the larger specimen (ISIR 1128). In ISIR 1128, the proximal and distal widths represent 0.48 and 0.51 times the total length of the bone, respectively. The ratio of proximal expansion closely resembles that of ‘*Chasmatosaurus*’ *yuani* (0.48: IVPP V2719, 0.50: IVPP V4067) and is lower in *Cuyosuchus huenei* (0.41: MCNAM 2669). By contrast, the proximal end of the humerus is considerably more transversely expanded in erythrosuchids (e.g. *Erythrosuchus africanus*: 0.70: SAM-PK-905; *Garjainia prima*: 0.57: 951/36-1; *Garjainia madiba*: 0.63: BP/1/5360; *Shansisuchus shansisuchus*: 0.58–0.62: Young [146]: table 7). The proximal end is symmetrically expanded transversely, whereas the entepicondyle is slightly more expanded than the ectepicondyle on the distal end of the bone. The main axis of the proximal end is rotated approximately 48° with respect to the main axis of the distal end in the two more complete specimens (figure 35), closely resembling the condition in ‘*Chasmatosaurus*’ *yuani* (IVPP V2719; figure 36*c*,*h*), whereas a higher degree of torsion (*ca* 70° or higher) is present in *Prolacerta broomi* (BP/1/2675; figure 36*a*,*f*), *Tasmaniosaurus triassicus* (UTGD 54655: tibia B of Ezcurra [158], here reinterpreted as a left humerus, see Discussion; figure 36*g*) and *Antarctanax shackletoni* ([109]; figure 36*b*). By contrast, the main axes of the proximal and distal ends of the humerus of *Cuyosuchus huenei* (MCNAM PV 2669) and erythrosuchids (e.g. *Guchengosuchus shiguaiensis*: [163]; *Erythrosuchus africanus*: [141]; *Garjainia prima*: [140]; figure 36*d*,*i*) are sub-parallel to each other. The humerus is crescent-shaped in proximal view, with a continuously concave ventral margin (figure 35*g*). The dorsal margin of the proximal end is convex on its medial half and laterally becomes shallowly concave. The proximal articular surface of the bone is rugose and possesses a shallow longitudinal depression along its entire extension. This depression is deeper in the smaller individual (ISIR 1127), probably because of a lower degree of ossification. The internal tuberosity is not offset from the proximal margin of the bone and is situated distal to the point at which the deltopectoral crest merges with the proximal articular surface (figure 35*b*,*c*: it). The dorsolateral corner of the humerus is continuously convex in proximal view, as occurs in *Cuyosuchus huenei* (MCNAM PV 2669), *Garjainia prima* (Maidment *et al.*, 2020), *Erythrosuchus africanus* (SAM-PK-905), and *Shansisuchus shansisuchus* [146]. By contrast, the humerus of ‘*Chasmatosaurus*’ *yuani* possesses a large, subtriangular and dorsolaterally oriented projection on this region of the bone (IVPP V2719).

The deltopectoral crest of the Panchet proterosuchid specimens extends along 45% of the total length of the bone in the larger specimen (ISIR 1128; figure 35*a*–*c*,*f*,*g*: dpc) and approximately 40% in the smaller one (ISIR 1127; figure 35*h*: dpc), resembling the condition in other early archosauriforms (e.g. ‘*Chasmatosaurus*’ *yuani*: 37%: IVPP V2719, 42%: IVPP V4067; *Cuyosuchus huenei*: 48%: Rusconi [162]: figure 38*c*; *Garjainia prima*: 48%: PIN 951/36-1; *Garjainia madiba*: 43%: BP/1/5360; *Shansisuchus shansisuchus*: 40–48%: Young [146]: table 7). By contrast, the deltopectoral crest is more distally extended along the shaft in *Erythrosuchus africanus* (55%: SAM-PK-905). The deltopectoral crest is mainly ventrally oriented in the preserved Panchet proterosuchid specimens. The base of the deltopectoral crest possesses a slight medial bowing and, as a result, its lateral surface is shallowly concave, as occurs in other early archosauriforms (e.g. *Antarctanax shackletoni*: [109]; ‘*Chasmatosaurus*’ *yuani*: IVPP V2719; *Garjainia prima*: [140]; *Erythrosuchus africanus*: SAM-PK-905). This concave lateral surface has a distinct muscle insertion area, probably homologous to the insertion area of the *M. deltoideus clavicularis* of extant crocodiles ([164]; figure 35*f*: M.dcl). This surface extends onto the dorsolateral surface of the bone as a massive muscle scar and reaches proximally the margin of the proximal articular surface of the bone. This scar probably represents the area of origin of the *M. triceps brevis* ([140]; figure 35*f*: M.trb). The distal margin of the deltopectoral crest possesses a thick tuberosity that finishes at the apex of the process as a flat, proximoventrally facing surface, resembling the condition in several non-eucrocopodan archosauromorphs [18]. Immediately proximal to the apex of the deltopectoral crest, there are several deep pits and ridges. This surface is the insertion area of the *M. supracoracoideus* complex and, more proximally, the *M. coracobrachialis brevis dorsalis* in extant crocodiles ([164]; figure 35*a*,*g*: M.sco). The medial surface of the deltopectoral crest is proximodistally concave proximally and becomes convex distally, along the surface of the tuberosity. The latter surface possesses a distinct muscle scar formed by thin striations, on which inserts the *M. pectoralis* in extant crocodiles ([164]; figure 35*b*: M.pec). The base of the deltopectoral crest and the dorsal surface of the proximal end of the bone are separated by a sharp change in slope, which is more angled in the smaller specimen. Along this change in slope, there is a striated surface that reaches proximally the articular surface of the bone and probably represents proximally the insertion area of the *M. scapulohumeralis caudalis* proximally and distally the insertion of the *M. latissimus dorsi* ([140]; figure 35*e*,*f*: M.shc, M.lad). The ventromedial corner of the proximal end of the humerus is slightly striated, probably indicating the insertion area of the *Mm. subcoracoideus* and *subscapularis* ([140]; figure 35*b*: M.sbc + sbs).

**Figure 37.**
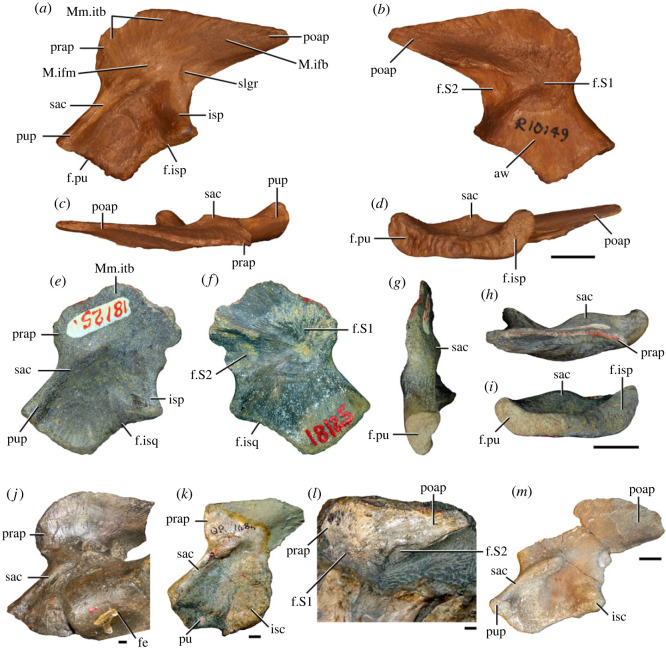
Panchet proterosuchid left ilia (*a*–*i*) and comparison with ilia of selected Early Triassic non-eucrocopodan archosauriforms (*j–m*). (*a–d*) NHMUK PV R10149, (*e–i*) GSI 18125, (*j*) ‘*Chasmatosaurus*’ *yuani* (IVPP V4067), (*k, l*) *Proterosuchus alexanderi* (NMQR 1484, holotype), and (*m*) *Vonhuenia friedrichi* (PIN 1025/406) in (*a, e*, *j*, *k*, *m*) lateral, (*b, f*, *l*) medial, (*c, h*) dorsal, (*d, i*) ventral, and (*g*) anterior views. aw, acetabular wall; f.isq, facet for ischium; f.pu, facet for pubis; f.S1, facet for sacral rib 1; f.S2, facet for sacral rib 2; fe, femur; isc, ischium; isp, ischial peduncle; M.ifb, *M. iliofibularis*; M.ifm, *M. iliofemoralis*; Mm.itb, *Mm. iliotibalis*; poap, postacetabular process; prap, preacetabular process; pu, pubis; pup, pubic peduncle; sac, supraacetabular crest; slgr, semilunar groove. Scale bars equal 1 cm in (*a*–*i*), and 5 mm in (*j–m*).

**Figure 38.**
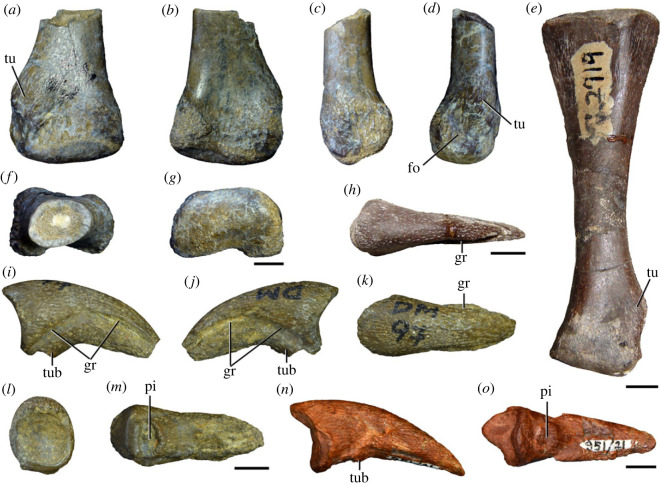
Panchet cf. proterosuchid bones of the posterior autopodium and other selected Early Triassic non-eucrocopodan archosauriforms. (*a–d, f, g*) Panchet cf. proterosuchid (ISIR 1130), (*e, h*) ‘*Chasmatosaurus*’ *yuani* (IVPP V2719), (*i–m*) Panchet cf. proterosuchid (ISIR 1131), and (*n, o*) *Garjainia prima* (PIN 951/21) in (*a, e, k*) dorsal, (*b, m, o*) ventral, (*c, d, i, j*) side, (*f, l*) proximal, (*g*) distal, and (*n*) medial views. fo, fossa; gr, groove; pi, pit; tu, tuber; tub, flexor tuber. Scale bars equal 5 mm.

The maximum constriction of the shaft occurs close to its mid-length and at this point the cross-section is oval with a transverse main axis. The ventral surface of the distal end possesses a shallowly concave depression that separates the entepicondyle from the ectepicondyle. The dorsal surface possesses a more shallowly concave and proximally extended surface that separates both distal regions, which probably represents the origin area of the *M. brachialis* ([165]; figure 35*e*: M.br). Along the transition between this concave surface and the distal articular surface of the bone there is a series of moderately large pits aligned mainly in a single row. These pits may be related to the attachment of the articular capsule of the elbow. The ventrolateral surface of the distal end possesses a thick supinator process that delimits anteriorly a shallow ectepicondylar groove (figure 35*a*,*d*,*e*,*f*,*h*: spr, engr). There is no ectepicondylar foramen, resembling the condition of most other archosauromorphs [18], but contrasting with the notch-shaped, partially closed opening of ‘*Chasmatosaurus*’ *yuani* (IVPP V2719, V4067; figure 36*c*,*h*: not). The ectepicondyle is more dorsally expanded than the entepicondyle as a result of the presence of a very thick tuberosity that runs along the dorsolateral surface of the distal half of the bone and reaches the distal margin. The entepicondyle (figure 35: entc) is moderately well developed medially, resembling the condition in *Tasmaniosaurus triassicus* (UTGD 54655; figure 36*g*) and the vast majority of early archosauriforms (e.g. *Antarctanax shackletoni*: [109]; *Cuyosuchus huenei*: MCNAM PV 2669; *Guchengosuchus shiguaiensis*: [163]; *Garjainia prima*: [140]; *Erythrosuchus africanus*: SAM-PK-905; figure 36*b*,*d*,*i*). However, the entepicondyle is considerably more abruptly projected from the shaft, forming an angle of greater than 45° with respect to the longitudinal axis of the shaft in dorsal or ventral views, in the proterosuchids *Proterosuchus alexanderi* (NMQR 1484; figure 36*e*,*j*) and ‘*Chasmatosaurus*’ *yuani* (IVPP V2719, V4067; figure 36*c*,*h*). In ventral view, the entepicondyle and ectepicondyle are subequally distally developed, as in *Proterosuchus alexanderi* (NMQR 1484; figure 36*e*), ‘*Chasmatosaurus*’ *yuani* (IVPP V2719, V4067; figure 36*c*) and *Antarctanax shackletoni* ([109]; figure 36*b*), but the entepicondyle is distinctly more distally projected than the ectepicondyle in *Tasmaniosaurus triassicus* (UTGD 54655; figure 36*g*), *Cuyosuchus huenei* (MCNAM PV 2669) and erythrosuchids (e.g. *Guchengosuchus shiguaiensis*: [163]; *Garjainia prima*: [140]; *Erythrosuchus africanus*: SAM-PK-905). The dorsal surface of the entepicondyle possesses a muscle scar composed of thick striations and may represent the origin area of the flexor musculature [165]. The distal articular surface of the bone is well ossified and very rugose, indicating the presence of a cartilaginous capping. There is a broadly transversely concave surface that separates the two distal condyles. The distal surface of the entepicondyle is formed by two transversely flat surfaces that meet in a right-angled change of slope that produced a distally projected apex. The surface of the ectepicondyle is continuously dorsoventrally convex. There are no distinct ulnar and radial condyles (=capitellum and trochlea), as occurs in most other early crocopods [18].

#### 6.7.2. Ilium

The pelvic region is represented by a left partial ilium originally reported by Satsangi [45] (GSI 18125; figure 37*e*–*i* and table 14) and a cast of a fairly complete left ilium (NHMUK PV R10149; figure 37*a*–*d* and table 14). GSI 18125 lacks most of the postacetabular process and the dorsal margin of the iliac blade is damaged, whereas NHMUK PV R10149 only lacks a small portion of the anterodorsal corner of the iliac blade. The original specimen of NHMUK PV R10149 could not be located during this study and does not represent a cast of GSI 18125 before any potential breakage, as there are minor differences in their morphology and the latter is a smaller specimen. Both ilia possess a consistent morphology between each other and are described together.

**Table 14.** Measurements in millimetres of Panchet proterosuchid left ilia. Values with an asterisk indicate incomplete measurements (owing to post-mortem damage) and the value given is the maximum measurable. The maximal deviation of the callipers is 0.02 mm, but measurements were rounded to the nearest 0.1 mm.

	GSI 18125	NHMUK PV R10149
length iliac blade	28.1*	45.3
length acetabulum	21.1	24.5
height acetabulum	24.3	29.5
length postacetabular process	—	22.6
length pubic peduncle	13.4	15.8
distal anteroposterior width pubic peduncle	4.4	6.1
maximum height	38.7*	39.2

The lateral surface of the preacetabular process and the portion of the iliac blade dorsal to the supraacetabular crest are mostly flat. A series of thin, parallel striations are present adjacent to the anterior, dorsal and posterior margins of the iliac blade. These striations converge towards the dorsal margin of the acetabulum but they do not reach it. These striations probably represent the origin areas of the *Mm. iliotibialis* ([140]; figure 37*a*,*e*: Mm.itb). The preacetabular process is anteriorly short and semi-circular in lateral view (figure 37*a*,*e*: prap), resembling the condition in *Proterosuchus alexanderi* (NMQR 1484; figure 37*k*,*l*: prap), ‘*Chasmatosaurus*’ *yuani* (IVPP V4067; figure 37*j*: prap) and *Garjainia prima* [140]. By contrast, *Cuyosuchus huenei* [162], *Erythrosuchus africanus* [141] and *Shansisuchus shansisuchus* [146] have a subtriangular preacetabular process with a distinct anterior apex in lateral or medial views. The preacetabular process of the Panchet proterosuchid specimens curves slightly medially towards its anterior tip (figure 37*c*,*h*: prap), resembling the condition in *Cuyosuchus huenei* [162], *Garjainia prima* (PIN 951/8-1), *Erythrosuchus africanus* (NHMUK PV R3592) and *Proterosuchus alexanderi* (NMQR 1484), but this condition occurs more conspicuously in the latter species. By contrast, the preacetabular process is mostly straight in ‘*Chasmatosaurus*’ *yuani* (IVPP V4067) in dorsal view. The central region of the iliac blade, immediately dorsal to the supraacetabular crest, is slightly inflated, rugose and pitted, probably indicating the origin area of the *M. iliofemoralis* ([140]; figure 37*a*: M.ifm).

The postacetabular process is subtriangular, with a tapering posterior end, and is mainly posteriorly oriented (figure 37*a*,*b*: poap), as occurs in *Proterosuchus alexanderi* (NMQR 1484; figure 37*l*: poap). *Erythrosuchus africanus* and *Shansisuchus shansisuchus* also have a tapering postacetabular process, but with a more rounded posterior tip [141,146], but the postacetabular process is more sub-rectangular in *Garjainia prima* [140], *Garjainia madiba* [153] and *Cuyosuchus huenei* [162]. The lateral surface of the postacetabular process of the Panchet proterosuchid specimen is dorsoventrally convex, contrasting with the flat anterior half of the iliac blade. This convex surface has anteroventrally-to-posterodorsally oriented striations that probably indicate the origin site of the *M. iliofibularis* ([140]; figure 37*a*: M.ifb). The length of the postacetabular process is 0.92 times the length of the acetabulum, closely resembling the ratio in ‘*Chasmatosaurus*’ *yuani* (*ca* 0.92: Young [136]: figure 10), *Cuyosuchus huenei* (0.91: Rusconi [162]: figure 31*a*), *Garjainia prima* (0.98: PIN 951/8-1) and *Erythrosuchus africanus* (0.87: NHMUK PV R3592; 0.90: SAM-PK-905). The postacetabular process is distinctly longer in *Garjainia madiba* (1.19: BP/1/5525) and *Shansisuchus shansisuchus* (1.34: Young [146]: figure 30*b*) than in the Panchet proterosuchid. The dorsoventral axis of the postacetabular process possesses a slight lateral torsion towards the posterior end of the structure. As a result, the lateral surface of the posterior portion of the process faces slightly ventrally. The ventral portion of the base of the postacetabular process, immediately dorsal to the base of the ischiadic peduncle, possesses a semilunar groove with a dorsoventral main axis on its lateral surface (figure 37*a*: slgr). This depressed surface may be associated to the origin of the *M. caudofemoralis brevis* [140].

The iliac acetabular wall is ventrally developed as a subtriangular projection with continuous and transversely thick pubic and ischial articular surfaces on its ventral margin, which indicates the presence of a fully closed acetabulum (figure 37: aw, f.isq, f.pu). The lateral surface of the acetabular wall is gently concave and possesses a distinct and anteroposteriorly extended surface restricted to approximately the ventral half of the acetabulum. This surface possesses a distinct ‘zigzag’-shaped dorsal margin that probably indicates the dorsal limit of the hyaline cartilage [166]. The supraacetabular crest is restricted to the anterodorsal corner of the acetabulum, straight and ends abruptly posteriorly as a thick, rounded structure (figure 37*a*,*e*: sac), as occurs in *Proterosuchus alexanderi* (NMQR 1484; figure 37*k*: sac), ‘*Chasmatosaurus*’ *yuani* (IVPP V4067; figure 37*j*: sac), and a specimen referred to *Vonhuenia friedrichi* (PIN 1025/406; [40]; figure 37*m*: sac). The supraacetabular crest of erythrosuchids (e.g. *Garjainia prima*: [140]; *Erythrosuchus africanus*: [141]; *Shansisuchus shansisuchus*: [146]; *Bharitalasuchus tapani*: [102]) also ends abruptly posteriorly, but it bows distinctly dorsally. In addition, *Shansisuchus shansisuchus* and *Bharitalasuchus tapani* differ from the above-mentioned taxa in the presence of a supraacetabular crest that curves posteroventrally to frame the posterodorsal corner of the acetabulum [102]. The supraacetabular crest of the Panchet proterosuchid specimens is poorly laterally developed and its lateralmost projection occurs immediately posterior to the mid-length of the acetabulum.

The pubic peduncle is moderately long and oriented at an angle of *ca* 43° and *ca* 50° with respect to the anteroposterior plane in the two available specimens, respectively. The distal end of the pubic peduncle is slightly anteroposteriorly expanded in lateral view and possesses a semi-circular articular facet, with a convex anterior margin. The anterior margin of the pubic peduncle is straight in lateral view. The ischiadic peduncle (figure 37*a*,*e*: isp) is shorter than the pubic peduncle and lacks a well-rimmed antitrochanter, as is also the case in other non-archosaurian archosauriforms [18]. The ischiadic peduncle is posterolaterally oriented in ventral view and strongly laterally projected (figure 37*d*,*i*: f.isq). As a result, the ischiadic peduncle extends more laterally than the supraacetabular crest, as occurs in *Proterosuchus alexanderi* (NMQR 1484). By contrast, the ischiadic peduncle is approximately aligned to the sagittal plane or slightly posterolaterally oriented in ventral view in erythrosuchids (e.g. *Garjainia prima*: PIN 951/8; *Erythrosuchus africanus*: NHMUK PV R3592; *Bharitalasuchus tapani*: [102]). The distal end of the ischiadic peduncle is poorly posteriorly projected, resembling the condition in the referred specimen of *Vonhuenia friedrichi* (PIN 1025/406; figure 37*m*: isp), other proterosuchids (e.g. *Proterosuchus alexanderi*: NMQR 1484; ‘*Chasmatosaurus*’ *yuani*: Young [136]: figure 10), and *Cuyosuchus huenei* (Rusconi [162]: figure 31). By contrast, *Garjainia prima* [140], *Garjainia madiba* [153] and *Erythrosuchus africanus* [141] have a strongly posteriorly projected distal end of the ischiadic peduncle, forming a distinct heel, whereas in *Shansisuchus shansisuchus* (Young [146]: figures 29 and 30) and *Bharitalasuchus tapani* [102] the ischiadic peduncle is posteroventrally oriented along its entire length. The distal articular surface of the ischiadic peduncle is flat, but possesses a few, broad striations. This articular surface is subtriangular, with a posterolaterally oriented apex.

The medial surface of the iliac blade possesses two distinct facets for articulation with the sacral ribs. The facet for the first primordial sacral has an inverted L-shaped contour, with an anteroposteriorly oriented main axis and its most depressed area situated anteroventrally (figure 37*b*,*f*: f.S1), resembling the condition in other early archosauriforms (e.g. *Garjainia prima*: [140]). This facet is delimited anteroventrally by a low ridge that merges anterodorsally with the base of the preacetabular process. The facet for the second primordial sacral rib is crescent-shaped, with a convex anterodorsal margin (figure 37*b*,*f*: f.S2). The ventral portion of this facet is positioned at the base of the ischiadic peduncle and extends dorsally onto the anteroventral region of the base of the postacetabular process, as occurs in other early archosauriforms (e.g. referred specimen of *Vonhuenia friedrichi*: PIN 1025/406; *Garjainia prima*: [140]; *Erythrosuchus africanus*: [141]). The posterior end of the facet for the second primordial sacral rib tapers strongly along the ventromedial edge of the postacetabular process and finishes approximately at the mid-length of the process, as is the case in *Proterosuchus alexanderi* (figure 37*l*: f.S2). A longitudinal, slightly posterodorsally slanting, ridge separates both facets along the base of the postacetabular process and part of the central region of the iliac wall. The surfaces of the facets for the sacral ribs are finely striated. The medial surface of the acetabular wall is flat, with a posteroventrally-to-anterodorsally oriented low and rounded tuberosity situated at the level of the posterior margin of the acetabulum, as occurs in the referred specimen of *Vonhuenia friedrichi* (PIN 1025/406).

#### 6.7.3. Foot

The posterior autopodium is represented by the distal end of a fourth metatarsal (ISIR 1130; figure 38*a*–*d*,*f*,*g* and table 15) and an ungual pahalanx (ISIR 1131; figure 38*i*–*m* and table 15). The morphology of these bones closely resembles those of the foot of *Proterosuchus fergusi* (SAM-PK-K140) and ‘*Chasmatosaurus*’ *yuani* (IVPP V2719; figure 38*e*). The distal end of the metatarsal is 1.5 times broader transversely than it is dorsoventrally deep and is asymmetric, with one condyle dorsoventrally lower than the other, as occurs in the fourth metatarsal of *Proterosuchus fergusi* (SAM-PK-K140: mtt IV ratio = 1.5). By contrast, the distal end of the metatarsal III of *Proterosuchus fergusi* is symmetric and the ratio between the transverse width and dorsoventral depth is lower in the second and third metatarsals (SAM-PK-K140: mtt II−III ratio = 1.3). Only the medial surface of the distal end of the fourth metatarsal possesses a collateral fossa in *Proterosuchus fergusi* (SAM-PK-K140) and only one side has a fossa in ISIR 1130. Thus, this line of evidence may suggest that ISIR 1130 is a left element. On the other hand, there is a very well developed tuber placed immediately proximodorsal to this collateral groove and an equivalent tuber is present on the lateral side of the metatarsals of *Erythrosuchus africanus* (BP/1/2096). This would indicate that ISIR 1130 is a right side element. As a consequence, we consider the assignment of ISIR 1130 to the right or left side as ambiguous. The presence of a tuber immediately proximodorsal to the distal end of the metatarsals (figure 38*a*,*d*: tu) also occurs in ‘*Chasmatosaurus*’ *yuani* (IVPP V2719; figure 38*e*: tu), but not in *Proterosuchus fergusi* (SAM-PK-K140). The distal end of the Panchet cf. proterosuchid metatarsal is symmetrically transversely expanded with respect to the shaft. The shaft is slightly transversely broader than deep where it is broken off (figure 38*f*). The dorsal surface of the bone lacks a distinct extensor fossa, as occurs in the metatarsals of *Proterosuchus fergusi* (SAM-PK-K140), ‘*Chasmatosaurus*’ *yuani* (IVPP V2719), *Garjainia prima* (PIN 951), and *Erythrosuchus africanus* (BP/1/2096). As mentioned, the lateral surface of the bone lacks a collateral fossa and there are several thick longitudinal striations in this area. The distal articular surface possesses two condyles that are broadly separated from each other on the ventral surface but not on the distal surface (figure 38*a*,*b*,*g*). Both condyles are subequally transversely broad, but the medial condyle is dorsoventrally lower than the lateral one. The medial condyle possesses a convex ventral margin in distal view, whereas the lateral condyle possesses a squared profile.

**Table 15.** Measurements in millimetres of Panchet cf. proterosuchid posterior autapodial bones. Metatarsal IV (ISIR 1130) and ungual phalanx (ISIR 1131). Values with an asterisk indicate incomplete measurements (owing to post-mortem damage) and the value given is the maximum measurable. The maximal deviation of the callipers is 0.02 mm, but measurements were rounded to the nearest 0.1 mm.

	Mtt IV	ungual
length	26.6*	21.1*
proximal height	—	11.5
proximal width	—	9.9
distal height	12.4	—
distal width	18.9	—

The ungual pahalanx (ISIR 1131) lacks its distal tip and closely resembles those of ‘*Chasmatosaurus*’ *yuani* (IVPP V2719; figure 38*h*), *Proterosuchus fergusi* (SAM-PK-K140), and *Garjainia prima* ([140]; figure 38*n*,*o*). In particular, when compared with the other most abundant tetrapods of the Panchet Formation, ISIR 1131 differs from dicynodont unguals (e.g. *Lystrosaurus georgi*: [167]) in being transversely narrower, dorsoventrally deeper, and more ventrally curved. ISIR 1131 is less ventrally curved than the manual unguals of *Proterosuchus fergusi* (SAM-PK-K140) and ‘*Chasmatosaurus*’ *yuani* (IVPP V4067), but resembles the degree of curvature of the pedal unguals of these same specimens. As a result, ISIR 1131 is interpreted as a pedal ungual. ISIR 1131 is continuously ventrally curved and its most distally preserved portion extends ventrally to the level of the flexor tubercle. The dorsal surface of the claw is transversely convex and unkeeled. The ventral surface is transversely flat. These surfaces possess multiple low striations and pits, resembling the condition of the unguals of ‘*Chasmatosaurus*’ *yuani* (IVPP V2719). The flexor tubercle is poorly ventrally developed and possesses a proximoventrally facing pit (figure 38*i*,*j*,*o*: tub, pi). The latter condition closely resembles that of a pedal ungual of *Garjainia prima*, but its tubercle is lower than in the Indian specimen (Maidment *et al.* [140]: figure 24*e–g*; figure 38*n*,*o*: tub, pi). The Panchet cf. proterosuchid ungual is moderately transversely compressed and symmetric in dorsal view, as in other early archosauriforms (e.g. *‘Chasmatosaurus*’ *yuani*: IVPP V2719; *Garjainia prima*: [140]). A well-defined, ventrally curved collateral groove is present on each side of the claw and is confluent with the proximoventral corner of the bone (figure 38*i*–*k*: gr). As a result, the collateral groove separates the flexor tubercle from the articular surface of the bone in side views. The portion of the ungual ventral to the collateral groove is slightly more transversely expanded than the dorsal one, thus forming low collateral platforms (figure 38*k*), resembling the condition in *Garjainia prima* (Maidment *et al.* [140]: figure 24*e–g*). The proximal articular surface is dorsoventrally concave and slightly dorsoventrally deeper than broad. There is no proximodorsal lip.

## 7. Phylogenetic analyses

### 7.1. Analysis 1

**Figure 39.**
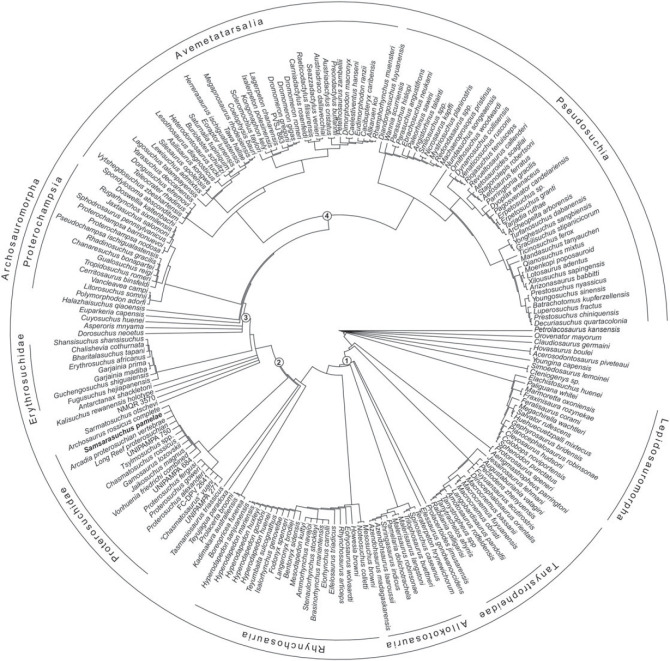
Strict consensus tree of 250 000 most parsimonious trees found in Analysis 1 under implied weighting with *k* = 20. *Samsarasuchus pamelae* is indicated with bold font. Numered clades are as follows: 1, Crocopoda; 2, Archosauriformes; 3, Eucrocopoda; 4, Archosauria.

The analyses using the different concavity constant values (*k* = 19–24) recovered more than 250 000 most parsimonious trees (MPTs)—the maximum number of trees set to be stored in memory—in all cases (*k* = 19: fit of 197.31479, CI of 0.18248 and RI of 0.63889; *k* = 20: fit of 190.15540, CI of 0.18248 and RI of 0.63889; *k* = 21: fit of 183.50984, CI of 0.18267 and RI of 0.63935; *k* = 22: fit of 177.31872, CI of 0.18267 and RI of 0.63935; *k* = 23: fit of 171.54123, CI of 0.18267 and RI of 0.63935; *k* = 24: fit of 166.13645, CI of 0.18267 and RI of 0.63935). Beyond the terminals added here, the strict consensus trees (SCTs; figure 39 and electronic supplementary material, figures S1–S6) generated from each set of MPTs show a topology mostly congruent with that found by the analysis of Ezcurra & Sues [100] under implied weighting with a *k* = 10 (which was the highest k value used by these authors). The only exceptions are the position of *Hyperodapedon mariensis* at the base of the genus *Hyperodapedon* (*k* = 21–24), *Teyumbaita sulcognathus* as the sister taxon to *Hyperodapedon huenei* (*k* = 21–24), *Cuyosuchus huenei* as an early diverging eucrocopod (all *k* values; not specified hereafter if found using all *k* values), *Halazhaisuchus qiaoensis* as the sister taxon to Proterochampsia + Archosauria (*k* = 19–20), *Sphodrosaurus pennsylvanicus* as the sister taxon to the genus *Proterochampsa* at the base of Proterochampsidae (*k* = 21–24), a *Polymorphodon adorfi* + (*Litorosuchus somnii* + *Vancleavea campi*) clade as sister taxon to Proterochampsidae (*k* = 19–20), *Scleromochlus taylori* at the base of Lagerpetidae, *Mandasuchus tanyauchen* as the sister taxon to Paracrocodylomorpha, *Arizonasaurus babbitti* as the sister taxon to *Xilousuchus sapingensis*, *Youngosuchus sinensis* as the sister taxon to *Prestosuchus nyassicus* at the base of Loricata, and *Batrachotomus kupferzellensis* as the sister taxon to a clade composed of *Luperosuchus fractus* + (*Prestosuchus chiniquensis* + *Decuriasuchus quartacolonia*). As a result, the overall topology of the SCTs from these analyses will not be described in detail.

The new species *Samsarasuchus pamelae* is recovered within a Proterosuchidae composed of additional 16 terminals: *Proterosuchus fergusi*, *Proterosuchus alexanderi*, *Proterosuchus goweri*, *Archosaurus rossicus*, ‘*Chasmatosaurus*’ *yuani*, *Vonhuenia friedrichi*, *Jaikosuchus magnus*, *Gamosaurus lozovskii*, *Chasmatosuchus rossicus*, *Tsylmosuchus* spp., UNIPAMPA 271, UNIPAMPA 684, UNIPAMPA 750, FC-DPV 2641, the Long Reef proterosuchian and the Arcadia proterosuchian vertebrae (figure 39, table 16). The analyses optimized the following 11 character states as synapomorphies of Proterosuchidae (those states preserved in *Samsarasuchus pamelae* indicated with an asterisk): premaxilla with anteroposteriorly deep base of the prenarial process (character 35: 0 → 1); premaxilla with lateroventrally opening anterior alveoli in mature individuals (character 44: 0 → 1); parietal with supratemporal fossa well exposed in dorsal view and mainly dorsally or dorsolaterally facing (character 161: 1 → 0); parietals with median posterior projection in dorsal view (character 854: 1 → 0); parabasisphenoid with posterolaterally oriented basipterygoid processes (character 248: 0 → 1); lower jaw with narrow symphyseal space and well organized rugosities (class II of Holliday & Nesbitt [159]) (character 859: 0 → 1); dentary with anterior end of the bone distinctly transversely broader than at level of or posterior to the sixth tooth position in dorsal or ventral view (character 891: 0 → 1); posterior cervical, anterior dorsal, and sometimes middle dorsal vertebrae with a thick, mainly vertical tuberosity immediately ventral to the transverse process (character 319: 0 → 1*); dorsal vertebrae with mammillary processes of the neural spines extended up to the thirteenth presacral vertebra or beyond (character 365: 2 → 3/4*); middle caudal vertebrae with accessory laminar process on the anterior face of the neural spine (character 380: 0 → 1); and ilium with main axis of the ventral articular surface of the ischiadic peduncle posteroventrally oriented in ventral view as a result of a strong lateral projection of the peduncle, in which its lateralmost point exceeds that of the supraacetabular crest (character 908: 0 → 1).

**Table 16.** Taxonomic reassessments of the non-erythrosuchid, non-eucrocopod archosauriform terminals included in our phylogenetic analyses.

specimen/taxon	least inclusive previous taxonomic assignment	least inclusive taxonomic assignment preferred here
*Antarctanax shackletoni*	non-proterosuchid, non-erythrosuchid, non-eucrocopod Archosauriformes	non-proterosuchid, non-erythrosuchid, non-eucrocopod Archosauriformes
Arcadia proterosuchian vertebrae	non-eucrocopodan Archosauriformes	Proterosuchidae -Chasmatosuchinae
*Archosaurus rossicus*	Proterosuchidae	Proterosuchidae -Chasmatosuchinae
*Chasmatosuchus rossicus*	Proterosuchidae/non-eucrocopod Archosauriformes	Proterosuchidae - Chasmatosuchinae
‘*Chasmatosuchus' vjushkovi*	Proterosuchidae/non-erythrosuchid, non-eucrocopod Archosauriformes	non-archosauriform Eucrocopoda/non-chasmatosuchine Proterosuchidae/non-erythrosuchid, non-eucrocopod Archosauriformes
FC-DPV 271	Proterosuchidae	Proterosuchidae - *Proterosuchus* sp.
*Gamosaurus lozovskii*	Proterosuchidae/non-eucrocopod Archosauriformes	Proterosuchidae -Chasmatosuchinae
*Jaikosuchus magnus*	?‘Rauisuchia’/non-eucrocopod Archosauriformes/*Chasmatosuchus*	Proterosuchidae -Chasmatosuchinae
*Kalisuchus rewanensis*	non-proterosuchid, non-erythrosuchid, non-eucrocopod Archosauriformes	non-proterosuchid, non-erythrosuchid, non-eucrocopod Archosauriformes
Long Reef proterosuchian	Proterosuchidae/non-eucrocopod Archosauriformes	Proterosuchidae -Chasmatosuchinae
NMQR 3570	Non-proterosuchid, non-erythrosuchid, non-eucrocopod Archosauriformes	Non-proterosuchid, non-erythrosuchid, non-eucrocopod Archosauriformes
*Proterosuchus alexanderi*	Proterosuchidae	Proterosuchidae - *Proterosuchus*
*Proterosuchus fergusi*	Proterosuchidae	Proterosuchidae – *Proterosuchus*
*Proterosuchus goweri*	Proterosuchidae	Proterosuchidae - *Proterosuchus*
‘*Chasmatosaurus' yuani*	Proterosuchidae	Proterosuchidae - *Proterosuchus*
*Samsarasuchus pamelae*	n.a.	Proterosuchidae - Chasmatosuchinae
*Sarmatosuchus otschevi*	Proterosuchidae/non-proterosuchid, non-erythrosuchid, non-eucrocopod Archosauriformes	Non-proterosuchid, non-erythrosuchid, non-eucrocopod Archosauriformes
*Tsylmosuchus* spp.	?‘Rauisuchia’/Proterosuchidae	Proterosuchidae -Chasmatosuchinae
UNIPAMPA 271	cf. *Proterosuchus*	*Proterosuchus* sp.
UNIPAMPA 684	cf. *Proterosuchus*	Proterosuchidae - Chasmatosuchinae
UNIPAMPA 750	cf. *Chasmatosuchus*	Proterosuchidae -Chasmatosuchinae
*Vonhuenia friedrichi*	Proterosuchidae/non-proterosuchid, non-eucrocopod Archosauriformes	Proterosuchidae -Chasmatosuchinae

**Figure 40.**
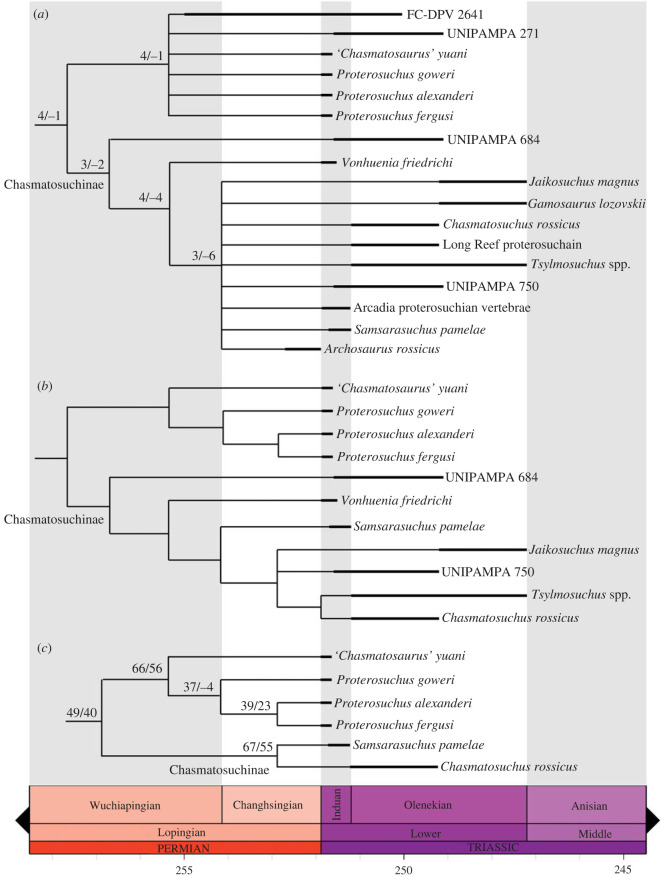
Time-calibrated consensus subtrees of Proterosuchidae recovered in Analysis 1 under implied weighting with *k* = 20. (*a*) Strict consensus tree; (*b*) SRCT after the *a posteriori* pruning of FC-DPV 2641, UNIPAMPA 271, *Gamosaurus lozovskii*, *Archosaurus rossicus*, the Arcadian proterosuchian vertebrae, and the Long Reef proterosuchian; and (*c*) SRCT after the a posteriori pruning of the terminals pruned in (*b*) and UNIPAMPA 684 and 750, *Vonhuenia friedrichi*, *Jaikosuchus magnus*, *Tsylmosuchus* spp., *Kalisuchus rewanensis*, and NMQR 3570. Values above each non-terminal branch in (*a* and *c*) are the absolute (left) and GC (Group present/contradicted) (right) bootstrap frequencies.

The SCTs show two main groups within Proterosuchidae (figures 39 and 40*a* and electronic supplementary material, figures S1–S6). The first clade is composed of the three South African species of *Proterosuchus*, ‘*Chasmatosaurus*’ *yuani*, an isolated basioccipital from the Permo-Triassic Buena Vista Formation of Uruguay (FC-DPV 271; [13,32]), and an isolated probable D4−D5 from the Lower Triassic Sanga do Cabral Formation of Brazil previously assigned to cf. *Proterosuchus* (UNIPAMPA 271; [110]). This group is considered here to represent the genus *Proterosuchus* (including ‘*Chasmatosaurus*’) and possesses the following four synapomorphies: posterior cervical, anterior dorsal and sometimes anterior–middle cervical and middle–posterior dorsal vertebrae without prezygodiapophyseal lamina (character 317: 1 → 0); coracoid with postglenoid process tapering posteriorly in lateral view (character 403: 0 → 1); fourth to sixth cervical vertebrae with neural spine distinctly anterodorsally canted, with the top of the neural spine anterodorsally oriented and parallel anterior and posterior margins of the neural spine in lateral view (character 767: 0 → 1); and tibia with proximal posterior hemicondyles separated by a distinct change in angle or shallow notch or notches in proximal view (character 810: 0 → 1).

The relationships within the genus *Proterosuchus* are unresolved in the SCTs, but the iterPCR protocol [168] found that UNIPAMPA 271 is alternatively recovered in all possible positions within this clade to the exclusion of a sister taxon relationship with *Proterosuchus alexanderi* and *Proterosuchus fergusi*. The a posteriori pruning of UNIPAMPA 271 allows the recovery of ‘*Chasmatosaurus*’ *yuani* as the sister taxon to the clade composed of the three South African species of *Proterosuchus* and FC-DPV 271. This latter clade is supported by three synapomorphies: parietals with pineal fossa on the median line of their dorsal surface (character 162: 0 → 1), basioccipital lacking or with extremely short occipital neck (character 231: 0 → 1), and basioccipital with ventrally projected basal tubera that are parallel to each other (character 233 0 → 1). In addition, the a posteriori pruning of FC-DPV 271 resolves a group composed of *Proterosuchus alexanderi* and *Proterosuchus fergusi*. The clade formed by these two species has two synapomorphies: antorbital fenestra length twice its height or less (character 697: 0 → 1); and posterior cervical, anterior dorsal, and sometimes middle dorsal vertebrae without a thick, mainly vertical tuberosity immediately below the transverse process (character 319: 1 → 0; represents a reversal of the plesiomorphic condition of Proterosuchidae and it is present in only some MPTs).

The second main clade within Proterosuchidae includes an isolated middle–posterior dorsal vertebra from the Sanga do Cabral Formation of Brazil (UNIPAMPA 684) and a group composed of *Vonhuenia friedrichi* and a large polytomy formed by *Samsarasuchus pamelae*, *Jaikosuchus magnus*, *Gamosaurus lozovskii*, *Chasmatosuchus rossicus*, *Tsylmosuchus* spp., *Archosaurus rossicus*, an isolated cervical vertebra from the Sanga do Cabral Formation of Brazil (UNIPAMPA 750), the Long Reef proterosuchian vertebrae and the Arcadia proterosuchian vertebrae (figure 40*a* and electronic supplementary material, figures S1–S6). All this clade is named here Chasmatosuchinae (see Systematic Palaeontology) and is supported by the following five synapomorphies, which are not necessarily preserved in *Vonhuenia friedrichi* (*ca* 85% of missing data) and/or UNIPAMPA 684 (*ca* 98% of missing data) (indicated with an asterisk because they are present in *Samsarasuchus pamelae*): anterior–middle and sometimes posterior postaxial cervical vertebrae with distally restricted transverse expansion of the neural spines (not mammillary process) (character 321: 0 → 1*; unknown in *Vonhuenia friedrichi* and UNIPAMPA 684); ninth presacral centrum with a ventral keel (character 892: 0 → 1*; unknown in UNIPAMPA 684); third to eighth or ninth presacral vertebrae with diagonal, anterodorsally-to-posteroventrally oriented ridge that reaches the base of the prezygapophysis and it is not connected to the diapophysis on the lateral surface of the neural arch (character 895: 0 → 1*; unknown in UNIPAMPA 684); fourth to eight presacral vertebrae with posterior expansion of the neural spine, resulting in a posterodorsally tilted posterior margin in an angle higher than 15° with respect to the anterior margin of the neural spine in lateral view (character 901: 0 → 1*; unknown in *Vonhuenia friedrichi* and UNIPAMPA 684); and at least some middle and posterior dorsal vertebrae with a single ventral keel on the centrum (character 353: 0 → 2*; unknown in *Vonhuenia friedrichi*). The clade that includes all chasmatosuchines to the exclusion of UNIPAMPA 684 has the following synapomorphy (indicated with an asterisk because the character state is present in *Samsarasuchus pamelae*): posterior cervical, anterior dorsal, and sometimes anterior–middle cervical and middle–posterior dorsal vertebrae with postzygodiapophyseal lamina (character 318: 0 → 1*). Finally, the group that is composed of all chasmatosuchines to the exclusion of *Vonhuenia friedrichi* and UNIPAMPA 684 has the following two synapomorphies (indicated with an asterisk because they are present in *Samsarasuchus pamelae*): posterior cervical, anterior and sometimes middle–posterior dorsal vertebrae with posterior centrodiapophyseal lamina (character 316: 0 → 1*); and fourth to sixth cervical vertebrae with strongly developed longitudinal lamina or tuberosity extended posteriorly from the base of the transverse process, flaring laterally as a prominent and thick, wing-like shelf (character 334: 0 → 1*).

In a second round of pruning, the iterPCR protocol found that *Archosaurus rossicus*, *Gamosaurus lozovskii*, and the Arcadia proterosuchian vertebrae acted as wildcards within Chasmatosuchinae. *Gamosaurus lozovskii* is alternatively nested in all possible positions within Chasmatosuchinae, *Archosaurus rossicus* is found as sister taxon to *Chasmatosuchus rossicus*, *Tsylmosuchus* spp., *Samsarasuchus pamelae*, and the Long Reef proterosuchian, and the Arcadia proterosuchian vertebrae are found as sister taxon to *Samsarasuchus pamelae*, *Jaikosuchus magnus*, and UNIPAMPA 750. The *a posteriori* exclusion of these three topologically unstable terminals resulted in the recognition of *Samsarasuchus pamelae* as the sister taxon to a clade of unresolved internal relationships that includes *Jaikosuchus magnus*, *Chasmatosuchus rossicus*, *Tsylmosuchus* spp., the Long Reef proterosuchian, and UNIPAMPA 750. This clade possesses the following synapomorphy: third to fifth cervical vertebrae with maximum height of neural spine versus height of posterior articular surface of centrum of 1.40–3.0 (character 342: 3 → 4&5&6*). Finally, a third round of pruning after the additional exclusion of the Long Reef proterosuchian recovered a sister taxon relationship between *Chasmatosuchus rossicus* and *Tsylmosuchus* spp. (figure 40*b*), which is supported by the presence of third to fifth cervical vertebrae with maximum height of neural spine versus height of posterior articular surface of centrum of 1.74–3.0 (character 342: 4 → 5&6).

Archosauriforms more crownward than proterosuchids (i.e. *Sarmatosuchus otschevi*, NMQR 3570, *Kalisuchus rewanensis*, *Antarctanax shackletoni*, and a clade that includes Erythrosuchidae + Eucrocopoda; figure 39) possess the following synapomorphies in those trees where *Antarctanax shackletoni* is found as the earliest diverging member of the clade: posterior cervical, anterior dorsal, and sometimes anterior–middle cervical and middle–posterior dorsal vertebrae with postzygodiapophyseal lamina (character 318: 0 → 1); and fifth cervical vertebra to middle dorsal vertebrae with gradual transverse expansion of the distal half of the neural spine, but lacking distinct mammillary processes on the lateral surface of the neural spine (character 320: 2 → 1). By contrast, the following synapomorphy is optimized if a clade composed of *Kalisuchus rewanensis* + NMQR 3570 is positioned as the earliest diverging terminals of the group that includes archosauriforms more crownward than proterosuchids: cranium and lower jaw with large interdental plates close to or contacting with each other (character 1: 1 → 2).

The iterPCR protocol recognized that NMQR 3570 and *Kalisuchus rewanensis* were unstable taxa among the most immediate sister taxa to the Erythrosuchidae + Eucrocopoda clade. Their *a posteriori* pruning resulted in the recovery of *Antarctanax shackletoni* and *Sarmatosuchus otschevi* as the successive sister taxa to the Erythrosuchidae + Eucrocopoda clade. *Sarmatosuchus otschevi* is placed as the sister taxon to all other archosauriforms with the exception of proterosuchids and *Antarctanax shackletoni* because of the presence of middle and posterior dorsal vertebrae with diapophysis level with the anteroposterior middle of the centrum (character 660: 0 → 1). In all the MPTs, *Kalisuchus rewanensis* and NMQR 3570 form a clade that could include or not *Antarctanax shackletoni* or *Sarmatosuchus otschevi*. This group is supported by the presence of an antorbital fenestra with a squared or slightly obtuse anteroventral corner in lateral view as a result of a sharp inflexion between the anterior and ventral margins of the opening (character 14: 0 → 2; unknown in *Antarctanax shackletoni* and *Sarmatosuchus otschevi*). Beyond these clades recovered in all the MPTs, the other relationships among these non-proterosuchid early archosauriforms are strongly variable and several of the alternative groupings are not supported by synapomorphies, e.g. the position of *Kalisuchus rewanensis* + NMQR 3570 as more crownward than *Sarmatosuchus otschevi* and *Antarctanax shackletoni*, and the position of *Sarmatosuchus otschevi* and *Antarctanax shackletoni* as the sister taxon to the *Kalisuchus rewanensis* + NMQR 3570 clade.

The bootstrap frequencies of Proterosuchidae are extremely low (absolute = 3–4%; GC = −1−−3%) and they increase slightly after the *a posteriori* pruning of fragmentary terminals (i.e. *Archosaurus rossicus*, UNIPAMPA 271, 684, 750, FC-DPV 2641, *Vonhuenia friedrichi*, *Jaikosuchus magnus*, *Gamosaurus lozovskii*, *Tsylmosuchus* spp., Long Reef proterosuchian, the Arcadian proterosuchian vertebrae, *Kalisuchus rewanensis*, and NMQR 3570; analysis under implied weighting with *k* = 20). The absolute and GC bootstrap frequencies of Proterosuchidae increase to 49% and 40%, respectively (figure 40*c*). The absolute and GC bootstrap frequencies calculated without fragmentary terminals are 66% and 56% for the *Proterosuchus*/‘*Chasmatosaurus*’ clade, 37% and −4% for the clade that includes the three species of *Proterosuchus* from South Africa, 39% and 23% for the *Proterosuchus fergusi* + *Proterosuchus alexanderi* clade, and 67% and 55% for Chasmatosuchinae (i.e. *Chasmatosuchus rossicus + Samsarasuchus pamelae*), respectively (electronic supplementary material, figure S7).

### 7.2. Analysis 2: results using the ‘*Jaikosuchus* Ochev hypodigm’

The analyses with *k* = 19−24 found more than 250 000 MPTs in all cases (*k* = 19: fit of 197.37802, CI of 0.18242 and RI of 0.63883; *k* = 20: fit of 190.21652, CI of 0.18242 and RI of 0.63883; *k* = 21: fit of 183.56899, CI of 0.18262 and RI of 0.63929; *k* = 22: fit of 177.37604, CI of 0.18262 and RI of 0.63929; *k* = 23: fit of 171.59683, CI of 0.18262 and RI of 0.63929; *k* = 24: fit of 166.19044, CI of 0.18262 and RI of 0.63929). The non-eucrocopodan archosauriform region of all the SCTs and subsequent SRCTs are identical to those of Analysis 1 (electronic supplementary material, figures S8–S13). There is no optimized apomorphy related to the fibula referred by Ochev [113] to *Jaikosuchus magnus* that supports the position of the ‘*Jaikosuchus* Ochev hypodigm’ terminal within Proterosuchidae. The bootstrap frequencies are very low throughout the non-erythrosuchid, non-eucrocopod archosauriform part of the tree.

### 7.3. Analysis 3: results using the ‘*Jaikosuchus + Vytshegdosuchus*’ terminal

This analyses with *k* = 19−24 found more than 250 000 MPTs in all cases (*k* = 19: fit of 197.37223, CI of 0.18239 and RI of 0.63869; *k* = 20: fit of 190.21186, CI of 0.18239 and RI of 0.63869; *k* = 21: fit of 183.56635, CI of 0.18239 and RI of 0.63869; *k* = 22: fit of 177.37538, CI of 0.18259 and RI of 0.63915; *k* = 23: fit of 171.59691, CI of 0.18259 and RI of 0.63915; *k* = 24: fit of 166.19118, CI of 0.18259 and RI of 0.63915). The SCTs have the same topology, which are almost identical topologically to those of Analyses 1 and 2. The only difference is the position of the ‘*Jaikosuchus + Vytshegdosuchus*’ terminal as an aphanosaurian avemetatarsalian in all the MPTs (electronic supplementary material, figures S14–S19). Although *Jaikosuchus magnus* was recovered consistently as a chasmatosuchine proterosuchid in Analyses 1–2, the position of the ‘*Jaikosuchus + Vytshegdosuchus*' terminal within Archosauria is not completely unexpected because *Vytshegdosuchus zheshartensis* was found as a member of Aphanosauria in these previous analyses. The ‘*Jaikosuchus + Vytshegdosuchus*’ terminal has the following synapomorphies of Aphanosauria: postaxial cervical vertebrae with shallow excavation immediately lateral to the base of neural spines (character 337: 0 → 1); Cv3–Cv5 with maximum height of neural spine versus height of posterior articular surface of centrum between 1.40 and 1.68 (character 342: 2/3 → 4); anterior and middle postaxial cervical neural spines with an anterior overhang as a result of an anteriorly curved anterior margin of the neural spine (character 343: 0 → 1); and hyposphene-hypantrum present at least in one anterior–middle cervical vertebra (character 896: 2 → 3). The ‘*Jaikosuchus + Vytshegdosuchus*' terminal is found more closely related to other aphanosaurians than to *Spondylosoma absconditum* because of the presence of fourth to eight presacral vertebrae with posterior expansion of the neural spine, resulting in a posterodorsally tilted posterior margin higher than 15° with respect to the anterior margin of the neural spine in lateral view (character 901: 0 → 1). The relationships between the ‘*Jaikosuchus + Vytshegdosuchus*’ terminal and the other more deeply nested aphanosaurians are unresolved, but the iterPCR protocol recognized that *Dongusuchus efremovi* was the topologically unstable terminal. The *a posteriori* pruning of this species resulted in the recovery of *Yarasuchus deccanensis* as the sister taxon to the ‘*Jaikosuchus + Vytshegdosuchus*' terminal and *Teleocrater rhadinus*. This clade is supported by two synapomorphies: third to eighth or ninth presacral vertebrae with diagonal, anterodorsally-to-posteroventrally oriented ridge that reaches the base of the prezygapophysis and it is not connected to the diapophysis on the lateral surface of the neural arch (character 895: 0 → 1) and ilium with medially curved preacetabular process in dorsal view (character 906: 0 → 1). The bootstrap frequencies are very low (less than 50%) among non-erythrosuchid, non-eucrocopod archosauriforms and Aphanosauria.

### 7.4. Analysis 4: results using the ‘*Kalisuchus rewanensis* combined’ terminal

**Figure 41.**
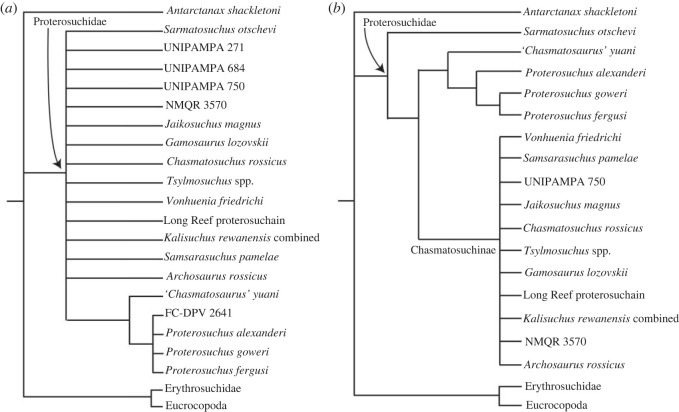
Selected consensus subtrees recovered in Analysis 4 under implied weighting with *k* = 19–24 focused on the non-erythrosuchid, non-eucrocopodan archosauriform region of the trees—Erythrosuchidae and Eucrocopoda have been collapsed for simplicity. (*a*) Strict consensus tree; and (*b*) strict reduced consensus tree after the *a posteriori* pruning of FC-DPV 3570 and UNIPAMPA 271 and 684.

The analyses with *k* = 19−24 found more than 250 000 MPTs in all cases (*k* = 19: fit of 197.36122, CI of 0.18237 and RI of 0.63864; *k* = 20: fit of 190.20265, CI of 0.18237 and RI of 0.63864; *k* = 21: fit of 183.55715, CI of 0.18256 and RI of 0.63911; *k* = 22: fit of 177.36657, CI of 0.18256 and RI of 0.63911; *k* = 23: fit of 171.58949, CI of 0.18256 and RI of 0.63911; *k* = 24: fit of 166.18501, CI of 0.18256 and RI of 0.63911). The SCTs are mostly congruent to those of Analyses 1–2, but the ‘*Kalisuchus rewanensis* combined’ terminal, NMQR 3570, and *Sarmatosuchus otschevi* are recovered as proterosuchids in all the MPTs (figures 41*a* and electronic supplementary material, S20–S25). These three terminals are recovered in a polytomy at the base of Proterosuchidae together with all the other members of the group and a clade composed of ‘*Chasmatosaurus*’ *yuani* + (FC-DPV 2641 + *Proterosuchus alexanderi* + *Proterosuchus fergusi* + *Proterosuchus goweri*). In these analyses, Proterosuchidae has only one synapomorphy: posterior cervical, anterior dorsal, and sometimes middle dorsal vertebrae with a thick, mainly vertical tuberosity immediately ventral to the transverse process (character 319: 0 → 1*). However, the synapomorphies of Proterosuchidae are strongly limited because of the high amount of missing data present in UNIPAMPA 271 (98% of missing data). When this latter specimen is not considered, Proterosuchidae has the following six synapomorphies in addition to character 319 (0 → 1): premaxilla with strongly downturned main body, prenarial process obscured by the postnarial process in lateral view (if the postnarial process is long enough) and postnarial process parallel or posteroventrally oriented with respect to the main axis of the premaxillary body (character 29: 1 → 2); premaxilla with narial fossa expanded in the anteroventral corner of the naris (character 32: 0 → 1); premaxilla with sharp dorsal flange at the base of the postnarial process delimiting the posteroventral border of the external naris (character 38: 0 → 1); maxilla with posterior extension at level or posterior to the posterior orbital border in non-early juvenile individuals in lateral view (character 71: 1 → 0); dentary with large foramina aligned in two distinct rows starting on the anteroventral corner of the bone (character 268: 0 → 1); and frontal transversely broader than the transverse width of the bone posterior to the contribution to the orbital margin (character 717: 0 → 1).

The iterPCR protocol shows that *Sarmatosuchus otschevi* is resolved as the earliest diverging member of Proterosuchidae after the *a posteriori* pruning of UNIPAMPA 271, and its exclusion from the clade that includes more deeply nested proterosuchids is supported by the absence of the following seven synapomorphies: premaxilla with anteroposteriorly deep base of the prenarial process (character 35: 0 → 1); basioccipital– parabasisphenoid with loose, overlapping suture in non-early juvenile individuals (character 225: 1 → 0); parabasisphenoid with posterolaterally oriented basipterygoid processes (character 248: 0 → 1); Cv3–Cv5 with maximum height of neural spine versus height of posterior articular surface of centrum between greater than 1.17 (character 342: 2 → 3–6*); middle caudal vertebrae with accessory laminar process on the anterior face of the neural spine (character 380: 0 → 1); lower jaw with narrow symphyseal space and well organized rugosities (class II of Holliday & Nesbitt [159]) (character 859: 0 → 1); and dentary with anterior end of the bone distinctly transversely broader than at level of or posterior to the sixth tooth position in dorsal or ventral view (character 891: 0 → 1).

The *a posteriori* pruning of UNIPAMPA 271 also results in the recovery of Chasmatosuchinae (figure 41*b* and electronic supplementary material, figure S26), as in previous analyses. In particular, NMQR 3570 and ‘*Kalisuchus rewanensis* combined’ are recovered as members of Chasmatosuchinae. The placement of ‘*Kalisuchus rewanensis* combined’ in Chasmatosuchinae is supported by all apomorphies already scored in the ‘Archadia proterosuchian vertebrae’ terminal and the information provided by the holotype of *Kalisuchus rewanensis* (an isolated maxilla) does not add proterosuchid apomorphies. The position of NMQR 3570 within Chasmatosuchinae is a consequence of the placement of ‘*Kalisuchus rewanensis* combined’ in this clade because both terminals share the following apomorphy: antorbital fenestra with squared or slightly obtuse anteroventral corner as a result of a sharp inflexion between the anterior and ventral margins of the opening in lateral view (character 14: 0 → 2). NMQR 3570 is positioned as the sister taxon to the ‘*Kalisuchus rewanensis* combined’ terminal in some MPTs, but this is not supported by any unambiguous apomorphy. NMQR 3570 acquires multiple alternative positions within Chasmatosuchinae in the other MPTs. The branch supports are extremely low for this part of the tree, as was the case in previous analyses.

### 7.5. Analysis 5: results including ‘*Chasmatosuchus*’ *vjushkovi*

This analyses with *k* = 19–24 found more than 250 000 MPTs in all cases (*k* = 19: fit of 197.31479, CI of 0.18248 and RI of 0.63908; *k* = 20: fit of 190.15540, CI of 0.18248 and RI of 0.63908; *k* = 21: fit of 183.50984, CI of 0.18267 and RI of 0.63955; *k* = 22: fit of 177.31872, CI of 0.18267 and RI of 0.63955; *k* = 23: fit of 171.54123, CI of 0.18267 and RI of 0.63955; *k* = 24: fit of 166.13645, CI of 0.18267 and RI of 0.63955). In all the SCTs, Proterosuchidae is recognized as a clade composed of two groups, one corresponding to the *Proterosuchus*/‘*Chasmatosaurus*’ clade and the other to Chasmatosuchinae (electronic supplementary material, figures S27–S32). The taxonomic content of these clades is the same as in Analyses 1 and 2. ‘*Chasmatosuchus*’ *vjushkovi* is recovered in a polytomy with Proterosuchidae, the Erythrosuchidae + Eucrocopoda clade, and the successive sister taxa to the latter clade found in Analyses 1–3 (e.g. *Sarmatosuchus otschevi*, *Kalisuchus rewanensis*, *Antarctanax shackletoni*). The iterPCR protocol found that the a posteriori pruning of *Antarctanax shackletoni* resolves the position of *Sarmatosuchus otschevi*, *Kalisuchus rewanensis*, and NMQR 3570 as closer to the Erythrosuchidae + Eucrocopoda clade than to Proterosuchidae within Archosauriformes; but the relationship between these terminals is unresolved. In these SRCTs, ‘*Chasmatosuchus*’ *vjushkovi* is positioned in a trichotomy with Proterosuchidae and the clade formed by all other archosauriforms. Among the MPTs, ‘*Chasmatosuchus*’ *vjushkovi* is alternatively recovered as the sister taxon to Archosauriformes, a proterosuchid outside of the *Proterosuchus*/‘*Chasmatosaurus*’ + Chasmatosuchinae clade, or as one of the successive sister taxa to the clade composed of *Kalisuchus rewanensis* + NMQR 3570 + *Sarmatosuchus otschevi* + (Erythrosuchidae + Eucrocopoda). ‘*Chasmatosuchus*’ *vjushkovi* shares with archosauriforms the following apomorphy: premaxilla with strongly downturned main body, prenarial process obscured by the postnarial process in lateral view (if the postnarial process is long enough) and postnarial process parallel or posteroventrally oriented with respect to the main axis of the premaxillary body (character 29: 1 → 2). In the MPTs in which ‘*Chasmatosuchus*’ *vjushkovi* is recovered within Proterosuchidae, the exclusion of this species from the *Proterosuchus*/‘*Chasmatosaurus*’ + Chasmatosuchinae clade is because of the absence of a premaxilla with an anteroposteriorly deep base of the prenarial process (character 35: 0 → 1), and its exclusion from more crownward archosauriforms is because of the absence of a cranium or lower jaw with large interdental plates close to or contacting with each other (character 1: 0/1 → 2). The position of ‘*Chasmatosuchus*’ *vjushkovi* within Proterosuchidae or as one of the earliest branching taxa leading to Eucrocopoda is not supported by apomorphies, but by homoplasy minimization. The branch supports are extremely low for this part of the tree (less than 50%), as was the case in previous analyses.

### 7.6. Analysis 6: results using the less inclusive hypodigms

The analyses with *k* = 19–24 found more than 250 000 MPTs in all cases (*k* = 19: fit of 196.94388, CI of 0.18306 and RI of 0.63877; *k* = 20: fit of 189.78703, CI of 0.18306 and RI of 0.63877; *k* = 21: fit of 183.14451, CI of 0.18314 and RI of 0.63897; *k* = 22: fit of 176.96059, CI of 0.18314 and RI of 0.63897; *k* = 23: fit of 171.19005, CI of 0.18314 and RI of 0.63897; *k* = 24: fit of 165.79197, CI of 0.18314 and RI of 0.63897). The SCTs (electronic supplementary material, figures S33–S38) recovered a Proterosuchidae at the base of Archosauriformes that is composed of the three species of *Proterosuchus* from South Africa, ‘*Chasmatosaurus*’ *yuani*, UNIPAMPA 271 and 684, FC-DPV 2641, and ‘*Archosaurus rossicus* holotype.’ This taxonomic content of Proterosuchidae is restricted to the *Proterosuchus*/‘*Chasmatosaurus*’ clade of Analyses 1–5, with the additional placement of *Archosaurus rossicus* within it rather than in a clade that includes *Chasmatosuchus rossicus*. The position of *Archosaurus rossicus* within the *Proterosuchus*/‘*Chasmatosaurus*’ clade is supported by the following two synapomorphies: premaxilla with an anteroposteriorly deep base of the prenarial process (character 35: 0 → 1); and premaxilla with lateroventrally opened anterior alveoli in mature individuals (character 44: 0 → 1). The iterPCR protocol shows that the a posteriori pruning of *Archosaurus rossicus* resolves a clade composed of ‘*Chasmatosaurus*’ *yuani* and UNIPAMPA 271 and 684, which is supported by the presence of posterior cervical, anterior dorsal, and sometimes middle dorsal vertebrae with a thick, mainly vertical tuberosity immediately ventral to the transverse process (character 319: 0 → 1). The groups formed by the three species of *Proterosuchus* from South Africa and FC-DPV 2641 is supported by the three synapomorphies listed in Analysis 1 for this clade and the presence of parabasisphenoid with a pair of thick parasphenoid crests running along the ventrolateral border of the basisphenoid body and framing the ventromedial floor of the vidian canal (character 246: 0 → 1).

As in Analyses 1–2, the clade immediately crownward to Proterosuchidae is formed by a polytomy composed of *Sarmatosuchus otschevi*, NMQR 3570, ‘*Kalisuchus rewanensis* holotype’, *Antarctanax shackletoni*, and the Erythrosuchidae + Eucrocopoda clade. *Samsarasuchus pamelae*, the Arcadia proterosuchian vertebrae, UNIPAMPA 750, ‘*Vonhuenia friedrichi* holotype’, *Tsylmosuchus* spp., ‘*Chasmatosuchus rossicus* holotype’, *Gamosaurus lozovskii*, *Jaikosuchus magnus*, the Long Reef proterosuchian and *Guchengosuchus shiguaiensis* are included within Chasmatosuchinae and this clade is found within Erythrosuchidae, contrasting with its placement within Proterosuchidae in Analyses 1–5. The placement of the chasmatosuchines within Erythrosuchidae is supported by the presence of a ninth presacral centrum with a ventral keel (character 892: 0 → 1). ‘*Vonhuenia friedrichi* holotype’ is found at the base of Chasmatosuchinae because it lacks the following synapomorphy of more deeply nested members of the clade: posterior cervical, anterior dorsal and sometimes middle dorsal vertebrae with a thick, mainly vertical tuberosity immediately ventral to the transverse process (character 319: 0 → 1). Among these chasmatosuchines, ‘*Chasmatosuchus rossicus* holotype’, the Long Reef proterosuchian, and *Guchengosuchus shiguaiensis* form a clade of unresolved relationships in the SCTs, which is supported by the presence of: postaxial cervical vertebrae with a shallow, posterolaterally facing fossa on the posterior portion of the neural arch ventral to the postzygapophysis (character 335: 1 → 0; ambiguous in those MPTs in which the Long Reef proterosuchian is positioned at the base of the clade); and dorsal vertebrae with spinoprezygapophyseal lamina (character 682: 0 → 1; ambiguous in those MPTs in which ‘*Chasmatosuchus rossicus* holotype’ is positioned at the base of the clade).

All the other chasmatosuchines (*Tsylmosuchus* spp., *Gamosaurus lozovskii*, *Jaikosuchus magnus*, UNIPAMPA 750, the Arcadia proterosuchian vertebrae and *Samsarasuchus pamelae*) are positioned in a clade that has the following synapomorphies: fifth cervical vertebra to middle dorsal vertebrae with distinct mammillary processes on the lateral surface of the neural spine (character 320: 1 → 2*; ambiguous in those MPTs in which *Gamosaurus lozovskii* is positioned at the base of the clade); and fourth to sixth cervical vertebrae with strongly developed longitudinal lamina or tuberosity extended posteriorly from the base of the transverse process, flaring laterally as a prominent and thick, wing-like shelf (character 334: 0 → 1*). If *Gamosaurus lozovskii* and the Arcadia proterosuchian vertebrae are pruned a posteriori, *Samsarasuchus pamelae* is recovered as the sister taxon to a trichotomy composed of *Tsylmosuchus* spp., *Jaikosuchus magnus* and UNIPAMPA 750. This clade has one synapomorphy: first serial occurrence of the posterior centrodiapophyseal lamina in the ninth presacral vertebra or posterior to it (character 893: 1 → 0*). Finally, the *Tsylmosuchus* spp., *Jaikosuchus magnus* and UNIPAMPA 750 clade is supported by the presence of third to fifth cervical vertebrae with maximum height of neural spine versus height of posterior articular surface of centrum of 1.40–3.0 (character 342: 3 → 4&5&6). The bootstrap frequencies are very low (less than 50%) in the non-eucrocopod archosauriform region of the tree.

### 7.7. Analysis 7: results using all Panchet cf. proterosuchid and proterosuchid specimens as a single terminal

The analyses with *k* = 19–24 found more than 250 000 MPTs in all cases (*k* = 19: fit of 197.41629, CI of 0.18237 and RI of 0.63920; *k* = 20: fit of 190.25450, CI of 0.18237 and RI of 0.63920; *k* = 21: fit of 183.60663, CI of 0.18256 and RI of 0.63966; *k* = 22: fit of 177.41330, CI of 0.18256 and RI of 0.63966; *k* = 23: fit of 171.63369, CI of 0.18256 and RI of 0.63966; *k* = 24: fit of 166.22687, CI of 0.18256 and RI of 0.63966). The topologies of the SCTs and the subsequent SRCTs are identical to those of Analysis 1 (electronic supplementary material, figures S38–S44). Proterosuchidae is supported by the same 11 synapomorphies of Analysis 1, but two additional synapomorphies are optimized here because of the extra information included in the ‘*Samsarasuchus pamelae* expanded’ terminal: quadrate with angle between the posterior margins of the dorsal and ventral ends of 143°–158° (character 177: 2 → 3*); and humerus with transverse width of the proximal end versus total length of the bone in non-early juvenile individuals of 0.48–0.70 (character 416: 1 → 2*). Similarly, one additional synapomorphy is optimized for the *Proterosuchus*/‘*Chasmatosaurus*’ clade because of its absence in ‘*Samsarasuchus pamelae* expanded’: humerus with strong medial development of the entepicondyle, being distinctly offset from the shaft and forming an angle less than 45° with respect to the longitudinal axis of the shaft (character 425: 1 → 2). The apomorphies of Chasmatosuchinae and its lesser inclusive clades are the same as in Analysis 1. The bootstrap frequencies are very low (less than 50%) in the non-eucrocopod archosauriform region of the tree. The *a posteriori* pruning of fragmentary terminals (i.e. *Archosaurus rossicus*, UNIPAMPA 271, 684, 750, FC-DPV 2641, *Vonhuenia friedrichi*, *Jaikosuchus magnus*, *Gamosaurus lozovskii*, *Tsylmosuchus* spp., Long Reef proterosuchian, the Arcadian proterosuchian vertebrae, *Kalisuchus rewanensis* and NMQR 3570; analysis under implied weighting with *k* = 20) result in higher frequencies: Proterosuchidae = 68% and 62%, absolute and GC bootstrap frequencies, respectively; the *Proterosuchus*/‘*Chasmatosaurus*’ clade = 72% and 64%; the clade that includes the three species of *Proterosuchus* from South Africa = 41% and −6%; the *Proterosuchus fergusi* + *Proterosuchus alexanderi* clade = 47% and 29%; and Chasmatosuchinae = 77% and 60%.

### 7.8. Leaf stability in the phylogenetic analyses

**Figure 42.**
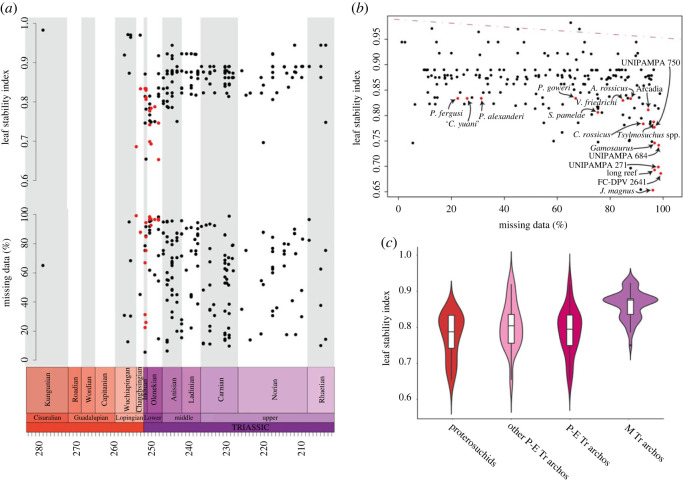
Plots of LSI and missing data. (*a*) LSI (top) and proportion of missing data (bottom) versus geological time; (*b*) LSI versus proportion of missing data; and (*c*) violin plots showing the distribution of the LSI values for different archosauromorph groups. Red dots in (*a*) and (*b*) indicate proterosuchids. The violet dotted line in (*b*) represents the phylogenetic generalized least squares regression line. archos, archosauromorphs; M Tr, Middle Triassic; P-E Tr, Permian–Early Triassic.

The mean of the LSI of all the terminals included in Analysis 5 (to the exclusion of *Petrolacosaurus kansensis*, which was used to root the trees and as a result has an LSI of 1) is 0.86 (standard deviation [s.d.] = 0.06). All proterosuchids have lower values than the mean of the dataset (LSI = 0.65−0.83, mean = 0.77, s.d. = 0.06) and this is clearly visible in the plot of LSI against geological time (figure 42*a*). Nevertheless, Permian–Early Triassic non-proterosuchid archosauromorphs have similarly low values (LSI = 0.65–0.92, mean = 0.80, s.d. = 0.07) and the two groups do not differ significantly in the phylogenetic ANOVA (pANOVA; *p* = 1.00) (figure 42*c*). The plot of LSI against geological time shows that Middle and Late Triassic terminals have generally higher LSI values than older taxa (figure 42*a*). Indeed, the mean of the LSI of Middle Triassic archosauromorphs is 0.86 (s.d. = 0.04) and they differ significantly from the LSI values of Permian–Early Triassic non-proterosuchid archosauromorphs (pANOVA *p*-value = 0.03) (figure 42*c*). Proterosuchids do not differ significantly from Middle Triassic archosauromorphs (pANOVA *p*-value = 0.20). However, when all Early Triassic archosauromorphs are compared with those of the Middle Triassic there is a significant difference between their LSI values (pANOVA *p*-value = 0.01) (figure 42*c*).

The plot of the amount of missing data of each terminal against geological time does not show a clear concentration of fragmentary terminals in any particular portion of the studied temporal range, but an apparently random distribution of the data (figure 42*a*). This contrasts with the concentration of the lowest LSI values around the Permo-Triassic boundary. The phylogenetic generalized least-squares (pGLS) regression of missing data versus the mean age of the terminals is not significant (*p*-value = 0.13; *R*^2^ = 0.01; figure 42*b*), nor is the pGLS regression of LSI against geological time (*p*-value = 0.62; *R*^2^ < 0.01). The pANOVA did not find significant differences between the missing data values of proterosuchids, Permian–Early Triassic non-proterosuchid archosauromorphs and Middle Triassic archosauromorphs; nor between Early Triassic and Middle Triassic archosauromorphs.

The plot of LSI against missing data shows that the lowest LSI values occur only in terminals with a proportion of missing data greater than 80%, but several other highly fragmentary terminals also have relatively high LSI values (greater than 0.80; figure 42*b*). The pGLS regression of LSI versus missing data is significant (*p*-value < 0.01), but this model explains a very low amount of variance (*R*^2^ = 0.10) (figure 42*b*). Indeed, the plot of the residuals of this model against geological time is very similar to that of LSI against geological time. Finally, the pGLS regression of LSI versus missing data + geological time finds a significant interaction only with missing data (*p*-value < 0.01) and not with the age of the terminals (*p*-value = 0.83); the explained variance of this model is very low (*R*^2^ = 0.10).

## 8. Discussion

### 8.1. *Samsarasuchus pamelae* as a new genus and species of proterosuchid

Following the discovery of proterosuchids in Lower Triassic beds in South Africa and China, the previously named proterosuchid genus and species from the Panchet Formation, ‘*Ankistrodon*’ and ‘*Ankistrodon indicus*’, were considered *nomina dubia* [18,35]. Indeed, the proterosuchid remains from the Indian unit have been generally considered as co-generic with those of the above-mentioned countries, being assigned to either *Proterosuchus* or ‘*Chasmatosaurus*’ [37,44–46,73–76]. The new information about early archosauriforms published in recent decades bolsters the non-diagnostic status of the holotype of ‘*Ankistrodon indicus*’. This species has labiolingually compressed tooth crowns with a distally serrated carena and ankylothecodont tooth implantation (see Description). This preserved morphology is indistinguishable from those of ‘*Chasmatosaurus*’ *yuani* and the three species of *Proterosuchus* from South Africa. As a result, ‘*Ankistrodon*’ and ‘*Ankistrodon indicus*’ are not valid, and the diagnostic Panchet proterosuchid specimens described here cannot be assigned to these taxa.

As mentioned in the Diagnosis of the Systematic Palaeontology section, there is a unique combination of character states that allow the postaxial cervical to middle dorsal vertebrae of the Panchet proterosuchids to be distinguished from other nominal early archosauriform species. At the same time, these features provide a morphological link between the holotype, a Cv9, and the other vertebrae here unambiguously referred to *Samsarasuchus pamelae*. Moreover, it is possible to track a gradual serial change in the morphology of the postaxial cervical and dorsal vertebrae that is consistent with the interpretation that they belong to a single proterosuchid species (figure 2).

The new anatomical information provided here for the Panchet proterosuchid *Samsarasuchus pamelae* has shown multiple differences with the South African and Chinese proterosuchids, including: a diagonal ridge that reaches the prezygapophysis on the lateral surface of the neural arch of Cv3–Cv8; a strongly laterally developed longitudinal shelf projecting posteriorly from the base of the diapophysis and a vertical or posterodorsally canted neural spine in Cv4–Cv6; a posteriorly expanded neural spine in Cv4–Cv8; an anteriorly curved anterior margin and distally restricted transverse expansion of the neural spine in anterior–middle cervical vertebrae; a longitudinal keel on the ventral surface of the centrum in Cv9; a prezygodiapophyseal lamina in posterior cervical and anterior dorsal vertebrae; a posterior centrodiapophyseal lamina in anterior dorsal vertebrae; and a longitudinal keel on the ventral surface of the middle dorsal centra. Thus, all these character states demonstrate that the anatomy of *Samsarasuchus pamelae* is considerably different from that of the genera *Proterosuchus* and ‘*Chasmatosaurus*’ than previously assumed. In addition, several of the cf. proterosuchid and proterosuchid specimens from the Panchet Formation that cannot be unambiguously referred to *Samsarasuchus pamelae* also clearly differ from those of *Proterosuchus* spp. and ‘*Chasmatosaurus*’ *yuani* in the presence of: a foramen on the medial wall of the quadrate foramen; postzygapophyses well-developed posteriorly, forming a distinct notch with the posterior margin of the neural spine, and a dorsally convex dorsal margin of the neural spine in the axis; second sacral rib with posterolateral process oriented at an angle of *ca* 125° with respect to the sagittal plane, forming an obtuse angle with the anterolateral portion of the rib in dorsal view; and an entepicondyle of the distal end of the humerus poorly projected from the level of the shaft. Unfortunately, chasmatosuchine species do not preserve these regions of the skeleton and it is not possible to determine if they are features present in members of this clade.

**Figure 43.**
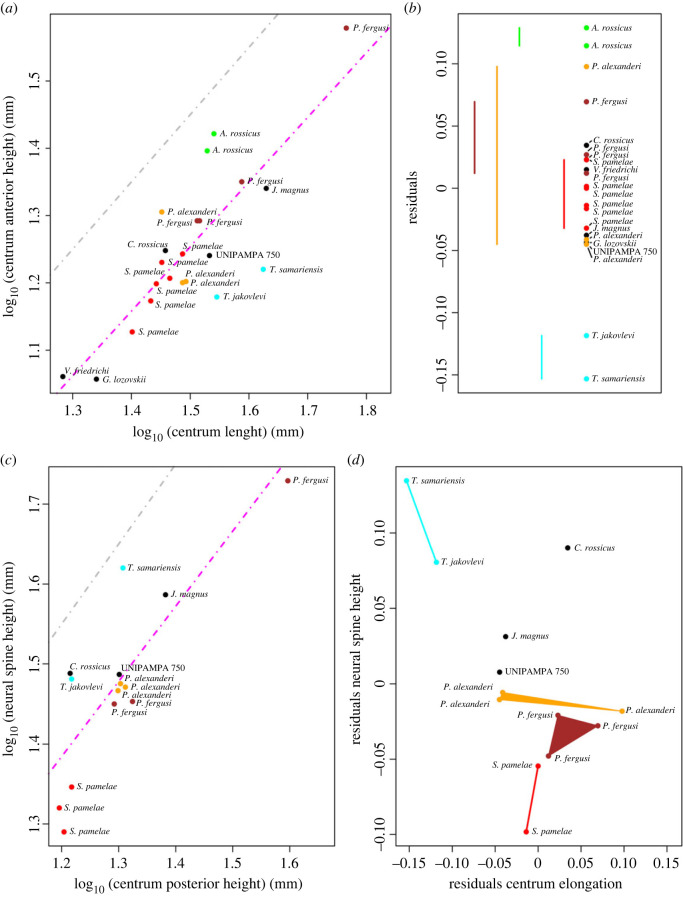
Bivariate plots and generalized least squares (GLS) regressions of proterosuchid Cv3–5 proportions. (*a*) Plot of log_10_(centrum length) versus log_10_(centrum anterior height); (*b*) residuals of the pGLS regression of (*a*), showing species ranges; (*c*) plot of log_10_(centrum posterior height) versus log_10_(neural spine height); and (*d*) plot of the residuals of (*a*) versus (*c*), with convex hulls showing the space occupied by different species. The violet dotted lines correspond to the GLS regressions and the grey dotted lines correspond to a line with a slope = 1.

Ezcurra [18] discussed that the historically collected proterosuchid vertebrae from the Panchet Formation (several of them now forming part of the hypodigm of *Samsarasuchus pamelae*) differed from those of the Russian *Chasmatosuchus rossicus*, *Jaikosuchus magnus*, *Tsylmosuchus samariensis* and *Gamosaurus lozovskii* in the absence of a shelf-like, laterally flaring thick tuberosity projected posteriorly from the base of the diapophysis along the lateral surface of the centrum. This statement was based on a vertebra here interpreted as a Cv3 (GSI 2111) and an anterior dorsal vertebra (GSI 2116), which both belong to vertebral regions that lack such a shelf-like tuberosity in *Samsarasuchus pamelae*. By contrast, the specimens studied here allowed us to recognize the presence of this character state in *Samsarasuchus pamelae*, which is also present in the four species mentioned above, as well as in the Russian *Tsylmosuchus jakovlevi* and the Arcadia proterosuchian vertebrae. When *Samsarasuchus pamelae* is compared to these members of Chasmatosuchinae there are multiple differences with known species. For example, the anterior–middle cervical vertebrae of *Samsarasuchus pamelae* are proportionally shorter (centrum length versus centrum anterior height less than 2.0) than those of *Tsylmosuchus* spp. and the neural spines are proportionally shorter (neural spine height versus posterior articular surface of centrum height less than 1.4) than those of *Tsylmosuchus* spp., *Chasmatosuchus rossicus*, *Jaikosuchus magnus* and UNIPAMPA 750 (figure 43). In addition, an anterior cervical vertebra that has been referred to ‘*Blomosuchus georgii*’ and which is here considered probably referable to *Vonhuenia friedrichi* (PIN 1025/420) lacks the strongly laterally developed, wing-like tuberosity on the lateral surface of the centrum present in *Samsarasuchus pamelae* and other chasmatosuchines. Beyond these differences, the holotype of *Samsarasuchus pamelae* differs from other Permo-Triassic archosauromorphs in the presence of a unique combination of character states (see Diagnosis in Systematic Palaeontology), including an autapomorphic posteriormost cervical vertebra (Cv9) with two pairs of mammillary processes on the neural spine and a dorsolaterally oriented anterior pair of mammillary processes. This evidence supports the proposal that the proterosuchid from the Panchet Formation represents a distinct species.

The results of the phylogenetic analyses further support the distinction between *Samsarasuchus pamelae* and other proterosuchids (figure 40*b*). The Indian taxon is recovered outside of the genus *Proterosuchus* and outside the more deeply nested chasmatosuchines. This phylogenetic arrangement bolsters the erection of a new genus for the Panchet proterosuchid.

### 8.2. The taxonomic content of Chasmatosuchinae

Most of our phylogenetic analyses found a subclade within Proterosuchidae that is composed of seven genera (*Vonhuenia*, *Jaikosuchus*, *Gamosaurus*, *Chasmatosuchus*, *Tsylmosuchus*, *Samsarasuchus* and *Archosaurus*) and several taxonomically unnamed terminals (UNIPAMPA 684 and 750, the Long Reef proterosuchian, and the Arcadia proterosuchian vertebrae) in all the MPTs (figures 39 and 40*a* and table 16). Sennikov [40] erected the subfamily Archosaurinae to include *Archosaurus rossicus* within Proterosuchidae and this name could be used for the subclade found in our analyses. However, we prefer to erect the new clade Chasmatosuchinae because *Archosaurus rossicus* (based only on its holotype) is recovered more closely related to the genera *Proterosuchus* and ‘*Chasmatosaurus*’ than to *Chasmatosuchus* and *Samsarasuchus* in Analysis 6, which uses the less inclusive hypodigms of the non-erythrosuchid, non-eucrocopod archosauriform terminals. If we use the name Archosaurinae for the new subclade of Proterosuchidae, it should include *Archosaurus rossicus* as an internal specifier and would increase the probability that its minimum taxonomic content (i.e. *Archosaurus + Chasmatosuchus*, and maybe also *Samsarasuchus*) becomes unstable. For example, if we follow the results of Analysis 6, Archosaurinae would have the same taxonomic content as Proterosuchidae or the genus *Proterosuchus* depending on its phylogenetic definition. Moreover, Analysis 6 found the clade that includes *Chasmatosuchus rossicus* and most of the genera listed above within Erythrosuchidae. As a consequence, the phylogenetic definition of Chasmatosuchinae not only includes ‘*Chasmatosaurus*’ *yuani* and the three species of proterosuchids from South Africa as external specifiers, but also the erythrosuchid *Erythrosuchus africanus* and the archosaur *Alligator mississippiensis* (see Systematic Palaeontology). As a result, the erection of Chasmatosuchinae and the phylogenetic definition proposed here seeks a long-term stability in its taxonomic content as a clade that includes species closer to *Chasmatosuchus* than to *Proterosuchus*.

Among the chasmatosuchine species recovered here, *Vonhuenia friedrichi* lacks a number of apomorphies present in all other members of the clade and as a result it is the most morphologically dissimilar species of the group. However, suboptimal positions that place *Vonhuenia friedrichi* within the *Proterosuchus*/‘*Chasmatosaurus*’ clade are not supported by apomorphies. Thus, the placement of *Vonhuenia friedrichi* within Chasmatosuchinae seems to be rather robust with the currently available evidence for the species.

The latest Permian and unambiguously oldest known proterosuchid *Archosaurus rossicus* is recovered in the phylogenetic analyses as more deeply nested within Chasmatosuchinae than *Vonhuenia friedrichi*. Under suboptimal topologies, the placement of *Archosaurus rossicus* in the *Proterosuchus*/ ‘*Chasmatosaurus*’ clade is not supported by apomorphies. Similarly, a suboptimal position of *Gamosaurus lozovskii* within the *Proterosuchus*/‘*Chasmatosaurus*’ clade is not supported by apomorphies.

The holotype of *Jaikosuchus magnus* has an overall morphology that resembles the anterior–middle cervical vertebrae of the aphanosaurians *Teleocrater rhadinus* and *Yarasuchus deccanensis*. In particular, *Jaikosuchus magnus* resembles crown archosaurs in the presence of large epipophyses that are adjacent to the postzygapophyseal facet and have a posterior free end. It is noteworthy that an isolated ilium that represents the holotype of *Vytshegdosuchus zheshartensis* was collected in the same Russian Gorizont as *Jaikosuchus magnus*. *Vytshegdosuchus zheshartensis* was consistently recovered in our phylogenetic analyses as an aphanosaurian avemetatarsalian (see below for further implications of this result) and the resemblances between *Jaikosuchus magnus* and aphanosaurs may suggest that both belong to the same or closely related species. Analysis 3 explored the phylogenetic implications of this taxonomic hypothesis and found the ‘*Jaikosuchus* + *Vytshegdosuchus*' terminal within Aphanosauria. As a consequence, although *Jaikosuchus magnus* is possibly a chasmatosuchine proterosuchid, their potential archosaurian affinities and the relationship between *Jaikosuchus* and *Vytshegdosuchus* are worthy of further analyses, especially if more complete specimens become available in the future.

### 8.3. The species-level taxonomy of chasmatosuchine proterosuchids

The holotypes of several of the species here classified as chasmatosuchine proterosuchids are based on one or two isolated cervical vertebrae (*Tsylmosuchus jakovlevi*, *Tsylmosuchus samariensis*, *Gamosaurus lozovskii*, *Chasmatosuchus rossicus*, *Vonhuenia friedrichi*). Poor knowledge about serial variation of the cervical vertebrae in non-erythrosuchid, non-eucrocopod archosauriforms has resulted in different interpretations of the α taxonomy of these species. For example, Ezcurra [18] concluded that *Gamosaurus lozovskii* was a junior synonym of *Jaikosuchus magnus*, and *Tsylmosuchus samariensis* was a junior synonym of *Chasmatosuchus rossicus*. Here, we revisit the taxonomy of these species on the light of the new evidence provided by the cervical series of *Samsarasuchus pamelae*.

First, we plotted the log_10_(centrum length) versus log_10_(centrum anterior height) of Cv3–5 of several proterosuchid specimens and its linear regression (figure 43*a*). The regression had a slope of 0.9607 (*R*^2^ = 0.6581, *p*-value < 0.001), indicating an overall isometric relationship between centrum length and anterior height independent of body size in proterosuchids. Specimens of *Samsarasuchus pamelae* are positioned close to the regression line, with the range of their residuals being close to 0 (figure 43*b*). Similarly, vertebrae of *Proterosuchus alexanderi* also have a good fit to the regression line, with the range of residuals including 0. It is striking that the range of residuals of *Proterosuchus alexanderi* is considerably greater than that of *Samsarasuchus pamelae* despite consisting of measurements from a single, articulated cervical series (NMQR 1484). Specimens of *Proterosuchus fergusi* are always above the regression line and, thus, their residuals are all positive, although not higher than the most extreme value of *Proterosuchus alexanderi*. The two anterior–middle cervical vertebrae referred to *Archosaurus rossicus* depart distinctly from other proterosuchid specimens as a result of possessing proportionally shorter vertebral centra than predicted by the regression (figure 43*a*,*b*). Thus, the range of their residuals has higher values than in other proterosuchid specimens. Conversely, specimens of *Tsylmosuchus* present a similar, but opposite pattern, because their anterior–middle cervical centra are proportionally longer than in other proterosuchids. The range of their residuals is negative and departs considerably from that of other proteorsuchids (figure 43*a*,*b*). The residual for the cervical vertebra referred to *Vonhuenia friedrichi* falls within the range of the residuals of *Samsarasuchus pamelae*. By contrast, a vertebra referred to *Chasmatosuchus rossicus* has a higher residual than the range of the Indian species and the values of the holotypes of *Jaikosuchus magnus* and *Gamosaurus lozovskii*, and UNIPAMPA 750 have lower residuals, although all of them within the range of the residuals of *Proterosuchus alexanderi*.

Second, we plotted the log_10_(centrum posterior height) versus log_10_(neural spine height) of Cv3–5 of several proterosuchid specimens and its linear regression (figure 43*c*). The regression has a slope of 0.9393 (*R*^2^ = 0.6642, *p*-value < 0.001), indicating a non-allometric overall growth of the neural spine height with respect to the centrum posterior height. The cervical vertebrae of *Proterosuchus alexanderi* and UNIPAMPA 750 are those that are better adjusted to the regression line (figure 43*d*). The cervical vertebrae of *Proterosuchus fergusi* have slightly negative residual values, whereas that of the holotype of *Jaikosuchus magnus* has a low positive residual value. The cervical vertebrae of *Samsarasuchus pamelae* have more negative residual values than those of other proterosuchids, demonstrating the presence of proportionally shorter neural spines (see Diagnosis in Systematic Palaeontology). On the other hand, the holotypes of *Tsylmosuchus samariensis* and *Tsylmosuchus jakovlei* and a specimen referred to *Chasmatosuchus rossicus* depart distinctly towards more extreme positive values as a result of the presence of proportionally very tall neural spines (figure 43*c*).

When the information from both linear regressions is combined in a biplot of their residuals, there are interesting patterns that bolster taxonomic decisions (figure 43*d*). Although the neural spine of the anterior–middle cervical vertebra referred to *Chasmatosuchus rossicus* possesses a similar proportional height to those of *Tsylmosuchus samariensis* and *T. jakoveli*, *Tsylmosuchus* species clearly differ in the presence of proportionally longer centra. Based on the lack of strong evidence and in agreement with this result, we do not follow here the hypothesis of synonymy between *Chasmatosuchus rossicus* and *Tsylmosuchus samariensis* (contra Ezcurra [18]). Similarly, we also do not follow the proposed synonymy between *Jaikosuchus magnus* and *Gamosaurus lozovskii* because both species differ in the presence of a thicker diagonal ridge on the lateral surface of the neural arch and the lack of epipophyses in the anterior–middle cervical vertebrae of the latter (contra Ezcurra [18]). Indeed, the thick diagonal ridge of *Gamosaurus lozovskii* differs from the very thin ridge present in other chasmatosuchines and can be interpreted as an autapomorphy of the species. Similarly, if *Jaikosuchus magnus* is considered a proterosuchid, the presence of epipophyses adjacent to the postzygapophyses and with a free posterior end would represent an autapomorphy. The results of the morphometric analysis also show that *Samsarasuchus pamelae* differs from all other proterosuchids in the presence of proportionally shorter neural spines, from *Tsylmosuchus* spp. in the presence of proportionally shorter centra, and from *Archosaurus rossicus* in the presence of proportionally longer centra. This is in agreement with the results of the phylogenetic analyses.

In conclusion, here we follow previous taxonomic proposals that considered *Jaikosuchus magnus*, *Chasmatosuchus rossicus*, *Gamosaurus lozovskii* and *Vonhuenia friedrichi* as valid species and the genus *Tsylmosuchus* as differentiable from the above-mentioned taxa (e.g. [34,40]). Regarding the Arcadia proterosuchian vertebrae, we have found enough evidence to support species-level differentiation. In particular, it is possible that the Arcadia proterosuchian vertebrae could be referrable to *Kalisuchus rewanensis*, whose holotype is based on an isolated maxilla ([111]; see below), but current evidence indicates that it is slightly more parsimonious that they belong to different non-eucrocopod lineages.

### 8.4. Implications for the taxonomic content of Proterosuchidae

Ezcurra [18] concluded that the taxonomic content of Proterosuchidae was considerably more limited than previously thought based on the results of the first comprehensive phylogenetic analysis of non-archosaurian archosauromorphs. This revised taxonomic content of Proterosuchidae was unambiguously restricted to five nominal species: *Archosaurus rossicus*, ‘*Chasmatosaurus*’ *yuani*, *Proterosuchus fergusi*, *Proterosuchus alexanderi* and *Proterosuchus goweri*. Other putative proterosuchid species were reinterpreted as tanystropheids (e.g. ‘*Exilisuchus tubercularis*'; [18]), non-archosauriform crocopods (e.g. *Tasmaniosaurus triassicus*; [18,158]), non-proterosuchid early diverging archosauriforms (e.g. *Sarmatosuchus otschevi*, *Vonhuenia friedrichi*, *Kalisuchus rewanensis*, *Chasmatosuchus rossicus*; [18]), or even pseudosuchian archosaurs (e.g. ‘*Chasmatosaurus ultimus*’, *Koilamasuchus gonzalezdiazi*; [18,42,169]). Three extremely fragmentary taxa, ‘*Chasmatosuchus*’ *vjushkovi*, ‘*Blomosuchus georgii*’ and ‘*Ankistrodon indicus*’, were ambiguously recovered as proterosuchids [18]. The new information provided by *Samsarasuchus pamelae* has allowed the first comprehensive insights into the vertebral anatomy of a non-*Proterosuchus* proterosuchid and acts as a link between the anatomy of *Proterosuchus* and that of other early diverging archosauriforms that are limited to or mostly known from isolated or very limited vertebral remains. The inclusion of *Samsarasuchus pamelae* in the phylogenetic analysis changed the optimization of several characters for this part of the tree.

Our phylogenetic analyses recovered at least 11–13 unambiguous nominal species of Proterosuchidae (the variation in the number of species is because the three species of *Tsylmosuchus* were considered as a genus-level terminal in our analysis), thus representing more than double the previous assessment of the taxonomic content of the clade. ‘*Chasmatosuchus*’ *vjushkovi* is ambiguously found as a proterosuchid, being alternatively recovered in some trees as a non-proterosuchid archosauriform outside of the Erythrosuchidae + Eucrocopoda clade or as the sister taxon to Archosauriformes. In addition, at least six terminals that are indeterminate at the species-level have been also found as members of Proterosuchidae (UNIPAMPA 271, 684, 750, FC-DPV 2641, the Long Reef proterosuchian and the Arcadia proterosuchian vertebrae). In terms of taxonomic content, Proterosuchidae is now richer than Erythrosuchidae, the other main clade of non-eucrocopod archosauriforms. Indeed, the revised taxonomic richness of Proterosuchidae resembles that of other major Triassic archosauromorph clades, such as Tanystropheidae, Allokotosauria and Proterochampsia.

**Figure 44.**
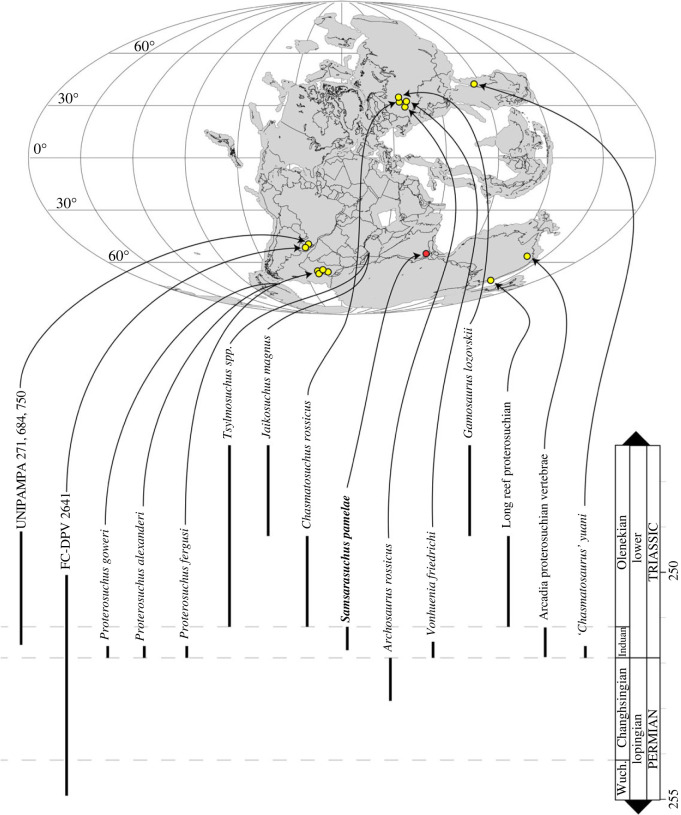
Spatial and temporal distribution of the unambiguous proterosuchid species recovered in phylogenetic Analysis 1. The occurrence of the new genus and species *Samsarasuchus pamelae* is highlighted with a red dot. Palaeomap for 250 Ma downloaded from Fossilworks using data from the Paleobiology Database. Wuch., Wuchiapingian.

The previously more limited taxonomic content of Proterosuchidae was interpreted as indicating a short-lived ‘disaster’ clade, restricted to a few species that occur shortly before and after the end-Permian mass extinction [18]. In our revised phylogeny, this short-lived ‘disaster’ clade is restricted to the genera *Proterosuchus* and ‘*Chasmatosaurus*’, and unambiguously to the Induan of South Africa and China (figure 44). By contrast, the Russian stratigraphic sequence that yielded several chasmatosuchine species indicates that Proterosuchidae had a broader temporal range, with the latest Permian *Archosaurus rossicus*, the Induan *Vonhuenia friedrichi*, the early Olenekian *Chasmatosuchus rossicus*, *Tsylmosuchus jakovlevi* and *Tsylmosuchus samariensis*, and the late Olenekian *Gamosaurus lozovskii*, *Jaikosuchus magnus* and *Tsylmosuchus donensis* (figure 44). These results have noteworthy implications in the context of the early evolutionary radiation of archosauromorphs after the end-Permian mass extinction (see below).

The analysis of the LSIs shows that several species that are here recovered as members of Proterosuchidae are relatively unstable in the phylogenetic trees (figure 42*a*,*b*). In particular, *Chasmatosuchus rossicus*, *Tsylmosuchus* spp., *Gamosaurus lozovskii* and *Jaikosuchus magnus* have lower values than *Proterosuchus* spp., ‘*Chasmatosaurus*’ *yuani* and *Samsarasuchus pamelae*. The analyses recovered a significant correlation between LSI and the amount of missing data. However, this model explains a low amount of the variation of the data and it is probable that the presence of character states that are homoplastic with regard to other clades, mainly in the vertebral column, contributes to the phylogenetic instability of these species. Recovery of more complete material of these more poorly known proterosuchid species may help increasing the topological robusticity of this part of the early archosauriform tree and contribute to a higher stability of the taxonomic content of Proterosuchidae.

### 8.5. The possible proterosuchid affinities of *Kalisuchus rewanensis*

Thulborn [111] described the early archosauriform *Kalisuchus rewanensis* from Lower Triassic rocks of northeastern Australia as a new genus and species of proterosuchid. The holotype is an isolated partial maxilla and Thulborn [111] referred several cervical, dorsal and caudal vertebrae to the species, together with some cranial, girdle and appendicular bones. These vertebrae and others from the same unit were scored here as the ‘Arcadia proterosuchian vertebrae’ because there is no direct evidence to refer them to *Kalisuchus rewanensis* and they show close similarities with those of *Samsarasuchus pamelae*. This scoring strategy also allowed testing the hypothesis that these vertebrae do not belong to *Kalisuchus rewanensis*. The resultant MPTs favour the hypothesis that they may belong to a different taxon because the Arcadia proterosuchian vertebrae are recovered within Chasmatosuchinae and the holotype of *Kalisuchus rewanensis* as more closely related to erythrosuchids + eucrocopods than to proterosuchids (figure 39). However, the referral of these vertebrae to *Kalisuchus rewanensis* is not still unlikely. Under this latter scenario (Analysis 4, in which the holotype of *Kalisuchus rewanensis* and the Arcadia proterosuchian vertebrae are merged into a single ‘*Kalisuchus rewanensis* combined’ terminal), ‘*Kalisuchus rewanensis* combined’ is recovered as a chasmatosuchine proterosuchid. This result implies a higher cranial morphological diversity for proterosuchids because *Kalisuchus rewanensis* possesses well-developed mesial serrations in the marginal dentition, interdental plates that contact each other at their bases, and a vertical ascending process of the maxilla, which are all character states absent in the genera *Proterosuchus* and ‘*Chasmatosaurus*’ [18]. The vertical ascending process of the maxilla of *Kalisuchus rewanensis* is also present in the isolated partial maxilla NMQR 3570 from the Olenekian of South Africa and, as a result, this specimen is also recovered as a chasmatosuchine proterosuchid under this scoring strategy. If NMQR 3570 belongs to Chasmatosuchinae, it would represent the first occurrence of Chasmatosuchinae in the Karoo Basin.

### 8.6. Humeral diversity in proterosuchids and the humerus of *Tasmaniosaurus triassicus*

The well-preserved humeri of the Panchet Formation referred to Proterosuchidae shed light on forelimb morphological diversity among early archosauriforms and their closest relatives. The Panchet proterosuchid humeri and those of other early archosauriforms distinctly differ from those of ‘*Chasmatosaurus*’ *yuani* and *Proterosuchus alexanderi*, which have an entepicondyle distinctly more transversely expanded than the ectepicondyle. The new information made available by the Panchet proterosuchid humeri allowed the recognition of the strongly asymmetric distal end of humerus as a synapomorphy of the *Proterosuchus*/‘*Chasmatosaurus*’ clade.

The Panchet proterosuchid humerus closely resembles a bone of the holotype of *Tasmaniosaurus triassicus* that was interpreted as a tibia by Ezcurra ([158]: figure 14*b*), when that element is rotated through 180° from the original interpretation. The supposed proximal end (as interpreted by Ezcurra [158]) of that element in *Tasmaniosaurus triassicus* is considerably more expanded than the tibiae of other early archosauromorphs (e.g. *Prolacerta broomi*: BP/1/2675; ‘*Chasmatosaurus*’ *yuani*: IVPP V2719; *Cuyosuchus huenei*: MCNAM PV 2669; *Garjainia prima*: [140]). As a result, here we interpret this bone as a left humerus and thus as the first forelimb element identified for *Tasmaniosaurus triassicus*. The main axis of the proximal end of this bone is rotated approximately 90° with respect to the main axis of the distal end, resembling the condition in *Prolacerta broomi* (BP/1/2675), but the angle of curvature is slightly lower in the Panchet proterosuchid humerus and distinctly lower in *Cuyosuchus huenei* (MCNAM PV 2669) and erythrosuchids (e.g. *Garjainia prima*: [140]; *Erythrosuchus africanus*: [141]) (figure 36). The shaft of the humerus of *Tasmaniosaurus triassicus* narrows up to its minimum transverse width close to the mid-length of the bone. In the distal end, the ectepicondyle is damaged, but is distinctly expanded laterally with respect to the shaft. The entepicondyle is moderately medially expanded, resembling the condition in the Panchet proterosuchid humerus, *Cuyosuchus huenei* (MCNAM PV 2669) and *Garjainia prima* [140], but contrasting with the more strongly medially projected entepicondyle apomorphic of the *Proterosuchus*/‘*Chasmatosaurus*’ clade. The entepicondyle is slightly more distally extended than the ectepicondyle. The combination of character states present in the humerus of *Tasmaniosaurus triassicus* is congruent with what would be expected based on its phylogenetic position as the sister taxon to Archosauriformes.

### 8.7. The timing of the origin of Avemetatarsalia

The identification of the oldest known archosaurs is particularly relevant because they establish the minimum age of the split between two of the groups of extant diapsids: Crocodylia and Aves. The currently oldest known archosaurs are pseudosuchians, namely the ctenosauriscid poposauroid *Ctenosauriscus koeneni* from the late Olenekian of Germany [170], the putative paracrocodylomorph *Vytshegdosuchus zheshartensis*, the ctenosauriscid poposauroid *Bystrowisuchus flerovi*, and the ‘rauisuchian’ *Scolotosuchus basileus* from the late Olenekian of Russia [9,28,171,172], and the ctenosauriscid poposauroid *Xilousuchus sapingensis* of a more uncertain late Olenekian–early Anisian age from China [143,170]. On the other hand, although putative but debated avemetatarsalian ichnofossils have been reported from the early Olenekian to early Anisian of Poland [173,174], no avemetatarsalian body fossil has been reported so far from the Early Triassic. The oldest known avemetatarsalian body fossils have been found in poorly temporally constrained Anisian–early Ladinian rocks of Russia, India and continental Africa (e.g. *Dongusuchus efremovi*, *Lutungutali sitwensis*, *Yarasuchus deccanensis*; [104,175,176]).

All our phylogenetic analyses recovered *Vytshegdosuchus zheshartensis* as an aphanosaurian avemetatarsalian instead of a pseudosuchian, representing the first Early Triassic, and thus the oldest, body fossil for the bird-line lineage of Archosauria. The presence of an avemetatarsalian in the late Olenekian is not unexpected because of the occurrence of pseudosuchians of this age, but the position of *Vytshegdosuchus zheshartensis* as an aphanosaur implies that the ghost lineage of Ornithodira extends back into the Early Triassic. This is in agreement with putative ornithodiran footprints reported from the Olenekian of Poland [173,174]. The holotype of *Vytshegdosuchus zheshartensis* is represented by an isolated ilium, but closely resembles that of the aphanosaurian *Teleocrater rhadinus*. In addition, an axis referred to *Vytshegdosuchus zheshartensis* [40] also resembles those of aphanosaurians. The isolated anterior–middle cervical vertebra that is the holotype of *Jaikosuchus magnus* comes from the same gorizont as *Vytshegdosuchus zheshartensis* and also resembles archosaurs, and particularly aphanosaurs, in some features (see Results). Finally, the fibula originally referred to *Jaikosuchus magnus* by Ochev [113] also resembles early avemetatarsalians in the presence of a sharp longitudinal ridge on the anterior surface of the shaft. As a result, although the revised phylogenetic position recovered here for *Vytshegdosuchus zheshartensis* is preliminary and should be treated with caution because it was not the focus of this work, it has important implications for the origin of Archosauria. Thus, a revision of the anatomy of *Vytshegdosuchus zheshartensis* and other potential archosaur remains from the upper Olenekian Yarengian Gorizont of Russia warrant future research effort.

### 8.8. Implications for the evolutionary history of Archosauromorpha around the end-Permian mass extinction

Current knowledge of the impact of the end-Permian mass extinction event upon terrestrial organisms is mostly restricted to South African, Russian and Chinese fossil assemblages and a truly global understanding remains incomplete. The Lower Triassic Panchet Formation of eastern India has historically yielded substantial fossil vertebrate remains in an area that occupied a palaeogeographic position close to 55° S, far from the well-sampled northern outcrops of China and Russia, and further north than localities in South Africa (R package paleoMap; [177]). Moreover, the proximity of the Panchet area to the Tethys Ocean may have resulted in a distinct climate from that of the more inland South African beds (e.g. [178]). Thus, the new information about the archosauriform assemblage of the Panchet Formation enriches our knowledge about the aftermath of the mass extinction event.

Previous studies focused on body size evolution of Archosauromorpha have not found significant differences between the medians of the Permian and earliest Triassic assemblages [179]. In particular, some Permian proterosuchid specimens from Russia, including the holotype and vertebrae referred to *Archosaurus rossicus*, belong to an animal of similar size to those of the largest known specimens of *Proterosuchus fergusi* from the Induan of South Africa and ‘*Chasmatosaurus*’ *yuani* from the Induan of China (CGS GHG 231, IVPP V4067; [31,180]). In the Panchet Formation, a partial anterior dorsal vertebra of *Samsarasuchus pamelae* that lacks most of its neural arch (ISIR 1099) is considerably larger than the other vertebrae referred to the species (centrum height greater than 35 mm). The individual to which ISIR 1099 belonged likely matched the size of the largest proterosuchid specimens of the *Lystrosaurus declivis* Assemblage Zone (AZ) of South Africa and China (e.g. CGS GHG 231, IVPP V4067). Thus, this is further evidence for the presence of relatively large archosauriform specimens in the aftermath of the end-Permian mass extinction event.

Proterosuchids are restricted to a short stratigraphic section of 5–14 m above the Permo-Triassic boundary and they disappear during the first recovery phase of the extinction event in the *Lystrosaurus declivis* AZ of South Africa (probably less than three million years; [53]). The ‘*Chasmatosaurus*’-bearing levels of China are not as well stratigraphically constrained as those of South Africa, and it is possible that the biostratigraphic ranges of ‘*Chasmatosaurus*’ in these horizons are also limited to the first few metres above the Permo-Triassic boundary. Thus, the biostratigraphic range of *Proterosuchus* and ‘*Chasmatosaurus*’ in South Africa and China closely resembles, but is even more chronostratigraphically restricted than, that of the dicynodont genus *Lystrosaurus* [53,97]. In these *Proterosuchus*/‘*Chasmatosaurus*’-bearing beds, the most numerically abundant taxon is the genus *Lystrosaurus*, whereas diapsids represent a minor component of the assemblage. Strikingly, proterosuchids and dicynodonts made up similar proportions of all the specimens collected in the sandstones close to the Deoli locality of the upper Panchet Formation during our fieldwork of 2015 (*ca* 30% for each group). By contrast, the shales of the lower Panchet Formation have yielded several specimens of *Lystrosaurus* but no archosauriform so far [77,78,89]. Thus, it is possible that the relatively high abundance of archosauriforms in the upper Panchet Formation is because they represent younger rocks than those of the *Proterosuchus*/‘*Chasmatosaurus*’-bearing beds of South Africa and China. Those latter beds are probably closer to the time of deposition of the shales of the lower Panchet Formation. If this interpretation is followed, the high abundance of archosauriforms in the upper Panchet Formation would represent a later phase of the aftermath of the end-Permian mass extinction, in which proterosuchids are not only the numerically dominant diapsids, but also one of the most abundant groups of tetrapods together with dicynodonts. However, a temporally driven difference seems to be contradicted by the fact that the upper half of the *Lystrosaurus declivis* AZ in the Karoo Basin (upper half of the Katberg Formation) is currently devoid of archosauriform remains and the parareptile *Procolophon trigoniceps* is a conspicuous member of the assemblage [97,181]; parareptiles are currently unknown in the Panchet Formation (see ‘Palaeontological and geological context of the Panchet proterosuchid specimens'). Another interpretation could be that the palaeogeographic position of the Panchet Formation and its proximity to the Tethys Ocean favoured the abundance of archosauriforms in this part of Pangaea, contrasting with the more inland position of the Karoo Basin. The fossil record of the middle Induan–lower Olenekian Sanga do Cabral Formation of Brazil may favour the presence of regional differences because *Procolophon trigoniceps* is abundant and there are some proterosuchid specimens, but synapsids, including dicynodonts, are extremely rare or absent, contrasting with coeval beds of South Africa [182,183]. Also, a combination of both temporal and geographical factors cannot be ruled out without more detailed biostratigraphic and palaeobiogeographic studies. An improved understanding of the timing of deposition of the Panchet Formation and its tetrapod content throughout the sequence would help to clarify these differences between the earliest Triassic archosauriform assemblages. Thus, further research on the fossil vertebrates of the Panchet Formation is needed, as well as more sampling of other vertebrate-bearing continental units above and below the Permo-Triassic boundary.

## 9. Conclusion

**Figure 45.**
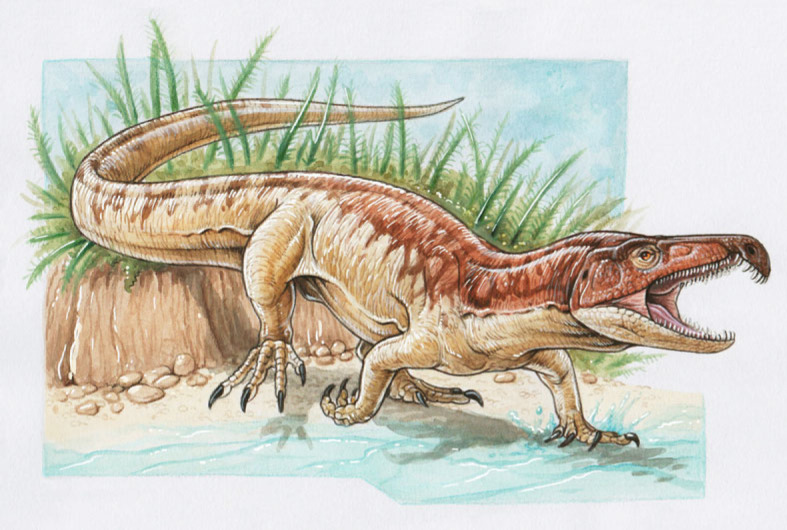
Life reconstruction of *Samsarasuchus pamelae* gen. et sp. nov. Artwork by Gabriel Lio.

Here, we describe the new proterosuchid genus and species *Samsarasuchus pamelae* (figure 45) based on the re-study of historically collected specimens and new discoveries made in recent fieldwork. The hypodigm of this new taxon includes an almost complete presacral vertebral series (figure 2). As a result, *Samsarasuchus pamelae* can be compared with species of the genera *Proterosuchus* and ‘*Chasmatosaurus*’—which include some partial skeletons (e.g. holotype of *Proterosuchus alexanderi* and a few referred specimens of *Proterosuchus fergusi* and ‘*Chasmatosaurus*’ *yuani*; [36,47,49,184])—and also several species and unnamed forms from the Early Triassic of Russia, Brazil and Australia that are mostly known from isolated presacral vertebrae (e.g. *Chasmatosuchus rossicus*, *Vonhuenia friedrichi*, *Gamosaurus lozovskii*, Sanga do Cabral and Arcadia formations specimens) [34,40,110–112,185]. The integration of the new information in the most comprehensive phylogenetic dataset focused on early archosauromorphs recovered a taxonomically, geographically and temporally broader Proterosuchidae than in other recent quantitative analyses (figure 44). In addition, we recovered *Samsarasuchus pamelae* as a member of a novel clade within Proterosuchidae, here named as the new subfamily Chasmatosuchinae. The latter clade also includes forms from Russia, Brazil and Australia. Some cranial bones, axes, posterior dorsal, sacral and caudal vertebrae, and appendicular elements from the Panchet Formation are assigned to cf. proterosuchids or proterosuchids because they do not preserve the unique combination of character states diagnostic of *Samsarasuchus pamelae* and, as a result, cannot be referred unambiguously to this species. In particular, the new anatomical information provided by *Samsarasuchus pamelae* and the cf. proterosuchid and proterosuchid specimens show a higher morphologically diversity among proterosuchids than previously thought, contrasting with previous ideas that the proterosuchid from the Panchet Formation was very similar to species of the genera *Proterosuchus* and *‘Chasmatosaurus*’ from South Africa and China (e.g. [37,45]).

Our phylogenetic analyses also bolster the occurrence of crown archosaurs in upper Olenekian beds of Russia and recovered *Vytshegdosuchus zheshartensis* as the oldest known avemetatarsalian body fossil. The ghost lineage of Avemetatarsalia already extended back into the late Olekenian because of the occurrence of pseudosuchians of this age [28,143,170–172] and Olenekian ichnofossils have been previously suggested as referable to the bird-line of Archosauria [173,174]. *Vytshegdosuchus zheshartensis* opens the possibility that avemetatarsalians were present in the same assemblages as the youngest chasmatosuchines or occur shortly after them. This uncertainty depends on the interpretation of taxa such as *Jaikosuchus magnus*, *Tsylmosuchus* spp. and *Gamosaurus lozovskii* as proterosuchids. The occurrences of both major clades of Archosauria by the late Olenekian are congruent with the idea of a mainly cryptic phylogenetic diversity of archosauromorphs that remains to be discovered [6].

Several Early Triassic archosauromorphs, including multiple proterosuchid species, are the most topologically unstable terminals of our phylogenetic analyses. This is partially driven by high proportions of missing data, as well as character states that are homoplastic with other archosauromorph clades (e.g. aphanosaurian and poposauroid archosaurs). Thus, the search for and discovery of new more complete proterosuchid specimens will be crucial to reaching a more robust understanding of the anatomy, taxonomy, phylogeny and macroevolution of this clade and its implications for the understanding of the aftermath of the end-Permian mass extinction. The study of *Samsarasuchus pamelae* and the other early archosauriform bones from the Panchet Formation has improved our knowledge of proterosuchids, confirming the high potential of the Indian unit to shed light on the aftermath of the end-Permian mass extinction and, in particular, on the taxonomy and phylogeny of early archosauriforms.

## Data Availability

All data associated with this work is included in electronic supplementary material [186]. R code and associated data files for the regression analyses and SCTs of the different analyses are provided in two zip folders.
